# A Species-Level Phylogeny of Extant Snakes with Description of a New Colubrid Subfamily and Genus

**DOI:** 10.1371/journal.pone.0161070

**Published:** 2016-09-07

**Authors:** Alex Figueroa, Alexander D. McKelvy, L. Lee Grismer, Charles D. Bell, Simon P. Lailvaux

**Affiliations:** 1 Department of Biological Sciences, University of New Orleans, New Orleans, LA, United States of America; 2 Department of Biology, The Graduate School and Center, City University of New York, New York, NY, United States of America; 3 Department of Biology, 6S-143, College of Staten Island, 2800 Victory Boulevard, Staten Island, NY, United States of America; 4 Department of Biology, La Sierra University, 4500 Riverwalk Parkway, Riverside, CA, United States of America; National and Kapodistrian University of Athens, GREECE

## Abstract

**Background:**

With over 3,500 species encompassing a diverse range of morphologies and ecologies, snakes make up 36% of squamate diversity. Despite several attempts at estimating higher-level snake relationships and numerous assessments of generic- or species-level phylogenies, a large-scale species-level phylogeny solely focusing on snakes has not been completed. Here, we provide the largest-yet estimate of the snake tree of life using maximum likelihood on a supermatrix of 1745 taxa (1652 snake species + 7 outgroup taxa) and 9,523 base pairs from 10 loci (5 nuclear, 5 mitochondrial), including previously unsequenced genera (2) and species (61).

**Results:**

Increased taxon sampling resulted in a phylogeny with a new higher-level topology and corroborate many lower-level relationships, strengthened by high nodal support values (> 85%) down to the species level (73.69% of nodes). Although the majority of families and subfamilies were strongly supported as monophyletic with > 88% support values, some families and numerous genera were paraphyletic, primarily due to limited taxon and loci sampling leading to a sparse supermatrix and minimal sequence overlap between some closely-related taxa. With all rogue taxa and *incertae sedis* species eliminated, higher-level relationships and support values remained relatively unchanged, except in five problematic clades.

**Conclusion:**

Our analyses resulted in new topologies at higher- and lower-levels; resolved several previous topological issues; established novel paraphyletic affiliations; designated a new subfamily, Ahaetuliinae, for the genera *Ahaetulla*, *Chrysopelea*, *Dendrelaphis*, and *Dryophiops*; and appointed *Hemerophis* (*Coluber*) *zebrinus* to a new genus, *Mopanveldophis*. Although we provide insight into some distinguished problematic nodes, at the deeper phylogenetic scale, resolution of these nodes may require sampling of more slowly-evolving nuclear genes.

## Introduction

Phylogenies form the cornerstone of our understanding of evolutionary relationships between organisms and provide a historical basis for testing and inferring ecological and evolutionary processes [1–4]. Although phylogenetic methodologies have witnessed an explosion of advancements, estimating large trees remains costly, time-intensive, and computationally difficult. Thus, most analyses have concentrated on resolving the relationships of smaller taxonomic groups, culminating in the accumulation of published sequences available for compiling into larger datasets, or "super-matrices" [5,6]. Coalescent-based species-trees methods are currently favored over concatenated approaches owing to their greater accuracy, but their use for large datasets is still impractical [7,8]. Consequently, many researchers rely on the supermatrix approach [9] or on shortcut coalescence methods [10]. The supermatrix uses concatenated sequences to estimate large-scale phylogenies with branch lengths [11–17]. This technique has earned criticism because large amounts of missing data may obscure phylogenetic signal, leading to uncertainty in topology and branch lengths [18–21], but shortcut coalescence methods are also prone to these same shortcomings [10]. However, several studies have shown that concatenated procedures may nonetheless produce similar results to species-trees [8,22], particularly when there is no agreement among gene trees, and between gene and species trees [7]. This is also the case for deep divergences because shortcut coalescence has difficulty integrating gene-tree incongruity at this level [10]. Our goal for this study was to estimate a species-level phylogeny for snakes using the supermatrix technique.

To date, only two studies have estimated a species-level phylogeny of snakes [15,23], with the latter adding more independent loci to the dataset of the former. These studies featured 1262 known snake species, integrated as part of a larger phylogeny focusing on Squamata, accounting for merely 39% of the total snake diversity at the time. At greater than 3,500 species [24], over a thousand more than the estimate provided by Heise *et al* [25] two decades earlier, and with the recent recognition of new families and subfamilies [26–31], phylogenetic estimates of the snake tree of life are markedly underrepresented. Indeed, the first phylogenetic analysis including all families and subfamilies was only recently completed [32], and only included one representative from each rank. Over the years, researchers have emphasized resolving higher-level snake relationships [15,22,23,25,27,32–49], and topology within families: typhlopids [26,29,31,50]; boids [30,51–53]; acrochordids [54]; xenodermatids [55]; homalopsids [56,57]; pareatids [58]; viperids [59–61]; elapids and lamprophiids [28,62–64]; dipsads [65,66]; pseudoxendontids [67]; natricines [68]; sibynophiids [27]; and colubrids [39,40]. Despite these efforts, many unresolved nodes remain scattered throughout the entire snake tree, such as the monophyly of Scolecophidia [15], topology of Typhlopinae [29], monophyly of Cylindrophiidae and Anomochilidae [35], topology of Booidea [30,53], placement of Xenophidiidae and Bolyeridae [53], and several issues within Caenophidia [22,39,40]. With higher-level relationships of snakes still not settled, our understanding of the snake tree of life remains incomplete.

Although snakes have received a great deal of attention from biologists [69–71], studies of snake biology from comparative and evolutionary perspectives are scarce relative to other reptile taxa such as lizards, in part because of the lack of comprehensive and well-supported snake phylogenies. Estimating a clade-wide species-level phylogeny for snakes with utility for testing evolutionary hypotheses will greatly augment our knowledge of snake biology. Here, we present an updated hypothesis on extant snake phylogeny with increased sampling using the supermatrix approach comprising 1745 taxa (1652 snake species + 7 outgroup taxa), representing 46.33% of the currently known snake species from all known families and subfamilies (Table 1), an increase of 7.24% from Pyron *et al* [15] and Zheng and Wiens [23]. Accepting this tree, we discuss higher-level relationships and highlight taxonomic issues at the genus-level.

**Table 1:** Number of taxa sampled per family or subfamily. Families are listed in order according to Fig 1. For the taxonomy of families and subfamilies, we use Adalsteinsson *et al*, [26] for Anomalepididae and Leptotyphlopidae, Pyron and Wallach [29] for Gerrhopilidae, Typhlopidae, and Xenotyphlopidae, Pyron et al [30] for Booidea, and Pyron *et al* [15] for Alethinophidia. The number of species per clade was taken from The Reptile Database (http://www.reptile-database.org/) on 10/01/2015. Percentages of the number of species sampled do not include taxa not assigned to species status. Paraphyletic taxa are included under their traditional family and/or subfamily. In the Total cell for total number of species, the number not in parentheses equals the sum of the values in the table and the number in the parentheses equals the number returned when a search for Serpentes is conducted in The Reptile Database. Percentage for total number of species sampled is based on 3566 species.

Clade	Number of Species Sampled (% Sampled)	Total Number of Species
**Scolecophidia**		
Anomalepididae	2 (11%)	18
Leptotyphlopidae	—	—
Epictinae	17 (23%)– 2 sp.	64
Leptotyphlopinae	18 (36%)	50
Gerrhopilidae	2 (11%)	18
Xenotyphlopidae	2 (100%)– 1 sp.	1
Typhlopidae		
Typhlopinae	52 (52%)– 19 sp.	64
Afrotyphlopinae	19 (26%)– 3 sp.	61
Madatyphlopinae	2 (15%)	13
Asiatyphlopinae*	49 (33%)– 8 sp.	124
**Alethinophidia**		
Aniliidae	1 (100%)	1
Tropidophiidae	10 (29%)	34
Calabariidae	1 (100%)	1
Candoiidae	3 (60%)	5
Sanziniidae	3 (75%)	4
Charinidae		
Charininae	3 (75%)	4
Ungaliophiinae	3 (100%)	3
Erycidae	9 (75%)	12
Boidae	24 (80%)	30
Cylindrophiidae	2 (15%)	13
Anomochilidae	1 (33%)	3
Uropeltidae	15 (28%)– 1 sp.	54
Xenopeltidae	1 (50%)	2
Loxocemidae	1 (100%)	1
Pythonidae	32 (80%)	40
Bolyeridae	1 (50%)	2
Xenophidiidae	1 (50%)	2
Acrochordidae	3 (100%)	3
Xenodermatidae	4 (22%)	18
Pareatidae	16 (80%)	20
Viperidae		
Viperinae	66 (67%)	98
Azemiopinae	1 (50%)	2
Crotalinae	190 (82%)– 1 sp.	231
Homalopsidae	26 (47%)– 1 sp.	53
Lamprophiidae		
Psammophiinae	45 (87%)– 3 sp.	52
Prosymninae	5 (31%)	16
Pseudaspidinae	2 (100%)	2
Atractaspidinae	7 (30%)	23
Aparallactinae	11 (23%)	47
Lamprophiinae	31 (43%)	72
Pseudoxyrhophiinae	61 (64%)– 4 sp.	89
Elapidae	195 (54%)– 1 sp.	358
Colubridae		
Sibynophiinae	6 (55%)	11
Natricinae	110 (47%)– 3 sp.	226
Pseudoxenodontinae	5 (36%)– 1 sp.	11
Dipsadinae	242 (32%)– 2 sp.	754
Grayiinae	3 (75%)	4
Calamariinae	4 (5%)	87
Ahaetullinae **subfam**. **nov**.	27 (48%)	56
Colubrinae	315 (47%)– 3 sp.	670
*Incertae Sedis*	4^†^	22
**TOTAL**	1652 (46.33%)	3549 (3566)

*Number of species of *Xerotyphlops* is included in Asiatyphlopinae.

**Fig 1:**
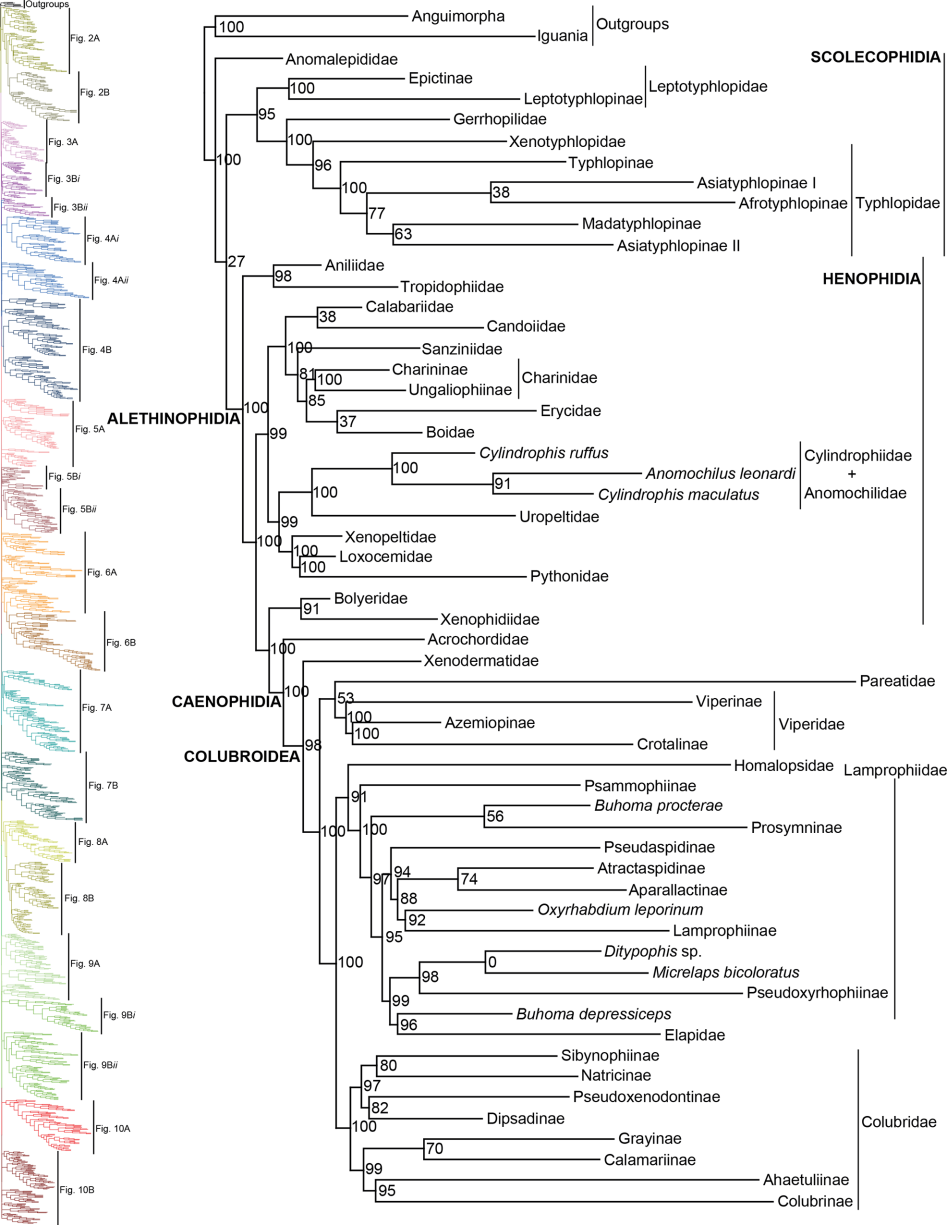
Abridged phylogeny on final dataset of 1652 snake species and seven outgroup taxa displaying higher-level relationships. Maximum-likelihood phylogenetic estimate based on 10 concatenated genes. Tips represent families and sub-families. Commonly recognized higher-level clades are labeled in all caps and bold. Species classified as Lamprophiidae *incertae sedis* are also shown since they did not place within a subfamily. Node values represent SHL support values. Skeleton of the species tree is displayed on the left, colored and labeled as they appear in Figs 2–10.

^†^*Buhoma depressiceps*, *Buhoma procterae*, and *Oxyrhabdium leporinum* are all listed as *incertae sedis* on The Reptile Database, but *Micrelaps bicoloratus* is not. We list these four species as *incertae sedis* because of their variable topological history (see Fig 1).

## Materials and Methods

### Tissue data collection and sequence acquisition

We constructed a dataset of 1745 taxa (1659 species), of which the following seven species represent outgroups: *Calotes versicolor*, *Chamaeleo calyptratus*, *Elgaria multicarinata*, *Heloderma suspectum*, *Liolaemus darwinii*, *Plica plica*, and *Varanus salvator*. The dataset consisted of 9,523 bp from the following 10 genes: three mitochondrial protein-coding genes, cytochrome b (cyt-b; 1,107 bp; 1,398 taxa), NADH subunit 2 (ND2; 1,042 bp; 334 taxa), and NADH subunit 4 (ND4; 802 bp; 986 taxa); two non-coding ribosomal genes (12S; 790 bp; 1,023 taxa) and (16S; 649 bp; 1,167 taxa); and five nuclear protein-coding genes, brain-derived neurotrophic factor precursor (BDNF; 675 bp; 314 taxa), neurotrophin-3 (NT3; 669 bp; 449 taxa), oocyte maturation factor Mos (c-mos; 753 bp; 957 taxa), and two recombination-activating genes (RAG-1.1; 926 bp; 209 taxa, RAG-1.2; 880 bp; 166 taxa; RAG-1.3; 517 bp; 153 taxa), and (RAG-2; 716 bp; 153 taxa). We split RAG-1 into three separate alignments because the majority of sequences did not overlap, but instead formed three separate segments of overlapping sequences. Sequences for seven outgroups and 1591 snake species were downloaded from GenBank (S1 Table). To maximize gene coverage for each species, we combined sequences from multiple individuals of the same species. We sequenced an additional 150 tissue samples from 88 species, of which 61 were not previously sequenced (S2 Table). Eighteen we field collected and 132 we obtained from museum vouchers. For field collected samples, we obtained tissue from tail clips or ventral scale clips using sterilized scissors, from snakes collected in Costa Rica and Singapore. We placed all tissue samples in 90% ethanol under the Alexander D. McKelvy Field Series (ADM). Methods for tissue collection were approved by the University of New Orleans Animal Welfare Committee and by both permitting agencies for each country: Costa Rica, Ministerio del Ambiente y Energía Sistema Nacional de Areas de Conservación, permit ACTo-GASP-PIN-023-2010, and; Singapore, NParks, permit NP/RP11-030. Museum tissue samples represent a combination of liver, muscle, and heart tissue and were gathered from the following museums: AMNH, CAS, FMNH, KU, LSUHC, LSUMNS, MVZ, and YPM (refer to S2 Table for museum codes). Species we sequenced are identified by species name and voucher number (S2 Table). For taxonomic classification, we consulted The Reptile Database (http://www.reptile-database.org/). As of October 2015, the database recognizes 3566 species of snakes. Our dataset accounted for approximately 46.33% of currently recognized snake species.

### DNA extraction, amplification, sequencing, and alignment

We extracted genomic DNA from tissue samples following the standard protocol provided for Qiagen® DNeasy kits. We sequenced six genes: 16S, c-mos, cyt-b, ND4, NT3, and RAG-1. A list of the primers used, their source, and annealing temperatures are provided in S3 Table. We aliquoted a 2 μl portion of each purified DNA extract and combined it with GoTaq Green MasterMix (Promega Corp.), primers from respective gene, and deionized water to create a 10 μl reaction to be used in the Polymerase Chain Reaction (PCR). We placed all PCR reactions on a thermal cycler under the following protocol: 95°C for 2 min; 95°C for 30 s; 50°C for 30 s for 40 cycles; 72°C for 1:15 min; 72°C for 3–5 min; and chilled at 4°C until taken off cycler. Next, we cleaned the PCR products using 1 μL of ExoSap-IT (USB Corp.) per 10 μL of PCR product. We performed cycle sequencing on purified PCR products using 1 μL primer (10 μM), 2 μL template, and 5 μL deionized water along with a Big Dye Terminator 3.1 (Amersham Pharmacia Biotech) reaction premix for 50 cycles of 96°C for 10 s; 45°C for 5 s; and 60°C for 4 min and purified using a Sephdex column, then used an ABI 3130XL Genetic Analyzer to determine nucleotide sequences of each sample.

We aligned all sequences using the default parameters of the Geneious alignment, and refined alignments using the default parameters of the MUSCLE alignment [72] in the program Geneious v4.8.4 (http://www.genious.com; [73]). We then edited alignments by eye and trimmed ambiguous end regions. For some genes, a few species had identical sequences with other taxa so we retained the first taxon in alphabetical order ([15]; S1 Table). Finally, we used Geneious to concatenate all genes to create a supermatrix. This matrix contained 71.41% of missing data; however, previous studies have shown that missing data does not negatively influence topology, branch length estimates, and node support [15,23,40,41]. We deposited all sequences generated from this study in GenBank (S2 Table). The final alignment is available in Phylip format in S1 File.

### Phylogenetic inference

We performed phylogenetic analyses on the 10-gene concatenated matrix using the maximum likelihood (ML) criterion in the program RAxML HpC-2 v8 [74] on the CIPRES portal (http://www.phylo.org; [75]). First, we analyzed each gene separately to check topological congruence by performing rapid bootstrap analyses and pruned misplaced taxa with suspect placement out of the alignment, before concatenating them into the final alignment. The following five species were removed from the alignment due to poor placement for all genes: *Boiga siamensis* FMNH267726, *Chrysopelea ornata* LSUHC7158, *Dipsadoboa werneri*, *Emydocephalus ijmae*, and *Psammodynastes pictus* FMNH267940. We conducted analyses by generating starting trees under the default parsimony model and obtained node support from 100 non-parametric bootstrap replicates using the GTRGAMMA model for all genes and codon partitions since the GTRGAMMA model is recommended over GTR + Γ + I as the 25 rate categories implemented with GTRGAMMA accounts for potentially invariant sites [76]. After concatenating the genes, we performed a rapid bootstrap analysis on the data partitioned by gene and codon position and obtained node support from 1000 non-parametric bootstrap replicates using the GTRGAMMA model.

Rogue taxa can present themselves in phylogenetic estimates due to ambiguous or insufficient phylogenetic signal [77]. These taxa decrease resolution and support in any best tree estimate because they cannot be placed with any confidence anywhere in the tree due to occupying numerous different phylogenetic positions in a set of trees [78]. Thus to produce a more informative best tree estimate with improved clade support, we identified and eliminated rogue taxa with the webserver version of RogueNaRok at http://rnr.h-its.org/submit [79] using the support on best tree estimate threshold, optimizing support, and maximum dropset size of 1. To avoid pruning a large number of taxa, we only pruned 22 taxa that had a random improvement score (i.e., fraction of improvement in bootstrap support values throughout the tree when the selected taxon is pruned and all rogue taxa above it are also pruned) above 0.8 (S4 Table). We acknowledge that excluding additional rogue taxa will improve clade support values, but we wanted to include a maximum number of taxa to estimate a more comprehensive phylogeny. After pruning rogue taxa, the final dataset resulted in 1745 taxa (1659 species). We then performed 10 ML searches on 10 random stepwise addition parsimony-based starting trees using the GTRGAMMA model. Next, we executed a final topology optimization on the best scoring ML tree to produce a nearest-neighbor interchange (NNI)-optimized estimate of the ML tree also using the GTRGAMMA model. Finally, we assessed node support using the non-parametric Shimodaira-Hasegawa-Like (SHL) implementation of the approximate likelihood-ratio test (aLRT; [80]) based on several advantages over other support methods and considered SHL values of 85% or greater as strong support [15]. We also estimated the tree with all rogue taxa from the first analysis and species classified as *incertae sedis*, all within the family Lamprophiidae (*Buhoma depressiceps*, *Buhoma procterae*, *Micrelaps bicoloratus*, and *Oxyrhabdium leporinum*), eliminated to scrutinize their influence on higher-level relationships.

### Nomenclatural acts

The electronic edition of this article conforms to the requirements of the amended International Code of Zoological Nomenclature, and hence the new names contained herein are available under that Code from the electronic edition of this article. This published work and the nomenclatural acts it contains have been registered in ZooBank, the online registration system for the ICZN. The ZooBank LSIDs (Life Science Identifiers) can be resolved and the associated information viewed through any standard web browser by appending the LSID to the prefix “http://zoobank.org/”. The LSID for this publication is: urn:lsid:zoobank.org:pub: 3966804E-D532-4C52-92AC-BECAE776E434. The electronic edition of this work was published in a journal with an ISSN, and has been archived and is available from the following digital repositories: PubMed Central, LOCKSS.

## Results and Discussion

### Higher-level phylogeny

**Fig 2:**
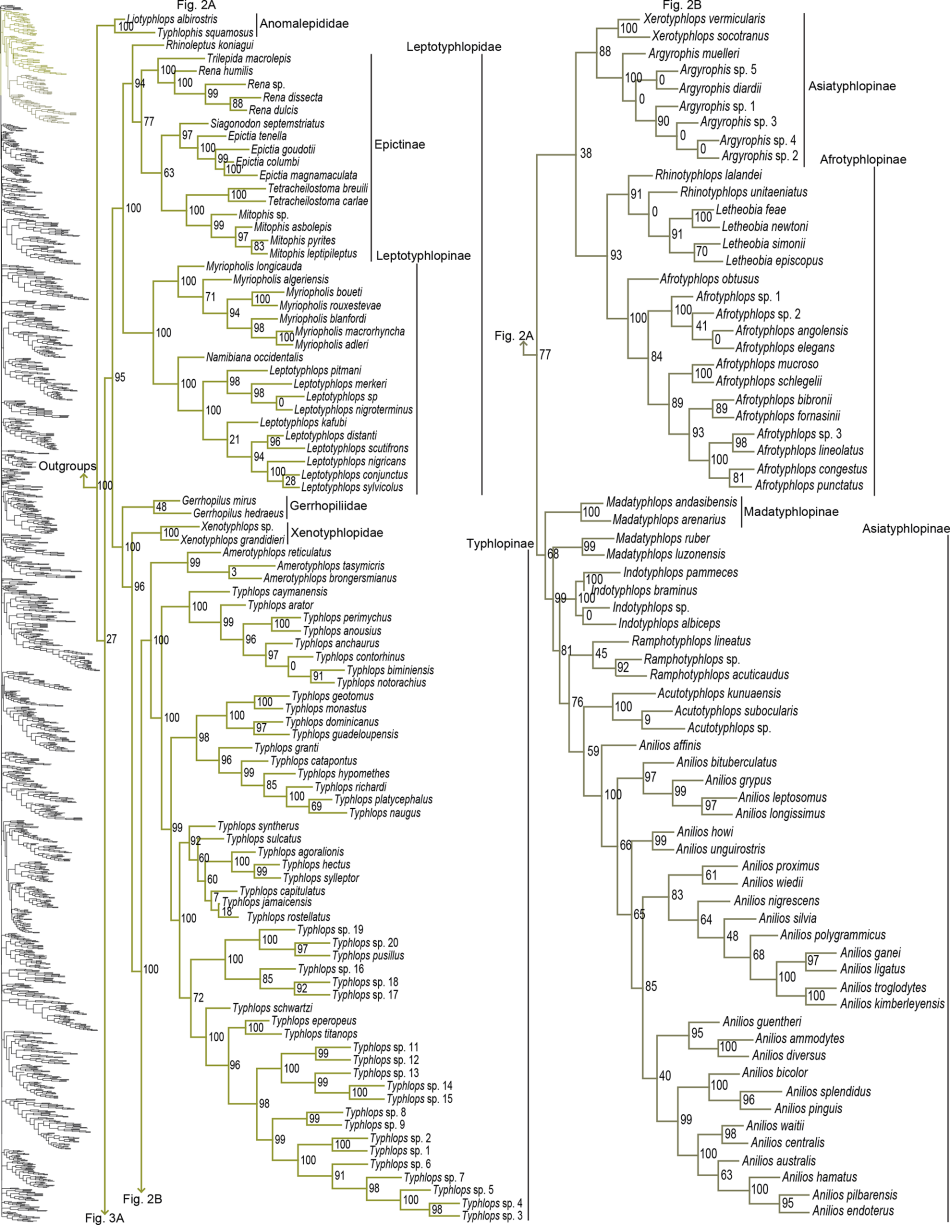
Species-level phylogeny on final dataset of 1652 snake species. Maximum-likelihood phylogenetic estimate based on 10 concatenated genes. Node values represent SHL support values. Seven outgroup taxa are not shown. Colors of clades indicate their position in the overall tree, shown at left. Newly sequenced taxa are highlighted in bold. Skeleton of the species tree is displayed on the left with displayed subfamilies/families highlighted. Letters denoted by i and ii represent parts of the tree where external branches do not connect to the part of the tree immediately preceding it. A) Anomalepididae, Epictinae, Leptotyphlopinae, Gerrhopilidae, Xenotyphlopidae, and Typhlopinae. B) Asiatyphlopinae I, Afrotyphlopinae; Madatyphlopinae, and Asiatyphlopinae II.

**Fig 3:**
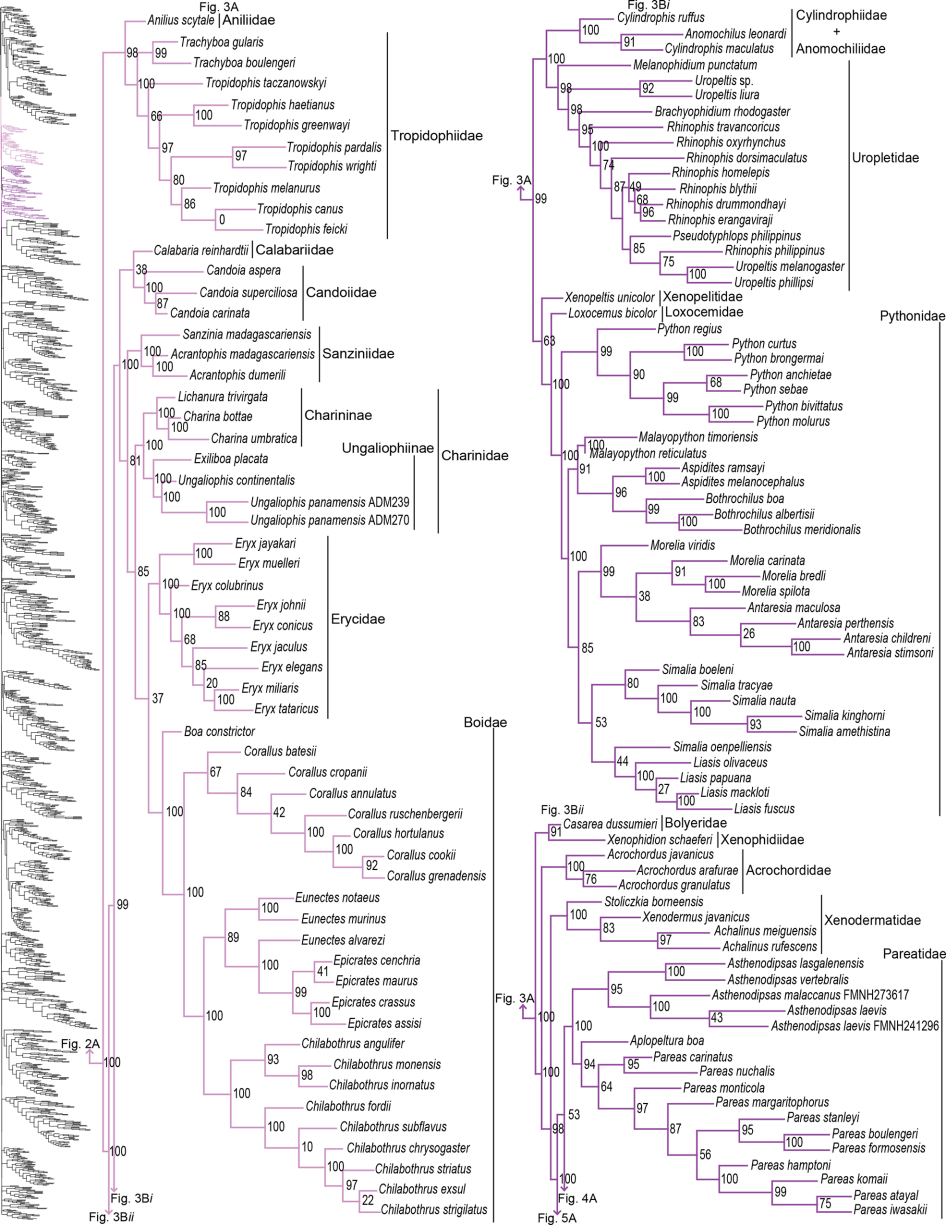
Phylogenetic tree of Serpentes continued. A) Aniliidae, Tropidophiidae, Calabariidae, Candoiidae, Sanziniidae, Charininae, Ungaliophiinae, Erycidae, and Boidae. B*i*) Cylindrophiidae + Anomochilidae, Uropeltidae, Xenopeltidae, Loxocemidae, and Pythonidae. B*ii*) Bolyeridae, Xenophidiidae, Acrochordidae, Xenodermatidae, and Pareatidae.

**Fig 4:**
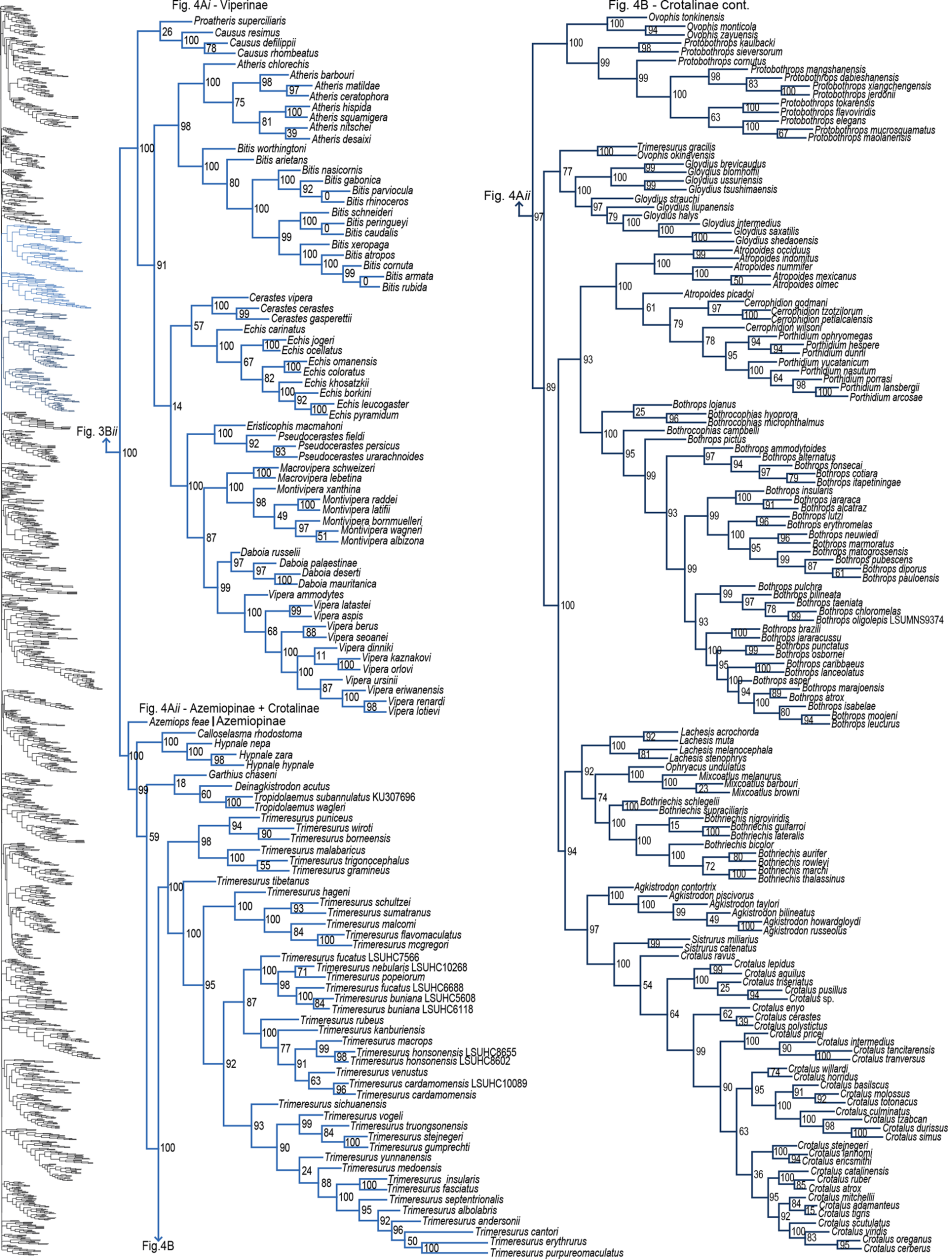
Phylogenetic tree of Serpentes continued. A*i*) Viperinae. A*ii*) Azemiopinae and Crotalinae. B) Crotalinae continued.

**Fig 5:**
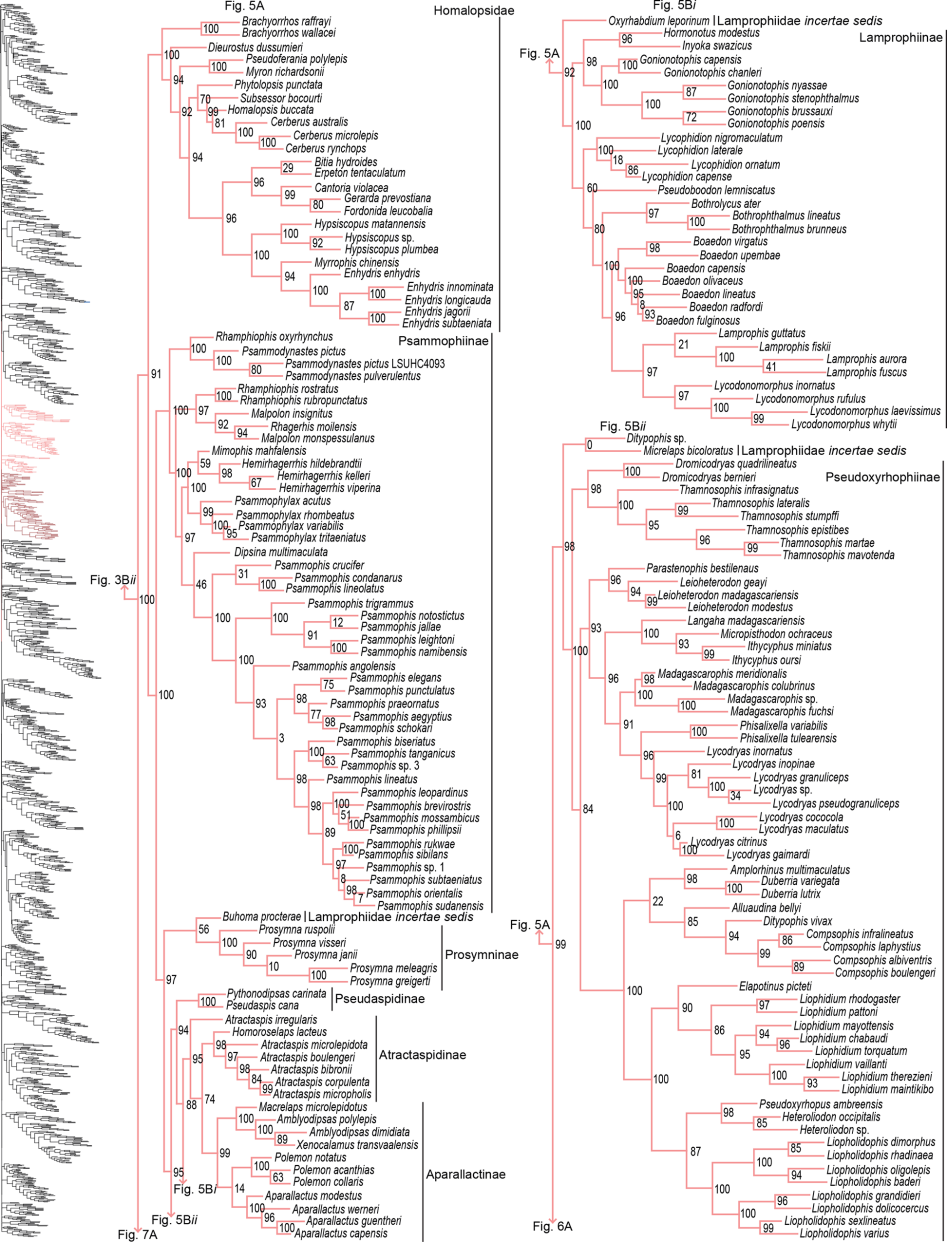
Phylogenetic tree of Serpentes continued. A) Homalopsidae, Psammophiinae, *Buhoma procterae*, Prosymninae, Pseudaspidinae, Atractaspidinae, and Aparallactinae. B*i*) *Oxyrhabdium leporinum* and Lamprophiinae. B*ii*) *Ditypophis* sp. + *Micrelaps bicoloratus* and Pseudoxyrhophiinae.

**Fig 6:**
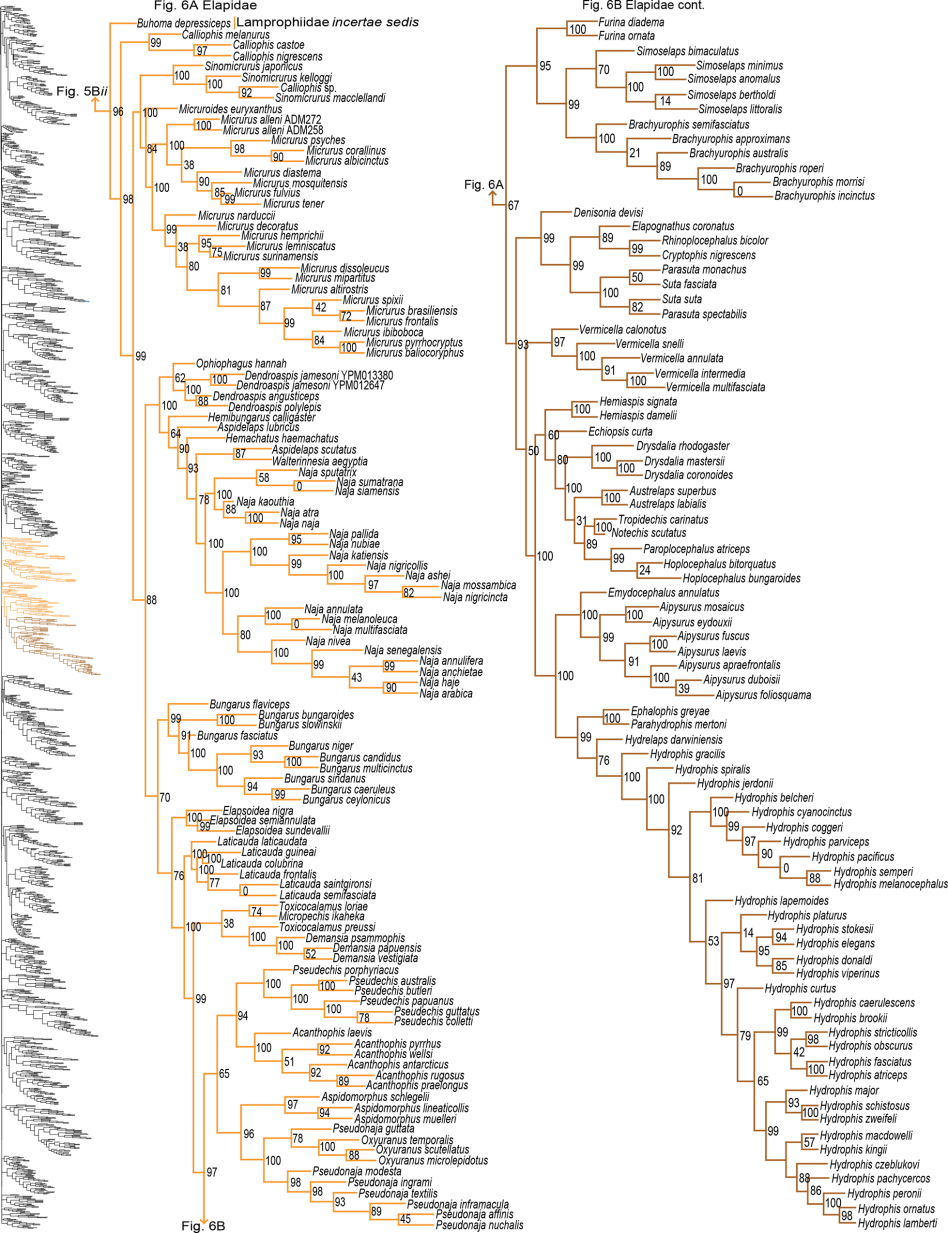
Phylogenetic tree of Serpentes continued. A) *Buhoma depressiceps* and Elapidae. B) Elapidae continued.

**Fig 7:**
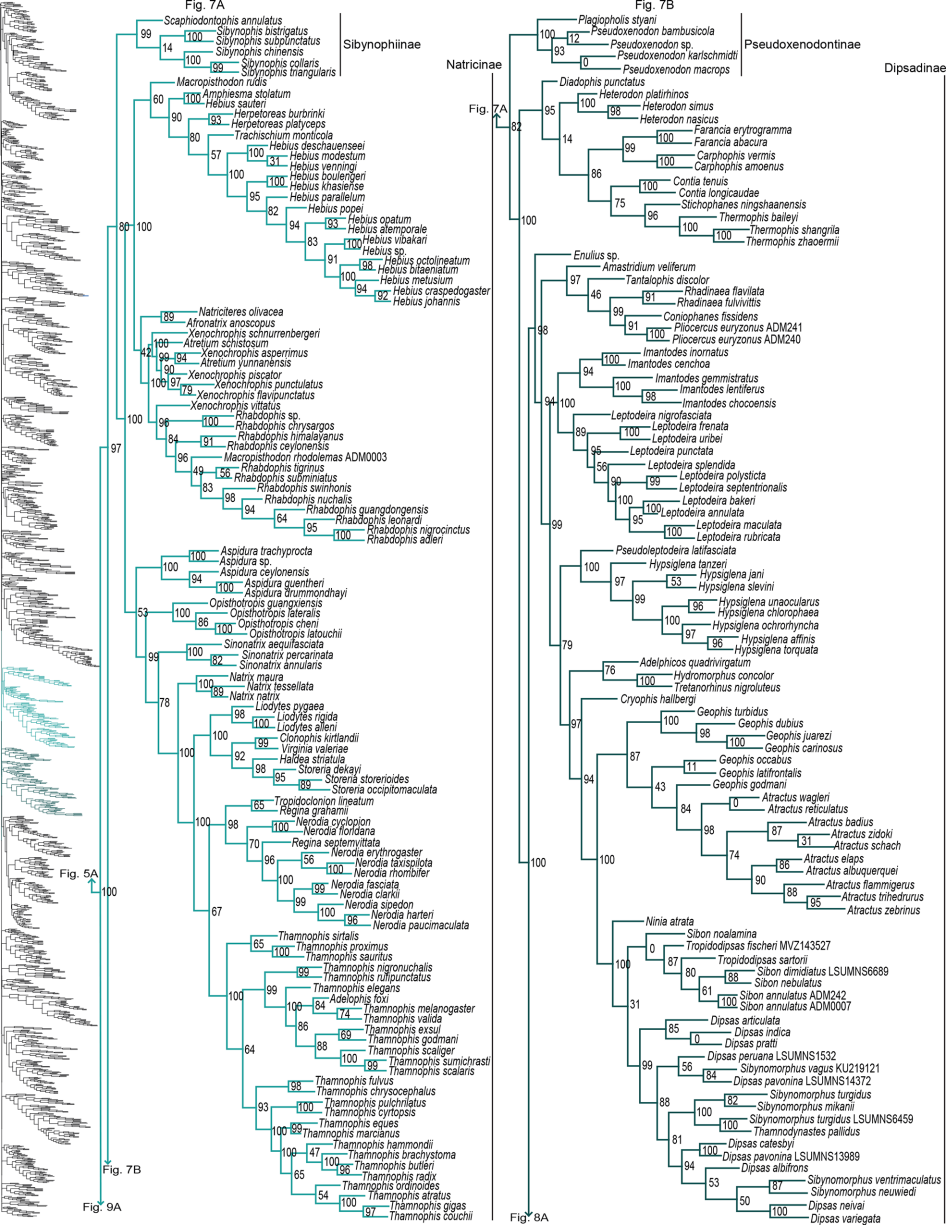
Phylogenetic tree of Serpentes continued. A) Sibynophiinae and Natricinae. B) Pseudoxenodontinae and Dipsadinae.

**Fig 8:**
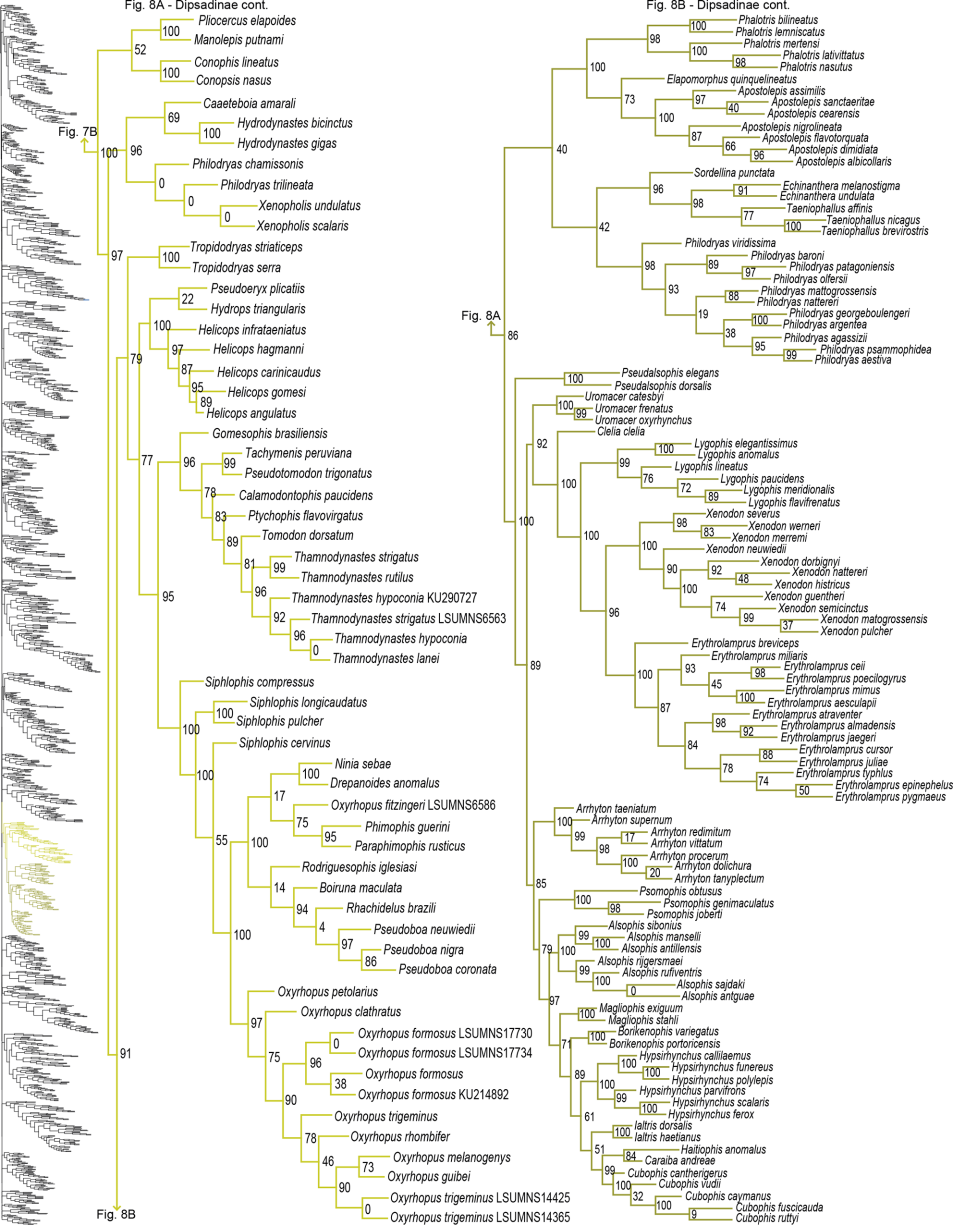
Phylogenetic tree of Serpentes continued. A) Dipsadinae continued. B) Dipsadinae continued.

**Fig 9:**
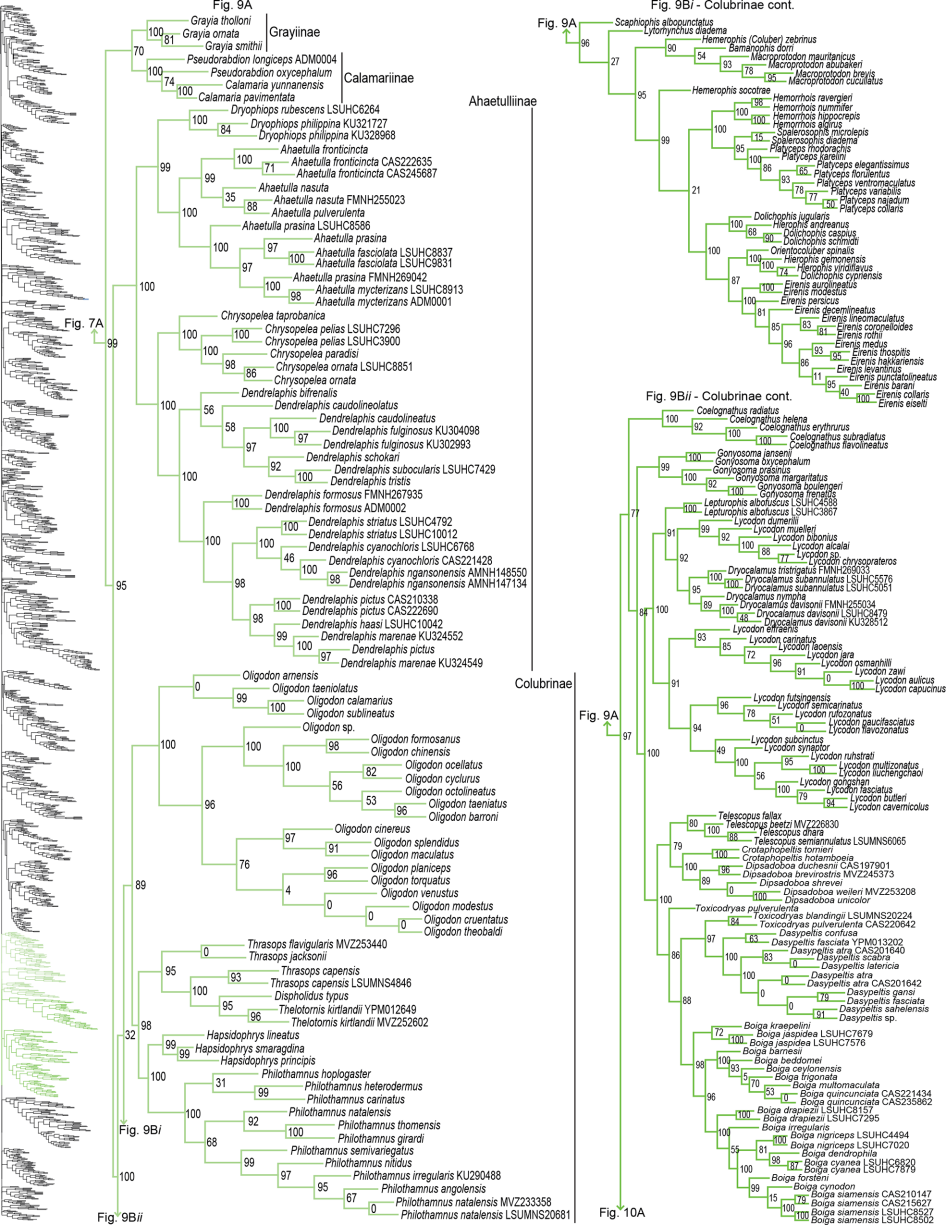
Phylogenetic tree of Serpentes continued. A) Grayiinae, Calamariinae, Ahaetullinae **subfam. nov.**, and Colubrinae. B*i*) Colubrinae continued. B*ii*) Colubrinae continued.

**Fig 10:**
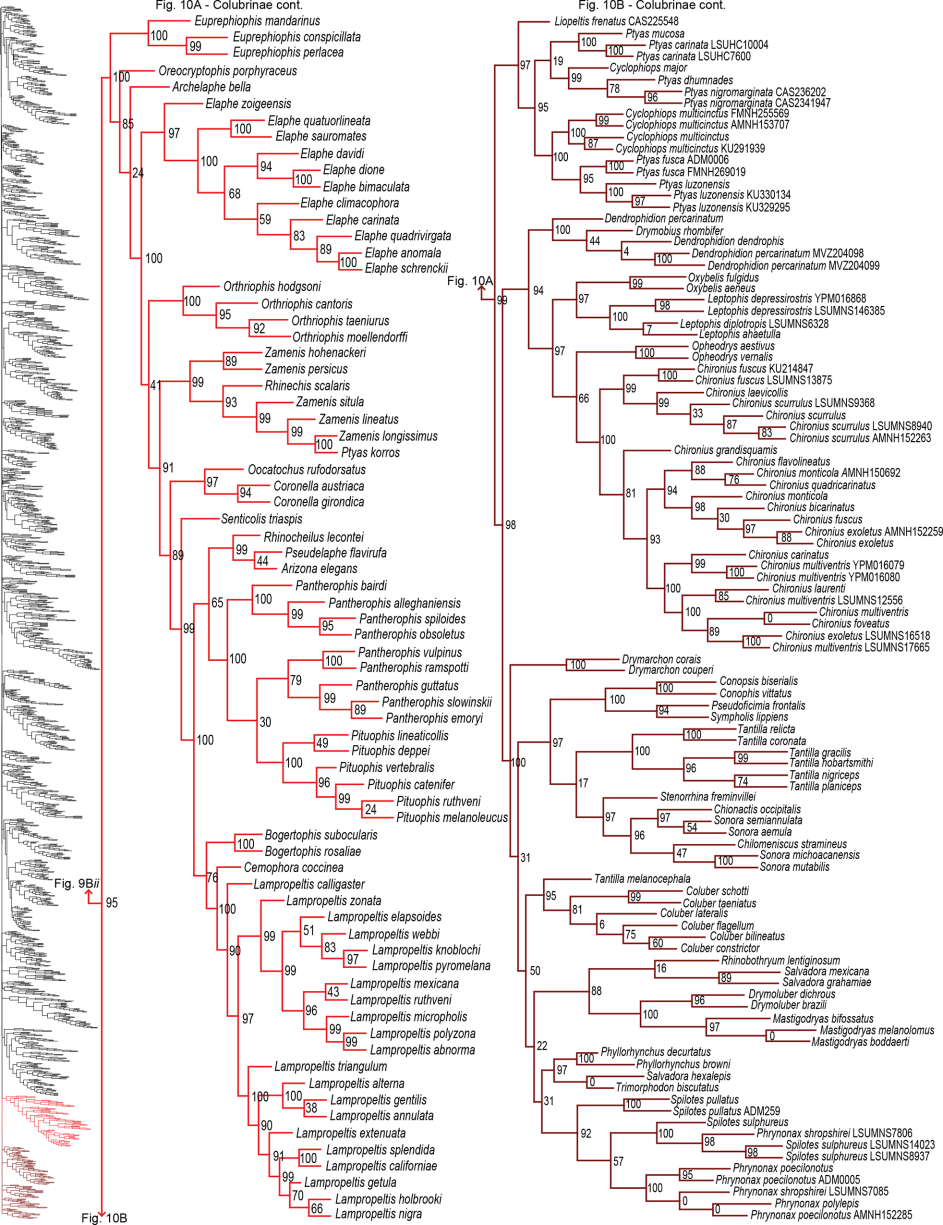
Phylogenetic tree of Serpentes continued. A) Colubrinae continued. B) Colubrinae continued.

As in previous studies, we find very strong support (SHL = 100) for the clade Serpentes [15,23,36,42,48,81]. In Fig 1 we display a summary of the full ML tree (lnL = -919390.188) to exhibit relationships above the genus-level and present the full species-level tree in Figs 2–10, made available in Newick format in S2 File. Overall, more than half of the nodes in the full species-tree received strong support (73.45% of nodes with SHL values > 85). In the following section we largely compare our tree to Pyron *et al* [15], since they provide a recent detailed comparison to preceding publications and because theirs is the only other clade-wide species-level tree (but see [23]). In general, we substantiate many of the higher-level relationships reported in Pyron *et al* [15]; however, several differences also exist. Support for monophyly for each family and subfamily was above 88%, except for Gerrhopilidae (SHL = 48), and Cylindrophiidae was paraphyletic with Anomochilidae [23,35,53].

#### Scolecophidia

Similar to many prior examinations, we find relationships within Scolecophidia unresolved [15,23,25,31,32,41,42,46–48,82–85], with studies showing either Scolecophidia [25,31,84,85], Anomalepididae [15,41] or Leptotyphlopidae + Typhlopoidea [23,42,46,47,48] as sister to all snakes. Morphology also reveals uncertainty surrounding Scolecophidia (reviewed in [84]), but based on the presence of vestigial supratemporal and ectopterygoid bones, absent in other scolecophidians, Anomalepididae may be the most basal scolecophidian [85]. We believe future work will lead to a reclassification of Scolecophidia, but until then relationships within the infraorder remain problematic. In addition, we find weak support for the placement of Asiatyphlopinae, Afrotyphlopinae, and Madatyphlopinae within Typhlopidae as in previous studies [15,23,29,31,50,86]. The issue appears to lie primarily with the placement of *Argyrophis* [50] and *Xerotyphlops* [15,23,50], which together formed Asiatyphlopinae I. *Xerotyphlops* is represented by two species, one occurring in the eastern Mediterranean and the other on Socotra Island [86], and *Argyrophis* is distributed from western Asia to Southeast Asia [29,86]. Discordance in topology therefore appears associated with these two genera being intermediate in distribution between African and Asian typhlopids, which may show affinities to clades from both regions.

#### Henophidia

As mentioned above, Cylindrophiidae is paraphyletic with Anomochilidae. Difficulty in resolving this relationship is likely due to the representation of *Anomochilus* by one species and two genes (12S and 16S), and *Cylindrophis* by two species with greater gene coverage. Both of these families were formerly shown as part of or paraphyletic with Uropeltidae [41,42,47,48]. Based on the history of paraphyly between these families, Burbrink and Crother [84] recommended synonymizing Cylindrophiidae and Anomochilidae with Uropeltidae to resolve these families. However, we recommend retaining the current classification until more species are sampled (Table 1) on the grounds that Cylindrophiidae + Anomochilidae share morphological features not present in Uropeltidae [35,84] and since strong support has been shown distinguishing them from Uropeltidae [15,23,32,41]. For boids, our analysis validates the taxonomic changes made in Pyron *et al* [30], but differs in topology from previous assessments in the placement of Calabariidae, Candoiidae, and Sanziniidae [15,23,53]. Although the relationship Erycidae + Boidae is recovered in all studies [15,23], except one [53], support for this relationship is low. Thus, the only node we can have confidence in is the one joining Charininae and Ungaliophiinae [15,23,53].

#### Xenophidiidae and Bolyeridae

Perhaps the most notable difference from the topology of Pyron *et al* [15] was the placement we recovered for Xenophidiidae + Bolyeridae (SHL = 91). Earlier studies showed them as sister to various clades within Henophidia [23,32,38,41,42], but we found very strong support (SHL = 100) for them as sister to Caenophidia (SHL = 100), as also shown in other studies [53,85]. In addition, these snakes possess morphological characters, particularly within the palate, bolstering their close relationship with Caenophidia and not to Henophidia [85]. Pyron *et al* [15] is the only study showing a disassociation between these families placing Xenophidiidae as sister to Alethinophidia, with the exception for Aniliidae + Tropidophiidae, and Bolyeridae as sister to Booidea. Currently, both clades are represented by one species and Xenophidiidae by only one gene (cyt-b). Both clades contain two species; for Xenophidion, both species are known only from one specimen each, and for Bolyeridae, *Bolyeria* is extinct, and *Casarea* is rare [38], so obtaining additional sequences for either clade is unlikely. If this placement is retained, then Caenophidia should be redefined to include Xenophidiidae and Bolyeridae, or they should be given their own taxonomic grouping.

#### Caenophidia

Pyron *et al* [22] recently reviewed and attempted to resolve several problematic issues within Caenophidia. The major problems hindering resolution of this clade are 1) placement of Xenodermatidae inside or outside of Colubroidea; 2) placement of Homalopsidae; 3) topology of Lamprophiidae; and 4) topology of Colubridae. Previous studies have placed Xenodermatidae as sister to Acrochordidae [15,37] or as basal in Colubroidea [23,27,40,42,47,87], have placed Homalopsidae as sister to Lamprophiidae + Elapidae [15,27,40] or as sister to (Lamprophiidae + Elapidae) + Colubridae [23,32,39,42,45,47], and have shown conflicting topologies for the subfamilies within Lamprophiidae and Colubridae [15,23,27,28,37,40,45,47]. Pyron *et al* [22] used seven methods to examine these relationships showing Xenodermatidae as basal in Colubroidea with varying support and Homalopsidae as sister to (Lamprophiidae + Elapidae) + Colubridae with strong support. However, they expressed little confidence in resolving the topology within Lamprophiidae and Colubridae since several divergences were defined by low support. We confirm their findings that Xenodermatidae is sister to the rest of Colubroidea (SHL = 100) and that relationships within Lamprophiidae and Colubridae remain unresolved, but our findings for the placement of Homalopsidae contradicted theirs, as we recovered strong support (SHL = 91) for Homalopsidae + Lamprophiidae, and found Elapidae to be nested within Lamprophiidae. Typically, Lamprophiidae and Elapidae are recovered as distinct clades [15,22,28,39,40,41,64], but we found strong support (SHL = 96) for Elapidae + *Buhoma depressiceps* as sister to Pseudoxyrhophiinae (SHL = 99), shown previously only in Pyron and Burbrink [32]. The topology of Lamprophiidae is complicated by the presence of several *incertae sedis* taxa (see Lamprophiidae [28,32,39,41]), but Elapidae remains nested within Lamprophiidae even when these taxa are removed (S1 Fig). In addition, we found the placement of Pareatidae and Viperidae within Colubroidea unresolved. Pareatidae is consistently placed as sister to Viperidae, which is sister to Colubridae, Elapidae, Homalopsidae, and Lamprophiidae [15,22,23,27,32,41,42]. A possible explanation for this is that our dataset includes the greatest sampling of pareatids, adding seven additional species previously not included in higher-level relationships, two we sequenced and five from You *et al* [58].

#### Lamprophiidae

Part of the issue with resolving the topologies within Lamprophiidae, and within Colubridae, is that they exemplify rapid radiations manifested by the presence of short internodes [22]. Yet another major issue hindering progress within Lamprophiidae is the presence of several *incertae sedis* taxa, not identified as rogue taxa by RogueNaRok. These taxa constantly show contrasting phylogenetic placement between studies [15,23,28,39,40,64,87]. We are reluctant in placing any confidence in the topology between subfamilies recovered for Lamprophiidae, despite high support values. However, the topology after all rogues and *incertae sedis* taxa were pruned remained essentially the same (S1 Fig) adding supplementary support for this topology. Nonetheless, our topology differs from earlier studies. Previous studies have consistently recovered the sister relationship between Aparallactinae + Atractaspidinae [15,22,28,32,39,40,41,64]; however, we found this relationship unresolved, likely due to the strong placement (SHL = 95) of *Atractaspis irregularis* as sister to these two clades, and this taxon is represented by only one gene. The topology recovered here was Psammophiinae + ((*B*. *procterae* + Prosymninae) + (Pseudaspidinae + (Atractaspidinae + Aparallactinae) + (*O*. *leporinum* + Lamprophiinae)) + (((*Ditypophis* sp. + *M*. *bicoloratus*) + Pseudoxyrhophiinae) + (*B*. *depressiceps* + Elapidae)))). All nodes received strong support (SHL > 88), except for subclades *B*. *procterae* + Prosymninae and *Ditypophis* sp. + *M*. *bicoloratus*. Pyron *et al* [15] had augmented the definition of Pseudaspidinae to include *Buhoma* and *Psammodynastes*. With added sampling of *Psammodynastes*, we recovered this genus as paraphyletic with *Rhamphiophis oxyrhynchus* (SHL = 100) within Psammophiinae, making *Rhamphiophis* paraphyletic (Fig 5A). *Buhoma*, on the other hand, was split with *B*. *procterae* sister to Prosymninae and *B*. *depressiceps* sister to Elapidae. *Oxyrhabdium leporinum* was sister to Lamprophiinae and *Micrelaps bicoloratus* was placed within Pseudoxyrhophiinae. In all preliminary and final analyses, *Psammodynastes* constantly occupied the same phylogenetic position; however, placement of the other four species was erratic and always differed. Therefore, we tentatively include *Psammodynastes* as part of Psammophiinae. Due to their perpetual variable placement, we continue recognizing *Buhoma*, *M*. *bicoloratus*, and *O*. *leporinum* as Lamprophiidae *incertae sedis*.

#### Colubridae

For Colubridae, we recovered the following four subclades: i) Sibynophiinae + Natricinae (SHL = 80); ii) Pseudoxenodontinae + Dipsadinae (SHL = 82); iii) Grayiinae + Calamariinae (SHL = 70); and iv) Ahaetuliinae **subfam. nov.** + Colubrinae (SHL = 95). The nodes between these subclades all received very strong support (SHL > 97). The only consistently recovered clade among these is subclade ii [22,27,32,40,41]; although other studies do not recover this subclade [15,23,65]. Several studies also regularly recovered the subclade Natricinae + (Pseudoxenodontinae + Dipsadinae) [22,27,32,40], but we do not uncover that relationship here. Instead, Natricinae formed a subclade with Sibynophiinae, also reported in [41]. The subfamily Sibynophiinae was only recently included in molecular analyses, originally grouped with Calamariinae [27], then subsequently placed as sister to Grayiinae + Colubrinae [15,23], and to Calamariinae + (Colubrinae + Grayiinae) [22]. The subfamily Grayiinae was also recently described [45] and grouped with Calamariinae in that study, also recovered in Pyron and Burbrink [32]. However, Grayiinae has most frequently been grouped with Colubrinae [15,22,23,27,39–41]. Dipsadinae is exclusively a New World family, but recent placement of *Stichophanes* and *Thermophis* as sister to Dipsadinae [15,88,89] expanded its distribution into the Old World. Pyron *et al* [15] did not include *Stichophanes*, and they mentioned that *Thermophis* may even warrant its own subfamily. However, our results do not uphold this view since we show *Stichophanes* + *Thermophis* (SHL = 96; Fig 7B) as placed within Dipsadinae. Wang *et al* [89], on the other hand, supported *Stichophanes* + *Thermophis* as sister to Dipsadinae, but their dataset was not as extensive and did not include *T*. *zhaoermii*. Until now, the basal node of Colubrinae has remained ambiguous. Pyron *et al* [15] suggested that monophyly of *Ahaetulla*, *Chrysopelea*, and *Dendrelaphis* at the base of Colubrinae, may warrant recognition as a distinct subfamily, but support for division of these taxa in their study was low. Due to increased sampling, and the inclusion of *Dryophiops*, we established strong support for recognizing these taxa as a new subfamily, using the name proposed by Pyron *et al* [15], Ahaetuliinae **subfam. nov.**

#### Higher-level phylogeny with all rogue taxa eliminated

With all rogue taxa (101) and *incertae sedis* species (4) eliminated, higher-level relationships and support values remained relatively unchanged (S1 Fig). Where changes in topology or support values occurred, it was in the problematic clades discussed above, specifically Typhlopidae, Booidea, Pareatidae + Viperidae, Lamprophiidae, and Colubridae. For Typhlopidae, *Xerotyphlops* formed a clade by itself, sister to all other typhlopids. Madatyphlopinae formed a moderately supported (SHL = 87) clade with Typhlopinae. However, the placements of Afrotyphlopinae and Asiatyphlopinae remained unresolved. In Booidea, the placement of Calabariidae + Candoiidae swapped with Sanziniidae, greatly altering support values throughout Booidea, except in Charininae + Ungaliophiinae. Within Colubroidea, the placement of Pareatidae and Viperidae remains unresolved. Interestingly, with *incertae sedis* species removed from Lamprophiidae, topology of the subfamilies and of Elapidae within Lamprophiidae remained the same and the relationship between Atractaspidinae and Aparallactinae was strongly resolved, providing compelling support for the topology recovered. However, the node joining Prosymninae to all other lamprophiids became ambiguous. Relationships within Colubridae remained stable, except that Pseudoxenodontinae placed as sister to all other colubrids. In addition, we note that the sister relationship of Xenopeltidae to Loxocemidae + Pythonidae became ambiguous, and that with the exclusion of Xenophidiidae as a rogue taxon, Bolyeridae still placed as sister to Caenophidia with high support (SHL = 99), upholding its position outside of Henophidia.

### Genus- and species-level phylogeny

Of the 147 samples we sequenced, two genera (*Dryophiops*, and *Liopeltis*) and 61 species were not previously incorporated in any phylogenetic analyses. *Dryophiops* placed within Ahaetullinae **subfam. nov.** as sister to *Ahaetulla* (SHL = 99), and *Liopeltis* fell within Colubrinae as sister taxon (SHL = 97) to *Ptyas* + *Cyclophiops*. We recovered strong support for the phylogenetic placement of 105 of our samples (SHL > 85). For taxa where our sequences resulted in multiple terminals of the same species, the following species were not monophyletic: *Ahaetulla nasuta*, *A*. *prasina*, *Chironius exoletus*, *C*. *fuscus*, *C*. *monticola*, *C*. *multiventris*, *Dasypeltis fasciata*, *Dendrelaphis cyanochloris*, *D*. *marenae*, *Dendrophidion percarinatum*, *Philothamnus natalensis*, *Phrynonax poecilonotus*, *P*. *shropshirei*, *Psammodynastes pictus*, *Sibynomorphus turgidus*, *Spilotes sulphureus*, and *Trimeresurus fucata*. Throughout the entire tree, most genera were monophyletic with varied node support. Space does not allow for exhaustive scrutiny at the generic and species level of our tree with previous publications, although a cursory examination reveals consistency with previous publications. Instead, we focus on assessing the placement of paraphyletic genera, most of which require greater sampling of species and genes, or perhaps individuals, to provide an improved appraisal of their phylogenetic positions.

Paraphyly at the lower-level of the tree emerged due to various reasons. For some clades paraphyly is well-established and confirmed here, more notably in *Brachyophidium*, *Pseudotyphlops*, *Rhinophis*, and *Uropeltis* in Uropeltidae (Fig 3B*i*) [15,41,53,90]; *Ovophis* and *Trimeresurus* in respect to *Ovophis okinavensis* + *Trimeresurus gracilis* as basal to *Gloydius* (Fig 4B) [61,91]; *Adelophis*, *Amphiesma*, *Atretium*, *Nerodia*, *Regina*, *Thamnophis*, *Tropidoclonion*, and *Xenochrophis* in Natricinae (Fig 7A) [15,68,92,93]; and *Dipsas*, *Geophis*, and *Sibynomorphus* in Dipsadinae (Fig 7B) [15,49,65,66]. Additional taxa include: variable placement of *Morelia viridis* (Fig 3B*i*) [15,38,52,94] and *Bothrocophias campbelli* (Fig 4B) [95]; and *Suta* with *Parasuta* (Fig 6B) [15,96]. Clearly, these clades require further inspection. On the other hand, we were able to rectify other paraphyletic taxa with strong support, specifically within Colubrinae: *Boiga*, *Chironius*, *Coronella*, *Crotaphopeltis*, *Dasypeltis*, *Dipsadoboa*, *Hapsidophrys*, and *Philothamnus*, *Rhinechis*, and *Scaphiophis*.

In some taxa, such as *Cerrophidion wilsoni* (Fig 4B), *Atractus irregularis* (Fig 5A), *Ditypophis* sp. (Fig 5B*ii*), *Aspidelaps irregularis* (Fig 6A), *Pseudonaja guttata* (Fig 6A), *Geophis* with *Atractus* (Fig 7B), *Sibon noalamina* (Fig 7B), *Philodryas chamissonis* and *P*. *trilineata* (Fig 8A), *Conophis* and *Conopsis* (Fig 8A & Fig 10B), *Ptyas korros* (Fig 10A), *Tantilla melanocephala* (Fig 10B), and *Salvadora hexalepis* (Fig 10B), sequence overlap with related taxa was zero or minimal. Whereas for the following taxa, their placement were unresolved: Typhlopidae, *Rhinotyphlops unitaeniata* (Fig 2B); Uropeltidae, *Rhinophis philippinus* (Fig 3B); Pythonidae, *Simalia oenpelliensis* (Fig 3B); Viperidae, *Atropoides picadoi* and *Bothrops lojanus* (Fig 4B); Elapidae, *Toxicocalamus loriae* (Fig 6A); Natricinae, *Macropisthodon rhodolemas* ADM0003 (Fig 7A); Dipsadinae, *Oxyrhopus fitzingeri* LSUMNS6586 and *Siphlophis cervinus* (Fig 8A); Calamariinae, *Pseudorabdion oxycephalum* (Fig 9A); and Colubrinae, *Hierophis andreanus* and *Dolichophis cypriensis* (Fig 9B*i*), *Pantherophis* and *Pituophis* (Fig 10A), *Drymobius rhombifer*, *Dendrophidion dendrophis*, *Chilomeniscus stramineus*, *Tantilla melanocephala*, and *Salvadora hexalepis* (Fig 10B).We do not classify *Calliophis* and *Sinomicrurus* as paraphyletic until the identity of *Calliophis* sp. is known.

For some clades, paraphyly was strongly supported allowing us to synonymize these taxa. Within Psammophiinae, we synonymize *Rhagerhis moilensis with Malpolon*. This species consistently forms a monophyletic clade with *Malpolon* [15,28,62,97] (Fig 5A), but two studies [64,98], inaccurately cite Kelly *et al* [62] as providing evidence for their separation. In Aparallactinae, we synonymize *Xenocalamus* with *Amblyodipsas* (Fig 5A), also recovered in Pyron *et al* [15], the only other study including these taxa. Within Colubrinae we synonymize several clades. First, we synonymize *Lepturophis* and *Dryocalamus* with *Lycodon*, which forms a strong clade (SHL = 100) with these taxa strongly embedded within [15,99] (Fig 9B*ii*). Next, we synonymize *Rhinechis scalaris*, a species with an erratic phylogenetic history [100,101], with *Zamenis*, but the addition of more genes shows it related to *Zamenis* [15,102] (Fig 10A), with which it has morphological affinities to [103]. Finally, we also synonymize *Cyclophiops* with *Ptyas*. Previously recovered as sister clades [15,104], our increased sampling for both genera shows that *Ptyas* forms a strong clade (SHL = 95) with the two species of *Cyclophiops* strongly nested within two separate subclades (Fig 10B). Conversely, in other clades paraphyly was strong, but we do not propose taxonomic changes, specifically in *Hebius sauteri* placing with *Amphiesma* (Fig 7A), *Balanophis ceylonensis* within *Rhabdophis* (Fig 7A), *Thamnodynastes pallidus* placing with *Sibynomorphus* (Fig 7B), *Pliocercus* split (Figs 7B & 8A), *Ninia* split (Figs 7B & 8A), *Dispholidus typus* within *Thelotornis* (Fig 9A), *Chionactis occipitalis* placing with *Sonora* (Fig 10B), and *P*. *shropshirei* LSUMNS7806 within *Spilotes* (Fig 10B), mainly because these taxa, or taxa they placed with, are presented for the first time in a phylogenetic analysis.

In the case of *Hemerophis*, after the genus *Bamanophis* was erected for *Coluber dorri* [105], *H*. *zebrinus* remained as the only Old World *Coluber* representative, until it was recently recognized as *Hemerophis* without justification [24,106]. Yet, the two are distantly-related within a clade of Old World racers [15,40,107,108]. *H*. *zebrinus* is typically placed in a clade sister to *Bamanophis* and *Macroprotodon*, but a very recent study incorporating new sequence data for *Rhynchocalamus*, not included here, places *H*. *zebrinus* as the basal lineage within this clade sister to (*Bamanophis* + *Macroprotodon*) and all other Old World racers [109]; while *H*. *socotrae*, occupies a branch away from this clade. Nagy *et al* [108] shows weak support for a sister relationship between the two using maximum parsimony, but shows them separated with greater support using Bayesian inference and ML. Therefore, we create a new genus for *H*. *zebrinus*, *Mopanveldophis* **gen. nov.**

### Supermatrix approach

Despite the utility of the supermatrix approach, this method is also potentially responsible for uncertainty in some nodes. Compiling available molecular data from numerous studies leads to a sparse data matrix with a substantial portion of missing data unequally scattered throughout the alignment due to sampling differences between studies [11]. Our dataset consisted of 71.41% of missing data with several taxa represented by a single gene to taxa with data spanning all loci. Heterogeneity in sparse data matrices can alter topological relationships and negatively impact tree support by increasing the presence of rogue taxa [110]. Rogue taxa typically are characterized by little character data that do not overlap with closely-related taxa [21]. We identified and removed 22 rogue taxa from our data matrix, 12 of which were delineated by one gene and eight by two genes. The genes 12S, 16S, c-mos, and ND4 were most associated with rogue taxa. These genes evolve more slowly and are not adequate for delimiting species-level relationships (see methods), and several families in our tree are only represented by one or two individuals with few sequenced loci (i.e., Anomalepididae, Anomochilidae, Bolyeridae, Cylindrophiidae, and Xenophidiidae; Table 1). Many taxa in the tree with low support were also represented by a single gene. Furthermore, lack of sequence overlap between closely-related species can also lead to misplacement of taxa in the tree, sometimes with high support as mentioned above. However, many taxa with extensive missing data were placed correctly in the tree (e.g., *Chironius multiventris*, *Pseudocerastes urarachnoides*, *Rhabdophis chrysargos*, *Trimeresurus wiroti*), grouping with closely-related taxa with high support, confirming that increased taxon sampling is a favorable choice for improving phylogenetic accuracy [111], even with a high percentage of missing data [112]. This can occur when the overall number of characters in the data matrix is high [5,113–116], especially for SHL support values since they are not negatively affected by the amount of missing data in the data matrix [40].

In many cases, denser sampling influenced phylogenetic relationships and node support [117]. For example, adding 30 samples of 18 species (14 never before sequenced) to Ahaetuliinae, resolved the basal Colubrinae node and distinguished Ahaetuliinae as a new subfamily. Increased taxon sampling also resolved several paraphyletic issues at the generic level, identified new associations of paraphyly, mostly due to poor gene sampling, resulted in new phylogenetic hypotheses for some taxa such as *Scaphiophis*, *Stichophanes* + *Thermophis*, and *Xerotyphlops*, and prompted us to make some taxonomic changes. Moreover, our sequencing contribution resulted in complete or nearly complete taxonomic coverage of several genera, including *Ahaetulla*, *Asthenodipsas*, *Chrysopelea*, *Dendroaspis*, *Dryocalamus*, *Dryophiops*, *Phrynonax*, *Ptyas*, and *Ungaliophis*, and greatly increased representation of species of the speciose genera *Boiga* and *Dendrelaphis*. Nonetheless, many challenges exist to estimating the snake tree of life.

### Taxonomic descriptions

**Subfamily Ahaetuliinae subfam. nov.** urn:lsid:zoobank.org:act: 22C47597-1DEF-45A4-ABAC-11C4911557AD

**Type genus:** *Ahaetulla* Link [118]

**Content:** Four genera containing 56 species. *Ahaetulla* (8 species), *Chrysopelea* (5 species), *Dendrelaphis* (41 species), and *Dryophiops* (2 species).

**Etymology:** From the Sri Lankan language Sinhala, ahaetulla/ahata gulla/as gulla, meaning “eye plucker” or “eye picker” for belief that they pluck out the eyes of humans as accounted by the Portuguese traveler João Ribeiro in 1685 (as cited in [119]).

**Diagnosis and definition:** Snakes of this subfamily are arboreal and are diagnosed by keeled ventral and subcaudal scales (laterally notched in some species), and enlarged posterior grooved fangs lacking in some *Dendrelaphis*. Support for monophyly of this clade is very strong (SHL = 100) as also reported in Pyron *et al* [15]. Ahaetuliinae is further split into two monophyletic groups: 1) *Dryophiops* and *Ahaetulla* (SHL = 96) and; 2) *Chrysopelea* and *Dendrelaphis* (SHL = 100). Diagnostic characteristics of the first group include, elongate and laterally-compressed bodies, elongate heads, 15 smooth mid-body dorsal scale rows, and large eyes with horizontal pupils and well-developed canthus rostralis outfitting these snakes with binocular vision [120]. Features diagnostic of the second group include, slender body, rectangular slightly compressed heads, large eyes with round pupils, 13–17 smooth to weakly-keeled mid-body dorsal scale rows. *Chrysopelea* are celebrated for their unique gliding behavior, whereas *Dendrelaphis* are capable of jumping [121].

**Sister taxon:** Previously placed within Colubrinae, Ahaetuliinae forms a strong (SHL = 95) sister relationship with Colubrinae, also weakly supported by Pyron *et al* [15].

**Distribution:** Members of this subfamily inhabit various habitats, but are mostly associated with forests distributed from Pakistan, Sri Lanka and India, north to Nepal and Bangladesh, eastwards all throughout Southeast Asia to southern China, Philippines, Papua New Guinea, and northeast Australia.

**Remarks:** The name *Ahaetulla* has suffered from a tumultuous nomenclatural history [122]. In addition, members of these genera have historically been grouped with unrelated taxa based on absence or presence of hypapophyses [123,124].

**Genus *Mopanveldophis* gen. nov.** urn:lsid:zoobank.org:act: 3B0CB6A0-1EEC-4512-9E77-B105C22ACABB

**Type species:** *Mopanveldophis zebrinus*.

**Content:** The genus is monotypic containing only the species, *Mopanveldophis zebrinus*.

**Etymology:** The generic nomen *Mopanveldophis* is derived from the word “mopanveld”, the name of the type of habitat the specimens were found in, and the Greek adjective *ophis*, meaning “snake”. This name refers to veld habitat distributed in Southern Africa, from the Afrikaans word “field”, that is dominated by the mopane tree, *Colophospermum mopane*, from the Sechuana word “mopani”.

**Diagnosis and definition:** As described in Broadley and Schätti [125] and Bauer *et al* [126], a snake with pale grey dorsal coloration and irregular broad, dark crossbands becoming faint in coloration posteriorly and on tail. Ventrals are uniform white with irregular lateral black spots, and subcaudals are also white with lateral grey stippling. Dorsal portion of head is uniform grey-brown with yellowish orange snout and labials, and dark markings on supralabials 2–6. Dorsal scales with two apical pits, 23 scale rows near neck, 23 at midbody, and 17–19 anterior to the vent. Approximately 195 ventrals, 90 paired subcaudals, and divided anal scute. Nine supralabials with the fifth and sixth entering the orbit, one anterior subocular smaller than the loreal shield and situated above the fourth and anterior part of the fifth supralabials, and two preoculars and two postoculars. Also, diagnosed by a single large lower anterior temporal shield above the 7^th^ and 8^th^ supralabials, two upper anterior temporal, three posterior temporal, and maxillary with 17 + 2 teeth separated by a diastema. Its banded pattern was suggested as Batesian mimicry of the sympatric spitting cobra, *Naja nigricollis*. *Bamanophis* differs by having 25–27 scale rows near neck, 29–33 at midbody, and 17 near vent, 229–265 ventral scale and 75–95 paired subcaudals, lacking an anterior subocular, having one posterior subocular, 10 supralabials, and 15–19 maxillary teeth with diastema [105].

**Sister taxa:** *M*. *zebrinus* is basal lineage to a clade including *Bamanophis* + *Macroprotodon*, placed within a larger clade of Old World racers [15,40,107,108].

**Distribution:** Currently recognized as endemic to northern Namibia, Africa [127], but its range may extend into Angola, Africa [126].

**Remarks:** First described from a dead specimen collected in 1991 [125], the species is currently known from only three specimens [126]. Upon its description it was assigned to the genus *Coluber*, presumably on basis of similar morphology, but then switched to *Hemerophis* [24,106] with no published reasoning. Schätti and Trape [105] provide an account detailing the differences of *Bamanophis* to other racer species, including *M*. *zebrinus*.

## Conclusions

At less than half (46.33%) of the total snake diversity sampled, we provide the most comprehensive sampling effort to date, but remain far from fully estimating the snake tree of life. This sampling effort pales in comparison to larger clades such as birds that have approximately 70% of more than 10,000 species sequenced [11]. Although our results provide resolution for several higher-level nodes, these nodes may continue to prove problematic. Collectively, future analyses should target or pay special attention to the following ten issues: 1) resolving topology of Scolecophidia; 2) resolving topology of Typhlopinae; 3) resolving paraphyly of Cylindrophiidae with Anomochilidae; 4) placement of Xenophidiidae and Bolyeridae; 5) resolving topology of Booidea; 6) placement of Xenodermatidae; 7) placement of Pareatidae; 8) placement of Homalopsidae; 9) resolving topology of Lamprophiidae + Elapidae; and 10) resolving topology of Colubridae. Clearly, greater taxon and gene sampling will help better formulate a picture of snake relationships and resolve ambiguous nodes in the tree [111,117]. Taxa most lacking in representation are fossorial clades, mainly Afrotyphlopinae, Anomalepididae, Aparallactinae, Calamariinae, Cylindrophiidae, Epictinae, Gerrhopilidae, Madatyphlopinae, Uropeltidae, and Xenodermatidae at below 30% (Table 1). Similar deficiencies occur at the genus level, but are not listed here. The genes most frequently sampled for snakes are 12S, 16S, c-mos, cyt-b, and ND4, and should be considered as candidate genes in future studies. Sampling more nuclear genes will also be crucial in resolving deeper nodes [23]. Where coalescence-based methods are practiced, researchers should place emphasis on short and weakly supported branches since they are more prone to incomplete lineage sorting and thus, conflict most often with branches on species-trees [8]. This phylogeny has major implications on snake evolution such as on the evolution of gape size and the evolution of venom-delivery systems [44,46,85], and serves as a resource for formulating future studies on snake phylogenetics.

## Supporting Information

S1 Fig Abridged phylogeny displaying higher-level relationships with all rogue taxa and incertae sedis species eliminated. Maximum-likelihood phylogenetic estimate based on 10 concatenated genes. Tips represent families and sub-families. Commonly recognized higher-level clades are labeled in all caps and bold. Node values represent SHL support values. Skeleton of the species tree is displayed on the left, colored and labeled as they appear in Fig 1.
(EPS)

S1 File Data supermatrix comprising 1745 taxa representing 1652 snake species and 7 outgroup taxa, and 9523 base pairs from 10 loci. (PHY)

S2 File Newick format maximum-likelihood phylogeny for 1745 taxa representing 1652 snake species and 7 outgroup taxa displayed in Figs 2–10. (DOCX)

S1 Table List of GenBank accession numbers for 7 outgroup taxa and 1615 snake species. Two sequences were deleted during preliminary tree searches and 21 were identified as rogue taxa and pruned from the dataset leaving 1592 snake species from GenBank in the tree. Names represent species names as listed on The Reptile Database (http://www.reptile-database.org/) as of October 2015. Refer to S4 Table for list of rogue taxa. Taxa deleted during preliminary tree searches are highlighted in red, rogue taxa are highlighted in yellow, and sequences that were deleted because they were identical to other sequences are highlighted in green.
(DOCX)

S2 Table List of taxa, institutional voucher numbers, and GenBank accession numbers for tissue samples extracted and sequenced in this study. Tissue samples for *Boiga siamensis* FMNH267726, *Chrysopelea ornata* LSUHC7158, and *Psammodynastes pictus* FMNH267940 were represented by clear chromatograms, but placed poorly in preliminary phylogenetic trees, so they were not included in the final data matrix. *Tropidolaemus subannulatus* KU327425 was identified as a rogue taxon by RogueNaRok and was pruned from the dataset and thus, is not represented in the phylogeny.
(DOCX)

S3 Table Six loci, gene type, gene length, primer name, PCR annealing temperature and primer source. (DOCX)

S4 Table Rogue taxa as identified by RogueNaRok Web-Server (http://rnr.h-its.org/submit). Each taxon is associated with a raw improvement score (R.I.S.), which represents the fraction of improvement in bootstrap support values throughout the tree when the selected taxon is pruned and all rogue taxa above it are also pruned. We performed one run and chose to sacrifice relatively lower node support values to maximize the number of taxa represented in the phylogeny. Thus we elected to only prune taxa with R.I.S. greater than 0.8, resulting in a total of 22 pruned taxa (highlighted in bold).
(DOCX)
