# Supplementary material for: A Species-Level Phylogeny of Extant Snakes with Description of a New Colubrid Subfamily and Genus
Source: PLoS One. 2016 Sep 7;11(9):e0161070. doi: 10.1371/journal.pone.0161070 (PMC5014348; doi:10.1371/journal.pone.0161070)
Supplement: S2 File — (DOCX) [file pone.0161070.s003.docx]

**S1 File. Newick format maximum-likelihood phylogeny for 1745 taxa representing 1652 snake species and 7 outgroup taxa displayed in Fig 2.**

(((Heloderma_suspectum:6.229122,(Elgaria_multicarinata:4.989789,Varanus_salvator:3.889949)64:2.657709)100:2.091001,((Plica_plica:5.044157,Liolaemus_darwinii:5.298251)100:6.037607,(Chamaeleo_calyptratus:5.031263,Calotes_versicolor:6.465167)100:6.149096)52:2.790088)100:0.50047,((Liotyphlops_albirostris:1.188671,Typhlophis_squamosus:4.925207)100:1.682891,((((Rhinoleptus_koniagui:3.872932,((Trilepida_macrolepis:5.736544,(Rena_humilis:2.686746,(Rena_sp:4.941553,(Rena_dissecta:3.813885,Rena_dulcis:2.011336)88:3.053328)99:3.849972)100:2.037216)100:2.089308,((Siagonodon_septemstriatus:3.448174,(Epictia_tenella:3.426039,(Epictia_goudotii:4.127401,(Epictia_columbi:1.10055,Epictia_magnamaculata:4.117433)100:1.084492)99:2.202411)100:2.198589)97:2.276846,((Tetracheilostoma_breuili:4.549626,Tetracheilostoma_carlae:4.551041)100:5.222156,(Mitophis_sp:3.07311,(Mitophis_asbolepis:1.971187,(Mitophis_pyrites:1.971346,Mitophis_leptipileptus:1.966206)83:1.94508)97:3.062481)99:3.077127)100:3.084231)63:2.53384)77:1.14724)94:1.155244,((Myriopholis_longicauda:3.172332,(Myriopholis_algeriensis:3.481419,((Myriopholis_boueti:3.938044,Myriopholis_rouxestevae:3.086979)100:3.094065,(Myriopholis_blanfordi:3.124884,(Myriopholis_macrorhyncha:1.977333,Myriopholis_adleri:1.975205)100:3.082713)98:3.057281)94:3.052145)71:3.061607)100:3.100175,(Namibiana_occidentalis:3.168181,((Leptotyphlops_pitmani:4.912898,(Leptotyphlops_merkeri:4.914886,(Leptotyphlops_sp:3.433344,Leptotyphlops_nigroterminus:1.980476)0:3.04769)98:3.055604)98:3.057313,(Leptotyphlops_kafubi:3.752066,((Leptotyphlops_distanti:1.975224,Leptotyphlops_scutifrons:4.600781)96:1.935371,(Leptotyphlops_nigricans:4.603959,(Leptotyphlops_conjunctus:1.974177,Leptotyphlops_sylvicolus:1.976593)28:1.932949)100:1.937425)94:3.060492)21:3.058204)100:3.074393)100:3.074112)100:3.783384)100:1.360029,((Gerrhopilus_mirus:3.1646,Gerrhopilus_hedraeus:4.57475)48:3.94143,((Xenotyphlops_sp:4.707999,Xenotyphlops_grandidieri:2.13257)100:3.663174,(((Amerotyphlops_reticulatus:4.06018,(Amerotyphlops_tasymicris:5.730075,Amerotyphlops_brongersmianus:4.048969)3:5.156031)99:4.558681,((Typhlops_caymanensis:4.959652,(Typhlops_arator:3.008513,((Typhlops_perimychus:3.496412,Typhlops_anousius:3.496141)100:3.554378,(Typhlops_anchaurus:3.261305,(Typhlops_contorhinus:3.006443,(Typhlops_biminiensis:4.084394,Typhlops_notorachius:2.850166)91:2.805986)0:2.808779)97:2.804331)96:2.817647)99:3.928302)100:3.424119,((((Typhlops_geotomus:4.350709,Typhlops_monastus:3.509825)100:3.463855,(Typhlops_dominicanus:3.514866,Typhlops_guadeloupensis:4.34467)97:3.452721)100:3.756952,(Typhlops_granti:4.016143,(Typhlops_catapontus:3.186497,(Typhlops_hypomethes:3.801508,(Typhlops_richardi:3.165887,(Typhlops_platycephalus:2.818946,Typhlops_naugus:4.618275)69:2.797649)100:2.802487)85:2.807047)99:2.816884)96:2.845325)98:3.168801,((Typhlops_syntherus:3.216605,(Typhlops_sulcatus:3.2165,((Typhlops_agoralionis:2.894383,(Typhlops_hectus:3.506656,Typhlops_sylleptor:3.686366)99:2.829079)100:3.332033,(Typhlops_capitulatus:3.208705,(Typhlops_jamaicensis:0.06767,Typhlops_rostellatus:3.200369)18:0.004489)7:0.00106)60:0.010484)60:0.00454)92:0.124827,(((Typhlops_sp_19:4.335745,(Typhlops_sp_20:4.31724,Typhlops_pusillus:4.322655)97:4.394896)100:4.257859,(Typhlops_sp_16:4.348937,(Typhlops_sp_18:4.328195,Typhlops_sp_17:4.323635)92:4.272846)85:4.360337)100:4.325408,(Typhlops_schwartzi:2.929695,((Typhlops_eperopeus:2.864387,Typhlops_titanops:4.435786)100:2.859805,(((Typhlops_sp_11:4.309763,Typhlops_sp_12:4.313536)99:4.264468,(Typhlops_sp_13:4.31615,(Typhlops_sp_14:4.296393,Typhlops_sp_15:4.303093)100:4.268308)99:4.252617)100:4.286206,((Typhlops_sp_8:4.442879,Typhlops_sp_9:4.314748)99:4.292986,((Typhlops_sp_2:4.29549,Typhlops_sp_1:4.294941)100:4.299389,(Typhlops_sp_6:4.346875,(Typhlops_sp_7:4.311203,(Typhlops_sp_5:3.674365,(Typhlops_sp_4:3.671354,Typhlops_sp_3:3.671532)98:3.626212)100:4.249421)98:4.268969)91:4.254465)100:3.187566)99:3.188584)98:4.254189)96:2.80386)100:2.826571)72:1.378983)100:0.022085)99:1.127297)100:1.370997)100:1.208727,((((Xerotyphlops_vermicularis:2.885969,Xerotyphlops_socotranus:4.30269)100:2.966358,(Argyrophis_muelleri:3.007419,((Argyrhophis_sp_5:2.918106,Argyrophis_diardii:2.917985)0:2.912266,(Argyrhophis_sp_1:2.935975,(Argyrhophis_sp_3:2.90826,(Argyrhophis_sp_4:2.898496,Argyrhophis_sp_2:2.898496)0:2.828587)0:2.885933)90:2.828308)0:1.760803)100:3.607408)88:2.932061,((Rhinotyphlops_lalandei:3.291707,(Rhinotyphlops_unitaeniata:3.905229,((Letheobia_feae:2.895543,Letheobia_newtoni:3.723717)100:3.175955,(Letheobia_simonii:3.507797,Letheobia_episcopus:3.875394)70:3.437786)91:2.855936)0:2.813638)91:2.910571,(Afrotyphlops_obtusus:4.213424,((Afrotyphlops_sp_1:2.926057,(Afrotyphlops_sp_2:2.887177,(Afrotyphlops_angolensis:2.874965,Afrotyphlops_elegans:2.874539)0:2.826929)41:2.874576)100:3.275682,((Afrotyphlops_mucruso:2.903642,Afrotyphlops_schlegelii:2.903155)100:3.248612,((Afrotyphlops_bibronii:2.899534,Afrotyphlops_fornasinii:2.900242)89:3.242132,((Afrotyphlops_sp_3:2.899701,Afrotyphlops_lineolatus:2.899554)98:3.226211,(Afrotyphlops_congestus:3.319333,Afrotyphlops_punctatus:2.898954)81:2.820931)100:2.83756)93:2.80883)89:2.87488)84:2.798033)100:2.81824)93:4.387235)38:5.385783,((Madatyphlops_andasibensis:3.024514,Madatyphlops_arenarius:4.090466)100:4.968725,((Malayotyphlops_ruber:4.365647,Malayotyphlops_luzonensis:2.93059)99:4.022165,(((Indotyphlops_pammeces:4.985567,Indotyphlops_braminus:0.063706)100:0.092222,(Indotyphlops_sp:3.587887,Indotyphlops_albiceps:4.440159)0:1.070525)100:0.182006,((Ramphotyphlops_lineatus:4.904631,(Ramphotyphlops_sp:3.483786,Ramphotyphlops_acuticaudus:4.357236)92:3.140306)45:4.310168,((Acutotyphlops_kunuaensis:5.815486,(Acutotyphlops_subocularis:4.14039,Acutotyphlops_sp:5.82524)9:4.093151)100:4.393602,(Anilios_affinis:5.294916,((Anilios_bituberculatus:4.163915,(Anilios_grypus:4.167967,(Anilios_leptosomus:4.775828,Anilios_longissimus:4.152355)97:4.080345)99:4.068335)97:4.370889,((Anilios_howi:2.882517,Anilios_unguirostris:2.886186)99:3.353782,(((Anilios_proximus:4.776389,Anilios_wiedii:4.77379)61:4.692964,(Anilios_nigrescens:4.766613,(Anilios_silvia:4.765051,(Anilios_polygrammicus:4.151145,((Anilios_ganei:4.120302,Anilios_ligatus:4.110816)97:4.063161,(Anilios_troglodytes:4.118382,Anilios_kimberleyensis:4.114498)100:4.077115)100:4.069693)68:4.061491)48:4.07292)64:4.067827)83:4.376233,((Anilios_guentheri:4.201973,(Anilios_ammodytes:4.757949,Anilios_diversus:4.140036)100:4.1003)95:4.351331,((Anilios_bicolor:4.168066,(Anilios_splendidus:5.687459,Anilios_pinguis:4.122283)96:4.334112)100:4.366431,((Anilios_waitii:2.878549,Anilios_centralis:3.509558)98:3.352062,(Anilios_australis:2.83764,(Anilios_hamatus:4.15972,(Anilios_pilbarensis:4.129794,Anilios_endoterus:4.124747)95:4.086052)100:4.343954)63:2.816167)100:2.817739)99:2.810382)40:2.801937)85:2.8026)65:2.804676)66:2.819804)100:2.843688)59:3.066706)76:1.782449)81:0.006224)99:1.144575)63:1.152209)77:1.17358)100:1.159028)96:1.18533)100:1.271384)95:1.344975,((Anilius_scytale:2.035472,((Trachyboa_gularis:2.538265,Trachyboa_boulengeri:3.906002)99:1.60257,(Tropidophis_taczanowskyi:5.554007,((Tropidophis_haetianus:3.512759,Tropidophis_greenwayi:4.876581)100:3.522681,((Tropidophis_pardalis:5.456628,Tropidophis_wrighti:5.464224)97:6.406659,(Tropidophis_melanurus:2.937295,(Tropidophis_canus:4.144434,Tropidophis_feicki:4.895998)0:3.483511)86:1.12818)80:1.17595)97:1.16336)66:1.135549)100:1.263836)98:1.333939,((((Calabaria_reinhardtii:1.877267,(Candoia_aspera:6.034817,(Candoia_superciliosa:4.201055,Candoia_carinata:1.15957)87:1.137416)100:1.161165)38:1.431622,((Sanzinia_madagascariensis:3.883848,(Acrantophis_madagascariensis:1.482421,Acrantophis_dumerili:3.501529)100:1.167494)100:1.446174,(((Lichanura_trivirgata:1.822938,(Charina_bottae:1.157011,Charina_umbratica:4.139931)100:1.211986)100:1.391338,(Exiliboa_placata:3.829621,(Ungaliophis_continentalis:1.427132,(Ungaliophis_panamensis_ADM239:4.251402,Ungaliophis_panamensis_ADM270:4.251488)100:4.649904)100:0.040016)100:0.08423)100:0.040934,(((Eryx_jayakari:3.902905,Eryx_muelleri:4.253796)100:3.582464,(Eryx_colubrinus:1.807802,((Eryx_johnii:4.006693,Eryx_conicus:3.538794)88:3.525421,(Eryx_jaculus:5.594297,(Eryx_elegans:5.601534,(Eryx_miliaris:2.456494,Eryx_tataricus:3.916278)100:1.145837)20:1.123491)85:1.143857)68:1.135851)100:1.167758)100:1.16881,(Boa_constrictor:1.917823,((Corallus_batesii:3.828648,(Corallus_cropanii:4.880913,(Corallus_annulatus:3.564344,(Corallus_ruschenbergerii:4.217839,(Corallus_hortulanus:1.754042,(Corallus_cookii:2.810789,Corallus_grenadensis:2.865008)92:3.065735)100:2.902877)100:3.50796)42:3.487917)84:3.560412)67:3.926531,(((Eunectes_notaeus:3.534891,Eunectes_murinus:4.038606)100:3.523238,(Epicrates_alvarezi:4.1734,((Epicrates_cenchria:1.760516,Epicrates_maurus:1.918013)41:2.272138,(Epicrates_crassus:1.828479,Epicrates_assisi:3.581075)100:1.902527)99:3.490871)100:3.514722)89:3.490527,((Chilabothrus_angulifer:3.534421,(Chilabothrus_monensis:3.239727,Chilabothrus_inornatus:2.899371)98:3.504166)93:3.554088,(Chilabothrus_fordii:3.898421,(Chilabothrus_subflavus:4.223389,(Chilabothrus_chrysogaster:1.785439,(Chilabothrus_striatus:3.060567,(Chilabothrus_exsul:1.746888,Chilabothrus_strigilatus:2.102535)22:1.746694)97:1.828465)100:2.881442)10:3.478917)100:3.54511)100:4.106918)100:3.497598)100:3.513439)100:1.795329)37:1.332412)85:0.008835)81:0.006219)100:0.039383,(((Cylindrophis_ruffus:3.566931,(Anomochilus_leonardi:6.445247,Cylindrophis_maculatus:4.337504)91:4.3932)100:3.525062,(Melanophidium_punctatum:7.641809,((Uropeltis_sp:5.453768,Uropeltis_liura:5.457143)92:7.539091,(Brachyophidium_rhodogaster:7.64151,(Rhinophis_travancoricus:7.649162,(Rhinophis_oxyrhynchus:7.642594,(Rhinophis_dorsimaculatus:7.648657,((Rhinophis_homolepis:4.296325,(Rhinophis_blythii:4.206415,(Rhinophis_drummondhayi:2.309642,Rhinophis_erangaviraji:2.583636)96:0.013215)68:0.015071)49:0.044563,(Pseudotyphlops_philippinus:4.201016,(Rhinophis_philippinus:6.561058,(Uropeltis_melanogaster:4.806068,Uropeltis_phillipsi:4.590557)100:2.890672)75:3.129363)85:0.84436)97:1.154704)87:1.141531)74:1.132627)100:1.131445)95:1.125039)98:1.122371)98:1.221952)100:1.411411,(Xenopeltis_unicolor:2.170106,(Loxocemus_bicolor:1.542109,((Python_regius:6.098085,((Python_curtus:4.593571,Python_brongersmai:4.966197)100:5.698672,((Python_anchietae:4.224473,Python_sebae:3.550148)68:4.563642,(Python_bivittatus:5.626566,Python_molurus:4.606576)100:4.837732)99:3.497526)90:3.482008)99:3.735485,(((Malayopython_timoriensis:5.465652,Malayopython_reticulatus:0.033311)100:0.025962,((Aspidites_ramsayi:3.531331,Aspidites_melanocephalus:3.517767)100:3.504644,(Bothrochilus_boa:6.021852,(Bothrochilus_albertisii:3.514217,Bothrochilus_meridionalis:6.576278)100:3.498681)99:3.52235)96:3.721002)91:0.008005,((Morelia_viridis:4.914161,((Morelia_carinata:5.746951,(Morelia_bredli:5.708782,Morelia_spilota:3.554714)100:3.514379)91:3.791024,(Antaresia_maculosa:5.762773,(Antaresia_perthensis:5.758774,(Antaresia_childreni:5.382852,Antaresia_stimsoni:5.739278)100:5.369238)26:5.36147)83:5.672391)38:3.702905)99:2.368086,((Simalia_boeleni:3.521294,(Simalia_tracyae:4.081016,(Simalia_nauta:4.074651,(Simalia_kinghorni:5.990278,Simalia_amethistina:5.414849)93:5.941768)100:3.485269)100:3.491395)80:3.505912,(Simalia_oenpelliensis:5.786731,(Liasis_olivaceus:5.771333,(Liasis_papuana:2.222178,(Liasis_mackloti:2.197843,Liasis_fuscus:5.760117)100:2.19729)27:2.183224)100:2.194265)44:2.437451)53:1.371678)85:8.66E-4)100:0.709856)100:0.028219)100:0.059732)63:0.017922)99:0.022713)99:0.022758,((Casarea_dussumieri:1.21268,Xenophidion_schaeferi:5.895627)91:1.388391,((Acrochordus_javanicus:3.953766,(Acrochordus_arafurae:5.432924,Acrochordus_granulatus:3.441679)76:1.788857)100:2.149776,((Stoliczkia_borneensis:3.593578,(Xenodermus_javanicus:4.182834,(Achalinus_meiguensis:5.465946,Achalinus_rufescens:6.564976)97:6.020714)83:3.515932)100:1.511125,(((((Asthenodipsas_lasgalenensis:6.23124,Asthenodipsas_vertebralis:6.240512)100:7.817386,(Asthenodipsas_malaccanus_FMNH273617:6.197371,(Asthenodipsas_laevis:7.948831,Asthenodipsas_laevis_FMNH241296:6.196697)43:6.175784)100:6.256638)95:6.60082,(Aplopeltura_boa:4.876012,((Pareas_carinatus:2.889323,Pareas_nuchalis:7.655809)95:3.062081,(Pareas_monticola:4.843504,(Pareas_margaritophorus:5.262047,((Pareas_stanleyi:4.767485,(Pareas_boulengeri:4.747014,Pareas_formosensis:4.742032)100:4.738273)95:4.938634,(Pareas_hamptoni:2.910354,(Pareas_komaii:4.73968,(Pareas_atayal:3.620977,Pareas_iwasakii:3.617994)75:4.728507)99:5.541042)100:2.869476)56:3.484588)87:3.541861)97:4.130104)64:1.747866)94:1.770225)100:2.002318,(((Proatheris_superciliaris:6.57836,(Causus_resimus:6.265023,(Causus_defilippii:4.68884,Causus_rhombeatus:6.329652)78:4.748857)100:4.63535)26:4.797278,(((Atheris_chlorechis:6.499522,((Atheris_barbouri:5.959971,(Atheris_matildae:5.258795,Atheris_ceratophora:4.525836)97:5.673773)98:5.620993,((Atheris_hispida:4.89985,Atheris_squamigera:4.456503)100:5.558443,(Atheris_nitschei:4.463859,Atheris_desaixi:4.917029)39:5.54898)81:5.541516)75:6.176224)100:5.361931,(Bitis_worthingtoni:6.751436,(Bitis_arietans:5.462137,((Bitis_nasicornis:4.487814,(Bitis_gabonica:4.635928,(Bitis_parviocula:5.914858,Bitis_rhinoceros:4.66335)0:4.42151)92:4.590731)100:5.582194,((Bitis_schneideri:5.846897,(Bitis_peringueyi:4.616452,Bitis_caudalis:4.52407)0:4.457429)100:4.466298,(Bitis_xeropaga:5.995975,(Bitis_atropos:4.533964,(Bitis_cornuta:3.751583,(Bitis_armata:4.878357,Bitis_rubida:4.068309)0:3.613486)99:4.437642)100:4.46046)100:4.449612)99:5.72185)100:5.309672)80:5.112072)100:5.126324)98:5.13413,(((Cerastes_vipera:7.389248,(Cerastes_cerastes:4.526852,Cerastes_gasperettii:6.626916)99:5.130476)100:4.368459,(Echis_carinatus:5.273475,((Echis_jogeri:4.749345,Echis_ocellatus:3.472798)100:4.540498,((Echis_omanensis:4.735096,Echis_coloratus:3.474282)100:4.521755,(Echis_khosatzkii:4.731055,(Echis_borkini:4.738814,(Echis_leucogaster:4.722831,Echis_pyramidum:3.455597)100:3.375831)92:3.369208)100:3.418537)82:4.488694)67:5.124612)100:5.347471)57:4.440959,((Eristicophis_macmahoni:6.301798,(Pseudocerastes_fieldi:6.02288,(Pseudocerastes_persicus:4.528077,Pseudocerastes_urarachnoides:4.889078)93:5.666437)92:6.718005)100:5.804878,(((Macrovipera_schweizeri:5.096269,Macrovipera_lebetina:4.523047)100:5.607551,(Montivipera_xanthina:4.52273,((Montivipera_raddei:4.511924,Montivipera_latifii:4.515361)100:4.907339,(Montivipera_bornmuelleri:4.990725,(Montivipera_wagneri:4.515752,Montivipera_albizona:4.514764)51:4.428541)97:4.424255)49:4.427355)98:5.60052)100:4.872211,((Daboia_russelii:1.821032,(Daboia_palaestinae:4.672878,(Daboia_deserti:5.201898,Daboia_mauritanica:4.521788)100:4.576411)97:4.722064)97:2.883732,(Vipera_ammodytes:4.774975,((Vipera_latastei:4.646894,Vipera_aspis:4.54072)99:5.054395,((Vipera_berus:4.528795,Vipera_seoanei:4.649086)88:4.623337,((Vipera_dinniki:4.670575,(Vipera_kaznakovi:4.514036,Vipera_orlovi:4.52436)100:4.970286)11:3.552088,(Vipera_ursinii:4.440792,(Vipera_eriwanensis:4.641802,(Vipera_renardi:4.513269,Vipera_lotievi:4.512494)98:4.557844)100:4.623117)87:4.832397)100:3.390653)100:3.368262)68:4.434004)100:5.743479)99:2.862755)87:3.487081)100:3.49694)14:3.482078)91:3.490132)100:3.697556,(Azemiops_feae:3.812329,((Calloselasma_rhodostoma:6.823914,(Hypnale_nepa:5.164405,(Hypnale_zara:5.158977,Hypnale_hypnale:6.771061)98:5.120661)100:5.133)100:5.369982,((Garthius_chaseni:6.634194,(Deinagkistrodon_acutus:6.599552,(Tropidolaemus_subannulatus_KU307696:5.569337,Tropidolaemus_wagleri:5.776955)100:5.437202)60:5.379923)18:5.780928,((((Trimeresurus_puniceus:6.29221,(Trimeresurus_wiroti:7.337695,Trimeresurus_borneensis:6.25779)90:6.198964)94:6.459166,(Trimeresurus_malabaricus:6.569472,(Trimeresurus_trigonocephalus:6.209175,Trimeresurus_gramineus:6.550803)55:6.179683)100:6.193521)98:6.429799,(Trimeresurus_tibetanus:6.811107,((Trimeresurus_hageni:6.318386,((Trimeresurus_schultzei:5.61472,Trimeresurus_sumatranus:7.173558)93:5.629763,(Trimeresurus_malcolmi:7.187943,(Trimeresurus_flavomaculatus:5.604707,Trimeresurus_mcgregori:7.154574)100:5.577421)84:5.58534)100:6.182265)100:6.672581,(((Trimeresurus_fucatus_88_LSUHC7566:3.778576,((Trimeresurus_nebularis_89_LSUHC10268:3.78333,Trimeresurus_popeiorum:6.121021)71:3.616969,(Trimeresurus_fucatus_87_LSUHC6688:5.732414,(Trimeresurus_buniana_86_LSUHC5608:4.146693,Trimeresurus_buniana_85_LSUHC6118:3.334039)84:3.620708)100:3.760994)98:3.618092)100:3.634757,(Trimeresurus_rubeus:7.809495,(Trimeresurus_kanburiensis:8.194611,((Trimeresurus_macrops:5.339552,(Trimeresurus_honsonensis_73_LSUHC8655:4.1761,Trimeresurus_honsonensis_74_LSUHC8602:2.843908)98:3.861248)99:4.369079,(Trimeresurus_venustus:5.306528,(Trimeresurus_cardamonensis_72_LSUHC10089:3.002104,Trimeresurus_cardamomensis:4.521341)96:5.261406)63:2.803045)91:3.622372)77:3.616264)100:3.633114)87:4.287164,(Trimeresurus_sichuanensis:5.522043,((Trimeresurus_vogeli:4.843447,(Trimeresurus_truongsonensis:5.648392,(Trimeresurus_stejnegeri:4.783982,Trimeresurus_gumprechti:4.811281)100:4.744799)84:4.733553)99:4.773867,(Trimeresurus_yunnanensis:4.84424,(Trimeresurus_medoensis:4.827762,((Trimeresurus_insularis:5.603351,Trimeresurus_fasciatus:5.605324)100:4.779861,(Trimeresurus_septentrionalis:5.633763,(Trimeresurus_albolabris:4.488904,(Trimeresurus_andersonii:6.047697,(Trimeresurus_cantori:4.476628,(Trimeresurus_erythrurus:3.658023,Trimeresurus_purpureomaculatus:3.654337)100:3.619565)50:2.802549)96:2.83399)92:3.923277)95:4.751648)100:3.952924)88:3.929715)24:4.770997)90:5.36795)93:5.833687)92:4.241337)95:4.245386)100:3.187161)100:4.454878,(((Ovophis_tonkinensis:6.294971,(Ovophis_monticola:6.261143,Ovophis_zayuensis:6.247921)94:6.176867)100:6.526865,((Protobothrops_kaulbacki:6.633026,Protobothrops_sieversorum:6.273017)98:6.56332,(Protobothrops_mangshanensis:6.23021,((Protobothrops_cornutus:6.25922,(Protobothrops_dabieshanensis:5.622746,(Protobothrops_xiangchengensis:4.482441,Protobothrops_jerdonii:4.484017)100:5.56284)83:6.168542)98:6.195896,((Protobothrops_tokarensis:5.611553,Protobothrops_flavoviridis:5.611508)100:5.616068,(Protobothrops_elegans:5.675671,(Protobothrops_mucrosquamatus:5.602474,Protobothrops_maolanensis:4.47931)67:5.57007)100:5.576292)63:6.203227)100:5.400759)99:6.173517)99:5.121784)100:5.52257,(((Trimeresurus_gracilis:6.296438,Ovophis_okinavensis:6.246249)100:6.102088,(((Gloydius_brevicaudus:6.204061,Gloydius_blomhoffii:6.589875)99:5.42107,(Gloydius_ussuriensis:6.191532,Gloydius_tsushimaensis:6.588792)99:5.422269)100:5.73364,(Gloydius_strauchi:6.6507,(Gloydius_liupanensis:7.399202,(Gloydius_halys:2.914553,(Gloydius_intermedius:6.532651,(Gloydius_saxatilis:6.190958,Gloydius_shedaoensis:6.654358)100:5.365241)100:5.763826)100:2.566341)79:2.55124)97:2.554592)100:2.588965)77:2.554745,(((((Atropoides_occiduus:6.24309,Atropoides_indomitus:7.317164)99:6.258752,(Atropoides_nummifer:6.263062,(Atropoides_mexicanus:6.234572,Atropoides_olmec:6.238023)50:6.17263)100:6.227186)100:6.208425,(Atropoides_picadoi:6.304847,((Cerrophidion_godmani:5.637953,(Cerrophidion_tzotzilorum:4.493797,Cerrophidion_petlalcalensis:4.491363)100:5.577157)97:6.176042,(Cerrophidion_wilsoni:6.601536,((Porthidium_ophryomegas:4.508021,(Porthidium_hespere:5.134455,Porthidium_dunni:4.498098)94:3.656985)94:3.643657,(Porthidium_yucatanicum:4.527169,(Porthidium_nasutum:4.499427,(Porthidium_porrasi:4.491325,(Porthidium_lansbergii:3.660934,Porthidium_arcosae:5.189021)100:3.623131)98:3.616719)64:3.630952)100:3.629525)95:3.680056)78:5.368364)79:4.330782)61:4.356013)100:5.766738,((Bothrops_lojanus:6.172318,(Bothrocophias_hyoprora:6.235451,Bothrocophias_microphthalmus:6.231997)96:5.409592)25:5.05802,(Bothrocophias_campbelli:6.527131,(Bothrops_pictus:6.530185,((Bothrops_ammodytoides:4.589672,(Bothrops_alternatus:4.585682,(Bothrops_fonsecai:5.182686,(Bothrops_cotiara:4.475639,Bothrops_itapetiningae:4.484913)79:4.466162)97:4.505529)94:4.43179)97:6.020978,(((Bothrops_insularis:4.514519,(Bothrops_jararaca:4.484923,Bothrops_alcatraz:5.632825)91:4.427645)100:4.513653,((Bothrops_lutzi:5.216225,Bothrops_erythromelas:4.505244)96:4.516029,((Bothrops_neuwiedi:4.495624,Bothrops_marmoratus:5.102749)96:4.640963,(Bothrops_matogrossensis:5.222789,(Bothrops_pubescens:4.500356,(Bothrops_diporus:4.485856,Bothrops_pauloensis:4.49018)61:4.434578)87:4.434307)99:4.50106)95:3.371852)100:3.396325)99:3.767975,((Bothrops_pulchra:5.520321,(Bothrops_bilineata:4.568923,(Bothrops_taeniata:4.494835,(Bothrops_chloromelas:4.469304,Bothrops_oligolepis_LSUMNS9374:4.029585)99:3.658115)78:3.692343)97:3.623864)99:4.053663,((Bothrops_brazili:4.499712,Bothrops_jararacussu:4.499277)100:4.127116,((Bothrops_punctatus:4.490947,Bothrops_osbornei:4.490504)99:4.693245,((Bothrops_caribbaeus:4.487975,Bothrops_lanceolatus:4.488167)100:4.608081,(Bothrops_asper:2.115863,((Bothrops_marajoensis:4.642342,Bothrops_atrox:2.578517)89:2.555294,(Bothrops_isabelae:4.202387,(Bothrops_moojeni:4.49185,Bothrops_leucurus:4.487316)94:3.622943)80:4.062442)100:2.556455)94:1.744282)100:1.745337)95:1.739413)100:1.74294)93:1.736654)99:2.856972)93:3.481723)99:3.481952)95:3.482474)100:3.512606)93:3.529903,((((Lachesis_acrochorda:5.429157,Lachesis_muta:5.421876)92:5.432618,(Lachesis_melanocephala:6.122269,Lachesis_stenophrys:4.610947)81:4.625201)100:4.597032,((Ophryacus_undulatus:5.451729,(Mixcoatlus_melanurus:5.45014,(Mixcoatlus_barbouri:4.300475,Mixcoatlus_browni:5.424301)23:5.382097)100:5.372591)100:5.428519,((Bothriechis_schlegelii:2.278654,Bothriechis_supraciliaris:5.450512)100:2.230777,((Bothriechis_nigroviridis:5.470587,(Bothriechis_guifarroi:4.30977,Bothriechis_lateralis:4.309013)100:5.380525)15:5.365372,(Bothriechis_bicolor:5.436304,((Bothriechis_aurifer:4.308456,Bothriechis_rowleyi:4.310655)80:4.247769,(Bothriechis_marchi:4.307846,Bothriechis_thalassinus:4.306333)100:4.260998)72:5.359881)100:5.377434)100:4.318912)100:2.214036)74:2.218788)92:2.671154,((Agkistrodon_contortrix:3.899094,(Agkistrodon_piscivorus:4.569205,(Agkistrodon_taylori:5.430727,(Agkistrodon_bilineatus:3.68149,(Agkistrodon_howardgloydi:3.707835,Agkistrodon_russeolus:3.707311)100:5.081311)49:5.365785)99:5.701472)100:4.745457)100:3.52656,((Sistrurus_miliarius:6.587832,Sistrurus_catenatus:5.418423)99:5.572104,(Crotalus_ravus:6.008653,(((Crotalus_lepidus:3.403334,Crotalus_aquilus:4.997867)99:2.560382,(Crotalus_triseriatus:3.654724,(Crotalus_pusillus:5.414649,Crotalus_sp:6.353883)94:4.844767)25:3.812597)100:4.359809,((Crotalus_enyo:4.639639,(Crotalus_cerastes:2.60228,Crotalus_polystictus:2.663034)39:2.559848)62:4.307701,((Crotalus_pricei:3.662529,(Crotalus_intermedius:5.774346,(Crotalus_tancitarensis:5.736157,Crotalus_transversus:5.398166)100:5.909061)90:5.624567)100:3.888175,(((Crotalus_willardi:2.995011,Crotalus_horridus:3.844064)74:2.564319,((Crotalus_basiliscus:4.002098,(Crotalus_molossus:3.63497,Crotalus_totonacus:4.702004)92:3.617795)91:2.804451,(Crotalus_culminatus:5.364522,(Crotalus_tzabcan:6.165368,(Crotalus_durissus:4.579502,Crotalus_simus:5.034109)100:4.533197)98:3.717791)100:4.008171)100:3.820419)95:2.551655,((Crotalus_stejnegeri:3.857175,(Crotalus_lannomi:3.036338,Crotalus_ericsmithi:1.784479)94:2.563821)100:3.356398,((Crotalus_catalinensis:4.693134,(Crotalus_ruber:3.084434,Crotalus_atrox:1.772117)85:2.560401)100:1.761343,((Crotalus_mitchellii:2.586221,(Crotalus_adamanteus:1.84332,Crotalus_tigris:1.774097)15:2.555363)84:1.743628,(Crotalus_scutulatus:4.179091,(Crotalus_viridis:3.731763,(Crotalus_oreganus:3.620766,Crotalus_cerberus:3.761401)95:5.196494)83:2.549444)100:1.754526)92:1.740333)95:2.554528)36:2.551756)63:2.552492)90:4.297516)99:4.299691)64:4.299146)54:4.307843)100:4.337511)97:3.492734)94:1.118844)100:1.312254)89:0.005621)97:0.003446)100:0.017993)59:0.013227)99:0.011264)100:0.01936)100:0.05824)53:0.011768,((((Brachyorrhos_raffrayi:7.157245,Brachyorrhos_wallacei:7.157962)100:7.49563,(Dieurostus_dussumieri:6.67732,((Pseudoferania_polylepis:6.226163,Myron_richardsonii:6.237135)100:5.634113,((Phytolopsis_punctata:6.453558,(Subsessor_bocourti:5.826592,(Homalopsis_buccata:2.466756,(Cerberus_australis:5.81438,(Cerberus_microlepis:5.791246,Cerberus_rynchops:4.448446)100:4.436913)100:4.434856)81:1.069333)99:1.698925)70:1.728477,(((Bitia_hydroides:5.610053,Erpeton_tentaculatum:5.610775)29:5.548226,(Cantoria_violacea:5.605965,(Gerarda_prevostiana:5.600043,Fordonia_leucobalia:5.601646)80:5.552987)99:5.549297)96:5.576607,((Hypsiscopus_matannensis:5.601129,(Hypsiscopus_sp:5.953886,Hypsiscopus_plumbea:5.578861)92:5.553871)100:5.59912,(Myrrophis_chinensis:5.618792,(Enhydris_enhydris:5.601996,((Enhydris_innominata:6.503578,Enhydris_longicauda:5.932356)100:5.563357,(Enhydris_jagorii:5.938666,Enhydris_subtaeniata:5.584753)100:5.56739)87:5.548973)100:5.559174)94:5.581268)100:5.550052)96:6.38386)94:1.706623)92:1.71009)94:1.706554)100:1.990823,(((Rhamphiophis_oxyrhynchus:4.240461,(Psammodynastes_pictus:4.483823,(Psammodynastes_pictus_90_LSUHC4093:6.253828,Psammodynastes_pulverulentus:6.212033)80:6.754514)100:4.469533)100:3.951875,(((Rhamphiophis_rostratus:3.699597,Rhamphiophis_rubropunctatus:4.089983)100:3.683533,(Malpolon_insignitus:6.183509,(Rhagerhis_moilensis:6.259501,Malpolon_monspessulanus:4.488779)94:3.653539)92:3.634835)97:3.889916,(((Mimophis_mahfalensis:2.015149,(Hemirhagerrhis_hildebrandtii:3.861203,(Hemirhagerrhis_kelleri:4.505209,Hemirhagerrhis_viperina:4.857395)67:5.69941)98:4.036037)59:1.942253,(Psammophylax_acutus:5.76467,(Psammophylax_rhombeatus:3.821172,(Psammophylax_variabilis:1.088955,Psammophylax_tritaeniatus:4.630395)95:2.185394)100:2.20828)99:2.421199)100:1.174499,(Dipsina_multimaculata:6.642719,((Psammophis_crucifer:6.635657,(Psammophis_condanarus:6.233705,Psammophis_lineolatus:4.549365)100:4.460673)31:4.449094,((Psammophis_trigrammus:6.250848,((Psammophis_notostictus:5.08721,Psammophis_jallae:5.102658)12:5.065709,(Psammophis_leightoni:5.063381,Psammophis_namibensis:5.07131)100:5.087447)91:6.20201)100:6.744456,(Psammophis_angolensis:6.807143,(((Psammophis_elegans:4.825143,Psammophis_punctulatus:4.490566)75:4.999168,(Psammophis_praeornatus:3.406173,(Psammophis_aegyptius:5.135647,Psammophis_schokari:2.727235)98:2.711575)77:2.693926)98:3.327629,((Psammophis_biseriatus:4.795091,(Psammophis_tanganicus:4.781222,Psammophis_sp_3:2.729575)63:2.695668)100:2.717565,(Psammophis_lineatus:2.753583,((Psammophis_leopardinus:4.691644,(Psammophis_brevirostris:4.690311,(Psammophis_mossambicus:1.878183,Psammophis_phillipsii:3.614388)100:1.896155)51:1.068865)100:1.903144,((Psammophis_rukwae:3.870027,Psammophis_sibilans:1.889217)100:1.897338,(Psammophis_sp_1:4.703829,(Psammophis_subtaeniatus:4.700522,(Psammophis_orientalis:1.902466,Psammophis_sudanensis:3.870766)7:1.883788)98:1.070058)8:1.065018)97:1.890334)89:2.696106)98:2.698031)98:3.322468)3:4.437625)93:3.380369)100:4.484384)100:3.400748)46:2.383597)97:1.140617)100:1.155029)100:1.348894,((Buhoma_procterae:4.583144,(Prosymna_ruspolii:6.919901,(Prosymna_visseri:4.947088,(Prosymna_janii:4.491341,(Prosymna_meleagris:7.171355,Prosymna_greigerti:7.178372)100:8.003799)10:4.463616)90:4.505957)100:4.473592)56:4.920974,(((Pythonodipsas_carinata:4.969049,Pseudaspis_cana:4.497571)100:4.13834,((Atractaspis_irregularis:5.915111,((Homoroselaps_lacteus:2.933292,(Atractaspis_microlepidota:6.018069,(Atractaspis_boulengeri:3.697297,(Atractaspis_bibronii:3.70292,(Atractaspis_corpulenta:2.271558,Atractaspis_micropholis:1.762578)99:2.198744)84:2.191124)98:2.217644)97:2.230346)98:2.206636,((Macrelaps_microlepidotus:3.705226,(Amblyodipsas_polylepis:3.704262,(Amblyodipsas_dimidiata:5.928424,Xenocalamus_transvaalensis:3.690337)89:3.629123)100:3.65245)100:3.634702,((Polemon_notatus:3.714741,(Polemon_acanthias:3.695047,Polemon_collaris:3.683377)63:3.624631)100:3.650431,(Aparallactus_modestus:2.91663,(Aparallactus_werneri:5.148391,(Aparallactus_guentheri:5.133558,Aparallactus_capensis:2.559524)100:2.848759)96:2.814408)100:2.838389)14:2.817522)99:2.845004)74:2.197571)95:2.612986,(Oxyrhabdium_leporinum:5.557424,(((Hormonotus_modestus:5.364094,Inyoka_swazicus:7.628325)96:4.60456,((Gonionotophis_capensis:2.247335,Gonionotophis_chanleri:5.320974)100:2.265029,((Gonionotophis_nyassae:5.345437,Gonionotophis_stenophthalmus:5.344068)87:5.283208,(Gonionotophis_brussauxi:5.361118,Gonionotophis_poensis:5.328141)72:5.277582)100:5.257307)100:2.215707)98:1.517325,((Lycophidion_nigromaculatum:7.90566,(Lycophidion_laterale:5.76201,(Lycophidion_ornatum:7.864602,Lycophidion_capense:1.84179)86:1.986053)18:1.757209)100:1.776012,(Pseudoboodon_lemniscatus:8.096108,((Bothrolycus_ater:5.330866,(Bothrophthalmus_lineatus:5.283582,Bothrophthalmus_brunneus:5.291073)100:5.306781)97:5.698636,(((Boaedon_virgatus:5.524696,Boaedon_upembae:6.474681)98:3.648561,(Boaedon_capensis:4.944359,(Boaedon_olivaceus:3.611216,(Boaedon_lineatus:5.276416,(Boaedon_radfordi:5.537319,Boaedon_fuliginosus:1.370943)93:0.005615)8:0.004941)95:0.01165)100:0.023764)100:0.049918,((Lamprophis_guttatus:5.331133,(Lamprophis_fiskii:5.315915,(Lamprophis_aurora:7.613364,Lamprophis_fuscus:7.947745)41:6.110719)100:5.254547)21:5.261951,(Lycodonomorphus_inornatus:5.326741,(Lycodonomorphus_rufulus:5.308735,(Lycodonomorphus_laevissimus:7.632605,Lycodonomorphus_whytii:5.30415)99:5.250537)100:5.260526)97:5.259592)97:4.633042)96:0.004778)97:0.004611)80:0.006463)60:0.005373)100:0.013105)92:0.032457)88:0.010178)94:0.01114,(((Ditypophis_sp:4.980356,Micrelaps_bicoloratus:7.040345)0:2.864209,(((Dromicodryas_quadrilineatus:6.217713,Dromicodryas_bernieri:4.483416)100:4.683668,(Thamnosophis_infrasignatus:7.628424,((Thamnosophis_lateralis:4.507075,Thamnosophis_stumpffi:8.036772)99:3.66946,(Thamnosophis_epistibes:6.202197,(Thamnosophis_martae:4.267777,Thamnosophis_mavotenda:4.590646)99:6.172603)96:6.392135)95:3.670906)100:3.889007)98:2.01483,(((Parastenophis_betsileanus:4.535864,(Leioheterodon_geayi:6.258381,(Leioheterodon_madagascariensis:1.159374,Leioheterodon_modestus:5.126865)99:2.199233)94:2.499959)96:2.460762,((Langaha_madagascariensis:5.627667,(Micropisthodon_ochraceus:5.410213,(Ithycyphus_miniatus:5.40456,Ithycyphus_oursi:3.357937)99:3.33715)93:4.457779)100:4.70777,(((Madagascarophis_meridionalis:1.678781,Madagascarophis_colubrinus:6.336018)98:0.836619,(Madagascarophis_sp:5.898381,Madagascarophis_fuchsi:6.307573)100:5.525785)100:1.963308,((Phisalixella_variabilis:5.903547,Phisalixella_tulearensis:5.893115)100:6.344819,(Lycodryas_inornatus:6.386349,((Lycodryas_inopinae:5.346892,(Lycodryas_granuliceps:5.319414,(Lycodryas_sp:2.867031,Lycodryas_pseudogranuliceps:5.324593)34:2.547237)100:2.557728)81:2.773085,((Lycodryas_cococola:5.083881,Lycodryas_maculatus:5.084019)100:5.532247,(Lycodryas_citrinus:0.850436,Lycodryas_gaimardi:5.561158)100:0.832769)6:0.81409)100:1.648821)99:1.63514)96:2.751439)91:1.932817)96:1.961728)93:1.128326,(((Amplorhinus_multimaculatus:5.337701,(Duberria_variegata:4.178609,Duberria_lutrix:4.177375)100:5.296301)98:5.266745,(Alluaudina_bellyi:5.678886,(Ditypophis_vivax:4.56959,((Compsophis_infralineatus:3.11179,Compsophis_laphystius:5.268858)86:2.7079,(Compsophis_albiventris:4.844792,Compsophis_boulengeri:5.232008)89:4.443555)99:4.141242)94:5.246802)85:5.26447)22:4.189594,((Elapotinus_picteti:7.483842,((Liophidium_rhodogaster:5.090847,Liophidium_pattoni:5.090253)97:5.89285,((Liophidium_mayottensis:4.47245,(Liophidium_chabaudi:2.741491,Liophidium_torquatum:4.462884)96:2.706072)94:2.695492,(Liophidium_vaillanti:4.462791,(Liophidium_therezieni:4.456096,Liophidium_maintikibo:4.455845)93:4.427755)100:4.45353)95:3.07524)86:4.192112)90:4.20053,((Pseudoxyrhopus_ambreensis:4.512908,(Heteroliodon_occipitalis:3.365228,Heteroliodon_sp:5.610263)85:4.133734)98:5.285804,(((Liopholidophis_dimorphus:4.459265,Liopholidophis_rhadinaea:4.465558)85:4.447447,(Liopholidophis_oligolepis:4.447918,Liopholidophis_baderi:4.868034)94:4.456128)100:6.488609,((Liopholidophis_grandidieri:4.485476,Liopholidophis_dolicocercus:4.491377)96:4.633887,(Liopholidophis_sexlineatus:2.728885,Liopholidophis_varius:4.851986)99:2.715928)100:4.142347)100:4.192143)87:5.246984)100:5.252451)100:5.486284)84:1.127697)100:1.133582)98:1.335469,(Buhoma_depressiceps:5.009093,((Calliophis_melanurus:5.997753,(Calliophis_castoe:7.101442,Calliophis_nigrescens:7.102873)97:8.56751)99:5.411752,(((Sinomicrurus_japonicus:5.609998,(Sinomicrurus_kelloggi:6.553688,(Calliophis_sp:7.405272,Sinomicrurus_macclellandi:6.380234)92:6.363752)100:6.410366)100:6.308336,(Micruroides_euryxanthus:5.918155,(((Micrurus_alleni_ADM272:4.81243,Micrurus_alleni_ADM258:5.210415)100:5.162892,((Micrurus_psyches:7.411497,(Micrurus_corallinus:6.255731,Micrurus_albicinctus:6.255226)90:7.627434)98:9.490622,(Micrurus_diastema:8.489001,(Micrurus_mosquitensis:7.581144,(Micrurus_fulvius:1.774731,Micrurus_tener:7.591416)99:1.744276)85:2.858446)90:2.858766)38:2.856883)100:2.882023,(Micrurus_narduccii:5.825045,(Micrurus_decoratus:7.638357,((Micrurus_hemprichii:7.818765,(Micrurus_lemniscatus:6.699426,Micrurus_surinamensis:2.228851)75:2.184063)95:2.199049,((Micrurus_dissoleucus:7.70022,Micrurus_mipartitus:6.232229)99:7.766214,(Micrurus_altirostris:5.514706,((Micrurus_spixii:5.433995,(Micrurus_brasiliensis:4.617375,Micrurus_frontalis:4.61688)72:5.345412)42:5.386303,(Micrurus_ibiboboca:5.43613,(Micrurus_pyrrhocryptus:4.597879,Micrurus_baliocoryphus:4.595725)100:5.369219)84:5.367088)99:4.71006)87:7.914569)81:5.989208)80:2.189965)38:2.188919)99:2.419576)100:1.146045)84:1.124352)100:1.345474,(((Ophiophagus_hannah:4.061583,((Dendroaspis_jamesoni_YPM013380:6.222419,Dendroaspis_jamesoni_YPM012647:4.786747)100:5.032801,(Dendroaspis_angusticeps:2.730228,Dendroaspis_polylepis:5.985948)88:2.239604)100:2.245675)62:2.573226,(Hemibungarus_calligaster:7.281032,(Aspidelaps_lubricus:7.292267,(Hemachatus_haemachatus:7.288295,((Aspidelaps_scutatus:7.336896,Walterinnesia_aegyptia:7.310426)87:7.168884,(((Naja_sputatrix:7.858035,(Naja_sumatrana:7.773196,Naja_siamensis:7.31073)0:7.276504)58:8.070183,(Naja_kaouthia:1.759345,(Naja_atra:6.349544,Naja_naja:6.245622)100:4.183479)88:1.747164)100:1.76165,(((Naja_pallida:7.315036,Naja_nubiae:7.321339)95:7.422803,(Naja_katiensis:7.411927,(Naja_nigricollis:7.327728,(Naja_ashei:7.321819,(Naja_mossambica:7.324115,Naja_nigricincta:7.813564)82:7.260787)97:7.263419)100:7.339826)99:7.359331)100:6.723148,((Naja_annulata:7.286264,(Naja_melanoleuca:5.304692,Naja_multifasciata:7.591491)0:5.235611)100:5.252209,(Naja_nivea:6.388829,(Naja_senegalensis:9.690589,((Naja_annulifera:6.518071,Naja_anchietae:6.518454)99:6.463751,(Naja_haje:6.515678,Naja_arabica:6.515436)90:6.460992)43:7.260134)99:7.71443)100:6.59144)80:4.178318)100:4.412034)100:1.755269)78:1.743329)93:1.737433)90:1.741042)64:1.742957)100:0.007905,((Bungarus_flaviceps:7.475288,((Bungarus_bungaroides:7.405889,Bungarus_slowinskii:7.390911)100:7.250843,(Bungarus_fasciatus:1.331081,((Bungarus_niger:6.99226,(Bungarus_candidus:6.374162,Bungarus_multicinctus:6.369622)100:6.380952)93:6.560248,(Bungarus_sindanus:7.166702,(Bungarus_caeruleus:7.169296,Bungarus_ceylonicus:5.325291)99:5.296235)94:5.32782)100:5.5265)100:0.040977)91:0.014548)99:0.01905,((Elapsoidea_nigra:6.80582,(Elapsoidea_semiannulata:2.231839,Elapsoidea_sundevallii:6.98026)99:2.234144)100:2.672608,((Laticauda_laticaudata:4.512101,((Laticauda_guineai:7.122243,Laticauda_colubrina:1.096777)100:1.097251,(Laticauda_frontalis:5.648265,(Laticauda_saintgironsi:7.151348,Laticauda_semifasciata:6.804829)0:6.163281)77:2.112367)100:1.089164)100:1.329763,(((Toxicocalamus_loriae:5.333289,Micropechis_ikaheka:5.317632)74:5.261729,(Toxicocalamus_preussi:5.352431,(Demansia_psammophis:5.299185,(Demansia_papuensis:5.291406,Demansia_vestigiata:5.260118)52:5.251857)100:5.296498)100:5.254043)38:5.454043,((((Pseudechis_porphyriacus:5.323291,((Pseudechis_australis:5.294809,Pseudechis_butleri:6.650917)100:5.254641,(Pseudechis_papuanus:6.631436,(Pseudechis_guttatus:6.625152,Pseudechis_colletti:6.62149)78:6.362374)100:6.396664)100:5.296706)100:5.252056,(Acanthophis_laevis:6.750296,((Acanthophis_pyrrhus:6.435807,Acanthophis_wellsi:6.433386)92:6.849438,(Acanthophis_antarcticus:7.074433,(Acanthophis_rugosus:7.073649,Acanthophis_praelongus:5.283997)89:5.247146)92:5.240793)51:5.242293)100:3.646163)94:3.629305,((Aspidomorphus_schlegeli:6.455383,(Aspidomorphus_lineaticollis:6.452915,Aspidomorphus_muelleri:6.443629)94:6.361209)97:8.414333,((Pseudonaja_guttata:7.612514,(Oxyuranus_temporalis:7.609829,(Oxyuranus_scutellatus:5.295453,Oxyuranus_microlepidotus:5.719447)88:5.248006)100:5.244785)78:5.24905,(Pseudonaja_modesta:4.499654,(Pseudonaja_ingrami:6.797546,(Pseudonaja_textilis:4.47699,(Pseudonaja_inframacula:6.705353,(Pseudonaja_affinis:6.68586,Pseudonaja_nuchalis:6.688129)45:5.574005)89:6.984436)93:4.435973)98:4.446646)98:4.444464)100:4.445673)96:4.566554)65:2.78563,(((Furina_diadema:5.273197,Furina_ornata:5.271961)100:5.319767,((Simoselaps_bimaculatus:6.665607,((Simoselaps_minimus:6.97685,Simoselaps_anomalus:5.280767)100:5.270954,(Simoselaps_bertholdi:5.301477,Simoselaps_littoralis:7.388864)14:5.244653)100:5.266985)70:5.242866,(Brachyurophis_semifasciatus:5.319878,(Brachyurophis_approximans:7.668507,(Brachyurophis_australis:5.341187,(Brachyurophis_roperi:6.481904,(Brachyurophis_morrisi:6.459332,Brachyurophis_incinctus:6.882185)0:6.359058)100:7.148157)89:5.308292)21:5.498726)100:5.263693)99:5.249557)95:5.464718,((Denisonia_devisi:5.354592,((Elapognathus_coronatus:5.288712,(Rhinoplocephalus_bicolor:5.306871,Cryptophis_nigrescens:5.315337)99:5.280143)89:5.241227,((Parasuta_monachus:5.314849,Suta_fasciata:5.322025)50:5.246385,(Suta_suta:5.308095,Parasuta_spectabilis:5.296501)82:5.248765)100:5.276287)99:5.24595)99:4.391741,((Vermicella_calonotus:4.520399,(Vermicella_snelli:6.675644,(Vermicella_annulata:6.670135,(Vermicella_intermedia:4.477553,Vermicella_multifasciata:6.675766)100:4.444241)91:4.440076)100:4.44936)97:4.670471,(((Hemiaspis_signata:4.498202,Hemiaspis_damelii:4.500974)100:4.675505,(Echiopsis_curta:5.538093,((Drysdalia_rhodogaster:7.2322,(Drysdalia_mastersii:4.475258,Drysdalia_coronoides:4.472902)100:4.453418)100:4.789026,((Austrelaps_superbus:4.464107,Austrelaps_labialis:4.46391)100:4.799718,((Tropidechis_carinatus:3.880335,Notechis_scutatus:1.754967)100:1.755864,(Paroplocephalus_atriceps:5.289156,(Hoplocephalus_bitorquatus:4.475826,Hoplocephalus_bungaroides:7.819266)24:4.437878)99:4.781131)89:1.741126)31:1.739501)100:1.739537)80:1.735617)60:1.737562,((Emydocephalus_annulatus:4.483577,((Aipysurus_mosaicus:5.807477,Aipysurus_eydouxii:4.458991)100:4.636362,((Aipysurus_fuscus:4.649248,Aipysurus_laevis:4.461572)100:4.442248,(Aipysurus_apraefrontalis:4.459439,(Aipysurus_duboisii:4.455119,Aipysurus_foliosquama:6.644334)39:4.423076)100:4.625217)91:4.426691)99:3.371148)100:4.458749,((Ephalophis_greyae:4.480783,Parahydrophis_mertoni:4.491471)100:4.614927,(Hydrelaps_darwiniensis:4.489226,(Hydrophis_gracilis:4.665275,(Hydrophis_spiralis:4.649728,(Hydrophis_jerdonii:4.660458,((Hydrophis_belcheri:3.841229,(Hydrophis_cyanocinctus:3.837283,(Hydrophis_coggeri:3.833245,(Hydrophis_parviceps:3.833478,(Hydrophis_pacificus:5.183155,(Hydrophis_semperi:4.173768,Hydrophis_melanocephalus:4.179943)88:4.665169)0:3.917111)90:2.797563)97:2.799251)99:2.800458)100:3.619315,(Hydrophis_lapemoides:4.653331,((Hydrophis_platurus:4.460888,((Hydrophis_stokesii:3.644217,Hydrophis_elegans:3.644024)94:2.856672,(Hydrophis_donaldi:3.646708,Hydrophis_viperinus:3.644709)85:2.855577)95:2.798443)14:3.335266,(Hydrophis_curtus:4.461433,(((Hydrophis_caerulescens:3.638267,Hydrophis_brookii:3.640422)100:2.754167,((Hydrophis_stricticollis:3.642248,Hydrophis_obscurus:3.654951)98:2.751657,(Hydrophis_fasciatus:3.646579,Hydrophis_atriceps:3.638318)100:2.757526)42:2.797594)99:3.621148,((Hydrophis_major:3.647742,(Hydrophis_schistosus:2.831186,Hydrophis_zweifeli:2.826183)100:2.75139)93:3.616724,((Hydrophis_macdowelli:2.837579,Hydrophis_kingii:2.834664)57:2.751163,(Hydrophis_czeblukovi:5.376973,(Hydrophis_pachycercos:3.659089,(Hydrophis_peronii:3.646295,(Hydrophis_ornatus:2.829351,Hydrophis_lamberti:2.827163)98:2.748926)100:2.803086)86:1.9315)88:1.932883)93:3.614235)99:1.934239)65:2.99125)79:2.79679)97:2.798626)53:2.797287)81:4.423797)92:4.423474)100:4.439098)100:4.434501)76:3.36305)99:4.428722)100:4.664208)100:1.741951)50:1.739672)93:1.744626)67:1.737229)97:1.739597)99:1.748456)100:0.021413)76:0.005116)70:0.003864)88:0.002854)99:0.029198)98:0.014539)96:0.003919)99:0.005098)95:0.005242)97:0.004332)100:0.01375)91:0.00621,((((Scaphiodontophis_annulatus:4.665169,((Sibynophis_bistrigatus:5.695722,Sibynophis_subpunctatus:5.171076)100:4.604686,(Sibynophis_chinensis:5.179196,(Sibynophis_collaris:5.131803,Sibynophis_triangularis:5.130363)99:4.879314)100:4.537758)14:4.309385)99:3.207271,(((Macropisthodon_rudis:4.421221,((Amphiesma_stolatum:2.9319,Hebius_sauteri:3.639475)100:2.875244,((Herpetoreas_burbrinki:4.346131,Herpetoreas_platyceps:3.643455)93:3.746057,(Trachischium_monticola:4.686815,((Hebius_deschauenseei:3.637186,(Hebius_modestum:3.638343,Hebius_venningi:3.638673)31:3.61883)100:3.72526,((Hebius_boulengeri:3.644523,Hebius_khasiense:3.64451)100:3.714742,(Hebius_parallelum:3.738467,(Hebius_popei:3.744386,((Hebius_optatum:3.651199,Hebius_atemporale:3.649235)93:3.706981,((Hebius_vibakari:2.811896,Hebius_sp:2.911661)100:3.634437,((Hebius_octolineatum:4.138308,Hebius_bitaeniatum:2.818105)98:3.658582,(Hebius_metusium:4.169771,(Hebius_craspedogaster:2.816054,Hebius_johannis:2.850929)92:3.613171)94:2.81651)100:2.87244)91:3.613864)83:3.612803)94:3.617043)82:3.612501)95:3.620394)100:3.637113)57:3.619006)80:3.633951)90:3.447736)60:3.022616,((Natriciteres_olivacea:6.61665,Afronatrix_anoscopus:4.406276)89:3.784921,((Xenochrophis_schnurrenbergeri:6.573886,(Atretium_schistosum:4.783829,((Xenochrophis_asperrimus:4.763582,Atretium_yunnanensis:4.377951)94:2.572664,(Xenochrophis_piscator:3.386558,(Xenochrophis_punctulatus:6.127052,Xenochrophis_flavipunctatus:2.658739)79:2.404967)97:1.315799)90:0.021147)99:0.02716)100:0.046435,(Xenochrophis_vittatus:6.102402,((Rhabdophis_sp:5.641459,Rhabdophis_chrysargos:5.649976)100:6.896983,((Rhabdophis_himalayanus:6.393512,Balanophis_ceylonensis:4.563238)91:4.708867,(Macropisthodon_rhodolemas_ADM0003:5.506683,((Rhabdophis_tigrinus:4.114134,Rhabdophis_subminiatus:2.880105)56:2.806569,(Rhabdophis_swinhonis:5.914485,(Rhabdophis_nuchalis:3.634286,(Rhabdophis_guangdongensis:5.564143,(Rhabdophis_leonardi:5.552379,(Rhabdophis_nigrocinctus:5.568349,Rhabdophis_adleri:5.548196)100:5.550506)95:5.544996)64:5.895543)94:3.621011)98:4.071963)83:1.747796)49:3.03452)96:1.753068)84:1.741673)96:1.750552)100:0.653319)42:0.63563)100:0.638677,(((Aspidura_trachyprocta:5.271274,Aspidura_sp:5.272419)100:5.139068,(Aspidura_ceylonensis:5.352795,(Aspidura_guentheri:4.673117,Aspidura_drummondhayi:4.641325)100:5.128776)94:5.116143)100:5.362614,((Opisthotropis_guangxiensis:6.469793,(Opisthotropis_lateralis:5.832315,(Opisthotropis_cheni:3.666565,Opisthotropis_latouchii:5.832503)100:3.655153)86:4.267918)100:5.01716,((Sinonatrix_aequifasciata:6.312746,(Sinonatrix_percarinata:4.984537,Sinonatrix_annularis:4.634026)82:4.436579)100:5.340121,((Natrix_maura:5.848271,(Natrix_tessellata:6.010102,Natrix_natrix:2.921135)89:2.861479)100:3.347402,(((Liodytes_pygaea:3.964427,(Liodytes_rigida:3.938898,Liodytes_alleni:3.96328)100:3.938327)98:3.927553,((Clonophis_kirtlandii:3.957971,Virginia_valeriae:4.131976)99:4.395593,(Virginia_striatula:4.432356,(Storeria_dekayi:3.963554,(Storeria_storerioides:4.148113,Storeria_occipitomaculata:5.607306)89:4.716586)95:3.920963)98:3.917475)92:3.934566)100:3.108477,(((Tropidoclonion_lineatum:3.998506,Regina_grahamii:4.815522)65:4.736437,((Nerodia_cyclopion:3.981204,Nerodia_floridana:5.603419)100:4.748242,(Regina_septemvittata:5.24992,((Nerodia_erythrogaster:4.924273,(Nerodia_taxispilota:4.95211,Nerodia_rhombifer:4.483731)100:4.758259)56:4.422894,((Nerodia_fasciata:2.852695,Nerodia_clarkii:4.672691)99:3.356933,(Nerodia_sipedon:4.502739,(Nerodia_harteri:4.45615,Nerodia_paucimaculata:4.656171)96:4.94274)100:4.503173)99:3.06039)100:2.865546)96:2.859522)70:3.916396)98:3.088964,((Thamnophis_sirtalis:4.827254,(Thamnophis_proximus:3.991634,Thamnophis_sauritus:5.768507)100:3.95933)65:4.522701,(((Thamnophis_nigronuchalis:4.501668,Thamnophis_rufipunctatus:4.500619)99:6.03703,(Thamnophis_elegans:4.463437,((Adelophis_foxi:2.882167,(Thamnophis_melanogaster:4.480759,Thamnophis_valida:4.489814)74:4.447583)84:3.120579,((Thamnophis_exsul:4.456988,Thamnophis_godmani:4.88248)69:4.429619,(Thamnophis_scaliger:4.511992,(Thamnophis_sumichrasti:4.485166,Thamnophis_scalaris:4.482687)99:4.450327)100:4.731254)88:4.423196)86:1.889232)100:3.929659)99:4.145845,((Thamnophis_fulvus:4.520269,Thamnophis_chrysocephalus:4.874227)98:6.002439,((Thamnophis_pulchrilatus:4.649286,Thamnophis_cyrtopsis:4.491248)100:4.743613,((Thamnophis_eques:4.874634,Thamnophis_marcianus:1.804238)99:1.805049,((Thamnophis_hammondii:4.650936,(Thamnophis_brachystoma:4.641736,(Thamnophis_butleri:2.846438,Thamnophis_radix:4.469938)96:2.807562)100:2.804094)47:2.813625,(Thamnophis_ordinoides:4.491151,(Thamnophis_atratus:4.646858,(Thamnophis_gigas:4.639158,Thamnophis_couchii:4.478964)97:4.432229)100:4.431405)54:4.44961)65:2.005418)100:1.760486)100:2.86089)93:3.101836)64:2.920612)100:3.273241)67:2.857232)100:2.910099)100:3.673459)78:2.365625)99:2.374567)53:2.368796)100:0.677508)80:0.634482,((Plagiopholis_styani:6.029398,((Pseudoxenodon_bambusicola:3.401323,Pseudoxenodon_sp:6.392367)12:2.471654,(Pseudoxenodon_karlschmidti:5.402926,Pseudoxenodon_macrops:6.228454)0:4.569893)93:2.219922)100:2.67426,((Diadophis_punctatus:2.325586,((Heterodon_platirhinos:2.880535,(Heterodon_simus:4.983631,Heterodon_nasicus:4.700461)98:4.700948)100:2.896143,(((Farancia_erytrogramma:5.473796,Farancia_abacura:5.396321)100:5.388781,(Carphophis_vermis:5.814238,Carphophis_amoenus:5.407061)100:5.425751)99:5.429247,((Contia_tenuis:4.746848,Contia_longicaudae:5.513722)100:4.609069,(Stichophanes_ningshaanensis:5.423231,(Thermophis_baileyi:5.790826,(Thermophis_shangrila:5.589003,Thermophis_zhaoermii:4.749837)100:5.364707)100:5.397286)96:5.42551)75:3.501813)86:4.54701)14:2.808087)95:2.581642,((Enulius_sp:5.754426,((Amastridium_veliferum:5.231192,(Tantalophis_discolor:5.606062,((Rhadinaea_flavilata:6.221197,Rhadinaea_fulvivittis:4.968511)91:5.174381,(Coniophanes_fissidens:5.603399,(Pliocercus_euryzonus_ADM241:3.734722,Pliocercus_euryzonus_ADM240:3.461437)100:3.472269)91:2.367541)99:3.432687)46:3.432073)97:3.941669,((((Imantodes_inornatus:5.924741,Imantodes_cenchoa:3.530022)100:3.509156,(Imantodes_gemmistratus:6.10896,(Imantodes_lentiferus:5.47234,Imantodes_chocoensis:6.061676)98:4.580876)100:5.225712)94:3.523414,(Leptodeira_nigrofasciata:5.737288,((Leptodeira_frenata:4.621283,Leptodeira_uribei:4.621821)100:5.202664,(Leptodeira_punctata:5.289674,(Leptodeira_splendida:5.702675,((Leptodeira_polysticta:4.611034,Leptodeira_septentrionalis:4.666652)99:4.955562,((Leptodeira_bakeri:5.652413,Leptodeira_annulata:2.220015)100:2.191423,(Leptodeira_maculata:4.63257,Leptodeira_rubricata:4.627316)100:5.602579)95:2.184021)100:1.150338)90:2.187106)56:1.120339)95:2.194232)89:2.449888)100:1.292054,((Pseudoleptodeira_latifasciata:5.037542,(Hypsiglena_tanzeri:5.425481,((Hypsiglena_jani:4.629671,Hypsiglena_slevini:4.627947)53:5.293152,((Hypsiglena_unaocularus:4.327353,Hypsiglena_chlorophaea:3.976583)96:4.252339,(Hypsiglena_ochrorhyncha:4.306671,(Hypsiglena_affinis:4.769494,Hypsiglena_torquata:3.93243)96:3.923808)97:3.274108)100:4.55806)99:3.497517)97:4.744727)100:3.746558,((Adelphicos_quadrivirgatum:5.485935,(Hydromorphus_concolor:5.468748,Tretanorhinus_nigroluteus:5.472253)100:5.375633)76:6.327889,(Cryophis_hallbergi:5.502146,(((Geophis_turbidus:5.156631,(Geophis_dubius:6.054814,(Geophis_juarezi:5.938041,Geophis_carinosus:5.569997)100:4.855657)98:5.512161)100:5.381091,((Geophis_occabus:4.897794,Geophis_latifrontalis:4.887672)11:5.083071,(Geophis_godmani:5.160687,((Atractus_wagleri:4.531189,Atractus_reticulatus:5.344459)0:4.42339,((Atractus_badius:5.999256,(Atractus_zidoki:4.881282,Atractus_schach:5.613554)31:4.725288)87:6.528505,((Atractus_elaps:3.896754,Atractus_albuquerquei:4.100399)86:3.924666,(Atractus_flammigerus:6.287372,(Atractus_trihedrurus:5.237556,Atractus_zebrinus:5.908382)95:3.613177)88:5.1368)90:3.91855)74:3.932224)98:3.925275)84:3.922788)43:3.914123)87:4.762901,(Ninia_atrata:4.855051,((Sibon_noalamina:6.076179,(Tropidodipsas_fischeri_MVZ143527:3.062269,(Tropidodipsas_sartorii:5.261335,((Sibon_dimidiatus_LSUMNS6689:4.505841,Sibon_nebulatus:3.951672)88:4.117731,(Sibon_annulatus_ADM242:2.83891,Sibon_annulatus_ADM0007:2.83206)100:3.039764)61:2.803462)80:2.799191)87:2.800673)0:2.860855,((Dipsas_articulata:6.565232,(Dipsas_indica:6.1995,Dipsas_pratti:4.750423)0:3.982657)85:4.11817,((Dipsas_peruana_LSUMNS1532:4.014256,(Sibynomorphus_vagus_KU219121:5.122011,Dipsas_pavovina_LSUMNS14372:4.337112)84:3.943002)56:4.349276,(((Sibynomorphus_turgidus:6.623528,Sibynomorphus_mikanii:4.79866)82:4.762917,(Sibynomorphus_turgidus_LSUMNS6459:4.149694,Thamnodynastes_pallidus:4.800834)100:4.050049)100:4.08322,((Dipsas_catesbyi:4.152008,Dipsas_pavovina_LSUMNS13989:3.425771)100:2.825877,(Dipsas_albifrons:6.782193,((Sibynomorphus_ventrimaculatus:5.633621,Sibynomorphus_neuwiedi:5.995932)87:5.637851,(Dipsas_neivai:5.619496,Dipsas_variegata:5.98393)100:5.793855)50:7.650536)53:5.724861)94:2.802381)81:2.797038)88:2.801203)99:2.837075)31:3.428047)100:3.439647)98:3.434029)94:3.431185)97:3.435728)79:3.660825)99:0.013031)94:0.008511)98:0.008132,(((Pliocercus_elapoides:5.064745,Manolepis_putnami:4.655786)100:4.651265,(Conophis_lineatus:4.667746,Conopsis_nasus:5.213785)100:4.64607)52:4.924027,(((Caaeteboia_amarali:5.599665,(Hydrodynastes_bicinctus:5.412943,Hydrodynastes_gigas:5.400613)100:5.479262)69:6.143091,(Philodryas_chamissonis:5.280474,(Philodryas_trilineata:5.281749,(Xenopholis_undulatus:5.744628,Xenopholis_scalaris:5.410761)0:5.821627)0:4.549496)0:4.616557)96:2.924587,(((Tropidodryas_striaticeps:4.691024,Tropidodryas_serra:4.756191)100:5.070759,(((Pseudoeryx_plicatilis:4.376394,Hydrops_triangularis:4.317219)22:4.591782,(Helicops_infrataeniatus:4.414343,(Helicops_hagmanni:4.818423,(Helicops_carinicaudus:3.77832,(Helicops_gomesi:4.609856,Helicops_angulatus:1.090271)89:1.082939)95:1.69944)87:1.715879)97:2.831956)100:1.80548,((Gomesophis_brasiliensis:4.388789,((Tachymenis_peruviana:2.9791,Pseudotomodon_trigonatus:3.04374)99:3.302873,(Calamodontophis_paucidens:3.382617,(Ptychophis_flavovirgatus:3.377733,(Tomodon_dorsatum:2.8629,((Thamnodynastes_strigatus:3.270001,Thamnodynastes_rutilus:4.488366)99:2.8935,(Thamnodynastes_hypoconia_KU290727:2.99741,(Thamnodynastes_strigatus_LSUMNS6563:3.036253,(Thamnodynastes_hypoconia:3.459095,Thamnodynastes_lanei:2.98637)0:3.200115)96:3.208828)92:2.866222)96:1.740443)81:2.804633)89:1.737617)83:1.754373)78:3.426533)96:3.493007,(Siphlophis_compressus:3.56402,((Siphlophis_longicaudatus:5.31189,Siphlophis_pulcher:3.29095)100:2.889529,(Siphlophis_cervinus:3.698763,((((Ninia_sebae:5.0956,Drepanoides_anomalus:4.533877)100:4.508993,(Oxyrhopus_fitzingeri_LSUMNS6586:5.421687,(Phimophis_guerini:6.106941,Paraphimophis_rusticus:4.540101)95:4.50314)75:3.618791)17:3.615357,(Rodriguesophis_iglesiasi:4.506665,(Boiruna_maculata:3.65685,(Rhachidelus_brazili:4.15807,(Pseudoboa_neuwiedii:4.968075,(Pseudoboa_nigra:2.972915,Pseudoboa_coronata:5.370186)86:3.611889)97:3.615273)4:3.615084)94:3.610609)14:3.614235)100:2.808033,(Oxyrhopus_petolarius:4.041358,(Oxyrhopus_clathratus:4.772087,(((Oxyrhopus_formosus_LSUMNS17730:3.651075,Oxyrhopus_formosus_LSUMNS17734:4.012654)0:3.825068,(Oxyrhopus_formosus:4.974578,Oxyrhopus_formosus_KU214892:3.852972)38:3.918105)96:3.622113,(Oxyrhopus_trigeminus:4.850152,(Oxyrhopus_rhombifer:4.76792,((Oxyrhopus_melanogenys:4.834574,Oxyrhopus_guibei:3.904712)73:3.682075,(Oxyrhopus_trigeminus_LSUMNS14425:4.081643,Oxyrhopus_trigeminus_LSUMNS14365:3.9948)0:4.133322)90:2.79874)46:2.801222)78:2.801138)90:2.799756)75:2.799138)97:2.802417)100:2.809597)55:2.802096)100:2.384913)100:3.531449)95:4.591781)77:1.774789)79:2.005968,(((((Phalotris_bilineatus:4.706027,Phalotris_lemniscatus:4.27603)100:4.304687,(Phalotris_mertensi:5.225159,(Phalotris_lativittatus:4.756177,Phalotris_nasutus:4.421733)98:4.35415)100:4.312003)98:6.171032,(Elapomorphus_quinquelineatus:4.296069,((Apostolepis_assimilis:4.346596,(Apostolepis_sanctaeritae:4.086457,Apostolepis_cearensis:3.716449)40:3.493867)97:3.817051,(Apostolepis_nigrolineata:5.207206,(Apostolepis_flavotorquata:3.721674,(Apostolepis_dimidiata:3.648417,Apostolepis_albicollaris:4.207128)96:2.812715)66:3.433891)87:3.428269)100:3.441371)73:3.495525)100:3.495251,((Sordellina_punctata:4.297947,((Echinanthera_melanostigma:4.722372,Echinanthera_undulata:4.284538)91:4.239862,(Taeniophallus_affinis:4.384204,(Taeniophallus_nicagus:4.142395,Taeniophallus_brevirostris:3.655768)100:4.36384)77:5.006837)98:4.24427)96:5.363945,(Philodryas_viridissima:3.926312,((Philodryas_baroni:4.015475,(Philodryas_patagoniensis:4.3709,Philodryas_olfersii:2.839982)97:3.52664)89:4.243335,((Philodryas_mattogrossensis:3.253284,Philodryas_nattereri:2.849751)88:2.983435,((Philodryas_georgeboulengeri:3.451868,Philodryas_argentea:2.845537)100:2.921304,(Philodryas_agassizii:2.94265,(Philodryas_psammophidea:3.246788,Philodryas_aestiva:2.874523)99:3.204076)95:2.878495)38:2.798068)19:3.104144)93:2.3729)98:4.549203)42:4.555212)40:4.843269,((Pseudalsophis_elegans:5.575042,Pseudalsophis_dorsalis:4.667479)100:4.955264,(((Uromacer_catesbyi:2.623085,(Uromacer_frenatus:1.830978,Uromacer_oxyrhynchus:1.829977)99:1.734394)100:2.445576,(Clelia_clelia:3.800341,(((Lygophis_elegantissimus:3.546473,Lygophis_anomalus:3.938166)100:3.838522,(Lygophis_lineatus:2.982971,(Lygophis_paucidens:4.064618,(Lygophis_meridionalis:3.676144,Lygophis_flavifrenatus:4.063651)89:2.803519)72:3.738139)76:2.387013)99:3.747084,(((Xenodon_severus:3.195978,(Xenodon_werneri:4.595005,Xenodon_merremi:2.830747)83:2.812464)98:3.445207,(Xenodon_neuwiedii:4.064751,((Xenodon_dorbignyi:5.096925,(Xenodon_nattereri:5.432644,Xenodon_histricus:3.632103)48:2.801696)92:2.811384,(Xenodon_guentheri:5.094383,(Xenodon_semicinctus:3.65188,(Xenodon_matogrossensis:3.686105,Xenodon_pulcher:3.685782)37:4.199263)99:2.82683)74:3.158016)100:1.745711)90:2.369945)100:3.459576,(Erythrolamprus_breviceps:5.348853,((Erythrolamprus_miliaris:2.831546,((Erythrolamprus_ceii:2.861835,Erythrolamprus_poecilogyrus:3.251671)98:4.287555,(Erythrolamprus_mimus:4.736695,Erythrolamprus_aesculapii:2.82797)100:2.808376)45:2.8017)93:2.802765,((Erythrolamprus_atraventer:3.67671,(Erythrolamprus_almadensis:3.662568,Erythrolamprus_jaegeri:3.657144)92:2.821416)98:2.80501,((Erythrolamprus_cursor:3.698832,Erythrolamprus_juliae:3.695821)88:3.981128,(Erythrolamprus_typhlus:3.683366,(Erythrolamprus_epinephelus:3.706531,Erythrolamprus_pygmaeus:3.700246)50:3.979459)74:3.607099)78:3.609971)84:3.196456)87:2.366854)100:3.44029)96:3.662976)100:2.374125)100:2.456106)92:0.634392,((Arrhyton_taeniatum:1.827901,(Arrhyton_supernum:1.799625,((Arrhyton_redimitum:2.588259,Arrhyton_vittatum:2.582403)17:2.515658,(Arrhyton_procerum:2.579702,(Arrhyton_dolichura:2.556773,Arrhyton_tanyplectum:2.563537)20:2.526656)100:2.518147)98:2.534497)99:1.707859)100:2.087586,((Psomophis_obtusus:6.29901,(Psomophis_genimaculatus:5.177308,Psomophis_joberti:3.504653)98:3.4485)100:3.632322,(((Alsophis_sibonius:3.195157,(Alsophis_manselli:3.161809,Alsophis_antillensis:2.550798)100:1.736432)99:1.734981,(Alsophis_rijgersmaei:1.775743,(Alsophis_rufiventris:2.557663,(Alsophis_sajdaki:4.834151,Alsophis_antiguae:5.245915)0:3.434116)100:1.713406)99:1.814762)100:0.92537,((Magliophis_exiguum:1.791398,Magliophis_stahli:2.669208)100:1.879313,((Borikenophis_variegatus:2.723616,Borikenophis_portoricensis:1.781585)100:1.808425,(((Hypsirhynchus_callilaemus:1.792433,(Hypsirhynchus_funereus:2.562443,Hypsirhynchus_polylepis:2.559408)100:2.538523)100:2.085551,(Hypsirhynchus_parvifrons:2.11756,(Hypsirhynchus_scalaris:2.892644,Hypsirhynchus_ferox:2.528627)100:2.605836)99:1.713577)100:1.704684,((Ialtris_dorsalis:1.857355,Ialtris_haetianus:1.866622)100:2.167384,((Haitiophis_anomalus:5.542128,Caraiba_andreae:1.833306)84:2.18225,(Cubophis_cantherigerus:1.102862,(Cubophis_vudii:2.530851,(Cubophis_caymanus:2.563844,(Cubophis_fuscicauda:4.278019,Cubophis_ruttyi:4.279893)9:3.452888)100:2.503471)32:1.767986)100:1.110978)99:1.074821)51:1.066511)61:1.064349)89:1.069656)71:1.069881)97:1.073237)79:0.007295)85:0.273042)89:1.131779)100:1.125104)86:1.126587)91:1.129488)97:1.143019)100:1.289454)100:0.010703)100:0.01387)82:0.013774)97:0.003818,(((Grayia_tholloni:6.055038,(Grayia_ornata:2.573797,Grayia_smithii:6.014574)81:2.218664)100:2.20842,(Pseudorabdion_longiceps_ADM0004:5.44045,(Pseudorabdion_oxycephalum:6.786595,(Calamaria_yunnanensis:6.648732,Calamaria_pavimentata:2.886621)100:2.227703)74:2.184311)100:2.207794)70:2.629354,((((Dryophiops_rubescens_110_LSUHC6264:5.618179,(Dryophiops_philippina_KU321727:4.291056,Dryophiops_philippina_KU328968:5.623175)84:4.254492)100:4.644947,(((Ahaetulla_fronticincta:6.45324,(Ahaetulla_fronticincta_CAS222635:3.733602,Ahaetulla_fronticincta_CAS245687:4.60557)71:4.250331)100:4.84549,(Ahaetulla_nasuta:4.806362,(Ahaetulla_nasuta_FMNH255023:4.026427,Ahaetulla_pulverulenta:3.614155)88:3.182212)35:3.187655)99:3.190726,(Ahaetulla_prasina_57_LSUHC8586:4.316654,((Ahaetulla_prasina:7.01541,(Ahaetulla_fasciolata_55_LSUHC8837:3.624246,Ahaetulla_fasciolata_54_LSUHC9831:3.624661)100:3.630727)97:3.621801,(Ahaetulla_prasina_FMNH269042:3.97181,(Ahaetulla_mycterizans_56_LSUHC8913:3.631941,Ahaetulla_mycterizans_ADM0001:3.625586)98:3.622862)100:3.626009)97:4.286371)100:4.599608)100:3.212381)99:3.187767,((Chrysopelea_taprobanica:5.496533,((Chrysopelea_pelias_71_LSUHC7296:4.252481,Chrysopelea_pelias_70_LSUHC3900:4.252402)100:4.637281,(Chrysopelea_paradisi:6.473674,(Chrysopelea_ornata_69_LSUHC8851:4.28516,Chrysopelea_ornata:3.99509)86:3.197625)98:3.197731)100:3.200919)100:3.205403,((Dendrelaphis_bifrenalis:5.498238,(Dendrelaphis_caudolineolatus:7.293105,((Dendrelaphis_caudolineatus:6.670225,(Dendrelaphis_fulginosus_KU304098:5.145088,Dendrelaphis_fulginosus_KU302993:3.81121)97:3.620565)100:3.874946,(Dendrelaphis_schokari:5.024681,(Dendrelaphis_subocularis_77_LSUHC7429:5.18035,Dendrelaphis_tristis:4.6512)100:3.884411)92:3.668488)97:3.19078)58:3.180307)56:3.181024,((Dendrelaphis_formosus_FMNH267935:4.273988,Dendrelaphis_formosus_ADM0002:4.272289)100:4.292335,(((Dendrelaphis_striatus_79_LSUHC4792:3.639717,Dendrelaphis_striatus_78_LSUHC10012:3.633184)100:3.652211,(Dendrelaphis_cyanochloris_75_LSUHC6768:3.66366,(Dendrelaphis_cyanochloris_CAS221428:3.640774,(Dendrelaphis_ngansonensis_AMNH148550:2.84979,Dendrelaphis_ngansonensis_AMNH147134:2.857237)98:3.956402)100:2.830004)46:3.615833)100:3.628018,((Dendrelaphis_pictus_CAS210338:3.629745,Dendrelaphis_pictus_CAS222690:3.636509)100:3.648385,(Dendrelaphis_haasi_76_LSUHC10042:3.656582,(Dendrelaphis_marenae_KU324552:3.628105,(Dendrelaphis_pictus:4.386866,Dendrelaphis_marenae_KU324549:2.827783)97:3.810012)100:2.81717)99:3.625077)98:2.574635)98:4.245953)100:3.572256)100:3.193932)100:3.184001)100:3.234189,(((((Oligodon_arnensis:6.8116,(Oligodon_taeniolatus:5.287907,(Oligodon_calamarius:5.150845,Oligodon_sublineatus:5.156477)100:5.200234)99:5.871189)0:5.113252,((Oligodon_sp:8.216568,((Oligodon_formosanus:6.208587,Oligodon_chinensis:6.196365)98:6.371609,((Oligodon_ocellatus:5.611341,Oligodon_cyclurus:5.617189)82:4.952338,(Oligodon_octolineatus:5.708539,(Oligodon_taeniatus:4.496975,Oligodon_barroni:4.493861)96:4.823726)53:4.807061)56:6.854544)100:5.794319)100:6.196946,((Oligodon_cinereus:7.131202,(Oligodon_splendidus:6.580438,Oligodon_maculatus:6.215144)91:6.258412)97:6.933463,((Oligodon_planiceps:6.258968,Oligodon_torquatus:6.2617)96:6.192502,(Oligodon_venustus:7.59874,(Oligodon_modestus:6.241256,(Oligodon_cruentatus:4.51117,Oligodon_theobaldi:4.512557)0:4.815379)0:6.107248)0:6.193699)4:6.791136)76:5.114256)96:6.390752)100:5.336242,(((Thrasops_flavigularis_MVZ253440:6.250709,Thrasops_jacksonii:6.490976)0:5.860629,((Thelotornis_capensis:7.3728,Thelotornis_capensis_LSUMNS4846:5.742702)93:5.596579,(Dispholidus_typus:7.560105,(Thelotornis_kirtlandii_YPM012649:4.288295,Thelotornis_kirtlandii_MVZ252602:5.930149)96:4.259137)95:4.27508)100:4.262007)95:3.386057,((Hapsidophrys_lineatus:5.996113,(Hapsidophrys_smaragdina:2.225728,Hapsidophrys_principis:5.984966)99:2.193653)99:2.2003,((Philothamnus_hoplogaster:6.582216,(Philothamnus_heterodermus:7.018007,Philothamnus_carinatus:7.378286)99:6.178909)31:5.456847,((Philothamnus_natalensis:6.288369,(Philothamnus_thomensis:7.046336,Philothamnus_girardi:7.05015)100:6.209898)92:5.7825,(Philothamnus_semivariegatus:6.643955,(Philothamnus_nitidus:6.642767,(Philothamnus_irregularis_KU290488:6.278362,(Philothamnus_angolensis:4.88602,(Philothamnus_natalensis_MVZ233358:3.644472,Philothamnus_natalensis_LSUMNS20681:4.304459)0:3.61996)67:5.372505)95:5.366911)97:5.368055)99:5.386916)68:4.300564)100:5.506779)100:1.355162)98:0.215584)89:0.03012,(Scaphiophis_albopunctatus:6.927783,(Lytorhynchus_diadema:7.128149,((Hemerophis_zebrinus:7.275316,(Bamanophis_dorri:6.437695,(Macroprotodon_mauritanicus:5.947268,(Macroprotodon_abubakeri:4.82477,(Macroprotodon_brevis:5.919346,Macroprotodon_cucullatus:4.439926)95:4.734471)78:4.739281)93:5.408157)54:6.178221)90:6.469032,(Hemerophis_socotrae:6.575206,((((Hemorrhois_ravergieri:4.007936,Hemorrhois_nummifer:3.678884)98:3.638326,(Hemorrhois_hippocrepis:3.680525,Hemorrhois_algirus:3.685455)100:3.652156)100:4.792317,((Spalerosophis_microlepis:4.769049,Spalerosophis_diadema:3.666795)15:3.700112,(Platyceps_rhodorachis:4.064588,(Platyceps_karelini:3.687185,((Platyceps_elegantissimus:3.581481,Platyceps_florulentus:2.834088)65:3.660156,(Platyceps_ventromaculatus:4.42033,(Platyceps_variabilis:4.427212,(Platyceps_najadum:2.849456,Platyceps_collaris:2.857955)50:3.619682)77:2.797625)78:2.802266)93:4.006386)86:2.80052)100:2.832255)95:4.77124)100:4.961131,((Dolichophis_jugularis:4.489205,(Hierophis_andreanus:5.197758,(Dolichophis_caspius:3.627909,Dolichophis_schmidti:3.638564)90:3.631229)68:3.622919)100:4.991182,((Orientocoluber_spinalis:4.481429,(Hierophis_gemonensis:4.457408,(Hierophis_viridiflavus:3.641588,Dolichophis_cypriensis:4.367982)74:3.624838)100:2.822494)100:3.841101,((Eirenis_aurolineatus:4.659272,Eirenis_modestus:4.456281)100:3.890545,(Eirenis_persicus:4.870057,(Eirenis_decemlineatus:4.871815,((Eirenis_lineomaculatus:3.758381,(Eirenis_coronelloides:3.649457,Eirenis_rothii:3.654037)81:3.724543)83:3.830101,((Eirenis_medus:4.492873,(Eirenis_thospitis:3.976768,Eirenis_hakkariensis:3.639529)95:3.825961)93:2.829678,(Eirenis_levantinus:4.693717,(Eirenis_punctatolineatus:4.510821,(Eirenis_barani:4.535834,(Eirenis_collaris:3.664957,Eirenis_eiselti:4.000325)100:3.628364)40:2.975815)95:2.804469)11:2.803527)86:3.613437)96:2.819188)85:2.937419)81:2.804218)100:2.81262)87:4.85503)100:3.688229)21:6.224543)99:5.110196)95:5.708992)27:6.173734)96:5.50679)32:0.005063,(((Coelognathus_radiatus:6.515696,(Coelognathus_helena:7.276749,(Coelognathus_erythrurus:6.50481,(Coelognathus_subradiatus:6.393868,Coelognathus_flavolineatus:6.382382)100:6.440935)100:7.128379)92:6.40741)100:7.172331,(((Gonyosoma_jansenii:5.999732,Gonyosoma_oxycephalum:5.581362)100:5.847811,(Gonyosoma_prasinus:6.142068,(Gonyosoma_margaritatus:5.629378,(Gonyosoma_boulengeri:5.503677,Gonyosoma_frenatus:5.947198)100:4.608211)92:5.248037)100:4.929908)99:4.69625,((((Lepturophis_albofuscus_84_LSUHC4588:3.648399,Lepturophis_albofuscus_83_LSUHC3867:3.634217)100:3.999283,((Lycodon_dumerilii:6.800561,(Lycodon_muelleri:6.794193,(Lycodon_bibonius:6.782168,(Lycodon_alcalai:6.777174,(Lycodon_sp:3.376806,Lycodon_chrysoprateros:5.96972)77:4.175459)88:4.170438)100:4.180911)92:4.173737)99:4.732751,((Dryocalamus_tristrigatus_FMNH269033:3.652485,(Dryocalamus_subannulatus_82_LSUHC5576:3.639424,Dryocalamus_subannulatus_81_LSUHC5051:3.628437)100:3.633631)100:3.975029,(Dryocalamus_nympha:4.746546,(Dryocalamus_davisonii_FMNH255034:3.670586,(Dryocalamus_davisonii_80_LSUHC8479:3.635388,Dryocalamus_davisonii_KU328512:4.968572)48:3.618457)100:3.973814)89:2.563129)95:2.563121)92:2.555237)91:2.579589,((Lycodon_effraenis:7.212512,(Lycodon_carinatus:5.525204,(Lycodon_laoensis:6.617759,(Lycodon_jara:7.130129,(Lycodon_osmanhilli:5.037375,(Lycodon_zawi:5.806181,(Lycodon_aulicus:4.803209,Lycodon_capucinus:4.959522)100:5.566453)0:6.037151)91:5.307452)96:5.541609)72:5.285176)85:5.301966)93:5.524004,((Lycodon_futsingensis:6.901157,(Lycodon_semicarinatus:6.527658,(Lycodon_rufozonatus:4.752306,(Lycodon_paucifasciatus:5.931669,Lycodon_flavozonatus:6.013824)0:5.707243)51:5.537226)78:5.564156)96:5.731163,(Lycodon_subcinctus:7.18639,(Lycodon_synaptor:5.697443,((Lycodon_ruhstrati:5.593013,(Lycodon_multizonatus:5.584273,Lycodon_liuchengchaoi:5.582372)100:5.965468)95:5.728286,(Lycodon_gongshan:4.450517,(Lycodon_fasciatus:5.777695,(Lycodon_butleri:4.772381,Lycodon_cavernicolus:4.779536)94:5.730925)79:4.444308)100:4.562123)56:4.177187)100:4.178487)49:5.257315)94:4.629081)91:3.355804)100:1.743205,(((Telescopus_fallax:5.953271,(Telescopus_beetzi_MVZ226830:3.815923,(Telescopus_dhara:5.966866,Telescopus_semiannulatus_LSUMNS6065:4.922303)88:4.959507)100:3.648735)80:3.661882,((Crotaphopeltis_tornieri:5.59623,Crotaphopeltis_hotamboeia:5.595287)100:6.217007,((Dipsadoboa_duchesnii_CAS197901:5.005728,Dipsadoboa_brevirostris_MVZ245373:4.489748)96:3.992215,(Dipsadoboa_shrevei:6.04823,(Dipsadoboa_weileri_MVZ253208:5.618554,Dipsadoboa_unicolor:6.009848)100:5.567642)0:5.763296)89:3.613551)100:2.74752)79:2.776985,(Toxicodryas_pulverulenta:5.478978,(((Toxicodryas_blandingii_LSUMNS20224:4.776301,Toxicodryas_pulverulenta_CAS220642:5.972971)84:4.934389,((Dasypeltis_confusa:6.314956,Dasypeltis_fasciata_YPM013202:4.781458)63:4.772466,((Dasypeltis_atra_CAS201640:5.13747,(Dasypeltis_scabra:4.910107,Dasypeltis_latericia:6.087822)0:5.76247)83:4.757481,((Dasypeltis_atra:5.57104,Dasypeltis_atra_CAS201642:4.764688)0:5.068387,((Dasypeltis_gansi:5.648663,Dasypeltis_fasciata:5.649029)79:6.440668,(Dasypeltis_sahelensis:4.9014,Dasypeltis_sp:4.899974)91:5.629126)0:6.194359)0:3.672957)100:3.688813)100:3.846999)97:5.124277,((Boiga_kraepelini:6.206422,(Boiga_jaspidea_62_LSUHC7679:4.950315,Boiga_jaspidea_63_LSUHC7656:3.641782)100:3.657254)72:4.00508,((Boiga_barnesii:4.736107,(Boiga_beddomei:4.741795,(Boiga_ceylonensis:4.732834,(Boiga_trigonata:4.730182,(Boiga_multomaculata:5.348668,(Boiga_quincunciata_CAS221434:3.231014,Boiga_quincunciata_CAS235862:2.858906)0:3.614045)53:3.176753)70:1.741139)5:2.555186)93:2.554873)100:2.570511,((Boiga_drapiezii_60_LSUHC8157:3.641779,Boiga_drapiezii_61_LSUHC7295:4.883177)100:4.03293,(Boiga_irregularis:6.171649,(((Boiga_nigriceps_65_LSUHC4494:2.822574,Boiga_nigriceps_64_LSUHC7020:2.819636)100:3.674567,(Boiga_dendrophila:6.203374,(Boiga_cyanea_59_LSUHC6820:2.859263,Boiga_cyanea_58_LSUHC7879:2.856819)87:3.927303)98:2.844483)81:3.983638,(Boiga_forsteni:4.075086,(Boiga_cynodon:5.127726,((Boiga_siamensis_CAS210147:2.831117,Boiga_siamensis_CAS215627:2.828204)79:2.81054,(Boiga_siamensis_67_LSUHC8527:2.822649,Boiga_siamensis_66_LSUHC8502:2.822952)100:2.81516)100:3.000885)15:3.817759)99:2.560094)100:1.73542)55:2.553866)100:2.559392)96:2.555964)98:2.555416)88:2.551816)86:2.578224)100:2.560322)100:1.742462)84:1.735235)77:1.736332,(((Euprepiophis_mandarinus:6.938532,(Euprepiophis_conspicillata:6.823562,Euprepiophis_perlacea:6.768853)99:6.434595)100:7.401673,(Oreocryptophis_porphyraceus:5.846712,(Archelaphe_bella:6.44534,((Elaphe_zoigeensis:6.409932,((Elaphe_quatuorlineata:5.572496,Elaphe_sauromates:6.763603)100:5.574943,((Elaphe_davidi:6.763064,(Elaphe_dione:4.442009,Elaphe_bimaculata:4.434484)100:6.025552)94:5.544429,(Elaphe_climacophora:6.858829,(Elaphe_carinata:5.291547,(Elaphe_quadrivirgata:5.732205,(Elaphe_anomala:4.791309,Elaphe_schrenckii:4.87814)100:3.655578)89:4.42941)83:5.556272)59:5.542439)68:4.494032)100:5.561938)97:5.757912,((Orthriophis_hodgsoni:6.043676,(Orthriophis_cantoris:6.829311,(Orthriophis_taeniurus:6.412346,Orthriophis_moellendorffi:6.819196)92:5.560848)95:5.55421)100:5.771088,(((Zamenis_hohenackeri:6.409367,Zamenis_persicus:6.404364)89:5.602827,(Rhinechis_scalaris:6.447707,(Zamenis_situla:6.083431,(Zamenis_lineatus:4.544493,(Zamenis_longissimus:3.618366,Ptyas_korros:3.702926)100:4.616115)99:5.195643)99:5.60321)93:5.560098)99:5.118978,((Oocatochus_rufodorsatus:6.423687,(Coronella_austriaca:5.296486,Coronella_girondica:5.295849)94:5.553346)97:5.772727,(Senticolis_triaspis:6.326038,(((Rhinocheilus_lecontei:4.49753,(Pseudelaphe_flavirufa:4.494877,Arizona_elegans:3.678237)44:3.622487)99:4.072518,((Pantherophis_bairdi:6.521,(Pantherophis_alleghaniensis:6.02236,(Pantherophis_spiloides:5.924996,Pantherophis_obsoletus:5.251878)95:5.314664)99:5.989845)100:5.270185,(((Pantherophis_vulpinus:5.25598,Pantherophis_ramspotti:5.475896)100:5.810918,(Pantherophis_guttatus:5.36766,(Pantherophis_slowinskii:4.451483,Pantherophis_emoryi:5.096201)89:5.310219)100:5.259977)79:5.245682,((Pituophis_lineaticollis:5.653307,Pituophis_deppei:5.711064)49:5.233392,(Pituophis_vertebralis:5.275405,(Pituophis_catenifer:4.665204,(Pituophis_ruthveni:4.437348,Pituophis_melanoleucus:4.438373)24:4.432712)99:5.258437)96:5.669458)100:4.343679)30:6.042517)100:4.176038)65:3.82594,((Bogertophis_subocularis:4.551352,Bogertophis_rosaliae:4.550529)100:4.770003,(Cemophora_coccinea:3.874656,(Lampropeltis_calligaster:4.070109,((Lampropeltis_zonata:3.855422,((Lampropeltis_elapsoides:3.909563,(Lampropeltis_webbi:3.90539,(Lampropeltis_knoblochi:3.894894,Lampropeltis_pyromelana:3.677018)97:3.633168)83:3.618572)51:3.055633,((Lampropeltis_mexicana:3.646452,Lampropeltis_ruthveni:3.636068)43:3.621752,(Lampropeltis_micropholis:3.639758,(Lampropeltis_polyzona:3.635965,Lampropeltis_abnorma:3.635459)99:2.860788)99:3.841461)96:3.626696)91:3.617925)99:3.828452,(Lampropeltis_triangulum:3.850948,((Lampropeltis_alterna:3.63164,(Lampropeltis_gentilis:3.622733,Lampropeltis_annulata:3.622631)38:3.640571)100:4.026129,(Lampropeltis_extenuata:3.843245,((Lampropeltis_splendida:3.627548,Lampropeltis_californiae:3.626512)100:3.265513,(Lampropeltis_getula:1.744051,(Lampropeltis_holbrooki:3.631349,Lampropeltis_nigra:3.625787)66:3.150785)70:1.740384)99:1.751789)91:1.744537)90:1.739576)100:1.758859)97:1.747407)90:1.739615)100:1.79009)76:2.146208)100:2.867063)99:2.860108)89:2.85497)91:2.853187)41:2.853422)100:2.862632)24:2.854928)85:2.857249)100:3.041245,((Liopeltis_frenatus_CAS225548:3.301801,(((Ptyas_mucosa:4.659206,(Ptyas_carinata_91_LSUHC10004:4.163175,Ptyas_carinata_92_LSUHC7600:2.826913)100:3.017175)100:3.023922,(Cyclophiops_major:4.249747,(Ptyas_dhumnades:5.142348,(Ptyas_nigromarginata_CAS236202:3.964227,Ptyas_nigromarginata_CAS241947:3.947503)96:4.094338)78:4.151129)99:1.958691)19:1.746498,(((Cyclophiops_multicinctus_FMNH255569:2.841838,Cyclophiops_multicinctus_AMNH153707:2.823081)99:3.007812,(Cyclophiops_multicinctus:4.160849,Cyclophiops_multicinctus_KU291939:3.025318)87:1.734893)100:1.754733,((Ptyas_fusca_ADM0006:2.840304,Ptyas_fusca_FMNH269019:2.846247)100:2.819515,(Ptyas_luzonensis:5.327489,(Ptyas_luzonensis_KU330134:4.141416,Ptyas_luzonensis_KU329295:4.0043)97:2.809422)100:2.829999)95:3.013361)100:1.942153)95:1.742004)97:1.741491,(((Dendrophidion_percarinatum:5.344904,(Drymobius_rhombifer:4.986755,(Dendrophidion_dendrophis:5.370236,(Dendrophidion_percarinatum_MVZ204098:3.632553,Dendrophidion_percarinatum_MVZ204099:5.347716)100:3.63704)4:3.804352)44:3.625417)100:3.638398,(((Oxybelis_fulgidus:6.014873,Oxybelis_aeneus:5.692231)99:5.834426,((Leptophis_depressirostris_YPM016868:5.231314,Leptophis_depressirostris_LSUMNS146385:5.145054)98:4.935908,(Leptophis_diplotropis_LSUMNS6328:3.663466,Leptophis_ahaetulla:5.810298)7:3.627521)100:3.625737)97:2.566969,((Opheodrys_aestivus:5.653805,Opheodrys_vernalis:5.654108)100:6.426115,(((Chironius_fuscus_KU214847:3.856101,Chironius_fuscus_LSUMNS13875:3.65048)100:3.803452,(Chironius_laevicollis:4.991323,(Chironius_scurrulus_LSUMNS9368:4.183067,(Chironius_scurrulus:4.180927,(Chironius_scurrulus_LSUMNS8940:2.820791,Chironius_scurrulus_AMNH152263:2.821981)83:3.799353)87:3.61805)33:3.624089)99:3.629336)99:2.561634,(Chironius_grandisquamis:5.038756,(((Chironius_flavolineatus:4.349723,(Chironius_monticola_AMNH150692:3.019531,Chironius_quadricarinatus:4.348833)76:3.614315)88:3.626312,(Chironius_monticola:5.394009,(Chironius_bicarinatus:5.032452,(Chironius_fuscus:5.031608,(Chironius_exoletus_AMNH152259:3.025118,Chironius_exoletus:4.213291)88:3.619388)97:2.801089)30:2.798955)98:3.623979)94:2.556661,((Chironius_carinatus:5.773742,(Chironius_multiventris_YPM016079:3.185448,Chironius_multiventris_YPM016080:2.821839)100:3.81155)99:3.623066,((Chironius_laurenti:5.695505,Chironius_multiventris_LSUMNS12556:2.9958)85:3.618583,((Chironius_multiventris:5.610363,Chironius_foveatus:4.863996)0:6.133897,(Chironius_exoletus_LSUMNS16518:4.143998,Chironius_multiventris_LSUMNS17665:2.821578)100:3.791493)89:2.801814)100:2.567695)100:2.56349)93:2.553861)81:2.554288)100:2.566233)66:2.557221)97:3.616736)94:3.849507,((Drymarchon_corais:5.654621,Drymarchon_couperi:6.272733)100:6.077418,((((Conopsis_biserialis:6.39248,Conophis_vittatus:6.391159)100:5.587507,(Pseudoficimia_frontalis:5.595117,Sympholis_lippiens:5.595791)94:5.573924)100:5.971082,(((Tantilla_relicta:5.632244,Tantilla_coronata:5.63647)100:5.586032,((Tantilla_gracilis:5.631462,Tantilla_hobartsmithi:5.629941)99:5.564542,(Tantilla_nigriceps:5.643308,Tantilla_planiceps:5.638488)74:5.553237)96:5.566083)100:6.041829,(Stenorrhina_freminvillei:5.814299,((Chionactis_occipitalis:5.796583,(Sonora_semiannulata:2.906277,Sonora_aemula:4.526669)54:2.858286)97:2.886243,(Chilomeniscus_stramineus:6.618842,(Sonora_michoacanensis:4.58504,Sonora_mutabilis:4.593991)100:4.495873)47:4.605467)96:3.021878)97:2.891693)17:2.856959)97:3.465102,((Tantilla_melanocephala:5.115123,((Coluber_schotti:5.844828,Coluber_taeniatus:5.649658)99:6.290813,(Coluber_lateralis:6.448914,(Coluber_flagellum:5.800868,(Coluber_bilineatus:6.027847,Coluber_constrictor:2.903888)60:2.855543)75:2.868948)6:2.855707)81:2.859915)95:1.940501,(((Rhinobothryum_lentiginosum:6.903961,(Salvadora_mexicana:6.587208,Salvadora_grahamiae:6.437837)89:6.970898)16:7.127212,((Drymoluber_dichrous:5.612189,Drymoluber_brazili:5.976525)96:5.586466,(Mastigodryas_bifossatus:6.863577,(Mastigodryas_melanolomus:7.095566,Mastigodryas_boddaerti:6.40255)0:6.465227)97:7.123463)100:5.585737)88:5.971709,(((Phyllorhynchus_decurtatus:2.25506,Phyllorhynchus_browni:6.199294)100:2.455203,(Salvadora_hexalepis:6.187928,Trimorphodon_biscutatus:2.912702)0:3.53264)97:1.312826,((Spilotes_pullatus:4.872831,Spilotes_pullatus_ADM259:5.053512)100:5.039159,((Spilotes_sulphureus:5.252427,(Phrynonax_shropshirei_LSUMNS7806:5.59652,(Spilotes_sulphureus_LSUMNS14023:4.770156,Spilotes_sulphureus_LSUMNS8937:4.771115)98:4.741018)98:4.927724)100:4.973041,((Phrynonax_poecilonotus:5.462866,Phrynonax_poecilonotus_ADM0005:3.651333)95:3.622789,(Phrynonax_shropshirei_LSUMNS7085:5.35032,(Phrynonax_polylepis:6.27915,Phrynonax_poecilonotus_AMNH152285:3.659043)0:3.623136)0:3.616337)100:3.830741)57:3.622041)92:3.836293)31:0.03033)22:0.01251)50:0.008814)31:0.001459)100:0.009635)98:0.004403)99:0.005709)95:0.002607)97:0.00306)100:0.021425)95:0.005331)99:0.01161)100:0.01817)100:0.029122)100:0.062638)98:0.027708)100:0.044186)100:0.006997)100:0.0554)100:0.247644)27:0.020352)100:0.50047);
