# Supplementary material for: A Species-Level Phylogeny of Extant Snakes with Description of a New Colubrid Subfamily and Genus
Source: PLoS One. 2016 Sep 7;11(9):e0161070. doi: 10.1371/journal.pone.0161070 (PMC5014348; doi:10.1371/journal.pone.0161070)
Supplement: S1 Table — Two sequences were deleted during preliminary tree searches and 21 were identified as rogue taxa and pruned from the dataset leaving 1592 snake species from GenBank in the tree. Names represent species names as listed on The Reptile Database (http://www.reptile-database.org/) as of October 2015. Refer to S4 Table for list of rogue taxa. Taxa deleted during preliminary tree searches are highlighted in red, rogue taxa are highlighted in yellow, and sequences that were deleted because they were identical to other sequences are highlighted in green. (DOCX) [file pone.0161070.s004.docx]

**S1 Table. List of GenBank accession numbers for 7 outgroup taxa and 1615 snake species**. Two sequences were deleted during preliminary tree searches and 21 were identified as rogue taxa and pruned from the dataset leaving 1592 snake species from GenBank in the tree. Names represent species names as listed on The Reptile Database (<http://www.reptile-database.org/>) as of October 2015. Refer to S4 Table for list of rogue taxa. Taxa deleted during preliminary tree searches are highlighted in red, rogue taxa are highlighted in yellow, and sequences that were deleted because they were identical to other sequences are highlighted in green.

| **Name** | **12S** | **16S** | **BDNF** | **CMOS** | **CYTB** | **ND2** | **ND4** | **NT3** | **RAG1F** | **RAG1M** | **RAG1E** | **RAG2** |
| --- | --- | --- | --- | --- | --- | --- | --- | --- | --- | --- | --- | --- |
| **OUTGROUPS** |  |  |  |  |  |  |  |  |  |  |  |  |
| *Calotes versicolor* | -- | JX668217.1 | DQ340705.1 | JX838985.1 | AY572870.1 | -- | JX857560.1 | JX839246.1 | -- | -- | -- | -- |
| *Chamaeleo calyptratus* | -- | HF570443.1 | GU457847.1 | HF570667.1 | NC_012420 | -- | HF570566.1 | GU456003.1 | HF570766.1 | -- | -- | -- |
| *Elgaria multicarinata* | AY649110.1 | AY649151.1 | GU457854.1 | AF039479.1 | AF361528.1 | -- | DQ364660.1 | GU456010.1 | -- | GU457977.1 | -- | -- |
| *Heloderma suspectum* | -- | NC_008776.1 | GU457856.1 | AY487348.1 | NC_008776.1 | -- | NC_008776.1 | GU456012.1 | -- | GU457979.1 | -- | DQ119635.1 |
| *Liolaemus darwinii* | JF272804.1 | -- | KP820851.1 | JF272870.1 | KC150152.1 | -- | DQ237768.1 | KP820633.1 | -- | KP820640.1 | -- | -- |
| *Plica plica* | EF615595.1 | EF615664.1 | JF806028.1 | EF615737.1 | EF616028.1 | -- | EF616320.1 | JF804573.1 | -- | JF806213.1 |  | -- |
| *Varanus salvator* | EU621802.1 | EU621807.1 | EU402618.1 | AF435017.1 | EU621812.1 | EU747731.1 | AY033776.1 | EU390902.1 | -- | EU402828.1 | -- | -- |
| **SNAKES** |  |  |  |  |  |  |  |  |  |  |  |  |
| *Acanthophis antarcticus* | -- | -- | -- | -- | AY340133.1 | -- | AY340162.1 | -- | -- | -- | -- | -- |
| *Acanthophis laevis* | -- | -- | -- | -- | AY340138.1 | -- | AY340167.1 | -- | -- | -- | -- | -- |
| *Acanthophis praelongus* | EU547112.1 | EU547161.1 | -- | EU546926.1 | EU547063.1 | -- | AY340164.1 | -- | EU546887.1 | -- | -- | -- |
| *Acanthophis pyrrhus* | -- | -- | -- | -- | AY340139.1 | -- | AY340168.1 | -- | -- | -- | -- | -- |
| *Acanthophis rugosus* | -- | -- | -- | -- | AY340130.1 | -- | AY340159.1 | -- | -- | -- | -- | -- |
| *Acanthophis wellsi* | -- | -- | -- | -- | AY340140.1 | -- | AY340169.1 | -- | -- | -- | -- | -- |
| *Achalinus meiguensis* | FJ424614 | FJ424614.1 | -- | -- | FJ424614 | NC_011576.1 | FJ424614 | -- | -- | -- | -- | -- |
| *Achalinus rufescens* | -- | -- | -- | -- | -- | -- | U49319.1 | -- | -- | -- | -- | -- |
| *Acrantophis dumerili* | EU403569.1 | EU419794.1 | AY988032.1 | EU403581.1 | EU403574.1 | -- | -- | -- | -- | AY988066.1 | -- | -- |
| *Acrantophis madagascariensis* | EU403566.1 | AY336071.1 | FJ433973.1 | EU403578.1 | U69736.1 | -- | -- | -- | -- | AY487401.1 | AY487401.1 | FJ433903.1 |
| *Acrochordus arafurae* | -- | -- | -- | HM234059.1 | -- | -- | HM234056.1 | -- | -- | HM234062.1 | -- | -- |
| *Acrochordus granulatus* | AF544738.1 | AB177879.1 | FJ433981.1 | HM234057.1 | AB177879 | AB177879.1 | AB177879 | FJ434082.1 | -- | -- | -- | EF144093.1 |
| *Acrochordus javanicus* | AF512745.1 | AF512745.1 | AY988036.1 | HM234058.1 | -- | -- | HM234055.1 | -- | -- | HM234061.1 | -- | -- |
| *Acutotyphlops kunuaensis* | -- | -- | GU902419.1 | -- | -- | -- | -- | GU902590.1 | -- | -- | GU902669.1 | -- |
| *Acutotyphlops* sp. | -- | -- | GU902459.1 | -- | -- | -- | -- | GU902629.1 | -- | -- | GU902704.1 | -- |
| *Acutotyphlops subocularis* | KF993037.1 | KF993127.1 | GU902418.1 | -- | JQ910524.1 | -- | -- | GU902589.1 | -- | -- | GU902668.1 | -- |
| *Adelophis foxi* | -- | -- | KF258601.1 | -- | KF258652.1 | AF420071.1 | KF258635.1 | KF234031.1 | -- | -- | -- | -- |
| *Adelphicos quadrivirgatum* | -- | -- | -- | GQ895796.1 | GQ895853.1 | -- | -- | -- | -- | -- | -- | -- |
| *Afronatrix anoscopus* | -- | -- | EU402622.1 | AF471123.1 | -- | AF420075.1 | AF420076.1 | EU390906.1 | -- | EU402832.1 | -- | -- |
| *Afrotyphlops angolensis* | -- | -- | GU902389.1 | -- | -- | -- | -- | GU902562.1 | -- | -- | GU902639.1 | -- |
| *Afrotyphlops bibronii* | -- | -- | GU902450.1 | -- | -- | -- | -- | GU902620.1 | -- | -- | GU902696.1 | -- |
| *Afrotyphlops congestus* | -- | -- | GU902448.1 | -- | -- | -- | -- | GU902618.1 | -- | -- | GU902694.1 | -- |
| *Afrotyphlops elegans* | -- | -- | KF992882.1 | -- | -- | -- | -- | GU902564.1 | -- | -- | KF992945.1 | -- |
| *Afrotyphlops fornasinii* | -- | -- | GU902447.1 | -- | -- | -- | -- | GU902617.1 | -- | -- | GU902693.1 | -- |
| *Afrotyphlops lineolatus* | -- | -- | GU902451.1 | -- | -- | -- | -- | GU902621.1 | -- | -- | GU902697.1 | -- |
| *Afrotyphlops mucruso* | -- | -- | GU902387.1 | -- | -- | -- | -- | GU902560.1 | -- | -- | GU902637.1 | -- |
| *Afrotyphlops obtusus* | -- | -- | -- | -- | -- | -- | -- | GU902625.1 | -- | -- | GU902700.1 | -- |
| *Afrotyphlops punctatus* | HQ113893.1 | -- | GU902395.1 | -- | -- | HQ113933.1 | -- | GU902567.1 | -- | -- | GU902645.1 | -- |
| *Afrotyphlops schlegelii* | -- | -- | GU902449.1 | -- | -- | -- | -- | GU902619.1 | -- | -- | GU902695.1 | -- |
| *Afrotyphlops* sp. 1 | -- | -- | KF992881.1 | -- | -- | -- | -- | KF992923.1 | -- | -- | KF992944.1 | -- |
| *Afrotyphlops* sp. 2 | -- | -- | KF992879.1 | -- | -- | -- | -- | KF992921.1 | -- | -- | KF992942.1 | -- |
| *Afrotyphlops* sp. 3 | -- | -- | KF992880.1 | -- | -- | -- | -- | KF992922.1 | -- | -- | KF992943.1 | -- |
| *Agkistrodon bilineatus* | AF057230.1 | [AF057277.1](http://www.ncbi.nlm.nih.gov/nucleotide/5771345?report=genbank&log$=nucltop&blast_rank=1&RID=MS4NXXZN01R) | -- | -- | EU483408.1 | -- | AF156583.1 | -- | -- | -- | -- | -- |
| *Agkistrodon contortrix* | AF259224.1 | AF156566.1 | EU402623.1 | -- | EU483383.1 | -- | AF156577.1 | JN703027.1 | -- | EU402833.1 | -- | -- |
| *Agkistrodon howardgloydi* | AF156593.1 | AF156572.1 | -- | -- | -- | -- | AF156585.1 | -- | -- | -- | -- | -- |
| *Agkistrodon piscivorus* | AF259225.1 | AF057278.1 | JQ599004.1 | AF471096.1 | EU483436.1 | NC_009768.1 | AF156578.1 | -- | -- | -- | -- | -- |
| *Agkistrodon russeolus* | L01766.1 | AF156573.1 | -- | -- | -- | -- | AF156586.1 | -- | -- | -- | -- | -- |
| *Agkistrodon taylori* | AF156589.1 | AF156568.1 | -- | -- | EU483477.1 | -- | AF156581.1 | -- | -- | -- | -- | -- |
| *Ahaetulla fronticincta* | -- | -- | -- | AF471161.1 | AF471072.1 | -- | -- | -- | -- | -- | -- | -- |
| *Ahaetulla nasuta* | -- | -- | -- | KC347377.1 | KC347453.1 | -- | KC347526.1 | -- | -- | KC347415.1 | -- | -- |
| *Ahaetulla prasina* | -- | -- | -- | KC010300.1 | KC010339.1 | -- | -- | -- | -- | -- | -- | -- |
| *Ahaetulla pulverulenta* | KC347304.1 | KC347339.1 | -- | KC347378.1 | KC347454.1 | -- | KC347512.1 | -- | -- | KC347416.1 | -- | -- |
| *Aipysurus apraefrontalis* | -- | JX423420.1 | -- | KC014288.1 | KC014380.1 | -- | JX002981.1 | -- | KC014257.1 | -- | -- | -- |
| *Aipysurus duboisii* | -- | JX423423.1 | -- | FJ587163.1 | JX423393.1 | -- | JX423411.1 | -- | FJ587085.1 | -- | -- | -- |
| *Aipysurus eydouxii* | -- | DQ233986.1 | -- | FJ587166.1 | JX423397.1 | -- | JX423414.1 | -- | FJ587092.1 | -- | -- | -- |
| *Aipysurus foliosquama* | -- | -- | -- | -- | KP205508.1 | -- | -- | -- | -- | -- | -- | -- |
| *Aipysurus fuscus* | -- | DQ233988.1 | -- | FJ587168.1 | DQ233911.1 | -- | EF506634.1 | -- | FJ587089.1 | -- | -- | -- |
| *Aipysurus laevis* | EU547132.1 | DQ233990.1 | -- | FJ587170.1 | DQ233914.1 | -- | EF506638.1 | -- | FJ587088.1 | -- | -- | -- |
| *Aipysurus mosaicus* | -- | JX423428.1 | -- | -- | JX423399.1 | -- | JX423417.1 | -- | -- | -- | -- | -- |
| *Alluaudina bellyi* | FJ404142.1 | AY188044.1 | -- | AY187966.1 | -- | -- | FJ404345.1 | -- | -- | -- | -- | FJ404417.1 |
| *Alsophis antiguae* | AF158455.1 | AF158524.1 | -- | -- | -- | -- | -- | -- | -- | -- | -- | -- |
| *Alsophis antillensis* | AF158459.1 | FJ416702.1 | JQ599005.1 | -- | FJ416726.1 | FJ416764.1 | FJ416800.1 | -- | -- | -- | -- | FJ416837.1 |
| *Alsophis manselli* | -- | -- | -- | -- | FJ416727.1 | FJ416765.1 | FJ416801.1 | -- | -- | -- | -- | FJ416838.1 |
| *Alsophis rijgersmaei* | FJ416697.1 | FJ416708.1 | -- | -- | FJ416729.1 | FJ416767.1 | FJ416803.1 | -- | -- | -- | -- | FJ416840.1 |
| *Alsophis rufiventris* | FJ416698.1 | FJ416709.1 | -- | -- | FJ416730.1 | FJ416768.1 | FJ416804.1 | -- | -- | -- | -- | FJ416841.1 |
| *Alsophis sajdaki* | -- | -- | -- | -- | FJ416731.1 | FJ416769.1 | FJ416805.1 | -- | -- | -- | -- | FJ416842.1 |
| *Alsophis sibonius* | -- | -- | -- | -- | FJ416728.1 | FJ416766.1 | FJ416802.1 | -- | -- | -- | -- | FJ416839.1 |
| *Amastridium veliferum* | -- | -- | -- | GQ895797.1 | GQ334479.1 | -- | GQ334580.1 | GQ334663.1 | -- | -- | -- | -- |
| *Amblyodipsas dimidiata* | -- | -- | -- | DQ486170.1 | DQ486346.1 | -- | -- | -- | -- | -- | -- | -- |
| *Amblyodipsas polylepis* | FJ404128.1 | AY611844.1 | -- | AY611936.1 | AY612027.1 | -- | -- | -- | -- | -- | -- | FJ404403.1 |
| *Amerotyphlops brongersmianus* | KF993138.1 | -- | GU902390.1 | -- | KF993239.1 | -- | -- | GU902563.1 | -- | -- | GU902640.1 | -- |
| *Amerotyphlops reticulatus* | EU747730.1 | EU747730 | GU902396.1 | -- | EU747730 | EU747730.1 | EU747730 | GU902568.1 | -- | -- | GU902646.1 | -- |
| *Amerotyphlops tasymicris* | -- | -- | -- | -- | KF993283.1 | -- | -- | -- | -- | -- | -- | -- |
| *Amphiesma stolatum* | -- | -- | EU402624.1 | KJ685661.1 | JQ687432.1 | JQ687464.1 | JQ687425.1 | KJ685746.1 | KJ685610.1 | -- | -- | -- |
| *Amplorhinus multimaculatus* | FJ404143.1 | AY611880.1 | -- | DQ486164.1 | DQ486340.1 | -- | DQ486316.1 | -- | -- | -- | -- | FJ404418.1 |
| *Anilios affinis* | KF992951.1 | -- | -- | -- | JQ910526.1 | -- | -- | -- | -- | -- | -- | -- |
| *Anilios ammodytes* | KF992958.1 | KF993040.1 | JQ910309.1 | -- | KC489804.1 | -- | -- | -- | -- | -- | -- | -- |
| *Anilios australis* | AY442895.1 | AY442843.1 | JQ910312.1 | AF039474.1 | JQ910537.1 | DQ902231.1 | -- | GU902580.1 | -- | -- | GU902659.1 | -- |
| *Anilios bicolor* | AY442900.1 | AY442836.1 | JQ910401.1 | -- | JQ910632.1 | -- | -- | GU902581.1 | -- | -- | GU902660.1 | -- |
| *Anilios bituberculatus* | AY442893.1 | AY442831.1 | JQ910313.1 | -- | JQ910538.1 | -- | -- | GU902574.1 | -- | -- | GU902653.1 | -- |
| *Anilios centralis* | KF992971.1 | KF993058.1 | JQ910403.1 | -- | KC490114.1 | -- | -- | -- | -- | -- | -- | -- |
| *Anilios diversus* | KF992974.1 | KF993060.1 | JQ910323.1 | -- | KC490117.1 | -- | -- | GU902582.1 | -- | -- | GU902661.1 | -- |
| *Anilios endoterus* | KF992983.1 | KF993069.1 | JQ910328.1 | -- | KC490138.1 | -- | -- | GU902570.1 | -- | -- | GU902649.1 | -- |
| *Anilios ganei* | KF992986.1 | KF993073.1 | JQ910404.1 | -- | KC490220.1 | -- | -- | GU902583.1 | -- | -- | GU902662.1 | -- |
| *Anilios grypus* | AY442898.1 | AY442835.1 | JQ910333.1 | -- | JQ910564.1 | -- | -- | GU902584.1 | -- | -- | GU902663.1 | -- |
| *Anilios guentheri* | KF992998.1 | KF993083.1 | JQ910348.1 | -- | JQ910579.1 | -- | -- | GU902575.1 | -- | -- | GU902654.1 | -- |
| *Anilios hamatus* | AY442894.1 | AY442832.1 | JQ910355.1 | -- | JQ910586.1 | -- | -- | GU902572.1 | -- | -- | GU902651.1 | -- |
| *Anilios howi* | KF993009.1 | KF993096.1 | GU902414.1 | -- | JQ910587.1 | -- | -- | GU902585.1 | -- | -- | GU902664.1 | -- |
| *Anilios kimberleyensis* | KF993011.1 | KF993097.1 | JQ910358.1 | -- | KC490333.1 | -- | -- | GU902577.1 | -- | -- | GU902656.1 | -- |
| *Anilios leptosomus* | KF993015.1 | AY442830.1 | JQ910363.1 | -- | JQ910594.1 | -- | -- | -- | -- | -- | -- | -- |
| *Anilios ligatus* | KF993016.1 | KF993103.1 | JQ910368.1 | -- | KC490346.1 | -- | -- | GU902576.1 | -- | -- | GU902655.1 | -- |
| *Anilios longissimus* | AY442901.1 | AY442838.1 | JQ910301.1 | -- | JQ910525.1 | -- | -- | GU902579.1 | -- | -- | GU902658.1 | -- |
| *Anilios nigrescens* | KF993021.1 | KF993108.1 | JQ910372.1 | -- | KC490354.1 | -- | -- | -- | -- | -- | -- | -- |
| *Anilios pilbarensis* | AY442896.1 | AY442834.1 | JQ910397.1 | -- | JQ910628.1 | -- | -- | GU902571.1 | -- | -- | GU902650.1 | -- |
| *Anilios pinguis* | -- | KF993114.1 | JQ910402.1 | -- | JQ910627.1 | -- | -- | GU902586.1 | -- | -- | GU902665.1 | -- |
| *Anilios polygrammicus* | KF993025.1 | KF993115.1 | JQ910381.1 | -- | KC490391.1 | -- | -- | GU902591.1 | -- | -- | GU902671.1 | -- |
| *Anilios proximus* | KF993027.1 | KF993117.1 | JQ910398.1 | -- | KC490394.1 | -- | -- | -- | -- | -- | -- | -- |
| *Anilios silvia* | KF993028.1 | KF993118.1 | JQ910382.1 | -- | KC493653.1 | -- | -- | -- | -- | -- | -- | -- |
| *Anilios splendidus* | -- | -- | GU902416.1 | -- | -- | -- | -- | GU902587.1 | -- | -- | GU902666.1 | -- |
| *Anilios troglodytes* | KF993029.1 | KF993119.1 | JQ910383.1 | -- | JQ910614.1 | -- | -- | GU902588.1 | -- | -- | GU902667.1 | -- |
| *Anilios unguirostris* | KF993033.1 | AY442837.1 | JQ910387.1 | -- | JQ910618.1 | -- | -- | GU902578.1 | -- | -- | GU902657.1 | -- |
| *Anilios waitii* | KF993035.1 | AY442840.1 | JQ910405.1 | -- | JQ910636.1 | -- | -- | GU902573.1 | -- | -- | GU902652.1 | -- |
| *Anilios wiedii* | KF993036.1 | KF993126.1 | JQ910400.1 | -- | JQ910631.1 | -- | -- | -- | -- | -- | -- | -- |
| *Anilius scytale* | FJ755180 | FJ755180 | EU402625.1 | AF544722.1 | U69738.1 | NC_014343.1 | FJ755180 | FJ434066.1 | -- | AY988072.1 | -- | FJ433891.1 |
| *Anomochilus leonardi* | AY953430.1 | AY953431.1 | -- | -- | -- | -- | -- | -- | -- | -- | -- | -- |
| *Antaresia childreni* | EF545031.1 | EF545058.1 | -- | AY099967.1 | U69837.1 | -- | -- | -- | -- | -- | -- | -- |
| *Antaresia maculosa* | EF545029.1 | EF545056.1 | -- | -- | U69838.1 | -- | -- | -- | -- | -- | -- | -- |
| *Antaresia perthensis* | EF545032.1 | EF545059.1 | -- | -- | EF545104.1 | -- | -- | -- | -- | -- | -- | -- |
| *Antaresia stimsoni* | EF545030.1 | EF545057.1 | -- | -- | EF545103.1 | -- | -- | -- | -- | -- | -- | -- |
| *Aparallactus capensis* | FJ404129.1 | AY188045.1 | -- | AY187967.1 | AY188006.1 | -- | FJ404331.1 | -- | -- | -- | -- | FJ404404.1 |
| *Aparallactus guentheri* | -- | -- | -- | -- | AY235730.1 | -- | -- | -- | -- | -- | -- | -- |
| *Aparallactus modestus* | FJ404130.1 | AY611824.1 | -- | AY611916.1 | AY612007.1 | -- | FJ404332.1 | -- | -- | -- | -- | FJ404405.1 |
| *Aparallactus werneri* | -- | -- | EU402626.1 | AF471116.1 | AF471035.1 | -- | U49315.1 | EU390907.1 | -- | -- | -- | -- |
| *Aplopeltura boa* | AF544761.1 | AF544787.1 | FJ433984.1 | JF827696.1 | JF827673.1 | -- | JF827650.1 | FJ434085.1 | -- | -- | -- | -- |
| *Apostolepis albicollaris* | JQ598793.1 | JQ598856.1 | -- | JQ598965.1 | -- | -- | -- | -- | -- | -- | -- | -- |
| *Apostolepis assimilis* | GQ457781.1 | GQ457724.1 | JQ599007.1 | GQ457843.1 | -- | -- | -- | -- | -- | -- | -- | -- |
| *Apostolepis cearensis* | JQ598794.1 | JQ598857.1 | -- | JQ598966.1 | -- | -- | -- | -- | -- | -- | -- | -- |
| *Apostolepis cearensis* (*sanctaeritae*) | JQ598797.1 | JQ598859.1 | -- | JQ598969.1 | -- | -- | -- | -- | -- | -- | -- | -- |
| *Apostolepis dimidiata* | GQ457782.1 | GQ457725.1 | JQ599008.1 | GQ457844.1 | JQ598917.1 | -- | -- | -- | -- | -- | -- | -- |
| *Apostolepis flavotorquata* | JQ598795.1 | JQ598858.1 | -- | GQ895798.1 | GQ895854.1 | -- | -- | -- | -- | -- | -- | -- |
| *Apostolepis nigrolineata* | JQ598796.1 | -- | -- | JQ598968.1 | -- | -- | -- | -- | -- | -- | -- | -- |
| *Archelaphe bella* | -- | -- | -- | DQ902097.1 | DQ902134.1 | DQ902248.1 | DQ902316.1 | -- | -- | -- | -- | -- |
| *Argyrophis diardii* | -- | -- | KF992877.1 | -- | -- | -- | -- | KF992919.1 | -- | -- | KF992940.1 | -- |
| *Argyrophis muelleri* | -- | -- | KF992878.1 | -- | -- | -- | -- | KF992920.1 | -- | -- | -- | -- |
| *Argyrophis* sp. 1 | -- | -- | KF992870.1 | -- | -- | -- | -- | KF992912.1 | -- | -- | KF992933.1 | -- |
| *Argyrophis* sp. 2 | -- | -- | KF992871.1 | -- | -- | -- | -- | KF992913.1 | -- | -- | KF992934.1 | -- |
| *Argyrophis* sp. 3 | -- | -- | KF992872.1 | -- | -- | -- | -- | KF992914.1 | -- | -- | KF992935.1 | -- |
| *Argyrophis* sp. 4 | -- | -- | KF992873.1 | -- | -- | -- | -- | KF992915.1 | -- | -- | KF992936.1 | -- |
| *Argyrophis* sp. 5 | -- | -- | KF992874.1 | -- | -- | -- | -- | KF992916.1 | -- | -- | KF992937.1 | -- |
| *Arizona elegans* | AY122810.1 | -- | -- | DQ902058.1 | DQ902101.1 | DQ902204.1 | DQ902279.1 | KF215002.1 | -- | -- | -- | -- |
| *Arrhyton dolichura* | AF158438.1 | AF158507.1 | -- | -- | FJ416721.1 | FJ416759.1 | FJ416795.1 | -- | -- | -- | -- | FJ416832.1 |
| *Arrhyton procerum* | AF158452.1 | AF158521.1 | -- | -- | FJ416723.1 | FJ416761.1 | FJ416797.1 | -- | -- | -- | -- | FJ416834.1 |
| *Arrhyton redimitum* | AF158439.1 | AF158508.1 | -- | -- | FJ416720.1 | FJ416758.1 | FJ416794.1 | -- | -- | -- | -- | FJ416831.1 |
| *Arrhyton supernum* | AF158436.1 | AF158505.1 | -- | -- | FJ416718.1 | FJ416756.1 | FJ416792.1 | -- | -- | -- | -- | FJ416829.1 |
| *Arrhyton taeniatum* | AF158453.1 | AF158522.1 | -- | -- | FJ416717.1 | FJ416755.1 | FJ416791.1 | -- | -- | -- | -- | FJ416828.1 |
| *Arrhyton tanyplectum* | AF158446.1 | AF158516.1 | -- | -- | FJ416722.1 | FJ416760.1 | FJ416796.1 | -- | -- | -- | -- | FJ416833.1 |
| *Arrhyton vittatum* | AF158437.1 | AF158506.1 | -- | -- | FJ416719.1 | FJ416757.1 | FJ416793.1 | -- | -- | -- | -- | FJ416830.1 |
| *Aspidelaps lubricus* | -- | -- | -- | -- | FR693724.1 | -- | -- | -- | -- | -- | -- | -- |
| *Aspidelaps scutatus* | U96790.1 | AY188046.1 | -- | AY187968.1 | -- | AY058999.1 | AY058969.1 | -- | -- | -- | -- | -- |
| *Aspidites melanocephalus* | EF545033.1 | EF545060.1 | EU402627.1 | DQ465557.1 | U69741.1 | -- | -- | DQ465558.1 | -- | DQ465560.1 | -- | -- |
| *Aspidites ramsayi* | EF545034.1 | EF545061.1 | KF811057.1 | KF811094.1 | EF545105.1 | -- | -- | -- | -- | KF811158.1 | -- | -- |
| *Aspidomorphus lineaticollis* | GQ397245.1 | GQ397237.1 | -- | GQ397227.1 | -- | -- | -- | -- | -- | -- | -- | -- |
| *Aspidomorphus muelleri* | GQ397251.1 | GQ397242.1 | -- | GQ397232.1 | -- | -- | -- | -- | -- | -- | -- | -- |
| *Aspidomorphus schlegeli* | GQ397246.1 | GQ397238.1 | -- | GQ397228.1 | -- | -- | -- | -- | -- | -- | -- | -- |
| *Aspidura ceylonensis* | KC347324.1 | KC347361.1 | -- | KC347400.1 | KC347477.1 | -- | KC347527.1 | -- | -- | KC347438.1 | -- | -- |
| *Aspidura drummondhayi* | KC347305.1 | KC347340.1 | -- | KC347379.1 | KC347455.1 | -- | KC347519.1 | -- | -- | KC347417.1 | -- | -- |
| *Aspidura guentheri* | KC347306.1 | KC347341.1 | -- | KC347380.1 | KC347472.1 | -- | KC347507.1 | -- | -- | KC347418.1 | -- | -- |
| *Aspidura* sp. | KC347307.1 | KC347342.1 | -- | KC347381.1 | KC347456.1 | -- | KC347514.1 | -- | -- | KC347419.1 | -- | -- |
| *Aspidura trachyprocta* | KC347308.1 | KC347343.1 | -- | KC347382.1 | KC347473.1 | -- | KC347523.1 | -- | -- | KC347420.1 | -- | -- |
| *Asthenodipsas laevis* | -- | -- | -- | -- | KC916749.1 | -- | -- | -- | -- | -- | -- | -- |
| *Asthenodipsas lasgalenensis* | -- | -- | -- | -- | KC916763.1 | -- | -- | -- | -- | -- | -- | -- |
| *Asthenodipsas vertebralis* | -- | -- | -- | -- | KC916750.1 | -- | -- | -- | -- | -- | -- | -- |
| *Atheris barbouri* | -- | AJ275739.1 | -- | -- | AJ275686.1 | -- | -- | -- | -- | -- | -- | -- |
| *Atheris ceratophora* | DQ305410.1 | DQ305433.1 | -- | -- | JF825388.1 | -- | DQ305474.1 | -- | -- | -- | -- | -- |
| *Atheris chlorechis* | EU624244.1 | EU624278.1 | -- | -- | AJ275679.1 | -- | EU624211.1 | -- | -- | -- | -- | -- |
| *Atheris desaixi* | -- | AJ275733.1 | -- | -- | AJ275680.1 | -- | -- | -- | -- | -- | -- | -- |
| *Atheris hispida* | -- | AJ275734.1 | -- | -- | AJ275681.1 | -- | -- | -- | -- | -- | -- | -- |
| *Atheris matildae* | -- | -- | -- | -- | JF825389.1 | -- | -- | -- | -- | -- | -- | -- |
| *Atheris nitschei* | AY223650.1 | AY223663.1 | -- | AF471125.1 | AF471070.1 | -- | AY223618.1 | -- | -- | -- | -- | -- |
| *Atheris squamigera* | AF544762.1 | AF544788.1 | -- | AF544734.1 | AJ275684.1 | -- | EU624212.1 | -- | -- | -- | -- | -- |
| *Atractaspis bibronii* | FJ404131.1 | AY611840.1 | -- | AY611932.1 | AY612023.1 | -- | U49314.1 | -- | -- | -- | -- | FJ404406.1 |
| *Atractaspis boulengeri* | FJ404132.1 | AY611833.1 | -- | AY611925.1 | AY612016.1 | -- | FJ404334.1 | -- | -- | -- | -- | FJ404407.1 |
| *Atractaspis corpulenta* | Z46597.1 | AY611837.1 | -- | AY611929.1 | AY612020.1 | -- | FJ404335.1 | -- | -- | -- | DQ993174.1 | FJ404408.1 |
| *Atractaspis irregularis* | -- | -- | JN614212.1 | -- | -- | -- | -- | -- | -- | -- | -- | -- |
| *Atractaspis microlepidota* | -- | -- | -- | AF471127.1 | AF471046.1 | -- | -- | -- | -- | -- | -- | -- |
| *Atractaspis micropholis* | AF544740.1 | AY611823.1 | FJ433994.1 | AY611915.1 | AY612006.1 | -- | FJ404336.1 | FJ434095.1 | -- | -- | -- | EF144105.1 |
| *Atractus albuquerquei* | GQ457783.1 | GQ457726.1 | JQ599009.1 | GQ457845.1 | JQ598918.1 | -- | -- | -- | -- | -- | -- | -- |
| *Atractus badius* | AF158425.1 | AF158485.1 | -- | -- | -- | -- | -- | -- | -- | -- | -- | -- |
| *Atractus elaps* | -- | -- | -- | -- | EF078536.1 | -- | EF078584.1 | GU353273.1 | -- | -- | -- | -- |
| *Atractus flammigerus* | AF158402.1 | AF158471.1 | -- | -- | -- | -- | -- | -- | -- | -- | -- | -- |
| *Atractus reticulatus* | JQ598798.1 | -- | -- | JQ598970.1 | -- | -- | -- | -- | -- | -- | -- | -- |
| *Atractus schach* | AF158427.1 | AF158486.1 | -- | JQ598971.1 | -- | -- | -- | -- | -- | -- | -- | -- |
| *Atractus trihedrurus* | GQ457784.1 | GQ457727.1 | JQ599010.1 | -- | JQ598919.1 | -- | -- | -- | -- | -- | -- | -- |
| *Atractus wagleri* | -- | -- | -- | -- | GQ334480.1 | -- | GQ334581.1 | GQ334664.1 | -- | -- | -- | -- |
| *Atractus zebrinus* | JQ598800.1 | JQ598861.1 | -- | JQ598972.1 | -- | -- | -- | -- | -- | -- | -- | -- |
| *Atractus zidoki* | AF158426.1 | AF158487.1 | -- | -- | -- | -- | -- | -- | -- | -- | -- | -- |
| *Atretium schistosum* | KC347309.1 | -- | -- | KC347383.1 | KC347487.1 | -- | KC347525.1 | -- | -- | KC347421.1 | -- | -- |
| *Atretium yunnanensis* | -- | -- | -- | JQ687448.1 | GQ281787.1 | JQ687463.1 | JQ687423.1 | KJ685764.1 | KJ685602.1 | -- | -- | -- |
| *Atropoides indomitus* | -- | -- | -- | -- | -- | -- | KC354700.1 | -- | -- | -- | -- | -- |
| *Atropoides mexicanus* | KC847268.1 | KC847255.1 | -- | -- | KC847271.1 | -- | KC847289.1 | -- | -- | -- | -- | -- |
| *Atropoides nummifer* | DQ305422.1 | DQ305445.1 | -- | -- | EU684273.1 | -- | DQ061220.1 | -- | -- | -- | -- | -- |
| *Atropoides occiduus* | DQ305423.1 | DQ305446.1 | -- | -- | DQ061194.1 | -- | DQ061219.1 | -- | -- | -- | -- | -- |
| *Atropoides olmec* | AY223656.1 | AY223669.1 | -- | -- | AY223585.1 | -- | AY223632.1 | -- | -- | -- | -- | -- |
| *Atropoides picadoi* | AF057208.1 | AF057255.1 | -- | -- | AY220324.1 | -- | AY220347.1 | -- | -- | -- | -- | -- |
| *Austrelaps labialis* | EU547126.1 | EU547175.1 | -- | EU546939.1 | EU547077.1 | -- | EU547029.1 | -- | EU546900.1 | -- | -- | -- |
| *Austrelaps superbus* | EU547127.1 | EU547176.1 | -- | EU546940.1 | EU547078.1 | -- | EU547030.1 | -- | EU546901.1 | -- | -- | -- |
| *Azemiops feae* | AY352774.1 | AF057234.1 | EU402628.1 | AF544695.1 | AY352747.1 | -- | AY352808.1 | EU390909.1 | -- | EU402836.1 | -- | -- |
| *Balanophis ceylonensis* | KC347310.1 | KC347344.1 | -- | KC347384.1 | KC347474.1 | -- | KC347520.1 | -- | -- | KC347422.1 | -- | -- |
| *Bamanophis dorri* | -- | AY188081.1 | -- | AY188001.1 | AY188040.1 | AY487003.1 | AY487042.1 | -- | -- | -- | -- | -- |
| *Bitia hydroides* | EF395872.1 | EF395847.1 | -- | EF395921.1 | EF395896.1 | -- | -- | -- | -- | -- | -- | -- |
| *Bitis arietans* | GQ359655.1 | GQ359736.1 | -- | -- | GQ359487.1 | JX073288.1 | GQ359574.1 | -- | -- | EU852328.1 | -- | -- |
| *Bitis armata* | -- | -- | -- | -- | -- | JX073291.1 | -- | -- | -- | -- | -- | -- |
| *Bitis atropos* | EU624246.1 | EU624281.1 | -- | -- | AJ275691.1 | JX073287.1 | EU624214.1 | -- | -- | -- | -- | -- |
| *Bitis caudalis* | EU624247.1 | EU624282.1 | -- | -- | AJ275693.1 | JX073293.1 | EU624215.1 | -- | -- | -- | -- | -- |
| *Bitis cornuta* | EU624248.1 | EU624283.1 | -- | -- | AJ275694.1 | -- | EU624216.1 | -- | -- | -- | -- | -- |
| *Bitis gabonica* | EU624249.1 | EU624284.1 | -- | -- | AJ275695.1 | JX073296.1 | EU624217.1 | -- | -- | -- | -- | -- |
| *Bitis nasicornis* | DQ305411.1 | AY188048.1 | -- | AY187970.1 | DQ305457.1 | -- | DQ305475.1 | -- | -- | -- | -- | -- |
| *Bitis parviocula* | -- | -- | -- | -- | -- | JX073292.1 | -- | -- | -- | -- | -- | -- |
| *Bitis peringueyi* | DQ305412.1 | DQ305435.1 | -- | -- | DQ305458.1 | -- | DQ305476.1 | -- | -- | -- | -- | -- |
| *Bitis rhinoceros* | EU624250.1 | EU624285.1 | -- | -- | AJ275696.1 | -- | EU624218.1 | -- | -- | -- | -- | -- |
| *Bitis rubida* | EU624251.1 | EU624286.1 | -- | -- | -- | JX073290.1 | EU624219.1 | -- | -- | -- | -- | -- |
| *Bitis schneideri* | -- | -- | -- | -- | -- | JX073297.1 | -- | -- | -- | -- | -- | -- |
| *Bitis worthingtoni* | EU624252.1 | AJ275745.1 | -- | -- | AJ275692.1 | JX073295.1 | EU624220.1 | -- | -- | -- | -- | -- |
| *Bitis xeropaga* | EU624253.1 | EU624287.1 | -- | -- | -- | JX073294.1 | EU624221.1 | -- | -- | -- | -- | -- |
| *Boa constrictor* | AB177354 | AB177354 | KC330044.1 | AF544676.1 | AB177354 | NC_007398.1 | AB177354 | KC330109.1 | -- | KC330012.1 | -- | FJ433905.1 |
| *Boaedon capensis* | FJ404161.1 | AY611895.1 | -- | AY611986.1 | AY612077.1 | -- | FJ404362.1 | -- | -- | -- | -- | FJ404435.1 |
| *Boaedon fuliginosus* | FJ404165.1 | AY188079.1 | EU402646.1 | FJ404270.1 | AF471060.1 | -- | FJ404365.1 | EU390926.1 | -- | EU402849.1 | EU402849.1 | FJ404438.1 |
| *Boaedon lineatus* | FJ404168.1 | FJ404205.1 | -- | FJ387205.1 | FJ404303.1 | -- | -- | -- | -- | -- | -- | FJ404441.1 |
| *Boaedon olivaceus* | FJ404170.1 | AY611862.1 | -- | AY611953.1 | AY612044.1 | -- | KM519697.1 | -- | KM519731.1 | -- | -- | FJ404443.1 |
| *Boaedon radfordi* | -- | -- | -- | KM519749.1 | KM519715.1 | -- | KM519694.1 | -- | KM519729.1 | -- | -- | -- |
| *Boaedon upembae* | -- | -- | -- | KM519734.1 | KM519699.1 | -- | KM519680.1 | -- | KM519718.1 | -- | -- | -- |
| *Boaedon virgatus* | FJ404171.1 | AY611825.1 | -- | AY611917.1 | AY612008.1 | -- | FJ404369.1 | -- | -- | -- | -- | FJ404444.1 |
| *Bogertophis rosaliae* | AY122815.1 | -- | -- | DQ902059.1 | DQ902102.1 | DQ902205.1 | DQ902280.1 | -- | -- | -- | -- | -- |
| *Bogertophis subocularis* | AY122846.1 | -- | -- | DQ902060.1 | DQ902103.1 | DQ902206.1 | DQ902281.1 | -- | -- | -- | -- | -- |
| *Boiga barnesii* | KC347311.1 | KC347345.1 | -- | KC347385.1 | KC347469.1 | -- | KC347499.1 | -- | -- | KC347423.1 | -- | -- |
| *Boiga beddomei* | KC347312.1 | KC347346.1 | -- | KC347386.1 | KC347466.1 | -- | KC347495.1 | -- | -- | KC347424.1 | -- | -- |
| *Boiga ceylonensis* | KC347313.1 | KC347347.1 | -- | KC347387.1 | KC347467.1 | -- | KC347500.1 | -- | -- | KC347425.1 | -- | -- |
| *Boiga cynodon* | Z46468.1 | AF139566.1 | -- | KC010301.1 | KC010340.1 | -- | -- | -- | -- | -- | -- | -- |
| *Boiga dendrophila* | -- | -- | -- | AF471128.1 | AF471089.1 | -- | U49303.1 | -- | -- | -- | -- | -- |
| *Boiga forsteni* | KC347314.1 | KC347348.1 | -- | KC347388.1 | KC347468.1 | -- | KC347506.1 | -- | -- | KC347426.1 | -- | -- |
| *Boiga irregularis* | -- | AF139551.1 | -- | FJ710793.1 | FJ710794.1 | -- | -- | -- | -- | -- | -- | -- |
| *Boiga kraepelini* | -- | -- | -- | FJ710795.1 | FJ710796.1 | -- | -- | -- | -- | -- | -- | -- |
| *Boiga multomaculata* | -- | -- | -- | FJ710797.1 | FJ710798.1 | -- | -- | -- | -- | -- | -- | -- |
| *Boiga trigonata* | KC347315.1 | KC347349.1 | -- | KC347389.1 | KC347475.1 | -- | KC347515.1 | -- | -- | KC347427.1 | -- | -- |
| *Boiruna maculata* | GQ457785.1 | JQ598862.1 | JQ599011.1 | GQ895799.1 | GQ895855.1 | -- | -- | -- | -- | -- | -- | -- |
| *Borikenophis portoricensis* | FJ416696.1 | AF158517.1 | JQ599012.1 | AF471126.1 | AF471085.1 | FJ416770.1 | FJ416806.1 | -- | -- | -- | -- | FJ416844.1 |
| *Borikenophis variegatus* | -- | -- | -- | -- | FJ416734.1 | FJ416772.1 | FJ416808.1 | -- | -- | -- | -- | FJ416845.1 |
| *Bothriechis aurifer* | DQ305425.1 | DQ305448.1 | -- | -- | DQ305466.1 | -- | DQ305483.1 | -- | -- | -- | -- | -- |
| *Bothriechis bicolor* | DQ305426.1 | DQ305449.1 | -- | -- | DQ305467.1 | -- | DQ305484.1 | -- | -- | -- | -- | -- |
| *Bothriechis guifarroi* | KC847265.1 | KC847264.1 | -- | -- | KC847281.1 | -- | KC847287.1 | -- | -- | -- | -- | -- |
| *Bothriechis lateralis* | AF057211.1 | AF057258.1 | -- | -- | AY223588.1 | -- | U41873.1 | -- | -- | -- | -- | -- |
| *Bothriechis marchi* | DQ305428.1 | DQ305451.1 | -- | -- | DQ305469.1 | -- | DQ305486.1 | -- | -- | -- | -- | -- |
| *Bothriechis nigroviridis* | AF057212.1 | AF057259.1 | -- | -- | AY223589.1 | -- | AY223635.1 | -- | -- | -- | -- | -- |
| *Bothriechis rowleyi* | DQ305427.1 | DQ305450.1 | -- | -- | DQ305468.1 | -- | DQ305485.1 | -- | -- | -- | -- | -- |
| *Bothriechis schlegelii* | KC847270.1 | KC847257.1 | FJ433983.1 | -- | KC847272.1 | -- | KC847285.1 | FJ434084.1 | -- | -- | AY487374.1 | EF144095.1 |
| *Bothriechis supraciliaris* | DQ305429.1 | DQ305452.1 | -- | -- | DQ305470.1 | -- | DQ305487.1 | -- | -- | -- | -- | -- |
| *Bothriechis thalassinus* | DQ305424.1 | DQ305447.1 | -- | -- | DQ305465.1 | -- | DQ305482.1 | -- | -- | -- | -- | -- |
| *Bothrochilus albertisii* | EF545026.1 | EF545053.1 | KF811059.1 | KF811096.1 | U69835.1 | -- | -- | -- | -- | KF811160.1 | -- | -- |
| *Bothrochilus boa* | EF545028.1 | EF545055.1 | -- | -- | EF545101.1 | -- | -- | -- | -- | -- | -- | -- |
| *Bothrochilus meridionalis* | -- | -- | -- | -- | EU179542.1 | -- | -- | -- | -- | -- | -- | -- |
| *Bothrocophias campbelli* | -- | -- | -- | -- | AF191582.1 | -- | AF292622.1 | -- | -- | -- | -- | -- |
| *Bothrocophias hyoprora* | AF057206.1 | AF057253.1 | -- | -- | AY223593.1 | -- | FR691537.1 | -- | -- | -- | -- | -- |
| *Bothrocophias microphthalmus* | AY223657.1 | AY223670.1 | -- | -- | FR691567.1 | -- | FR691540.1 | -- | -- | -- | -- | -- |
| *Bothrolycus ater* | FJ404144.1 | AY611859.1 | -- | AY611950.1 | AY612041.1 | -- | FJ404347.1 | -- | -- | -- | -- | FJ404419.1 |
| *Bothrophthalmus brunneus* | FJ404145.1 | AY611874.1 | -- | AY611965.1 | AY612056.1 | -- | FJ404348.1 | -- | -- | -- | -- | FJ404420.1 |
| *Bothrophthalmus lineatus* | FJ404146.1 | FJ404198.1 | -- | FJ404251.1 | AF471090.1 | -- | FJ404349.1 | -- | -- | -- | -- | FJ404421.1 |
| *Bothrops alcatraz* | -- | -- | -- | -- | AY865820.1 | -- | -- | -- | -- | -- | -- | -- |
| *Bothrops alternatus* | EU867251.1 | EU867261.1 | -- | -- | EU867273.1 | -- | EU867285.1 | -- | -- | -- | -- | -- |
| *Bothrops ammodytoides* | AY223658.1 | AY223671.1 | -- | -- | AY223595.1 | -- | AY223639.1 | -- | -- | -- | -- | -- |
| *Bothrops asper* | EU624239.1 | GQ372868.1 | EU402630.1 | -- | FJ985697.1 | -- | EU624210.1 | EU390910.1 | -- | EU402838.1 | -- | -- |
| *Bothrops atrox* | GQ428495.1 | GQ428470.1 | DQ469794.1 | DQ469788.1 | AY223598.1 | -- | GQ428485.1 | -- | -- | DQ469790.1 | -- | -- |
| *Bothrops atrox* (*isabelae*) | -- | -- | -- | -- | AF292603.1 | -- | AF292641.1 | -- | -- | -- | -- | -- |
| *Bothrops bilineata* | AF057214.1 | AF057261.1 | -- | -- | AY223591.1 | -- | U41875.1 | -- | -- | -- | -- | -- |
| *Bothrops brazili* | EU867252.1 | EU867264.1 | -- | -- | EU867276.1 | -- | EU867288.1 | -- | -- | -- | -- | -- |
| *Bothrops caribbaeus* | -- | -- | -- | -- | AF292598.1 | -- | AF292636.1 | -- | -- | -- | -- | -- |
| *Bothrops chloromelas* | DQ305430.1 | DQ305453.1 | -- | -- | DQ305471.1 | -- | DQ305488.1 | -- | -- | -- | -- | -- |
| *Bothrops cotiara* | AF057217.1 | AF057264.1 | -- | -- | AY223597.1 | -- | AF292619.1 | -- | -- | -- | -- | -- |
| *Bothrops diporus* | DQ305431.1 | DQ305454.1 | -- | -- | DQ305472.1 | -- | DQ305489.1 | -- | -- | -- | -- | -- |
| *Bothrops erythromelas* | AF057219.1 | AF057266.1 | -- | -- | AY223600.1 | -- | U41877.1 | -- | -- | -- | -- | -- |
| *Bothrops fonsecai* | -- | -- | -- | -- | AF292580.1 | -- | AF292618.1 | -- | -- | -- | -- | -- |
| *Bothrops insularis* | AF057216.1 | AF057263.1 | -- | -- | AY223596.1 | -- | AF188705.1 | -- | -- | -- | -- | -- |
| *Bothrops itapetiningae* | EU867253.1 | EU867265.1 | -- | -- | EU867277.1 | -- | EU867289.1 | -- | -- | -- | -- | -- |
| *Bothrops jararaca* | EU867254.1 | EU867266.1 | -- | -- | EU867278.1 | -- | AF292627.1 | -- | -- | -- | -- | -- |
| *Bothrops jararacussu* | AY223661.1 | AY223674.1 | -- | -- | AY223602.1 | -- | AY223643.1 | -- | -- | -- | -- | -- |
| *Bothrops lanceolatus* | -- | -- | -- | -- | AF292599.1 | -- | AF292637.1 | -- | -- | -- | -- | -- |
| *Bothrops leucurus* | EU867255.1 | EU867267.1 | -- | -- | EU867279.1 | -- | AF246283.1 | -- | -- | -- | -- | -- |
| *Bothrops lojanus* | -- | -- | -- | -- | FR691566.1 | -- | FR691536.1 | -- | -- | -- | -- | -- |
| *Bothrops lutzi* | -- | -- | -- | -- | KF801130.1 | -- | KF801261.1 | -- | -- | -- | -- | -- |
| *Bothrops marajoensis* | -- | -- | -- | -- | AF292605.1 | -- | AF292643.1 | -- | -- | -- | -- | -- |
| *Bothrops marmoratus* | -- | -- | -- | -- | KF801137.1 | -- | KF801266.1 | -- | -- | -- | -- | -- |
| *Bothrops matogrossensis* | -- | -- | -- | -- | KF801148.1 | -- | FN431768.1 | -- | -- | -- | -- | -- |
| *Bothrops moojeni* | EU867256.1 | EU867268.1 | -- | -- | EU867280.1 | -- | EU867292.1 | -- | -- | -- | -- | -- |
| *Bothrops neuwiedi* | -- | JQ627282.1 | -- | -- | KF801178.1 | -- | AF292624.1 | -- | -- | -- | -- | -- |
| *Bothrops osbornei* | -- | -- | -- | -- | AF292595.1 | -- | AF292633.1 | -- | -- | -- | -- | -- |
| *Bothrops pauloensis* | EU867260.1 | EU867272.1 | -- | -- | EU867284.1 | -- | EU867296.1 | -- | -- | -- | -- | -- |
| *Bothrops pictus* | -- | -- | -- | -- | AF292583.1 | -- | AF292621.1 | -- | -- | -- | -- | -- |
| *Bothrops pubescens* | JN870180.1 | JN870192.1 | -- | -- | JN870200.1 | -- | KF801347.1 | -- | -- | -- | -- | -- |
| *Bothrops pulchra* | JN870179.1 | -- | -- | -- | -- | -- | AF292631.1 | -- | -- | -- | -- | -- |
| *Bothrops punctatus* | -- | -- | -- | -- | AF292594.1 | -- | AF292632.1 | -- | -- | -- | -- | -- |
| *Bothrops taeniata* | AF057215.1 | AF057262.1 | -- | -- | AY223592.1 | -- | AF292629.1 | -- | -- | -- | -- | -- |
| *Brachyophidium rhodogaster* | AY700992.1 | AY701023.1 | -- | -- | -- | -- | -- | -- | -- | -- | -- | -- |
| *Brachyorrhos raffrayi* | -- | -- | -- | -- | JX139713.1 | -- | -- | -- | -- | -- | -- | -- |
| *Brachyorrhos wallacei* | -- | -- | -- | -- | JX139716.1 | -- | -- | -- | -- | -- | -- | -- |
| *Brachyurophis approximans* | KF709658.1 | KF736330.1 | -- | -- | -- | -- | -- | -- | -- | -- | -- | -- |
| *Brachyurophis australis* | KF709663.1 | EU547154.1 | -- | -- | EU547056.1 | -- | EU547010.1 | -- | EU546881.1 | -- | -- | -- |
| *Brachyurophis incinctus* | KF709667.1 | -- | -- | -- | -- | -- | -- | -- | -- | -- | -- | -- |
| *Brachyurophis morrisi* | -- | KF736321.1 | -- | -- | -- | -- | -- | -- | -- | -- | -- | -- |
| *Brachyurophis roperi* | KF709666.1 | KF736372.1 | -- | -- | -- | -- | -- | -- | -- | -- | -- | -- |
| *Brachyurophis semifasciatus* | EU547107.1 | EU547155.1 | -- | EU546921.1 | EU547057.1 | -- | EU547011.1 | -- | EU546883.1 | -- | -- | -- |
| *Buhoma depressiceps* | FJ404147.1 | AY611860.1 | -- | AY611951.1 | AY612042.1 | -- | -- | -- | -- | -- | -- | FJ404422.1 |
| *Buhoma procterae* | FJ404148.1 | AY611818.1 | -- | AY611910.1 | AY612001.1 | -- | DQ486328.1 | -- | -- | -- | -- | FJ404423.1 |
| *Bungarus bungaroides* | -- | -- | -- | -- | AY973270.1 | -- | AJ830218.1 | -- | -- | -- | -- | -- |
| *Bungarus caeruleus* | -- | -- | -- | -- | AJ749305.1 | -- | AJ830220.1 | -- | -- | -- | -- | -- |
| *Bungarus candidus* | JN687932.1 | JN687933.1 | -- | -- | AJ565001.1 | JN123449.1 | AJ830234.1 | -- | -- | -- | -- | -- |
| *Bungarus ceylonicus* | KC347316.1 | KC347350.1 | -- | KC347390.1 | KC347457.1 | -- | KC347501.1 | -- | -- | KC347428.1 | -- | -- |
| *Bungarus fasciatus* | EU579523 | EU579523 | FJ433989.1 | AY058924.1 | EU579523 | AY058997.1 | EU579523 | FJ434090.1 | JF357954.1 | -- | -- | EF144100.1 |
| *Bungarus flaviceps* | -- | -- | -- | -- | AJ749351.1 | -- | AJ830251.1 | -- | -- | -- | -- | -- |
| *Bungarus multicinctus* | AF236684.2 | EU579522.1 | -- | AF435021.1 | EU579522.1 | NC_011392.1 | EU579522.1 | -- | -- | -- | -- | -- |
| *Bungarus niger* | -- | -- | -- | -- | AJ749304.1 | -- | AJ830241.1 | -- | -- | -- | -- | -- |
| *Bungarus sindanus* | -- | -- | -- | -- | AJ749346.1 | -- | AJ830242.1 | -- | -- | -- | -- | -- |
| *Bungarus slowinskii* | -- | -- | -- | -- | AJ749306.1 | -- | AJ830250.1 | -- | -- | -- | -- | -- |
| *Caaeteboia amarali* | GQ457807.1 | GQ457747.1 | -- | GQ457867.1 | JQ598921.1 | -- | -- | -- | -- | -- | -- | -- |
| *Cacophis squamulosus* | EU547101.1 | EU547150.1 | -- | EU366451.1 | EU547052.1 | -- | EU547007.1 | -- | EU366440.1 | -- | -- | -- |
| *Calabaria reinhardtii* | Z46464.1 | Z46494.1 | EU402631.1 | AF544682.1 | AY099985.1 | -- | AF302943.1 | AY988058.1 | -- | EU402839.1 | -- | FJ433902.1 |
| *Calamaria pavimentata* | -- | -- | FJ434005.1 | AF471103.1 | AF471081.1 | -- | -- | FJ434106.1 | -- | -- | EF144092.1 | EF144116.1 |
| *Calamaria yunnanensis* | JQ598801.1 | JQ598863.1 | -- | -- | JQ598922.1 | -- | -- | -- | -- | -- | -- | -- |
| *Calamodontophis paucidens* | GQ457786.1 | GQ457728.1 | -- | GQ457848.1 | -- | -- | -- | -- | -- | -- | -- | -- |
| *Calliophis bivirgata* | U96800.1 | -- | -- | AY058934.1 | AF217812.1 | AY059007.1 | AY058979.1 | -- | -- | -- | -- | -- |
| *Calliophis castoe* | -- | -- | -- | -- | -- | -- | JQ282155.1 | -- | -- | -- | -- | -- |
| *Calliophis melanurus* | KC347317.1 | KC347351.1 | -- | KC347391.1 | KC347458.1 | -- | KC347502.1 | -- | -- | KC347429.1 | -- | -- |
| *Calliophis nigrescens* | -- | -- | -- | -- | -- | -- | JQ282156.1 | -- | -- | -- | -- | -- |
| *Calliophis* sp. | -- | -- | -- | -- | AF217816.1 | -- | -- | -- | -- | -- | -- | -- |
| *Calloselasma rhodostoma* | AY352779.1 | AY352718.1 | -- | -- | AY223562.1 | -- | AY352813.1 | -- | -- | -- | -- | -- |
| *Candoia aspera* | EF545041.1 | EF545068.1 | -- | -- | U69751.1 | -- | -- | -- | -- | -- | -- | -- |
| *Candoia carinata* | AF544741.1 | EU419850.1 | FJ433974.1 | AY099961.1 | AY099984.1 | -- | -- | AY988048.1 | -- | AY988065.1 | AY988065.1 | FJ433904.1 |
| *Candoia superciliosa* | -- | -- | KF811060.1 | KF811097.1 | KF811113.1 | -- | -- | KF811131.1 | -- | KF811161.1 | -- | -- |
| *Cantoria violacea* | EF395873.1 | EF395848.1 | -- | EF395922.1 | EF395897.1 | -- | -- | -- | -- | -- | -- | -- |
| *Caraiba andreae* | AF158442.1 | AF158511.1 | -- | -- | FJ416743.1 | FJ416781.1 | FJ416817.1 | -- | -- | -- | -- | FJ416854.1 |
| *Carphophis amoenus* | AY577013.1 | AY577022.1 | -- | DQ112082.1 | AF471067.1 | -- | -- | -- | -- | -- | -- | -- |
| *Carphophis vermis* | -- | -- | -- | KP765638.1 | KP765656.1 | -- | -- | -- | -- | -- | -- | -- |
| *Casarea dussumieri* | AF544754.1 | AF544827.1 | EU402632.1 | AF544731.1 | U69755.1 | -- | -- | FJ434069.1 | -- | EU402840.1 | AY487408.1 | FJ433894.1 |
| *Causus defilippii* | GU045452 | GU045452 | EU402633.1 | -- | GU045452 | GU045452.1 | AY223617.1 | EU390913.1 | -- | -- | -- | -- |
| *Causus resimus* | AY223649.1 | AY223662.1 | -- | AF544696.1 | AY223555.1 | -- | AY223616.1 | -- | -- | -- | -- | -- |
| *Causus rhombeatus* | DQ305409.1 | DQ305432.1 | -- | -- | DQ305455.1 | -- | U41866.1 | -- | -- | -- | -- | -- |
| *Cemophora coccinea* | FJ623960.1 | -- | -- | AF471132.1 | KF216147.1 | DQ902249.1 | DQ902282.1 | KF215004.1 | -- | -- | -- | -- |
| *Cerastes cerastes* | HQ658427.1 | HQ267811.1 | -- | AF544679.1 | AF471028.1 | -- | EU624222.1 | -- | -- | EU852329.1 | -- | -- |
| *Cerastes gasperettii* | HQ658415.1 | HQ267799.1 | -- | -- | AJ275704.1 | -- | -- | -- | -- | -- | -- | -- |
| *Cerastes vipera* | -- | AJ275757.1 | -- | -- | AJ275705.1 | -- | -- | -- | -- | -- | -- | -- |
| *Cerberus australis* | EF395874.1 | EF395849.1 | -- | EF395923.1 | EF395898.1 | -- | -- | -- | -- | -- | -- | -- |
| *Cerberus microlepis* | EF395875.1 | EF395850.1 | -- | EF395924.1 | EF395899.1 | -- | -- | -- | -- | -- | -- | -- |
| *Cerberus rynchops* | EF395876.1 | EF395851.1 | -- | EF395925.1 | EF395900.1 | -- | U49327.1 | -- | EU366441.1 | -- | -- | -- |
| *Cerrophidion godmani* | EU684303.1 | DQ305442.1 | -- | -- | AY220328.1 | -- | AY220349.1 | -- | -- | -- | -- | -- |
| *Cerrophidion petlalcalensis* | DQ305420.1 | DQ305443.1 | -- | -- | DQ061202.1 | -- | DQ061227.1 | -- | -- | -- | -- | -- |
| *Cerrophidion tzotzilorum* | JN870182.1 | JN870193.1 | -- | -- | DQ061203.1 | -- | DQ061228.1 | -- | -- | -- | -- | -- |
| *Cerrophidion wilsoni* | -- | JQ627136.1 | -- | -- | -- | -- | -- | -- | -- | -- | -- | -- |
| *Charina bottae* | AF544743.1 | AF544816.1 | FJ433978.1 | AY099971.1 | AY099986.1 | -- | AF302959.1 | AY988059.1 | -- | AY988076.1 | AY487390.1 | FJ433908.1 |
| *Charina umbratica* | -- | -- | KF811062.1 | KF811099.1 | KF811115.1 | -- | AF302977.1 | KF811132.1 | -- | KF811163.1 | -- | -- |
| *Chilabothrus angulifer* | -- | -- | KC330046.1 | HQ399542.1 | HQ399513.1 | -- | KC329956.1 | HQ399533.1 | -- | HQ399523.1 | -- | -- |
| *Chilabothrus chrysogaster* | -- | -- | KC330047.1 | KC329982.1 | KC329924.1 | -- | KC329957.1 | KC330112.1 | -- | KC330015.1 | -- | -- |
| *Chilabothrus exsul* | -- | -- | KC330049.1 | KC329984.1 | U69782.1 | -- | KC329959.1 | KC330114.1 | -- | KC330017.1 | -- | -- |
| *Chilabothrus fordii* | -- | -- | KC330050.1 | KC329985.1 | U69784.1 | -- | KC329960.1 | KC330115.1 | -- | KC330018.1 | -- | -- |
| *Chilabothrus inornatus* | -- | -- | KC330052.1 | KC329987.1 | KC329929.1 | -- | KC329962.1 | KC330117.1 | -- | KC330020.1 | -- | -- |
| *Chilabothrus monensis* | -- | -- | KC330054.1 | KC329989.1 | U69792.1 | -- | KC329964.1 | KC330119.1 | -- | KC330022.1 | -- | -- |
| *Chilabothrus striatus* | -- | -- | KC330056.1 | KC329991.1 | U69791.1 | -- | KC329966.1 | KC330121.1 | -- | KC330027.1 | -- | -- |
| *Chilabothrus strigilatus* | -- | -- | KC330062.1 | KC750005.1 | KC750010.1 | -- | -- | KC750022.1 | -- | KC750045.1 | -- | -- |
| *Chilabothrus subflavus* | -- | -- | KC330070.1 | KC330005.1 | U69803.1 | -- | KC329972.1 | KC330135.1 | -- | KC330037.1 | -- | -- |
| *Chilomeniscus stramineus* | -- | -- | -- | GQ895800.1 | GQ895856.1 | -- | U49305.1 | -- | -- | -- | -- | -- |
| *Chionactis occipitalis* | -- | -- | -- | GQ895801.1 | GQ895857.1 | -- | -- | -- | -- | -- | -- | -- |
| *Chironius bicarinatus* | HM565744.1 | HM582206.1 | -- | HQ157811.1 | -- | -- | -- | -- | -- | -- | -- | -- |
| *Chironius carinatus* | HM565745.1 | HM582207.1 | -- | HQ529281.1 | HQ529280.1 | -- | -- | -- | -- | -- | -- | -- |
| *Chironius exoletus* | HM565746.1 | HM582208.1 | -- | HQ157812.1 | -- | -- | -- | -- | -- | -- | -- | -- |
| *Chironius flavolineatus* | HM565747.1 | HM582209.1 | -- | HQ157813.1 | -- | -- | -- | -- | -- | -- | -- | -- |
| *Chironius foveatus* | HM565748.1 | HM582210.1 | -- | HQ157814.1 | -- | -- | -- | -- | -- | -- | -- | -- |
| *Chironius fuscus* | HM565749.1 | HM582211.1 | -- | HQ157815.1 | -- | -- | -- | -- | -- | -- | -- | -- |
| *Chironius grandisquamis* | HM565750.1 | HM582212.1 | -- | HQ157816.1 | -- | -- | -- | -- | -- | -- | -- | -- |
| *Chironius laevicollis* | HM565751.1 | HM582213.1 | -- | HQ157817.1 | -- | -- | -- | -- | -- | -- | -- | -- |
| *Chironius laurenti* | HM565752.1 | -- | -- | HQ157818.1 | -- | -- | -- | -- | -- | -- | -- | -- |
| *Chironius monticola* | HM565753.1 | HM582214.1 | -- | HQ157819.1 | -- | -- | -- | -- | -- | -- | -- | -- |
| *Chironius multiventris* | HM565754.1 | -- | -- | -- | -- | -- | -- | -- | -- | -- | -- | -- |
| *Chironius quadricarinatus* | HM565755.1 | HM582215.1 | -- | HQ157820.1 | -- | -- | -- | -- | -- | -- | -- | -- |
| *Chironius scurrulus* | HM565756.1 | HM582216.1 | -- | HQ157821.1 | -- | -- | -- | -- | -- | -- | -- | -- |
| *Chrysopelea ornata* | KC347318.1 | KC347353.1 | -- | KC347393.1 | KC347481.1 | -- | KC347496.1 | -- | -- | KC347431.1 | -- | -- |
| *Chrysopelea paradisi* | -- | -- | -- | GQ895802.1 | GQ895858.1 | -- | -- | -- | -- | -- | -- | -- |
| *Chrysopelea taprobanica* | KC347319.1 | KC347354.1 | -- | KC347394.1 | KC347459.1 | -- | KM673290.1 | -- | -- | KC347432.1 | -- | -- |
| *Clelia clelia* | AF158403.1 | AF158472.1 | -- | JQ598973.1 | GQ895859.1 | -- | -- | -- | -- | -- | -- | -- |
| *Clonophis kirtlandii* | AF402625.1 | -- | KF258596.1 | -- | KF258647.1 | AF384827.1 | KF258630.1 | KF234026.1 | -- | -- | -- | -- |
| *Coelognathus erythrurus* | AY122665.1 | -- | -- | DQ902067.1 | DQ902108.1 | DQ902215.1 | DQ902288.1 | -- | -- | -- | -- | -- |
| *Coelognathus flavolineatus* | AY039162.1 | -- | -- | DQ902090.1 | DQ902128.1 | DQ902240.1 | DQ902308.1 | -- | -- | -- | -- | -- |
| *Coelognathus helena* | AY122675.1 | -- | -- | DQ902071.1 | DQ902112.1 | DQ902219.1 | DQ902292.1 | -- | -- | -- | -- | -- |
| *Coelognathus radiatus* | AY122676.1 | -- | -- | DQ902079.1 | DQ902121.1 | DQ902229.1 | DQ902317.1 | -- | -- | -- | -- | -- |
| *Coelognathus subradiatus* | AY122672.1 | -- | -- | DQ902084.1 | DQ902126.1 | DQ902235.1 | DQ902304.1 | -- | -- | -- | -- | -- |
| *Coluber bilineatus* | -- | -- | -- | KP765639.1 | KP765657.1 | -- | -- | -- | -- | -- | -- | -- |
| *Coluber constrictor* | AY122819.1 | L01770.1 | EU402634.1 | AY486938.1 | AY486914.1 | AY487002.1 | AY487041.1 | EU390914.1 | -- | EU402841.1 | -- | -- |
| *Coluber flagellum* | AY122823.1 | -- | -- | AY486951.1 | AY486927.1 | AY487021.1 | AY487060.1 | -- | -- | -- | -- | -- |
| *Coluber lateralis* | -- | -- | -- | KP765640.1 | KP765658.1 | -- | -- | -- | -- | -- | -- | -- |
| *Coluber schotti* | -- | -- | -- | KP765641.1 | KP765659.1 | -- | -- | -- | -- | -- | -- | -- |
| *Coluber taeniatus* | AY122669.1 | -- | -- | KP765642.1 | KP765660.1 | -- | -- | -- | -- | -- | -- | -- |
| *Compsophis albiventris* | FJ404149.1 | AY188050.1 | -- | AY187972.1 | -- | -- | FJ404351.1 | -- | -- | -- | -- | -- |
| *Compsophis boulengeri* | -- | EF204007.1 | -- | EF204001.1 | EF203995.1 | -- | -- | -- | -- | -- | -- | -- |
| *Compsophis infralineatus* | FJ404157.1 | EF204002.1 | -- | -- | EF203990.1 | -- | FJ404359.1 | -- | -- | -- | -- | FJ404431.1 |
| *Compsophis laphystius* | -- | EF204003.1 | -- | EF203997.1 | EF203991.1 | -- | -- | -- | -- | -- | -- | -- |
| *Coniophanes fissidens* | -- | -- | -- | -- | EF078538.1 | -- | EF078586.1 | GU353274.1 | -- | -- | -- | -- |
| *Conophis lineatus* | GQ457788.1 | GU018161.1 | JQ599016.1 | JQ598975.1 | JQ598924.1 | -- | -- | -- | -- | -- | -- | -- |
| *Conophis vittatus* | -- | -- | -- | GQ895806.1 | GQ895862.1 | -- | -- | -- | -- | -- | -- | -- |
| *Conopsis biserialis* | -- | -- | -- | GQ895804.1 | GQ895860.1 | -- | -- | -- | -- | -- | -- | -- |
| *Conopsis nasus* | -- | -- | -- | GQ895805.1 | GQ895861.1 | -- | -- | -- | -- | -- | -- | -- |
| *Contia longicaudae* | -- | -- | GU112366.1 | -- | GU112404.1 | -- | GU112429.1 | -- | -- | -- | -- | -- |
| *Contia tenuis* | AY577021.1 | AY577030.1 | GU112346.1 | AF471134.1 | GU112384.1 | -- | GU112419.1 | -- | -- | -- | -- | -- |
| *Corallus annulatus* | JX244285.1 | -- | JX576167.1 | KC750007.1 | KC750012.1 | -- | KC750018.1 | KC750024.1 | -- | KC750047.1 | -- | -- |
| *Corallus batesii* | -- | -- | KF811064.1 | KF811100.1 | KC750013.1 | -- | KC750019.1 | -- | -- | KC750048.1 | -- | -- |
| *Corallus caninus* | JX576162.1 | -- | JQ073072.1 | JX576174.1 | U69766.1 | -- | -- | JX576183.1 | -- | AY988061.1 | JQ073193.1 | -- |
| *Corallus cookii* | JX244287.1 | -- | JX244302.1 | JX244315.1 | HM348836.1 | -- | -- | JX244326.1 | -- | -- | -- | -- |
| *Corallus cropanii* | JX576163.1 | -- | JX576169.1 | JX576175.1 | JX576180.1 | -- | -- | JX576184.1 | -- | -- | -- | -- |
| *Corallus grenadensis* | JX244288.1 | -- | JX244303.1 | JX244316.1 | HM348837.1 | -- | -- | -- | -- | -- | -- | -- |
| *Corallus hortulanus* | JX244294.1 | -- | JX576170.1 | HQ399544.1 | HM348875.1 | -- | -- | HQ399535.1 | -- | HQ399525.1 | -- | -- |
| *Corallus ruschenbergerii* | JX244296.1 | -- | JX244311.1 | JX244322.1 | HM348841.1 | -- | -- | JX244330.1 | -- | -- | -- | -- |
| *Coronella austriaca* | AY122836.1 | EU022628.1 | -- | AY486954.1 | AY486930.1 | AY487026.1 | AY487065.1 | -- | -- | -- | -- | -- |
| *Coronella girondica* | AY122835.1 | JQ837564.1 | -- | AF471113.1 | JQ837644.1 | AY487027.1 | AY487066.1 | -- | -- | -- | -- | -- |
| *Crisantophis nevermanni* | GU018152.1 | GU018169.1 | -- | -- | -- | -- | -- | -- | -- | -- | -- | -- |
| *Crotalus adamanteus* | AF259255.1 | AF259147.1 | -- | JN620891.1 | JN620809.1 | -- | JN620959.1 | -- | -- | JN621000.1 | -- | -- |
| *Crotalus aquilus* | HQ257634.1 | AF259125.1 | -- | -- | AF259162.1 | -- | HQ257878.1 | -- | -- | -- | -- | -- |
| *Crotalus atrox* | AF259256.1 | AF259148.1 | -- | JN620893.1 | JN620811.1 | AY016239.1 | JN620961.1 | KF410312.1 | -- | JN621002.1 | -- | -- |
| *Crotalus basiliscus* | AF259244.1 | AF259136.1 | -- | -- | AY704844.1 | AY704796.1 | AY704894.1 | KF410313.1 | -- | -- | -- | -- |
| *Crotalus catalinensis* | AF259259.1 | AF259151.1 | -- | -- | AF259189.1 | -- | -- | -- | -- | -- | -- | -- |
| *Crotalus cerastes* | AF259235.1 | AF259128.1 | -- | JN620894.1 | JN620812.1 | AY016245.1 | JN620962.1 | KF410314.1 | -- | JN621003.1 | -- | -- |
| *Crotalus cerberus* | -- | -- | -- | JN620895.1 | JN620813.1 | AY016225.1 | JN620963.1 | -- | -- | -- | -- | -- |
| *Crotalus culminatus* | -- | -- | -- | -- | AY704831.1 | -- | AY704881.1 | -- | -- | -- | -- | -- |
| *Crotalus durissus* | AF259248.1 | AF259140.1 | -- | -- | AY704826.1 | AY704790.1 | AY704876.1 | -- | -- | -- | -- | -- |
| *Crotalus enyo* | AF259245.1 | AF259137.1 | -- | -- | AF259175.1 | AY016246.1 | -- | -- | -- | -- | -- | -- |
| *Crotalus ericsmithi* | -- | -- | -- | KF410301.1 | KF410284.1 | -- | KF410290.1 | KF410315.1 | -- | KF410330.1 | -- | -- |
| *Crotalus horridus* | AF259252.1 | AF259144.1 | -- | JN620897.1 | AF337057.1 | NC_014400.1 | JN620965.1 | KF410316.1 | -- | -- | -- | -- |
| *Crotalus intermedius* | JN022953.1 | AF259131.1 | -- | -- | -- | -- | JN022894.1 | -- | -- | -- | -- | -- |
| *Crotalus lannomi* | -- | -- | -- | KF410303.1 | KF410280.1 | -- | KF410292.1 | KF410317.1 | -- | KF410331.1 | -- | -- |
| *Crotalus lepidus* | HQ257533.1 | AF259123.1 | -- | JN620898.1 | JN620816.1 | -- | JN620966.1 | -- | -- | JN621005.1 | -- | -- |
| *Crotalus mitchellii* | AF259250.1 | AF259142.1 | -- | JN620899.1 | JN620817.1 | AY016241.1 | JN620967.1 | -- | -- | JN621006.1 | -- | -- |
| *Crotalus molossus* | AF259243.1 | AF259135.1 | -- | JN620902.1 | JN620820.1 | AY704797.1 | JN620970.1 | -- | -- | -- | -- | -- |
| *Crotalus oreganus* | DQ020024.1 | -- | -- | AF471135.1 | AF147871.1 | AY016217.1 | AF194162.1 | -- | -- | -- | -- | -- |
| *Crotalus polystictus* | AF259236.1 | AF259129.1 | -- | KF410305.1 | KF410281.1 | -- | KF410294.1 | KF410320.1 | -- | KF410332.1 | -- | -- |
| *Crotalus pricei* | JN022947.1 | AF259130.1 | -- | KF410306.1 | AF259167.1 | -- | JN022849.1 | KF410321.1 | -- | -- | -- | -- |
| *Crotalus pusillus* | HQ257636.1 | AF259122.1 | -- | -- | AF259159.1 | -- | HQ257880.1 | -- | -- | -- | -- | -- |
| *Crotalus ravus* | HQ257597.1 | AF057273.1 | -- | -- | AY223609.1 | -- | HQ257843.1 | -- | -- | -- | -- | -- |
| *Crotalus ruber* | AF259260.1 | AF259152.1 | -- | KP765643.1 | KP765661.1 | -- | DQ679838.1 | -- | -- | -- | -- | -- |
| *Crotalus scutulatus* | AF259254.1 | AF259146.1 | -- | -- | AF147876.1 | AY016237.1 | AF194167.1 | KF410322.1 | -- | -- | -- | -- |
| *Crotalus simus* | EU624240.1 | GQ372869.1 | -- | -- | HE867034.1 | -- | -- | -- | -- | -- | -- | -- |
| *Crotalus* sp. | HQ257557.1 | HQ257557.1 | -- | -- | -- | -- | HQ257803.1 | -- | -- | -- | -- | -- |
| *Crotalus stejnegeri* | -- | -- | -- | KF410309.1 | KF410283.1 | -- | KF410297.1 | KF410324.1 | -- | KF410333.1 | -- | -- |
| *Crotalus tancitarensis* | JN022906.1 | JN022906.1 | -- | -- | -- | -- | JN022851.1 | -- | -- | -- | -- | -- |
| *Crotalus tigris* | AF259249.1 | AF259141.1 | -- | JN620900.1 | JN620818.1 | AY016240.1 | JN620968.1 | GQ334665.1 | -- | JN621007.1 |  | -- |
| *Crotalus totonacus* | -- | -- | -- | -- | AY704838.1 | AY704795.1 | AY704888.1 | -- | -- | -- | -- | -- |
| *Crotalus transversus* | JN022895.1 | JN022895.1 | -- | -- | AF259169.1 | -- | JN022840.1 | -- | -- | -- | -- | -- |
| *Crotalus triseriatus* | AF259234.1 | AF259127.1 | -- | KF410310.1 | AF259164.1 | -- | HQ257865.1 | KF410325.1 | -- | -- | -- | -- |
| *Crotalus tzabcan* | -- | -- | -- | -- | AY704806.1 | -- | AY704856.1 | -- | -- | -- | -- | -- |
| *Crotalus viridis* | AF259253.1 | AF259145.1 | -- | JN620901.1 | AF471066.1 | AY016236.1 | JN620969.1 | -- | -- | JN621008.1 | -- | -- |
| *Crotalus willardi* | AF259241.1 | AF259133.1 | -- | -- | KF410279.1 | -- | KF410298.1 | KF410326.1 | -- | KF410335.1 | -- | -- |
| *Crotaphopeltis hotamboeia* | -- | -- | -- | -- | AF428023.1 | AF428008.1 | -- | -- | -- | -- | -- | -- |
| *Crotaphopeltis tornieri* | -- | -- | -- | AF471112.1 | AF471093.1 | AF428015.1 | -- | -- | -- | -- | -- | -- |
| *Cryophis hallbergi* | -- | -- | -- | GQ895807.1 | GQ334481.1 | -- | GQ334582.1 | GQ334666.1 | -- | -- | -- | -- |
| *Cryptophis nigrescens* | EU547119.1 | EU547168.1 | -- | EU546932.1 | EU547070.1 | -- | EU547022.1 | -- | EU546893.1 | -- | -- | -- |
| *Cubophis cantherigerus* | AF158405.1 | AF158475.1 | FJ433999.1 | AF544694.1 | AF544669.1 | FJ416782.1 | FJ416818.1 | FJ434100.1 | -- | -- | AY487376.1 | EF144109.1 |
| *Cubophis caymanus* | -- | -- | -- | -- | FJ416745.1 | FJ416784.1 | FJ416820.1 | -- | -- | -- | -- | FJ416856.1 |
| *Cubophis fuscicauda* | -- | -- | -- | -- | FJ416747.1 | FJ416786.1 | FJ416822.1 | -- | -- | -- | -- | -- |
| *Cubophis ruttyi* | -- | -- | -- | -- | FJ416746.1 | FJ416785.1 | FJ416821.1 | -- | -- | -- | -- | -- |
| *Cubophis vudii* | AF158443.1 | AF158512.1 | -- | JQ598976.1 | FJ416744.1 | FJ416783.1 | FJ416819.1 | -- | -- | -- | -- | FJ416855.1 |
| *Cyclophiops major* | -- | -- | AF497715.1 | KM870890.1 | KM870884.1 | KC347452.1 | -- | -- | -- | KM870895.1 | KM870895.1 | -- |
| *Cyclophiops multicinctus* | -- | -- | -- | KM870888.1 | KM870882.1 | -- | -- | -- | -- | KM870893.1 | KM870893.1 | -- |
| *Cylindrophis maculatus* | KC347320.1 | KC347355.1 | -- | KC347395.1 | KC347460.1 | -- | KC347494.1 | -- | -- | KC347433.1 | -- | -- |
| *Cylindrophis ruffus* | AB179619 | AB179619 | AY988037.1 | AF471133.1 | AB179619 | AY662538.1 | AB179619 | EU390915.1 | -- | AY988071.1 | -- | -- |
| *Daboia deserti* | -- | AJ275765.1 | -- | -- | AJ275712.1 | -- | -- | -- | -- | -- | -- | -- |
| *Daboia mauritanica* | EU624261.1 | EU624295.1 | -- | -- | AJ275714.1 | -- | EU624229.1 | -- | -- | -- | -- | -- |
| *Daboia palaestinae* | JN870183.1 | AJ275775.1 | -- | -- | AJ275722.1 | -- | -- | -- | -- | -- | -- | -- |
| *Daboia russelii* | EU913478 | EU913478 | EU402636.1 | AF471156.1 | EU913478 | NC_011391.1 | EU913478 | EU390916.1 | -- | EU402843.1 |  | -- |
| *Dasypeltis atra* | -- | -- | -- | AF471136.1 | AF471065.1 | -- | -- | -- | -- | -- | -- | -- |
| *Dasypeltis confusa* | -- | JQ801322.1 | -- | -- | -- | -- | -- | -- | -- | -- | -- | -- |
| *Dasypeltis fasciata* | -- | JQ801311.1 | -- | -- | -- | -- | -- | -- | -- | -- | -- | -- |
| *Dasypeltis gansi* | -- | JQ801317.1 | -- | -- | -- | -- | -- | -- | -- | -- | -- | -- |
| *Dasypeltis latericia* | -- | JQ801325.1 | -- | -- | -- | -- | -- | -- | -- | -- | -- | -- |
| *Dasypeltis sahelensis* | -- | JQ801329.1 | -- | -- | -- | -- | -- | -- | -- | -- | -- | -- |
| *Dasypeltis scabra* | -- | JQ801323.1 | -- | -- | AY235729.1 | -- | -- | -- | -- | -- | -- | -- |
| *Dasypeltis* sp. | -- | JQ801307.1 | -- | -- | -- | -- | -- | -- | -- | -- | -- | -- |
| *Deinagkistrodon acutus* | EU913476 | EU913476 | -- | -- | EU913476 | DQ836215.1 | EU913476 | -- | -- | -- | -- | -- |
| *Demansia papuensis* | EU547093.1 | EU547142.1 | -- | EU546910.1 | EU547044.1 | -- | EU547002.1 | -- | EU546871.1 | -- | -- | -- |
| *Demansia psammophis* | GQ397248.1 | GQ397240.1 | -- | GQ397230.1 | GQ397172.1 | -- | GQ397209.1 | -- | GQ397201.1 | -- | -- | -- |
| *Demansia vestigiata* | EU547094.1 | EU547143.1 | -- | AY058927.1 | EU547045.1 | AY058990.1 | EU547003.1 | -- | EU546872.1 | -- | -- | -- |
| *Dendrelaphis bifrenalis* | KC347321.1 | KC347356.1 | -- | KC347396.1 | KC347470.1 | -- | KC347509.1 | -- | -- | KC347434.1 | -- | -- |
| *Dendrelaphis calligaster* | -- | AF139569.1 | -- | -- | -- | -- | -- | -- | -- | -- | -- | -- |
| *Dendrelaphis caudolineatus* | AF544782.1 | AF544811.1 | -- | GQ895808.1 | GQ895864.1 | -- | -- | -- | -- | -- | -- | -- |
| *Dendrelaphis caudolineolatus* | -- | KC347357.1 | -- | -- | KC347484.1 | -- | KC347518.1 | -- | -- | -- | -- | -- |
| *Dendrelaphis pictus* | -- | -- | -- | -- | JX678825.1 | -- | U49304.1 | -- | -- | -- | -- | -- |
| *Dendrelaphis schokari* | -- | KC347358.1 | -- | KC347397.1 | KC347461.1 | -- | KC347497.1 | -- | -- | KC347435.1 | -- | -- |
| *Dendrelaphis tristis* | KC347322.1 | KC347359.1 | -- | KC347398.1 | KC347462.1 | -- | KC347493.1 | -- | -- | KC347436.1 | -- | -- |
| *Dendroaspis angusticeps* | AF544764.1 | FJ404194.1 | FJ433988.1 | AF544735.1 | -- | -- | JF357927.1 | FJ434089.1 | -- | -- | AY487395.1 | EF144099.1 |
| *Dendroaspis polylepis* | -- | -- | -- | FJ387197.1 | FJ404295.1 | AY059003.1 | AY058974.1 | -- | -- | -- | -- | -- |
| *Dendrophidion dendrophis* | -- | -- | -- | GQ895809.1 | GQ895865.1 | -- | -- | -- | -- | -- | -- | -- |
| *Dendrophidion percarinatum* | HM565757.1 | HM582217.1 | -- | HQ157822.1 | -- | -- | -- | -- | -- | -- | -- | -- |
| *Denisonia devisi* | EU547120.1 | EU547169.1 | -- | EU546933.1 | EU547071.1 | -- | EU547023.1 | -- | EU546894.1 | -- | -- | -- |
| *Diadophis punctatus* | AY577015.1 | AF544793.1 | EU402637.1 | AF471122.1 | AF471094.1 | -- | EU194091.1 | GU353272.1 | -- | -- | AY487403.1 | EF144110.1 |
| *Dieurostus dussumieri* | -- | JX463016.1 | -- | JX463012.1 | JX463014.1 | -- | -- | -- | -- | -- | -- | -- |
| *Dipsadoboa shrevei* | -- | -- | -- | -- | AF428038.1 | AF428018.1 | -- | -- | -- | -- | -- | -- |
| *Dipsadoboa unicolor* | -- | -- | -- | AF471139.1 | AF471062.1 | AF428017.1 | -- | -- | -- | -- | -- | -- |
| *Dipsadoboa werneri* | -- | -- | -- | -- | -- | AF428014.1 | -- | -- | -- | -- | -- | -- |
| *Dipsas albifrons* | JQ598803.1 | JQ598866.1 | JQ599019.1 | -- | JQ598925.1 | -- | -- | -- | -- | -- | -- | -- |
| *Dipsas articulata* | JQ598804.1 | JQ598867.1 | JQ599020.1 | -- | -- | -- | -- | -- | -- | -- | -- | -- |
| *Dipsas catesbyi* | Z46459.1 | Z46496.1 | JQ599021.1 | JQ598977.1 | JQ598926.1 | -- | -- | GU353277.1 | -- | -- | -- | -- |
| *Dipsas indica* | AF158421.1 | GQ457730.1 | -- | GQ457850.1 | -- | -- | -- | -- | -- | -- | -- | -- |
| *Dipsas neivai* | GQ457790.1 | GQ457731.1 | -- | GQ457851.1 | -- | -- | -- | -- | -- | -- | -- | -- |
| *Dipsas pratti* | -- | -- | -- | -- | GQ334482.1 | -- | GQ334583.1 | GQ334667.1 | -- | -- | -- | -- |
| *Dipsas variegata* | GQ457790.1 | GQ457731.1 | -- | -- | -- | -- | -- | -- | -- | -- | -- | -- |
| *Dipsina multimaculata* | -- | -- | -- | DQ486181.1 | DQ486357.1 | -- | DQ486332.1 | -- | -- | -- | -- | -- |
| *Dispholidus typus* | -- | AY188051.1 | -- | AY187973.1 | -- | -- | U49302.1 | -- | -- | -- | -- | -- |
| *Ditypophis* sp. | -- | -- | JQ073079.1 | -- | -- | -- | -- | -- | -- | JQ073200.1 | JQ073200.1 | -- |
| *Ditypophis vivax* | FJ404150.1 | AY188052.1 | -- | AY187974.1 | -- | -- | FJ404352.1 | -- | -- | -- | -- | FJ404424.1 |
| *Dolichophis caspius* | AY039126.1 | AY376768.1 | -- | AY376797.1 | HM210786.1 | AY487000.1 | AY487039.1 | -- | -- | -- | -- | -- |
| *Dolichophis cypriensis* | AY541502.1 | -- | -- | -- | JX315475.1 | -- | -- | -- | -- | -- | -- | -- |
| *Dolichophis jugularis* | AY039150.1 | AY376769.1 | -- | AY486941.1 | AY486917.1 | AY487007.1 | AY487046.1 | -- | -- | -- | -- | -- |
| *Dolichophis schmidti* | AY039159.1 | AY376772.1 | -- | AY376801.1 | AY486923.1 | AY487015.1 | AY487054.1 | -- | -- | -- | -- | -- |
| *Drepanoides anomalus* | GQ457791.1 | GQ457732.1 | -- | GQ895810.1 | GQ895866.1 | -- | -- | -- | -- | -- | -- | -- |
| *Dromicodryas bernieri* | FJ404151.1 | AY188053.1 | -- | AY187975.1 | DQ979987.1 | -- | FJ404353.1 | -- | -- | -- | -- | FJ404425.1 |
| *Dromicodryas quadrilineatus* | -- | AY188054.1 | -- | AY187976.1 | DQ979989.1 | -- | -- | -- | -- | -- | -- | -- |
| *Drymarchon corais* | HM565758.1 | HM582218.1 | -- | AF471137.1 | AF471064.1 | DQ902207.1 | DQ902314.1 | -- | -- | -- | -- | -- |
| *Drymarchon couperi* | -- | -- | -- | KP765646.1 | KP765662.1 | -- | -- | -- | -- | -- | -- | -- |
| *Drymobius rhombifer* | HM565761.1 | HM582220.1 | -- | GQ927313.1 | GQ927320.1 | -- | -- | -- | -- | -- | -- | -- |
| *Drymoluber brazili* | HM565760.1 | HM582219.1 | -- | HQ157825.1 | -- | -- | -- | -- | -- | -- | -- | -- |
| *Drymoluber dichrous* | HM565759.1 | HM582221.1 | -- | HQ157824.1 | GQ895869.1 | -- | -- | -- | -- | -- | -- | -- |
| *Dryocalamus nympha* | KC347323.1 | KC347360.1 | -- | KC347399.1 | KC347476.1 | -- | KC347498.1 | -- | -- | KC347437.1 | -- | -- |
| *Drysdalia coronoides* | U96796.1 | EU547173.1 | -- | EU546937.1 | EU547075.1 | -- | GU062880.1 | -- | EU546898.1 | -- | -- | -- |
| *Drysdalia mastersii* | EU547125.1 | EU547174.1 | -- | EU546938.1 | EU547076.1 | -- | EU547028.1 | -- | EU546899.1 | -- | -- | -- |
| *Drysdalia rhodogaster* | -- | -- | -- | -- | GU062854.1 | -- | GU062884.1 | -- | -- | -- | -- | -- |
| *Duberria lutrix* | FJ404154.1 | FJ404207.1 | -- | DQ486161.1 | DQ486337.1 | -- | DQ486313.1 | -- | -- | -- | -- | FJ404428.1 |
| *Duberria variegata* | FJ404155.1 | FJ404208.1 | -- | DQ486165.1 | DQ486341.1 | -- | DQ486317.1 | -- | -- | -- | -- | FJ404429.1 |
| *Echinanthera melanostigma* | GU018153.1 | GU018174.1 | -- | -- | JQ598928.1 | -- | -- | -- | -- | -- | -- | -- |
| *Echinanthera undulata* | JQ598807.1 | JQ598870.1 | JQ599022.1 | JQ598978.1 | JQ598929.1 | -- | -- | -- | -- | -- | -- | -- |
| *Echiopsis curta* | EU547121.1 | EU547170.1 | -- | EU546934.1 | EU547072.1 | -- | EU547024.1 | -- | EU546895.1 | -- | -- | -- |
| *Echis borkini* | GQ359645.1 | GQ359729.1 | -- | -- | GQ359480.1 | -- | GQ359564.1 | -- | -- | -- | -- | -- |
| *Echis carinatus* | GQ359604.1 | GQ359685.1 | -- | -- | GQ359436.1 | -- | GQ359524.1 | -- | -- | EU852325.1 | -- | -- |
| *Echis coloratus* | GQ359628.1 | GQ359712.1 | -- | -- | GQ359462.1 | -- | GQ359547.1 | -- | -- | EU852327.1 | -- | -- |
| *Echis jogeri* | GQ359648.1 | GQ359732.1 | -- | -- | GQ359483.1 | -- | GQ359567.1 | -- | -- | -- | -- | -- |
| *Echis khosatzkii* | GQ359615.1 | GQ359698.1 | -- | -- | GQ359449.1 | -- | GQ359534.1 | -- | -- | -- | -- | -- |
| *Echis leucogaster* | GQ359620.1 | GQ359703.1 | -- | -- | GQ359454.1 | -- | GQ359539.1 | -- | -- | -- | -- | -- |
| *Echis ocellatus* | GQ359594.1 | GQ359676.1 | -- | -- | GQ359426.1 | -- | GQ359515.1 | -- | -- | EU852324.1 | -- | -- |
| *Echis omanensis* | GQ359631.1 | GQ359715.1 | -- | -- | GQ359466.1 | -- | GQ359550.1 | -- | -- | -- | -- | -- |
| *Echis pyramidum* | GQ359611.1 | GQ359694.1 | -- | -- | GQ359445.1 | -- | GQ359530.1 | -- | -- | EU852326.1 | -- | -- |
| *Eirenis aurolineatus* | -- | AY376778.1 | -- | AY376807.1 | AY376749.1 | AY487031.1 | AY487070.1 | -- | -- | -- | -- | -- |
| *Eirenis barani* | -- | AY376785.1 | -- | AY376804.1 | AY376746.1 | -- | -- | -- | -- | -- | -- | -- |
| *Eirenis collaris* | -- | AY376795.1 | -- | AY376824.1 | AY376766.1 | -- | -- | -- | -- | -- | -- | -- |
| *Eirenis coronelloides* | -- | AY376787.1 | -- | AY376816.1 | AY376758.1 | -- | -- | -- | -- | -- | -- | -- |
| *Eirenis decemlineatus* | -- | AY376789.1 | -- | AY376818.1 | AY376760.1 | -- | -- | -- | -- | -- | -- | -- |
| *Eirenis eiselti* | -- | AY376776.1 | -- | -- | AY376747.1 | AY487030.1 | AY487069.1 | -- | -- | -- | -- | -- |
| *Eirenis hakkariensis* | -- | AY376790.1 | -- | AY376819.1 | AY376761.1 | -- | -- | -- | -- | -- | -- | -- |
| *Eirenis levantinus* | -- | AY376794.1 | -- | AY376808.1 | AY376765.1 | AY487032.1 | AY487071.1 | -- | -- | -- | -- | -- |
| *Eirenis lineomaculatus* | -- | AY376791.1 | -- | AY376820.1 | AY376762.1 | -- | -- | -- | -- | -- | -- | -- |
| *Eirenis medus* | AY647226.1 | AY376796.1 | -- | AY376825.1 | AY376767.1 | -- | -- | -- | -- | -- | -- | -- |
| *Eirenis modestus* | AY039160.1 | AY376792.1 | -- | AY486957.1 | AY486933.1 | AY487033.1 | AY487072.1 | -- | -- | -- | -- | -- |
| *Eirenis persicus* | -- | AY376786.1 | -- | AY376815.1 | AY376757.1 | -- | -- | -- | -- | -- | -- | -- |
| *Eirenis punctatolineatus* | AY647227.1 | AY376781.1 | -- | AY376810.1 | AY376755.1 | AY487034.1 | AY487073.1 | -- | -- | -- | -- | -- |
| *Eirenis rothii* | -- | AY376788.1 | -- | AY376817.1 | AY376759.1 | -- | -- | -- | -- | -- | -- | -- |
| *Eirenis thospitis* | -- | AY376783.1 | -- | AY376812.1 | AY376754.1 | -- | -- | -- | -- | -- | -- | -- |
| *Elaphe anomala* | AY122803.1 | NC_027001.1 | -- | -- | NC_027001.1 | NC_027001.1 | NC_027001.1 | -- | -- | -- | -- | -- |
| *Elaphe bimaculata* | AY122768.1 | -- | -- | DQ902062.1 | DQ902104.1 | DQ902210.1 | NC_024743.1 | -- | -- | -- | -- | -- |
| *Elaphe carinata* | AF236674.2 | HM439984.1 | -- | DQ902063.1 | KF669252.1 | DQ902211.1 | DQ902284.1 | -- | -- | -- | -- | -- |
| *Elaphe climacophora* | AY122772.1 | -- | -- | DQ902064.1 | DQ902105.1 | DQ902212.1 | DQ902285.1 | -- | -- | -- | -- | -- |
| *Elaphe davidi* | AY122775.1 | KM401547.1 | -- | -- | KM401547.1 | NC_025643.1 | KM401547.1 | -- | -- | -- | -- | -- |
| *Elaphe dione* | AF236673.2 | -- | -- | DQ902066.1 | DQ902107.1 | DQ902214.1 | DQ902287.1 | -- | -- | -- | -- | -- |
| *Elaphe quadrivirgata* | AY122794.1 | -- | -- | DQ902078.1 | DQ902120.1 | DQ902228.1 | DQ902300.1 | -- | -- | -- | -- | -- |
| *Elaphe quatuorlineata* | AY122798.1 | AF215267.1 | -- | AY486955.1 | AY486931.1 | AY487028.1 | AY487067.1 | -- | -- | -- | -- | -- |
| *Elaphe sauromates* | AY122795.1 | -- | -- | -- | -- | -- | -- | -- | -- | -- | -- | -- |
| *Elaphe schrenckii* | JQ815337.1 | -- | -- | DQ902082.1 | DQ902124.1 | DQ902233.1 | DQ902302.1 | -- | -- | -- | -- | -- |
| *Elaphe zoigeensis* | HQ330522.1 | -- | -- | -- | HQ330524.1 | -- | HQ330526.1 | -- | -- | -- | -- | -- |
| *Elapognathus coronatus* | EU547118.1 | EU547167.1 | -- | EU546931.1 | EU547069.1 | AY058995.1 | EU547021.1 | -- | EU546892.1 | -- | -- | -- |
| *Elapomorphus quinquelineatus* | GQ457794.1 | GQ457735.1 | JQ599023.1 | GQ457855.1 | JQ598930.1 | -- | -- | -- | -- | -- | -- | -- |
| *Elapotinus picteti* | -- | -- | -- | -- | EU394724.1 | -- | -- | -- | -- | -- | -- | -- |
| *Elapsoidea nigra* | U96804.1 | -- | -- | AY058930.1 | AF217820.1 | AY059004.1 | AY058975.1 | -- | -- | -- | -- | -- |
| *Elapsoidea semiannulata* | AF544745.1 | JF357946.1 | FJ433987.1 | AF544678.1 | AF039260.1 | -- | JF357928.1 | FJ434088.1 | -- | -- | AY487373.1 | EF144098.1 |
| *Elapsoidea sundevallii* | -- | AY188055.1 | -- | AY187977.1 | AY188016.1 | -- | -- | -- | -- | -- | -- | -- |
| *Emydocephalus annulatus* | EU547136.1 | DQ234001.1 | -- | FJ587172.1 | DQ233940.1 | -- | FJ593196.1 | -- | FJ587094.1 | -- | -- | -- |
| *Emydocephalus ijimae* | D31610.1 | -- | -- | -- | -- | -- | -- | -- | -- | -- | -- | -- |
| *Enhydris enhydris* | EF395879.1 | EF395855.1 | -- | EF395929.1 | EF395904.1 | -- | GU997190.1 | -- | -- | -- | -- | -- |
| *Enhydris innominata* | EF395880.1 | -- | -- | EF395930.1 | EF395905.1 | -- | -- | -- | -- | -- | -- | -- |
| *Enhydris jagorii* | AF499284.1 | AF499298.1 | -- | -- | AF499270.1 | -- | GU997192.1 | -- | -- | -- | -- | -- |
| *Enhydris longicauda* | EF395882.1 | EF395858.1 | -- | EF395932.1 | EF395907.1 | -- | GU997189.1 | -- | -- | -- | -- | -- |
| *Enhydris subtaeniata* | EF395881.1 | EF395857.1 | -- | EF395931.1 | JN392073.1 | -- | JN400729.1 | -- | -- | -- | -- | -- |
| *Enulius* sp. | -- | -- | -- | GQ895813.1 | GQ895870.1 | -- | -- | -- | -- | -- | -- | -- |
| *Ephalophis greyae* | -- | FJ587208.1 | -- | FJ587173.1 | JX002976.1 | -- | FJ593197.1 | -- | FJ587095.1 | -- | -- | -- |
| *Epicrates alvarezi* | -- | -- | -- | HQ399541.1 | HQ399512.1 | -- | -- | HQ399531.1 | -- | HQ399521.1 | -- | -- |
| *Epicrates assisi* | -- | -- | -- | HQ399539.1 | HQ399503.1 | -- | -- | HQ399529.1 | -- | -- | -- | -- |
| *Epicrates cenchria* | AF368059.1 | -- | KC330073.1 | KC330008.1 | HQ399501.1 | -- | KC329975.1 | HQ399528.1 | -- | KC330040.1 | -- | -- |
| *Epicrates crassus* | -- | AF215273.1 | -- | HQ399540.1 | HQ399504.1 | -- | -- | HQ399530.1 | -- | HQ399520.1 | -- | -- |
| *Epicrates maurus* | -- | -- | KC330074.1 | KC330009.1 | KC329951.1 | -- | KC329976.1 | KC330139.1 | -- | KC330041.1 | -- | -- |
| *Epictia columbi* | GQ469211.1 | GQ469211.1 | GQ469178.1 | AF544718.1 | AF544671.1 | -- | -- | GQ469018.1 | -- | -- | GQ469041.1 | FJ433887.1 |
| *Epictia goudotii* | GQ469220.1 | GQ469220.1 | -- | -- | -- | -- | -- | -- | -- | -- | -- | -- |
| *Epictia magnamaculata* | GQ469216.1 | GQ469216.1 | -- | -- | -- | -- | -- | -- | -- | -- | -- | -- |
| *Epictia tenella* | GQ469224.1 | GQ469224.1 | GQ469180.1 | GQ469065.1 | -- | -- | -- | GQ469020.1 | -- | -- | GQ469043.1 | -- |
| *Eristicophis macmahoni* | EU624259.1 | EU624293.1 | -- | -- | AJ275711.1 | -- | EU624227.1 | -- | -- | -- | -- | -- |
| *Erpeton tentaculatum* | EF395888.1 | EF395864.1 | -- | EF395936.1 | EF395913.1 | -- | -- | -- | -- | -- | -- | -- |
| *Erythrolamprus aesculapii* | GQ457795.1 | GQ457736.1 | JQ599024.1 | GQ895814.1 | GQ895871.1 | -- | -- | -- | -- | -- | -- | -- |
| *Erythrolamprus almadensis* | JQ598808.1 | JQ598871.1 | -- | JQ598979.1 | -- | -- | -- | -- | -- | -- | -- | -- |
| *Erythrolamprus atraventer* | JQ598809.1 | JQ598872.1 | -- | JQ598980.1 | -- | -- | -- | -- | -- | -- | -- | -- |
| *Erythrolamprus breviceps* | AF158464.1 | AF158533.1 | -- | -- | -- | -- | -- | -- | -- | -- | -- | -- |
| *Erythrolamprus ceii* | JQ598810.1 | JQ598873.1 | -- | JQ598981.1 | -- | -- | -- | -- | -- | -- | -- | -- |
| *Erythrolamprus cursor* | JX905307.1 | JX905311.1 | -- | -- | -- | -- | -- | -- | -- | -- | -- | -- |
| *Erythrolamprus epinephelus* | GU018158.1 | GU018176.1 | -- | -- | -- | -- | -- | -- | -- | -- | -- | -- |
| *Erythrolamprus jaegeri* | GQ457809.1 | GQ457749.1 | -- | GQ457869.1 | -- | -- | -- | -- | -- | -- | -- | -- |
| *Erythrolamprus juliae* | AF158445.1 | AF158514.1 | -- | -- | -- | -- | -- | -- | -- | -- | -- | -- |
| *Erythrolamprus miliaris* | AF158409.1 | AF158480.1 | JQ599025.1 | JQ598982.1 | JQ598931.1 | -- | -- | -- | -- | -- | -- | -- |
| *Erythrolamprus mimus* | GU018157.1 | GU018175.1 | -- | -- | -- | -- | -- | -- | -- | -- | -- | -- |
| *Erythrolamprus poecilogyrus* | JQ598812.1 | JQ598875.1 | -- | -- | -- | -- | -- | -- | -- | -- | -- | -- |
| *Erythrolamprus pygmaeus* | GU018154.1 | GU018172.1 | -- | -- | -- | -- | -- | -- | -- | -- | -- | -- |
| *Erythrolamprus reginae* | AF158433.1 | AF158501.1 | -- | GQ895819.1 | GQ895876.1 | -- | -- | -- | -- | -- | -- | -- |
| *Erythrolamprus typhlus* | GQ457811.1 | GQ457751.1 | -- | GQ457871.1 | -- | -- | -- | -- | -- | -- | -- | -- |
| *Eryx colubrinus* | AF544747.1 | AF544819.1 | EU402639.1 | AF544716.1 | U69811.1 | -- | -- | DQ465569.1 | -- | DQ465571.1 |  | FJ433906.1 |
| *Eryx conicus* | GQ225680.1 | AF512743.1 | AY988040.1 | GQ225672.1 | GQ225658.1 | -- | -- | -- | -- | AY988074.1 | -- | -- |
| *Eryx elegans* | -- | -- | -- | -- | U69819.1 | -- | -- | -- | -- | -- | -- | -- |
| *Eryx jaculus* | -- | -- | -- | -- | U69821.1 | -- | -- | -- | -- | -- | -- | -- |
| *Eryx jayakari* | HQ658444.1 | HQ267804.1 | DQ465566.1 | DQ465565.1 | -- | -- | -- | -- | -- | DQ465567.1 | -- | -- |
| *Eryx johnii* | -- | -- | DQ465576.1 | AY099975.1 | AY099987.1 | -- | -- | DQ465575.1 | -- | DQ465577.1 | -- | -- |
| *Eryx miliaris* | AF544746.1 | AF544818.1 | FJ433977.1 | AF544683.1 | U69824.1 | -- | AF302942.1 | -- | -- | -- | AY487393.1 | FJ433907.1 |
| *Eryx muelleri* | -- | -- | KF811065.1 | KF811101.1 | KF811116.1 | -- | -- | -- | -- | KF811164.1 | -- | -- |
| *Eryx tataricus* | AF236681.2 | -- | KF811066.1 | AF435018.1 | U69830.1 | -- | -- | -- | -- | KF811165.1 | -- | -- |
| *Eunectes murinus* | JX576166.1 | -- | KC330075.1 | HQ399537.1 | U69808.1 | -- | KC329977.1 | KC330140.1 | -- | KC330042.1 | -- | -- |
| *Eunectes notaeus* | AF368057.1 | AM236347.1 | KC330076.1 | HQ399536.1 | HQ399499.1 | AM236347.1 | KC329978.1 | HQ399526.1 | -- | HQ399516.1 | -- | -- |
| *Euprepiophis conspicillata* | AY122773.1 | -- | -- | DQ902065.1 | KF669241.1 | DQ902213.1 | DQ902286.1 | -- | -- | -- | -- | -- |
| *Euprepiophis mandarinus* | AY122784.1 | -- | -- | DQ902073.1 | DQ902115.1 | DQ902222.1 | DQ902294.1 | -- | -- | -- | -- | -- |
| *Euprepiophis perlacea* | KF850472.1 | KF850472.1 | -- | -- | KF669239.1 | KF850472.1 | KF850472.1 | -- | -- | -- | -- | -- |
| *Exiliboa placata* | AF512742.1 | AF512742.1 | EU402640.1 | AY099973.1 | AY099989.1 | -- | -- | AY988051.1 | -- | AY988068.1 | -- | -- |
| *Farancia abacura* | Z46467.1 | Z46491.1 | -- | AF471141.1 | U69832.1 | DQ902239.1 | U49307.1 | -- | -- | -- | -- | -- |
| *Farancia erytrogramma* | AY577017.1 | AY577026.1 | -- | KP765647.1 | KP765663.1 | -- | -- | -- | -- | -- | -- | -- |
| *Ficimia streckeri* | -- | -- | -- | GQ927314.1 | -- | -- | -- | -- | -- | -- | -- | -- |
| *Fordonia leucobalia* | EF395889.1 | EF395865.1 | -- | EF395937.1 | EF395914.1 | -- | -- | -- | -- | -- | -- | -- |
| *Furina diadema* | EU547102.1 | EU547151.1 | -- | EU546917.1 | EU547053.1 | -- | EU547008.1 | -- | EU546878.1 | -- | -- | -- |
| *Furina ornata* | EU547103.1 | EU547152.1 | -- | EU546918.1 | EU547054.1 | -- | EU547009.1 | -- | EU546879.1 | -- | -- | -- |
| *Garthius chaseni* | AY352791.1 | AY352729.1 | -- | -- | AY352760.1 | -- | AY352825.1 | -- | -- | -- | -- | -- |
| *Geophis carinosus* | -- | -- | -- | GQ895815.1 | GQ895872.1 | -- | -- | -- | -- | -- | -- | -- |
| *Geophis dubius* | -- | -- | -- | -- | KC917316.1 | -- | -- | -- | -- | -- | -- | -- |
| *Geophis godmani* | JQ598814.1 | JQ598877.1 | JQ599026.1 | -- | JQ598932.1 | -- | -- | -- | -- | -- | -- | -- |
| *Geophis juarezi* | -- | -- | -- | -- | KC917315.1 | -- | -- | -- | -- | -- | -- | -- |
| *Geophis latifrontalis* | -- | -- | -- | -- | KC917322.1 | -- | -- | -- | -- | -- | -- | -- |
| *Geophis occabus* | -- | -- | -- | -- | KC917323.1 | -- | -- | -- | -- | -- | -- | -- |
| *Geophis turbidus* | -- | -- | -- | -- | KC917321.1 | -- | -- | -- | -- | -- | -- | -- |
| *Gerarda prevostiana* | EF395891.1 | EF395867.1 | -- | EF395939.1 | EF395916.1 | -- | -- | -- | -- | -- | -- | -- |
| *Gerrhopilus hedraeus* | -- | -- | GU902392.1 | -- | -- | -- | -- | GU902565.1 | -- | -- | GU902642.1 | -- |
| *Gerrhopilus mirus* | AM236345.1 | AM236345.1 | GU902394.1 | -- | AM236345.1 | AM236345.1 | AM236345.1 | GU902566.1 | -- | -- | GU902644.1 | -- |
| *Gloydius blomhoffii* | AY352780.1 | AY352719.1 | -- | -- | AY352751.1 | -- | AY352814.1 | -- | -- | -- | -- | -- |
| *Gloydius brevicaudus* | JQ815354.1 | EU913477 | -- | JQ687515.1 | JQ687496.1 | DQ839399.1 | JQ687477.1 | -- | -- | -- | -- | -- |
| *Gloydius halys* | AF057191.1 | AF057238.1 | -- | -- | AY223564.1 | AY662540.1 | JQ356856.1 | -- | AY662614.1 | AY662614.1 | AY662614.1 | -- |
| *Gloydius intermedius* | EF012806.1 | JN870194.1 | -- | -- | JQ687488.1 | NC_025560.1 | JQ687469.1 | -- | -- | -- | -- | -- |
| *Gloydius liupanensis* | -- | -- | -- | JQ687510.1 | JQ687491.1 | -- | JQ687472.1 | -- | -- | -- | -- | -- |
| *Gloydius saxatilis* | JN870185.1 | JN870195.1 | -- | JQ687521.1 | JQ687502.1 | NC_025666.1 | JQ687483.1 | -- | -- | -- | -- | -- |
| *Gloydius shedaoensis* | EF012812.1 | AF057241.1 | -- | JQ687517.1 | JQ687498.1 | -- | JQ687479.1 | -- | -- | -- | -- | -- |
| *Gloydius strauchi* | EF012814.1 | AF057239.1 | -- | JQ687506.1 | JQ687487.1 | -- | JQ687468.1 | -- | -- | -- | -- | -- |
| *Gloydius tsushimaensis* | JN870186.1 | JN870196.1 | -- | -- | JN870203.1 | -- | JN870211.1 | -- | -- | -- | -- | -- |
| *Gloydius ussuriensis* | EF012818.1 | AF057240.1 | -- | JQ687520.1 | JQ687501.1 | NC_026553.1 | JQ687482.1 | -- | -- | -- | -- | -- |
| *Gomesophis brasiliensis* | GQ457796.1 | GQ457737.1 | -- | -- | -- | -- | -- | -- | -- | -- | -- | -- |
| *Gonionotophis brussauxi* | FJ404156.1 | AY611861.1 | -- | AY611952.1 | AY612043.1 | -- | FJ404358.1 | -- | -- | -- | -- | FJ404430.1 |
| *Gonionotophis capensis* | AF544769.1 | AF544798.1 | FJ433995.1 | AF544703.1 | HQ207116.1 | -- | HQ207158.1 | FJ434096.1 | -- | -- | AY487379.1 | EF144106.1 |
| *Gonionotophis chanleri* | FJ404184.1 | FJ404196.1 | -- | DQ486172.1 | DQ486348.1 | -- | FJ404381.1 | -- | -- | -- | -- | FJ404455.1 |
| *Gonionotophis nyassae* | FJ404182.1 | AY611896.1 | -- | AY611987.1 | HQ207108.1 | -- | HQ207150.1 | -- | -- | -- | -- | FJ404453.1 |
| *Gonionotophis poensis* | FJ404183.1 | AY611863.1 | -- | AY611954.1 | AY612045.1 | -- | FJ404380.1 | -- | -- | -- | -- | FJ404454.1 |
| *Gonionotophis stenophthalmus* | FJ404185.1 | AY611835.1 | -- | AY611927.1 | AY612018.1 | -- | FJ404382.1 | -- | -- | -- | -- | FJ404456.1 |
| *Gonyosoma boulengeri* | -- | -- | -- | AF471153.1 | AF471053.1 | -- | -- | -- | -- | KM870899.1 | KM870899.1 | -- |
| *Gonyosoma frenatus* | AY122777.1 | -- | -- | DQ902069.1 | DQ902110.1 | DQ902217.1 | DQ902290.1 | -- | -- | KM870897.1 | KM870897.1 | -- |
| *Gonyosoma jansenii* | AY122671.1 | -- | -- | DQ902100.1 | DQ902113.1 | DQ902220.1 | DQ902313.1 | -- | -- | -- | -- | -- |
| *Gonyosoma margaritatus* | -- | -- | -- | KM870891.1 | KM870891 | -- | -- | -- | -- | KM870896 | KM870896 | -- |
| *Gonyosoma oxycephalum* | AY122678.1 | Z46490.1 | -- | KC010302.1 | AF471084.1 | DQ902241.1 | DQ902309.1 | -- | -- | -- | -- | -- |
| *Gonyosoma prasinus* | AY122792.1 | -- | -- | DQ902077.1 | DQ902119.1 | DQ902227.1 | DQ902299.1 | -- | -- | KM870898.1 | KM870898.1 | -- |
| *Grayia ornata* | AF158434.1 | AF158503.1 | FJ434002.1 | AF544684.1 | -- | -- | AF544663.1 | FJ434103.1 | -- | -- | EF144091.1 | EF144113.1 |
| *Grayia smithii* | -- | -- | -- | DQ112080.1 | DQ112077.1 | -- | -- | -- | -- | -- | -- | -- |
| *Grayia tholloni* | -- | -- | -- | DQ486175.1 | DQ486351.1 | -- | DQ486326.1 | -- | -- | -- | -- | -- |
| *Gyalopion canum* | -- | -- | -- | GQ927315.1 | -- | -- | -- | -- | -- | -- | -- | -- |
| *Haitiophis anomalus* | FJ666091.1 | FJ666092.1 | -- | -- | -- | -- | -- | -- | -- | -- | -- | -- |
| *Haldea striatula* | AF402649.1 | -- | KF258606.1 | -- | KF258657.1 | AF384852.1 | KF258640.1 | KF234036.1 | -- | -- | -- | -- |
| *Hapsidophrys lineatus* | -- | AY611873.1 | -- | -- | AY612055.1 | -- | -- | -- | -- | -- | -- | -- |
| *Hapsidophrys principis* | -- | FJ913476.1 | -- | -- | FJ913492.1 | -- | -- | -- | -- | -- | -- | -- |
| *Hapsidophrys smaragdina* | AF158435.1 | AY611875.1 | FJ434003.1 | DQ112078.1 | AY612057.1 | -- | -- | FJ434104.1 | -- | -- | AY487381.1 | EF144114.1 |
| *Hebius atemporale* | -- | -- | -- | KJ685645.1 | KJ685645.1 | -- | -- | KJ685747.1 | KJ685587.1 | -- | -- | -- |
| *Hebius bitaeniatum* | -- | -- | -- | KJ685636.1 | KJ685636.1 | -- | -- | KJ685738.1 | KJ685578.1 | -- | -- | -- |
| *Hebius boulengeri* | -- | -- | -- | KJ685641.1 | KJ685641.1 | -- | -- | KJ685743.1 | KJ685583.1 | -- | -- | -- |
| *Hebius craspedogaster* | -- | -- | -- | KJ685622.1 | JQ687429.1 | JQ687459.1 | JQ687412.1 | KJ685723.1 | KJ685573.1 | -- | -- | -- |
| *Hebius deschauenseei* | -- | -- | -- | KJ685614.1 | KJ685614.1 | -- | -- | KJ685715.1 | KJ685558.1 | -- | -- | -- |
| *Hebius johannis* | -- | -- | -- | KJ685628.1 | KJ685628.1 | -- | -- | KJ685731.1 | KJ685605.1 | -- | -- | -- |
| *Hebius khasiense* | -- | -- | -- | KJ685618.1 | KJ685618.1 | -- | -- | KJ685719.1 | KJ685562.1 | -- | -- | -- |
| *Hebius metusium* | -- | -- | -- | KJ685657.1 | KJ685657.1 | -- | -- | KJ685766.1 | -- | -- | -- | -- |
| *Hebius modestum* | -- | -- | -- | KJ685620.1 | KJ685620.1 | -- | -- | KJ685721.1 | KJ685564.1 | -- | -- | -- |
| *Hebius octolineatum* | -- | -- | -- | KJ685624.1 | KJ685624.1 | -- | -- | KJ685725.1 | -- | -- | -- | -- |
| *Hebius optatum* | -- | -- | -- | KJ685637.1 | KJ685637.1 | -- | -- | KJ685739.1 | KJ685555.1 | -- | -- | -- |
| *Hebius parallelum* | -- | -- | -- | KJ685615.1 | KJ685615.1 | -- | -- | KJ685716.1 | KJ685559.1 | -- | -- | -- |
| *Hebius popei* | -- | -- | -- | KJ685642.1 | KJ685642.1 | -- | -- | KJ685744.1 | KJ685588.1 | -- | -- | -- |
| *Hebius sauteri* | AF402622.1 | -- | -- | KJ685651.1 | AF402905.1 | AF384824.1 | -- | KJ685754.1 | KJ685592.1 | -- | -- | -- |
| *Hebius* sp. | -- | -- | -- | KJ685635.1 | KJ685635.1 | -- | -- | KJ685737.1 | KJ685575.1 | -- | -- | -- |
| *Hebius venningi* | -- | -- | -- | KJ685650.1 | KJ685619.1 | -- | -- | KJ685752.1 | KJ685590.1 | -- | -- | -- |
| *Hebius vibakari* | JQ815346.1 | -- | -- | KJ685627.1 | JQ798797.1 | KP684155.1 | -- | KJ685729.1 | KJ685568.1 | -- | -- | -- |
| *Helicops angulatus* | GQ457797.1 | GQ457738.1 | JQ599027.1 | AF471160.1 | AF471037.1 | FJ416751.1 | -- | -- | -- | -- | -- | FJ416824.1 |
| *Helicops carinicaudus* | JQ598815.1 | -- | -- | JQ598984.1 | -- | -- | -- | -- | -- | -- | -- | -- |
| *Helicops gomesi* | GQ457798.1 | GQ457739.1 | -- | GQ457858.1 | -- | -- | -- | -- | -- | -- | -- | -- |
| *Helicops hagmanni* | JQ598816.1 | JQ598878.1 | -- | JQ598985.1 | -- | -- | -- | -- | -- | -- | -- | -- |
| *Helicops infrataeniatus* | GQ457799.1 | GQ457740.1 | -- | GQ457859.1 | JQ598933.1 | -- | U49310.1 | -- | -- | -- | -- | -- |
| *Hemachatus haemachatus* | U96797.1 | -- | -- | -- | AF217821.1 | -- | -- | -- | -- | -- | -- | -- |
| *Hemerophis socotrae* | AY039140.1 | AY188083.1 | -- | AY188003.1 | AY188042.1 | AY487016.1 | AY487055.1 | -- | -- | -- | -- | -- |
| *Hemerophis zebrinus* | -- | AY188084.1 | -- | AY188004.1 | AY188043.1 | AY487019.1 | AY487058.1 | -- | -- | -- | -- | -- |
| *Hemiaspis damelii* | EU547122.1 | DQ233979.1 | -- | FJ587161.1 | EU547073.1 | -- | FJ593193.1 | -- | EU546896.1 | -- | -- | -- |
| *Hemiaspis signata* | EU547123.1 | DQ233980.1 | -- | EU546936.1 | EU547074.1 | -- | EU547026.1 | -- | EU546897.1 | -- | -- | -- |
| *Hemibungarus calligaster* | -- | -- | -- | EF137419.1 | EF137411.1 | -- | EF137403.1 | -- | -- | -- | -- | -- |
| *Hemirhagerrhis hildebrandtii* | FJ404134.1 | FJ404214.1 | -- | FJ387214.1 | DQ486418.1 | -- | FJ404337.1 | -- | -- | -- | -- | FJ404409.1 |
| *Hemirhagerrhis kelleri* | -- | -- | -- | DQ486159.1 | DQ486335.1 | -- | DQ486311.1 | -- | -- | -- | -- | -- |
| *Hemirhagerrhis viperina* | -- | -- | -- | -- | DQ486453.1 | -- | DQ486289.1 | -- | -- | -- | -- | -- |
| *Hemorrhois algirus* | AY643307.1 | AY643349.1 | -- | AY486935.1 | AY486911.1 | AY486998.1 | AY487037.1 | -- | -- | -- | -- | -- |
| *Hemorrhois hippocrepis* | DQ451994.1 | AY643350.1 | -- | AY486940.1 | AY486916.1 | AY487006.1 | AY487045.1 | -- | -- | -- | -- | -- |
| *Hemorrhois nummifer* | AY039163.1 | AY376771.1 | -- | AY376800.1 | AY376742.1 | AY487010.1 | AY487049.1 | -- | -- | -- | -- | -- |
| *Hemorrhois ravergieri* | AY039131.1 | -- | -- | AY486944.1 | AY486920.1 | AY487011.1 | AY487050.1 | -- | -- | -- | -- | -- |
| *Herpetoreas burbrinki* | -- | -- | -- | -- | -- | -- | -- | KJ685761.1 | KJ685599.1 | -- | -- | -- |
| *Herpetoreas platyceps* | -- | -- | -- | KJ685640.1 | KJ685690.1 | -- | -- | KJ685742.1 | KJ685582.1 | -- | -- | -- |
| *Heterodon nasicus* | GQ457801.1 | AY577027.1 | -- | GQ457861.1 | KP765664.1 | -- | -- | -- | -- | -- | -- | -- |
| *Heterodon platirhinos* | AY577019.1 | AY577028.1 | JQ599028.1 | JQ598986.1 | GU112412.1 | FJ416750.1 | AF402659.1 | GU353271.1 | -- | -- | -- | FJ416823.1 |
| *Heterodon simus* | AY577020.1 | AY577029.1 | -- | AF471142.1 | -- | DQ902242.1 | DQ902310.1 | -- | -- | -- | -- | -- |
| *Heteroliodon occipitalis* | FJ404158.1 | FJ404211.1 | -- | FJ387211.1 | FJ404308.1 | -- | -- | -- | -- | -- | -- | FJ404432.1 |
| *Heteroliodon* sp. | -- | AY188057.1 | -- | AY187979.1 | -- | -- | -- | -- | -- | -- | -- | -- |
| *Hierophis andreanus* | AY647225.1 | -- | -- | -- | -- | -- | -- | -- | -- | -- | -- | -- |
| *Hierophis gemonensis* | AY039145.1 | AY376770.1 | -- | AY376799.1 | AY376741.1 | AY487005.1 | AY487044.1 | -- | -- | -- | -- | -- |
| *Hierophis viridiflavus* | AY643310.1 | AY376774.1 | -- | AY486949.1 | AY486925.1 | AY487018.1 | AY487057.1 | -- | -- | -- | -- | -- |
| *Homalopsis buccata* | EF395892.1 | AF499302.1 | EU402642.1 | EF395940.1 | EF395917.1 | -- | -- | FJ434087.1 | -- | -- | -- | EF144097.1 |
| *Homoroselaps lacteus* | FJ404135.1 | AY611809.1 | JQ599029.1 | AY611901.1 | AY611992.1 | AY059000.1 | FJ404338.1 | -- | -- | -- | -- | FJ404410.1 |
| *Hoplocephalus bitorquatus* | EU547128.1 | EU547177.1 | -- | EU546941.1 | EU547079.1 | -- | EU547031.1 | -- | EU546902.1 | -- | -- | -- |
| *Hoplocephalus bungaroides* | -- | -- | -- | -- | -- | -- | FJ516555.1 | -- | -- | -- | -- | -- |
| *Hormonotus modestus* | FJ404159.1 | FJ404195.1 | -- | FJ404261.1 | FJ404296.1 | -- | FJ404360.1 | -- | -- | -- | -- | FJ404433.1 |
| *Hydrelaps darwiniensis* | EU547133.1 | DQ234046.1 | -- | FJ587175.1 | KC014413.1 | -- | FJ593200.1 | -- | FJ587098.1 | -- | -- | -- |
| *Hydrodynastes bicinctus* | AF158430.1 | GQ457742.1 | JQ599030.1 | GQ457862.1 | JQ598935.1 | -- | -- | -- | -- | -- | -- | -- |
| *Hydrodynastes gigas* | GQ457803.1 | GQ457743.1 | JQ599031.1 | GQ895816.1 | GQ895873.1 | -- | -- | -- | -- | -- | -- | -- |
| *Hydromorphus concolor* | -- | -- | -- | GQ895817.1 | GQ895874.1 | -- | -- | -- | -- | -- | -- | -- |
| *Hydrophis atriceps* | -- | JQ217152.1 | -- | KC014291.1 | KC014395.1 | -- | JQ217216.1 | -- | KC014262.1 | -- | -- | -- |
| *Hydrophis belcheri* | -- | KC014321.1 | -- | KC014292.1 | KC014397.1 | -- | KC014472.1 | -- | KC014264.1 | -- | -- | -- |
| *Hydrophis brookii* | -- | KC014324.1 | -- | FJ587188.1 | KC014401.1 | -- | KC014475.1 | -- | FJ587110.1 | -- | -- | -- |
| *Hydrophis caerulescens* | -- | JQ217154.1 | -- | KC014294.1 | KC014403.1 | -- | JQ217218.1 | -- | KC014266.1 | -- | -- | -- |
| *Hydrophis coggeri* | -- | KC014328.1 | -- | KC014295.1 | KC014406.1 | -- | KC014479.1 | -- | KC014267.1 | -- | -- | -- |
| *Hydrophis curtus* | EU547134.1 | DQ234040.1 | -- | FJ587199.1 | DQ233968.1 | -- | FJ593227.1 | -- | FJ587122.1 | -- | -- | -- |
| *Hydrophis cyanocinctus* | -- | FJ587211.1 | -- | FJ587189.1 | KC572598.1 | -- | FJ593213.1 | -- | FJ587111.1 | -- | -- | -- |
| *Hydrophis czeblukovi* | -- | DQ234019.1 | -- | -- | DQ233944.1 | -- | -- | -- | -- | -- | -- | -- |
| *Hydrophis donaldi* | -- | JQ217147.1 | -- | KC014308.1 | JQ217203.1 | -- | JQ217211.1 | -- | KC014285.1 | -- | -- | -- |
| *Hydrophis elegans* | -- | DQ234021.1 | -- | FJ587190.1 | DQ233950.1 | -- | FJ593216.1 | -- | FJ587113.1 | -- | -- | -- |
| *Hydrophis fasciatus* | -- | KC014340.1 | -- | KC014298.1 | KC014417.1 | -- | KC014492.1 | -- | KC014270.1 | -- | -- | -- |
| *Hydrophis gracilis* | -- | KC014341.1 | -- | KC014299.1 | KC014420.1 | -- | KC014494.1 | -- | KC014271.1 | -- | -- | -- |
| *Hydrophis jerdonii* | -- | KC014373.1 | -- | -- | KC014450.1 | -- | KC014524.1 | -- | KC014283.1 | -- | -- | -- |
| *Hydrophis kingii* | -- | DQ234011.1 | -- | FJ587183.1 | DQ233930.1 | -- | FJ593207.1 | -- | FJ587106.1 | -- | -- | -- |
| *Hydrophis lamberti* | -- | KC014345.1 | -- | KC014300.1 | KC014421.1 | -- | KC014496.1 | -- | KC014272.1 | -- | -- | -- |
| *Hydrophis lapemoides* | -- | DQ234033.1 | -- | KC014301.1 | DQ233954.1 | -- | FJ593218.1 | -- | KC014273.1 | -- | -- | -- |
| *Hydrophis macdowelli* | -- | DQ234029.1 | -- | FJ587193.1 | JX002978.1 | -- | FJ593220.1 | -- | FJ587116.1 | -- | -- | -- |
| *Hydrophis major* | -- | DQ234018.1 | -- | FJ587186.1 | DQ233937.1 | -- | FJ593210.1 | -- | FJ587108.1 | -- | -- | -- |
| *Hydrophis melanocephalus* | D31609.1 | -- | -- | -- | KC572601.1 | -- | -- | -- | -- | -- | -- | -- |
| *Hydrophis obscurus* | -- | KC014353.1 | -- | -- | KC014431.1 | -- | KC014505.1 | -- | KC014276.1 | -- | -- | -- |
| *Hydrophis ornatus* | -- | DQ234027.1 | -- | FJ587194.1 | KC014436.1 | -- | KC014509.1 | -- | FJ587117.1 | -- | -- | -- |
| *Hydrophis pachycercos* | -- | KC014360.1 | -- | KC014303.1 | KC014439.1 | -- | KC014511.1 | -- | KC014279.1 | -- | -- | -- |
| *Hydrophis pacificus* | -- | DQ234035.1 | -- | FJ587197.1 | DQ233963.1 | -- | FJ593225.1 | -- | FJ587120.1 | -- | -- | -- |
| *Hydrophis parviceps* | -- | KC014362.1 | -- | KC014304.1 | KC014441.1 | -- | KC014513.1 | -- | KC014280.1 | -- | -- | -- |
| *Hydrophis peronii* | -- | DQ234004.1 | -- | FJ587180.1 | JQ217200.1 | -- | FJ593203.1 | -- | FJ587102.1 | -- | -- | -- |
| *Hydrophis platurus* | -- | KC014376.1 | -- | FJ587201.1 | KC014454.1 | -- | KC014529.1 | -- | FJ587124.1 | -- | -- | -- |
| *Hydrophis schistosus* | -- | FJ587210.1 | -- | FJ587187.1 | FJ587155.1 | -- | FJ593211.1 | -- | FJ587109.1 | -- | -- | -- |
| *Hydrophis semperi* | U96798.1 | -- | -- | -- | AF217822.1 | -- | -- | -- | -- | -- | -- | -- |
| *Hydrophis spiralis* | -- | KC014366.1 | -- | KC014305.1 | KC014446.1 | -- | KC014517.1 | -- | KC014281.1 | -- | -- | -- |
| *Hydrophis stokesii* | -- | DQ234010.1 | -- | FJ587182.1 | KC014391.1 | -- | FJ593206.1 | -- | FJ587104.1 | -- | -- | -- |
| *Hydrophis stricticollis* | -- | KC014371.1 | -- | KC014306.1 | KC014448.1 | -- | KC014521.1 | -- | KC014282.1 | -- | -- | -- |
| *Hydrophis viperinus* | -- | KC014379.1 | -- | KC014307.1 | JX002980.1 | -- | JX002986.1 | -- | KC014284.1 | -- | -- | -- |
| *Hydrophis zweifeli* | -- | JX987143.1 | -- | JX987185.1 | JX987166.1 | -- | JX987174.1 | -- | JX987177.1 | -- | -- | -- |
| *Hydrops triangularis* | GQ457804.1 | GQ457744.1 | JQ599032.1 | AF471158.1 | -- | -- | -- | -- | -- | -- | -- | -- |
| *Hypnale hypnale* | AY352778.1 | AY352717.1 | -- | -- | AY352750.1 | -- | AY352812.1 | -- | -- | -- | -- | -- |
| *Hypnale nepa* | KC347325.1 | KC347362.1 | -- | KC347401.1 | KC347485.1 | -- | KC347491.1 | -- | -- | KC347439.1 | -- | -- |
| *Hypnale zara* | KC347326.1 | KC347363.1 | -- | KC347402.1 | KC347463.1 | -- | KC347513.1 | -- | -- | KC347440.1 | -- | -- |
| *Hypsiglena affinis* | -- | -- | -- | -- | -- | -- | EU363055.1 | GU353278.1 | -- | -- | -- | -- |
| *Hypsiglena chlorophaea* | -- | KF548588.1 | -- | -- | KJ486459.1 | EU728587.1 | EU728577 | FJ455198.1 | -- | -- | -- | -- |
| *Hypsiglena jani* | -- | -- | -- | -- | EU728592 | EU728592.1 | EU728592 | FJ455193.1 | -- | -- | -- | -- |
| *Hypsiglena ochrorhyncha* | -- | -- | -- | -- | EU728582 | EU728578.1 | EU728582 | FJ455199.1 | -- | -- | -- | -- |
| *Hypsiglena slevini* | -- | -- | -- | -- | EU728584 | EU728584.1 | EU728584 | FJ455191.1 | -- | -- | -- | -- |
| *Hypsiglena tanzeri* | -- | -- | -- | -- | EU728588.1 | -- | EU363044.1 | -- | -- | -- | -- | -- |
| *Hypsiglena torquata* | -- | -- | -- | AF471159.1 | AF471038.1 | EU728591.1 | GQ334584.1 | FJ455192.1 | -- | -- | -- | -- |
| *Hypsiglena unaocularus* | -- | KF548589.1 | -- | -- | NC_024164.1 | KJ486458.1 | KF548605.1 | -- | -- | -- | -- | -- |
| *Hypsirhynchus callilaemus* | AF158440.1 | AF158509.1 | -- | -- | FJ416737.1 | FJ416775.1 | FJ416811.1 | -- | -- | -- | -- | FJ416848.1 |
| *Hypsirhynchus ferox* | AF158447.1 | AF158515.1 | -- | GQ895818.1 | GQ895875.1 | FJ416780.1 | FJ416816.1 | -- | -- | -- | -- | FJ416853.1 |
| *Hypsirhynchus funereus* | AF158451.1 | AF158520.1 | -- | -- | FJ416739.1 | FJ416777.1 | FJ416813.1 | -- | -- | -- | -- | FJ416850.1 |
| *Hypsirhynchus parvifrons* | AF158441.1 | AF158510.1 | JQ599006.1 | -- | FJ416740.1 | FJ416778.1 | FJ416814.1 | -- | -- | -- | -- | FJ416851.1 |
| *Hypsirhynchus polylepis* | AF158450.1 | AF158519.1 | -- | -- | FJ416738.1 | FJ416776.1 | FJ416812.1 | -- | -- | -- | -- | FJ416849.1 |
| *Hypsirhynchus scalaris* | AF158449.1 | AF158518.1 | -- | -- | FJ416741.1 | FJ416779.1 | FJ416815.1 | -- | -- | -- | -- | FJ416852.1 |
| *Hypsiscopus matannensis* | EF395883.1 | EF395859.1 | -- | EF473654.1 | EF395908.1 | -- | -- | -- | -- | -- | -- | -- |
| *Hypsiscopus plumbea* | EF395885.1 | EF395861.1 | -- | EF395934.1 | EF395910.1 | NC_010200.1 | U49328.1 | -- | -- | -- | -- | -- |
| *Hypsiscopus* sp. | EF395894.1 | EF395870.1 | -- | -- | EF395919.1 | -- | -- | -- | -- | -- | -- | -- |
| *Ialtris dorsalis* | AF158456.1 | AF158525.1 | -- | -- | FJ416735.1 | FJ416773.1 | FJ416809.1 | -- | -- | -- | -- | FJ416846.1 |
| *Ialtris haetianus* | AF158458.1 | AF158527.1 | -- | -- | FJ416736.1 | FJ416774.1 | FJ416810.1 | -- | -- | -- | -- | FJ416847.1 |
| *Imantodes cenchoa* | GQ457805.1 | GQ457745.1 | EU402643.1 | GQ457865.1 | GQ334484.1 | EU728586.1 | GQ334589 | GQ334669.1 | -- | EU402847.1 | -- | -- |
| *Imantodes chocoensis* | -- | -- | -- | -- | KC176250.1 | -- | KC176261.1 | -- | -- | -- | -- | -- |
| *Imantodes gemmistratus* | -- | -- | -- | -- | GQ334487.1 | -- | GQ334588.1 | -- | -- | -- | -- | -- |
| *Imantodes inornatus* | -- | -- | -- | -- | GQ334489.1 | -- | GQ334590.1 | GU353279.1 | -- | -- | -- | -- |
| *Imantodes lentiferus* | AF158463.1 | AF158532.1 | -- | -- | KC176252.1 | -- | KC176263.1 | -- | -- | -- | -- | -- |
| *Indotyphlops albiceps* | -- | -- | GU902382.1 | -- | -- | -- | -- | GU902555.1 | -- | -- | GU902632.1 | -- |
| *Indotyphlops braminus* | DQ343649.1 | DQ343649 | FJ433959.1 | AF544717.1 | DQ343649 | AY662539.1 | DQ343649 | GU902556.1 | -- | AY444062.1 | AY444062.1 | FJ433889.1 |
| *Indotyphlops pammeces* | -- | -- | GU902458.1 | -- | -- | -- | -- | GU902628.1 | -- | -- | GU902703.1 | -- |
| *Indotyphlops* sp. | -- | KC347375.1 | -- | -- | KC347488.1 | -- | KC347510.1 | -- | -- | KC347450.1 | -- | -- |
| *Inyoka swazicus* | -- | -- | -- | HQ207089.1 | HQ207130.1 | -- | HQ207173.1 | -- | -- | -- | -- | -- |
| *Ithycyphus miniatus* | -- | AY188058.1 | -- | AY187980.1 | -- | -- | -- | -- | -- | -- | -- | -- |
| *Ithycyphus oursi* | FJ404160.1 | FJ404212.1 | -- | FJ387212.1 | FJ404309.1 | -- | FJ404361.1 | -- | -- | -- | -- | FJ404434.1 |
| *Lachesis acrochorda* | JN870187.1 | JN870197.1 | -- | -- | JN870204.1 | -- | JN870212.1 | -- | -- | -- | -- | -- |
| *Lachesis melanocephala* | -- | -- | -- | -- | U96018.1 | -- | U96028.1 | -- | -- | -- | -- | -- |
| *Lachesis muta* | AF057221.1 | AF057268.1 | -- | -- | AY223604.1 | -- | U41885.1 | EU390924.1 | -- | -- | -- | -- |
| *Lachesis stenophrys* | AF057220.1 | AF057267.1 | EU402644.1 | -- | AY223603.1 | -- | U96026.1 | -- | -- | -- | -- | -- |
| *Lampropeltis abnorma* | -- | -- | -- | -- | KF216379.1 | -- | -- | KF215048.1 | -- | -- | -- | -- |
| *Lampropeltis alterna* | -- | -- | -- | FJ627799.1 | KF216444.1 | FJ627846.1 | AY497307.1 | KF215110.1 | -- | -- | -- | -- |
| *Lampropeltis annulata* | -- | -- | -- | -- | KF216178.1 | -- | -- | KF215061.1 | -- | -- | -- | -- |
| *Lampropeltis californiae* | -- | -- | -- | -- | AF337058.1 | -- | -- | KF215022.1 | -- | -- | -- | -- |
| *Lampropeltis calligaster* | -- | -- | -- | DQ902091.1 | KF216289.1 | DQ902243.1 | AY739644.1 | KF215015.1 | -- | -- | -- | -- |
| *Lampropeltis elapsoides* | -- | -- | -- | FJ627795.1 | KF216258.1 | -- | -- | KF215103.1 | -- | -- | -- | -- |
| *Lampropeltis extenuata* | FJ623964.1 | -- | -- | DQ902093.1 | KF216424.1 | DQ902245.1 | AF138776.1 | KF215005.1 | -- | -- | -- | -- |
| *Lampropeltis gentilis* | -- | -- | -- | -- | KF216224.1 | -- | AY739632.1 | KF215055.1 | -- | -- | -- | -- |
| *Lampropeltis getula* | AY122822.1 | -- | EU402645.1 | FJ627796.1 | AF337071.1 | -- | DQ360446.1 | KF215000.1 | -- | EU402848.1 | -- | -- |
| *Lampropeltis holbrooki* | -- | -- | -- | -- | AF337061.1 | -- | DQ360481.1 | KF215016.1 | -- | -- | -- | -- |
| *Lampropeltis knoblochi* | -- | -- | -- | -- | AF337143.1 | -- | AY497313.1 | KF215007.1 | -- | -- | -- | -- |
| *Lampropeltis mexicana* | FJ623962.1 | -- | -- | FJ627800.1 | KF216238.1 | FJ627836.1 | AY497310.1 | KF214997.1 | -- | -- | -- | -- |
| *Lampropeltis micropholis* | -- | -- | -- | -- | KF216437.1 | -- | -- | KF215085.1 | -- | -- | -- | -- |
| *Lampropeltis nigra* | -- | -- | -- | -- | AF337153.1 | -- | DQ360485.1 | KF215019.1 | -- | -- | -- | -- |
| *Lampropeltis polyzona* | -- | -- | -- | -- | KF216175.1 | -- | -- | KF215049.1 | -- | -- | -- | -- |
| *Lampropeltis pyromelana* | FJ623961.1 | -- | -- | FJ627794.1 | AF337132.1 | FJ627845.1 | AY497313.1 | KF215006.1 | -- | -- | -- | -- |
| *Lampropeltis ruthveni* | AY122820.1 | -- | -- | FJ627803.1 | AF337064.1 | FJ627847.1 | AY739641.1 | KF215014.1 | -- | -- | -- | -- |
| *Lampropeltis splendida* | -- | -- | -- | -- | AF337081.1 | -- | AY739629.1 | KF215021.1 | -- | -- | -- | -- |
| *Lampropeltis triangulum* | FJ623963.1 | -- | -- | FJ627798.1 | KF216244.1 | FJ627837.1 | FJ850979.1 | KF215056.1 | -- | -- | -- | -- |
| *Lampropeltis webbi* | -- | -- | -- | -- | JX648605.1 | -- | AY497308.1 | KF215008.1 | -- | -- | -- | -- |
| *Lampropeltis zonata* | -- | -- | -- | FJ627802.1 | AF337149.1 | -- | AF138762.1 | KF215018.1 | -- | -- | -- | -- |
| *Lamprophis aurora* | -- | -- | -- | HQ207101.1 | HQ207125.1 | -- | HQ207185.1 | -- | -- | -- | -- | -- |
| *Lamprophis fiskii* | FJ404163.1 | FJ404202.1 | -- | HQ207087.1 | DQ486354.1 | -- | HQ207166.1 | -- | -- | -- | -- | FJ404437.1 |
| *Lamprophis fuscus* | -- | -- | -- | -- | HQ207127.1 | -- | HQ207169.1 | -- | -- | -- | -- | -- |
| *Lamprophis guttatus* | FJ404166.1 | AY611890.1 | -- | HQ207094.1 | HQ207138.1 | -- | HQ207177.1 | -- | -- | -- | -- | FJ404439.1 |
| *Langaha madagascariensis* | FJ404172.1 | AY188059.1 | -- | AY187981.1 | -- | -- | FJ404370.1 | -- | -- | -- | -- | FJ404445.1 |
| *Laticauda colubrina* | U96799.1 | EU547138.1 | FJ433990.1 | EU366446.1 | EU547040.1 | AY058993.1 | FJ606513.1 | EU390927.1 | EU366433.1 | -- | AY487404.1 | EF144101.1 |
| *Laticauda frontalis* | -- | FJ587206.1 | -- | FJ587157.1 | -- | -- | FJ593190.1 | -- | FJ587081.1 | -- | -- | -- |
| *Laticauda guineai* | -- | -- | -- | -- | -- | -- | FJ606516.1 | -- | -- | -- | -- | -- |
| *Laticauda laticaudata* | D31608.1 | FJ587204.1 | -- | FJ587159.1 | FJ587153.1 | -- | FJ593192.1 | -- | FJ587083.1 | -- | -- | -- |
| *Laticauda saintgironsi* | -- | -- | -- | -- | -- | -- | FJ606506.1 | -- | -- | -- | -- | -- |
| *Laticauda semifasciata* | -- | -- | -- | -- | AB701339.1 | -- | -- | -- | -- | -- | -- | -- |
| *Leioheterodon geayi* | -- | AY188060.1 | -- | AY187982.1 | -- | -- | -- | -- | -- | -- | -- | -- |
| *Leioheterodon madagascariensis* | AF544768.1 | AY188061.1 | FJ433992.1 | AY187983.1 | -- | -- | U49318.1 | FJ434093.1 | -- | -- | AY487377.1 | EF144103.1 |
| *Leioheterodon modestus* | -- | JF357951.1 | -- | AY058933.1 | -- | AY059010.1 | JF357933.1 | -- | -- | -- | -- | -- |
| *Leptodeira annulata* | GQ457806.1 | GQ457746.1 | FJ433998.1 | AF544690.1 | GQ334493.1 | FJ416749.1 | GQ334594.1 | FJ434099.1 | -- | -- | AY487375.1 | EF144108.1 |
| *Leptodeira bakeri* | -- | -- | -- | -- | GQ334516.1 | -- | GQ334619.1 | GQ334673.1 | -- | -- | -- | -- |
| *Leptodeira frenata* | -- | -- | -- | -- | EF078532.1 | -- | EF078580.1 | FJ810242.1 | -- | -- | -- | -- |
| *Leptodeira maculata* | -- | -- | -- | -- | GQ334521.1 | -- | GQ334624.1 | GQ334674.1 | -- | -- | -- | -- |
| *Leptodeira nigrofasciata* | -- | -- | -- | -- | GQ334525.1 | -- | GQ334628.1 | GQ334681.1 | -- | -- | -- | -- |
| *Leptodeira polysticta* | EU728590.1 | EU728590.1 | -- | -- | GQ334547.1 | NC_013990.1 | GQ334652.1 | GQ334679.1 | -- | -- | -- | -- |
| *Leptodeira punctata* | -- | -- | -- | -- | EF078529.1 | -- | EF078577.1 | GQ334682.1 | -- | -- | -- | -- |
| *Leptodeira rubricata* | -- | -- | -- | -- | GQ334527.1 | -- | GQ334631.1 | -- | -- | -- | -- | -- |
| *Leptodeira septentrionalis* | GU018148.1 | GU018163.1 | -- | -- | EU728590 | -- | GQ334644.1 | GQ334678.1 | -- | -- | -- | -- |
| *Leptodeira splendida* | -- | -- | -- | -- | GQ334548.1 | -- | GQ334658.1 | GQ334680.1 | -- | -- | -- | -- |
| *Leptodeira uribei* | -- | -- | -- | -- | EF078531.1 | -- | EF078579.1 | FJ810243.1 | -- | -- | -- | -- |
| *Leptophis ahaetulla* | HM565762.1 | HM582222.1 | -- | GQ927316.1 | GQ927321.1 | -- | -- | -- | -- | -- | -- | -- |
| *Leptotyphlops conjunctus* | GQ469273.1 | GQ469280.1 | GQ469184.1 | GQ469069.1 | -- | -- | -- | GQ469023.1 | -- | -- | GQ469046.1 | -- |
| *Leptotyphlops distanti* | GQ469271.1 | GQ469271.1 | GQ469181.1 | GQ469066.1 | -- | -- | -- | GQ469021.1 | -- | -- | GQ469044.1 | -- |
| *Leptotyphlops kafubi* | GQ469253.1 | GQ469253.1 | GQ469183.1 | GQ469068.1 | -- | -- | -- | -- | -- | -- | -- | -- |
| *Leptotyphlops merkeri* | GQ469260.1 | GQ469260.1 | -- | -- | -- | -- | -- | -- | -- | -- | -- | -- |
| *Leptotyphlops nigricans* | GQ469236.1 | GQ469236.1 | -- | -- | -- | -- | -- | -- | -- | -- | -- | -- |
| *Leptotyphlops nigroterminus* | GQ469257.1 | GQ469257.1 | GQ469188.1 | GQ469073.1 | -- | -- | -- | GQ469027.1 | -- | -- | GQ469050.1 | -- |
| *Leptotyphlops pitmani* | GQ469255.1 | GQ469255.1 | -- | -- | -- | -- | -- | -- | -- | -- | -- | -- |
| *Leptotyphlops scutifrons* | GQ469270.1 | GQ469270.1 | -- | -- | -- | -- | -- | -- | -- | -- | -- | -- |
| *Leptoptyphlops* sp. |  |  | KF992887.1 |  |  |  |  | KF992929.1 |  |  | KF992950.1 |  |
| *Leptotyphlops sylvicolus* | GQ469272.1 | GQ469272.1 | GQ469192.1 | GQ469077.1 | -- | -- | -- | GQ469031.1 | -- | -- | GQ469054.1 | -- |
| *Letheobia episcopus* | -- | -- | KC848449.1 | -- | -- | -- | -- | KC848457.1 | -- | -- | -- | -- |
| *Letheobia feae* | -- | -- | KF992868.1 | -- | -- | -- | -- | KF992910.1 | -- | -- | KF992931.1 | -- |
| *Letheobia newtoni* | -- | -- | GU902388.1 | -- | -- | -- | -- | GU902561.1 | -- | -- | GU902638.1 | -- |
| *Letheobia simonii* | HQ113894.1 | -- | KC848448.1 | -- | -- | HQ113934.1 | -- | KC848459.1 | -- | -- | -- | -- |
| *Liasis fuscus* | EF545025.1 | EF545052.1 | -- | -- | EF545100.1 | -- | -- | -- | -- | -- | -- | -- |
| *Liasis mackloti* | EF545024.1 | EF545051.1 | FJ433970.1 | AF544726.1 | U69839.1 | -- | -- | FJ434075.1 | -- | -- | AY487397.1 | DQ119639.1 |
| *Liasis olivaceus* | EF545023.1 | EF545050.1 | -- | -- | U69841.1 | -- | -- | -- | -- | -- | -- | -- |
| *Liasis papuana* | EF545027.1 | EF545054.1 | FJ433971.1 | AF544720.1 | U69843.1 | -- | -- | FJ434076.1 | -- | -- | AY487405.1 | FJ433901.1 |
| *Lichanura trivirgata* | AF544749.1 | GQ200595.1 | EU402649.1 | AF544687.1 | U69844.1 | GQ200595.1 | AF302944.1 | DQ465578.1 | -- | EU402852.1 | -- | DQ119638.1 |
| *Liodytes alleni* | AF402633.1 | -- | KF258602.1 | -- | AF402916.1 | AF384835.1 | KF258636.1 | KF234032.1 | -- | -- | -- | -- |
| *Liodytes pygaea* | AF402637.1 | -- | KF258603.1 | -- | AF402920.1 | AF384839.1 | KF258637.1 | KF234033.1 | -- | -- | -- | -- |
| *Liodytes rigida* | AF402636.1 | -- | KF258608.1 | AF471120.1 | AF471052.1 | AF384838.1 | KF258642.1 | KF234038.1 | -- | -- | -- | -- |
| *Liophidium chabaudi* | FJ404173.1 | FJ404210.1 | -- | FJ387210.1 | EU394721.1 | -- | FJ404372.1 | -- | -- | -- | -- | FJ404446.1 |
| *Liophidium maintikibo* | -- | GQ913669.1 | -- | GQ913663.1 | EU394723.1 | -- | -- | -- | -- | -- | -- | -- |
| *Liophidium mayottensis* | -- | GQ913670.1 | -- | GQ913664.1 | GQ913675.1 | -- | HE798407.1 | -- | -- | -- | -- | -- |
| *Liophidium pattoni* | -- | GQ913671.1 | -- | GQ913665.1 | GQ913676.1 | -- | -- | -- | -- | -- | -- | -- |
| *Liophidium rhodogaster* | -- | DQ979964.1 | -- | DQ979971.1 | DQ979978.1 | -- | -- | -- | -- | -- | -- | -- |
| *Liophidium therezieni* | -- | GQ913673.1 | -- | GQ913667.1 | EU394722.1 | -- | -- | -- | -- | -- | -- | -- |
| *Liophidium torquatum* | -- | AY188062.1 | -- | AY187984.1 | DQ979984.1 | -- | -- | -- | -- | -- | -- | -- |
| *Liophidium vaillanti* | -- | GQ913674.1 | -- | GQ913668.1 | GQ913677.1 | -- | -- | -- | -- | -- | -- | -- |
| *Liopholidophis baderi* | -- | -- | -- | KC988269.1 | KC988264.1 | -- | -- | -- | -- | -- | -- | -- |
| *Liopholidophis dimorphus* | -- | DQ979966.1 | -- | DQ979973.1 | DQ979980.1 | -- | -- | -- | -- | -- | -- | -- |
| *Liopholidophis dolicocercus* | -- | DQ979968.1 | -- | DQ979975.1 | DQ979982.1 | -- | -- | -- | -- | -- | -- | -- |
| *Liopholidophis grandidieri* | -- | KC988277.1 | -- | KC988273.1 | KC988268.1 | -- | -- | -- | -- | -- | -- | -- |
| *Liopholidophis oligolepis* | -- | KC988274.1 | -- | KC988270.1 | KC988265.1 | -- | -- | -- | -- | -- | -- | -- |
| *Liopholidophis rhadinaea* | -- | KC988275.1 | -- | KC988271.1 | KC988266.1 | -- | -- | -- | -- | -- | -- | -- |
| *Liopholidophis sexlineatus* | FJ404174.1 | AY188063.1 | -- | AY187985.1 | DQ979985.1 | -- | FJ404373.1 | -- | -- | -- | -- | FJ404447.1 |
| *Liopholidophis varius* | -- | KC988276.1 | -- | KC988272.1 | KC988267.1 | -- | -- | -- | -- | -- | -- | -- |
| *Liotyphlops albirostris* | AF366693.1 | AF366762.1 | EU402650.1 | AF544727.1 | AF544672.1 | -- | -- | -- | -- | EU402853.1 | EU402853.1 | FJ433890.1 |
| *Loxocemus bicolor* | AF544755.1 | AF544828.1 | EU402651.1 | AY444035.1 | AY099993.1 | -- | -- | FJ434072.1 | -- | AY444061.1 | AY444061.1 | FJ433897.1 |
| *Lycodon alcalai* | -- | -- | -- | KC010303.1 | KC010344.1 | -- | -- | -- | -- | -- | -- | -- |
| *Lycodon aulicus* | HQ735417.1 | -- | -- | KC010305.1 | KC010346.1 | -- | -- | -- | -- | -- | -- | -- |
| *Lycodon bibonius* | -- | -- | -- | KC010309.1 | KC010351.1 | -- | -- | -- | -- | -- | -- | -- |
| *Lycodon butleri* | -- | -- | -- | KC010310.1 | KC010352.1 | -- | -- | -- | -- | -- | -- | -- |
| *Lycodon capucinus* | -- | -- | -- | KC010317.1 | KC010359.1 | -- | U49317.1 | -- | -- | -- | -- | -- |
| *Lycodon carinatus* | -- | KC347352.1 | -- | KC347392.1 | KC347486.1 | -- | -- | -- | -- | KC347430.1 | -- | -- |
| *Lycodon cavernicolus* | -- | -- | -- | -- | KJ607889.1 | -- | -- | -- | -- | -- | -- | -- |
| *Lycodon chrysoprateros* | -- | -- | -- | KC010318.1 | KC010360.1 | -- | -- | -- | -- | -- | -- | -- |
| *Lycodon dumerilii* | -- | -- | -- | KC010319.1 | KC010361.1 | -- | -- | -- | -- | -- | -- | -- |
| *Lycodon effraenis* | -- | -- | -- | KC010321.1 | KC010364.1 | -- | -- | -- | -- | -- | -- | -- |
| *Lycodon fasciatus* | -- | EU999215.1 | -- | -- | KC010365.1 | -- | -- | -- | -- | -- | -- | -- |
| *Lycodon flavozonatus* | -- | -- | -- | KF732934.1 | KF732927.1 | -- | KF732920.1 | -- | -- | -- | -- | -- |
| *Lycodon futsingensis* | -- | EU999210.1 | -- | -- | -- | -- | -- | -- | -- | -- | -- | -- |
| *Lycodon gongshan* | -- | -- | -- | KP901018.1 | KP901026.1 | -- | KP901033.1 | -- | KP901040.1 | -- | -- | -- |
| *Lycodon jara* | -- | -- | -- | KC010322.1 | KC010367.1 | -- | -- | -- | -- | -- | -- | -- |
| *Lycodon laoensis* | Z46455.1 | Z46485.1 | -- | KC010323.1 | KC010368.1 | -- | -- | -- | -- | -- | -- | -- |
| *Lycodon liuchengchaoi* | -- | -- | -- | KF732935.1 | KF732928.1 | -- | KF732921.1 | -- | -- | -- | -- | -- |
| *Lycodon muelleri* | -- | -- | -- | KC010326.1 | KC010373.1 | -- | -- | -- | -- | -- | -- | -- |
| *Lycodon multizonatus* | -- | -- | -- | KF732933.1 | KF732926.1 | -- | KF732919.1 | -- | -- | -- | -- | -- |
| *Lycodon osmanhilli* | -- | KC347364.1 | -- | KC347403.1 | -- | -- | KC347524.1 | -- | -- | KC347441.1 | -- | -- |
| *Lycodon paucifasciatus* | -- | EU999212.1 | -- | -- | -- | -- | -- | -- | -- | -- | -- | -- |
| *Lycodon rufozonatus* | AF233939.2 | HM439982.1 | JQ599018.1 | JF827695.1 | AF471063.1 | NC_024559.1 | KF732917.1 | -- | -- | -- | -- | -- |
| *Lycodon ruhstrati* | -- | EU999204.1 | -- | KF732932.1 | KF732925.1 | -- | KF732918.1 | -- | -- | -- | -- | -- |
| *Lycodon semicarinatus* | -- | AB008539 | -- | -- | NC_001945 | DQ902208.1 | NC_001945 | -- | -- | -- | -- | -- |
| *Lycodon* sp. | -- | -- | -- | KC010329.1 | KC010377.1 | -- | -- | -- | -- | -- | -- | -- |
| *Lycodon stormi* | -- | -- | -- | KC010331.1 | KC010380.1 | -- | -- | -- | -- | -- | -- | -- |
| *Lycodon subcinctus* | -- | -- | -- | KC010335.1 | KC010384.1 | -- | -- | -- | -- | -- | -- | -- |
| *Lycodon synaptor* | -- | -- | -- | KP901014.1 | KP901020.1 | -- | KP901028.1 | -- | KP901035.1 | -- | -- | -- |
| *Lycodon zawi* | -- | -- | -- | KC010336.1 | AF471040.1 | -- | -- | -- | -- | -- | -- | -- |
| *Lycodonomorphus inornatus* | FJ404167.1 | AY611891.1 | -- | HQ207093.1 | HQ207134.1 | -- | HQ207176.1 | -- | -- | -- | -- | FJ404440.1 |
| *Lycodonomorphus laevissimus* | -- | -- | -- | DQ486162.1 | DQ486338.1 | -- | DQ486314.1 | -- | -- | -- | -- | -- |
| *Lycodonomorphus rufulus* | FJ404175.1 | FJ404199.1 | -- | HQ207076.1 | HQ207111.1 | -- | HQ207153.1 | -- | -- | -- | -- | FJ404448.1 |
| *Lycodonomorphus whytii* | FJ404176.1 | FJ404200.1 | -- | FJ387201.1 | FJ404300.1 | -- | FJ404375.1 | -- | -- | -- | -- | FJ404449.1 |
| *Lycodryas citrinus* | FJ404191.1 | AY611865.1 | JQ073076.1 | AY611956.1 | AY612047.1 | -- | HE798413.1 | -- | -- | JQ073197.1 | JQ073197.1 | FJ404462.1 |
| *Lycodryas cococola* | -- | HE798439.1 | -- | HE798400.1 | HE798431.1 | -- | HE798421.1 | -- | -- | -- | -- | -- |
| *Lycodryas gaimardi* | -- | GU994844.1 | -- | GU994819.1 | GU994794.1 | -- | HE798412.1 | -- | -- | -- | -- | -- |
| *Lycodryas granuliceps* | -- | GU994848.1 | -- | GU994823.1 | GU994798.1 | -- | HE798410.1 | -- | -- | -- | -- | -- |
| *Lycodryas inopinae* | -- | GU994850.1 | -- | GU994825.1 | GU994800.1 | -- | HE798414.1 | -- | -- | -- | -- | -- |
| *Lycodryas inornatus* | -- | GU994859.1 | -- | GU994834.1 | GU994809.1 | -- | HE798415.1 | -- | -- | -- | -- | -- |
| *Lycodryas maculatus* |  | HE798438.1 | -- | HE798399.1 | AY612010.1 | -- | HE798420.1 | -- | -- | -- | -- | -- |
| *Lycodryas pseudogranuliceps* | -- | GU994858.1 | -- | GU994833.1 | GU994808.1 | -- | HE798408.1 | -- | -- | -- | -- | -- |
| *Lycodryas* sp. | -- | GU994840.1 | JQ073080.1 | GU994815.1 | -- | -- | HE798409.1 | -- | -- | JQ073201.1 | JQ073201.1 | -- |
| *Lycognathophis seychellensis* | -- | -- | -- | FJ387220.1 | -- | -- | -- | -- | -- | -- | -- | FJ404465.1 |
| *Lycophidion capense* | FJ404178.1 | AY611893.1 | EU402652.1 | AY611984.1 | DQ486344.1 | -- | DQ486320.1 | JN703031.1 | -- | EU402855.1 | -- | FJ404450.1 |
| *Lycophidion laterale* | FJ404179.1 | FJ404197.1 | -- | FJ387198.1 | FJ404297.1 | -- | FJ404377.1 | -- | -- | -- | -- | FJ404451.1 |
| *Lycophidion nigromaculatum* | FJ404180.1 | -- | -- | FJ404281.1 | FJ404298.1 | -- | -- | -- | -- | -- | -- | -- |
| *Lycophidion ornatum* | -- | -- | -- | AF471144.1 | AF471086.1 | -- | -- | -- | -- | -- | -- | -- |
| *Lygophis anomalus* | JQ598817.1 | JQ598879.1 | -- | -- | -- | -- | -- | -- | -- | -- | -- | -- |
| *Lygophis elegantissimus* | GQ457808.1 | GQ457748.1 | -- | GQ457868.1 | -- | -- | -- | -- | -- | -- | -- | -- |
| *Lygophis flavifrenatus* | JQ598818.1 | JQ598880.1 | -- | -- | -- | -- | -- | -- | -- | -- | -- | -- |
| *Lygophis lineatus* | -- | -- | DQ469795.1 | DQ469789.1 | -- | -- | -- | DQ469793.1 | -- | DQ469791.1 | -- | -- |
| *Lygophis meridionalis* | GQ457810.1 | GQ457750.1 | -- | GQ457870.1 | -- | -- | -- | -- | -- | -- | -- | -- |
| *Lygophis paucidens* | JQ598819.1 | -- | -- | JQ598987.1 | -- | -- | -- | -- | -- | -- | -- | -- |
| *Lytorhynchus diadema* | HQ658430.1 | HQ267794.1 | -- | DQ112079.1 | DQ112076.1 | -- | -- | -- | -- | -- | -- | -- |
| *Macrelaps microlepidotus* | FJ404137.1 | AY611810.1 | -- | AY611902.1 | AY611993.1 | -- | FJ404340.1 | -- | -- | -- | -- | FJ404412.1 |
| *Macropisthodon rudis* | -- | -- | -- | JQ687452.1 | JQ687434.1 | JQ687466.1 | JQ687427.1 | KJ685726.1 | KJ685566.1 | -- | -- | -- |
| *Macroprotodon abubakeri* | AY643297.1 | AY643338.1 | -- | -- | AY643380.1 | -- | -- | -- | -- | -- | -- | -- |
| *Macroprotodon brevis* | AY643293.1 | AY643334.1 | -- | -- | DQ907242.1 | DQ902270.1 | DQ902333.1 | -- | -- | -- | -- | -- |
| *Macroprotodon cucullatus* | AY643289.1 | AY188065.1 | -- | AF471145.1 | AF471087.1 | AY487025.1 | AY487064.1 | -- | -- | -- | -- | -- |
| *Macroprotodon mauritanicus* | AY643302.1 | AY643343.1 | -- | -- | AY643385.1 | -- | -- | -- | -- | -- | -- | -- |
| *Macrovipera lebetina* | EU624260.1 | EU624294.1 | -- | -- | AJ275713.1 | -- | DQ897729.1 | -- | -- | -- | -- | -- |
| *Macrovipera schweizeri* | EU624262.1 | AJ275768.1 | -- | -- | AJ275715.1 | -- | -- | -- | -- | -- | -- | -- |
| *Madagascarophis colubrinus* | -- | AY586193.1 | -- | AY586218.1 | AY586230.1 | -- | U49313.1 | -- | -- | -- | -- | -- |
| *Madagascarophis fuchsi* | -- |  | -- | KC514962.1 | KC514960.1 | -- | -- | -- | -- | -- | -- | -- |
| *Madagascarophis meridionalis* | FJ404181.1 | AY586212.1 | JQ073077.1 | AY586227.1 | AY586249.1 | -- | FJ404378.1 | -- | -- | JQ073198.1 | JQ073198.1 | FJ404452.1 |
| *Madagascarophis* sp. | -- | AY586217.1 | -- | AY586229.1 | AY586254.1 | -- | -- | -- | -- | -- | -- | -- |
| *Madatyphlops andasibensis* | -- | -- | GU902453.1 | -- | -- | -- | -- | GU902622.1 | -- | JQ073249.1 | GU902698.1 | -- |
| *Madatyphlops arenarius* | -- | -- | GU902455.1 | -- | -- | -- | -- | GU902624.1 | -- | -- | GU902699.1 | -- |
| *Magliophis exiguum* | FJ416694.1 | FJ416705.1 | -- | AF471117.1 | AF471071.1 | FJ416762.1 | FJ416798.1 | -- | -- | -- | -- | FJ416835.1 |
| *Magliophis stahli* | -- | -- | -- | -- | FJ416725.1 | FJ416763.1 | FJ416799.1 | -- | -- | -- | -- | FJ416836.1 |
| *Malayopython reticulatus* | Z46448.1 | EF545062.1 | FJ433969.1 | AF544675.1 | U69860.1 |  | -- | FJ434074.1 | EU624119.1 | EU624119.1 | EU624119.1 | FJ433899.1 |
| *Malayopython timoriensis* | EF545036.1 | EF545063.1 | -- | -- | EF545106.1 | -- | -- | -- | -- | -- | -- | -- |
| *Malayotyphlops luzonensis* | AF366694.1 | AF366763.1 | GU902393.1 | -- | -- | -- | -- | -- | -- | -- | GU902643.1 | -- |
| *Malayotyphlops ruber* | AF512728.1 | AF512728.1 | -- | -- | -- | -- | -- | -- | -- | -- | -- | -- |
| *Malpolon insignitus* | DQ451950.1 | -- | -- | -- | DQ451884.1 | -- | -- | -- | -- | -- | -- | -- |
| *Malpolon monspessulanus* | DQ451942.1 | AY188068.1 | -- | AY058936.1 | AY235721.1 | AY059011.1 | AY058989.1 | -- | -- | -- | -- | FJ404390.1 |
| *Manolepis putnami* | GU018151.1 | GU018171.1 | JQ599035.1 | GQ895820.1 | GQ895878.1 | -- | -- | -- | -- | -- | -- | -- |
| *Mastigodryas bifossatus* | HM565763.1 | HM582223.1 | -- | HQ157827.1 | -- | -- | -- | -- | -- | -- | -- | -- |
| *Mastigodryas boddaerti* | HM565764.1 | HM582224.1 | -- | GQ895811.1 | GQ895867.1 | -- | -- | -- | -- | -- | -- | -- |
| *Mastigodryas melanolomus* | -- | -- | -- | -- | GQ895868.1 | -- | -- | -- | -- | -- | -- | -- |
| *Melanophidium punctatum* | AY700993.1 | AY701024.1 | -- | -- | -- | -- | -- | -- | -- | -- | -- | -- |
| *Micrelaps bicoloratus* | -- | -- | -- | DQ486173.1 | DQ486349.1 | -- | -- | -- | -- | -- | -- | -- |
| *Micropechis ikaheka* | GQ397243.1 | FJ587207.1 | -- | EU366449.1 | EU547042.1 | -- | GQ397208.1 | -- | EU366435.1 | -- | -- | -- |
| *Micropisthodon ochraceus* | -- | AY188069.1 | -- | AY187991.1 | -- | -- | -- | -- | -- | -- | -- | -- |
| *Micruroides euryxanthus* | Z46433.1 | Z46483.1 | -- | EF137423.1 | EF137416.1 | -- | EF137408.1 | -- | -- | -- | -- | -- |
| *Micrurus albicinctus* | -- | -- | -- | -- | -- | -- | JF308714.1 | -- | -- | -- | -- | -- |
| *Micrurus altirostris* | -- | JQ627286.1 | -- | -- | -- | -- | AF228429.1 | -- | -- | -- | -- | -- |
| *Micrurus baliocoryphus* | -- | -- | -- | -- | -- | -- | AF228433.1 | -- | -- | -- | -- | -- |
| *Micrurus brasiliensis* | -- | -- | -- | -- | -- | -- | AF228427.1 | -- | -- | -- | -- | -- |
| *Micrurus corallinus* | -- | -- | -- | -- | -- | -- | JF308715.1 | -- | -- | -- | -- | -- |
| *Micrurus decoratus* | -- | -- | -- | -- | -- | -- | AF228441.1 | -- | -- | -- | -- | -- |
| *Micrurus diastema* | Z46454.1 | Z46484.1 | -- | -- | -- | -- | JF308710.1 | -- | -- | -- | -- | -- |
| *Micrurus dissoleucus* | -- | -- | -- | -- | -- | -- | JF308717.1 | -- | -- | -- | -- | -- |
| *Micrurus frontalis* | -- | -- | -- | -- | -- | -- | AF228425.1 | -- | -- | -- | -- | -- |
| *Micrurus fulvius* | U96805.1 | GU045453 | EU402653.1 | AY058935.1 | U69846.1 | AY059006.1 | GU045453 | EU390929.1 | -- | EU402856.1 |  | -- |
| *Micrurus hemprichii* | -- | -- | -- | -- | -- | -- | AF228442.1 | -- | -- | -- | -- | -- |
| *Micrurus ibiboboca* | -- | -- | -- | -- | -- | -- | AF228440.1 | -- | -- | -- | -- | -- |
| *Micrurus lemniscatus* | -- | -- | -- | -- | -- | -- | AF228438.1 | -- | -- | -- | -- | -- |
| *Micrurus mipartitus* | -- | -- | -- | -- | EF137414.1 | -- | EF137406.1 | -- | -- | -- | -- | -- |
| *Micrurus mosquitensis* | -- | -- | -- | -- | -- | -- | JF308712.1 | -- | -- | -- | -- | -- |
| *Micrurus narduccii* | -- | -- | -- | EF137420.1 | EF137412.1 | -- | EF137404.1 | -- | -- | -- | -- | -- |
| *Micrurus psyches* | -- | -- | -- | -- | -- | -- | JF308713.1 | -- | -- | -- | -- | -- |
| *Micrurus pyrrhocryptus* | -- | -- | -- | -- | -- | -- | JF308705.1 | -- | -- | -- | -- | -- |
| *Micrurus spixii* | -- | -- | -- | -- | -- | -- | AF228443.1 | -- | -- | -- | -- | -- |
| *Micrurus surinamensis* | AF544770.1 | AF544799.1 | FJ433991.1 | AF544708.1 | EF137415.1 | -- | JF308709.1 | FJ434092.1 | -- | -- | AY487411.1 | EF144102.1 |
| *Micrurus tener* | -- | -- | -- | -- | -- | -- | JF308711.1 | -- | -- | -- | -- | -- |
| *Mimophis mahfalensis* | AF544771.1 | AY188070.1 | JQ073081.1 | AF544688.1 | DQ486461.1 | -- | FJ404321.1 | -- | -- | JQ073202.1 | JQ073202.1 | FJ404391.1 |
| *Mitophis asbolepis* | GQ469210.1 | GQ469210.1 | GQ469174.1 | GQ469059.1 | GQ469086.1 | -- | -- | GQ469015.1 | -- | -- | GQ469037.1 | -- |
| *Mitophis leptipileptus* | GQ469197.1 | GQ469197.1 | GQ469185.1 | GQ469070.1 | GQ469085.1 | -- | -- | GQ469024.1 | -- | -- | GQ469047.1 | -- |
| *Mitophis pyrites* | GQ469194.1 | GQ469194.1 | GQ469170.1 | GQ469056.1 | GQ469079.1 | -- | -- | GQ469011.1 | -- | -- | GQ469033.1 | -- |
| *Mitophis* sp. | GQ469195.1 | GQ469195.1 | GQ469171.1 | GQ469057.1 | GQ469095.1 | -- | -- | GQ469012.1 | -- | -- | GQ469034.1 | -- |
| *Mixcoatlus barbouri* | HM363639.1 | HM363640.1 | -- | -- | HM363641.1 | -- | HM363642.1 | -- | -- | -- | -- | -- |
| *Mixcoatlus browni* | HM363650.1 | HM363651.1 | -- | -- | HM363652.1 | -- | HM363653.1 | -- | -- | -- | -- | -- |
| *Mixcoatlus melanurus* | AF057210.1 | AF057257.1 | -- | -- | AY223587.1 | -- | AY223634.1 | -- | -- | -- | -- | -- |
| *Montivipera albizona* | EU624265.1 | AJ275780.1 | -- | -- | AJ275727.1 | -- | EU624231.1 | -- | -- | -- | -- | -- |
| *Montivipera bornmuelleri* | -- | AJ275779.1 | -- | -- | AJ275726.1 | -- | -- | -- | -- | -- | -- | -- |
| *Montivipera latifii* | JN870191.1 | JN870199.1 | -- | -- | JN870205.1 | -- | -- | -- | -- | -- | -- | -- |
| *Montivipera raddei* | -- | AJ275784.1 | -- | -- | AJ275730.1 | -- | -- | -- | -- | -- | -- | -- |
| *Montivipera wagneri* | JN870188.1 | AJ275778.1 | -- | -- | AJ275725.1 | -- | JN870213.1 | -- | -- | -- | -- | -- |
| *Montivipera xanthina* | EU624268.1 | AJ275777.1 | -- | -- | AJ275724.1 | -- | EU624234.1 | -- | -- | -- | -- | -- |
| *Morelia bredli* | EF545016.1 | EF545043.1 | -- | -- | KJ666638.1 | -- | -- | -- | -- | -- | -- | -- |
| *Morelia carinata* | EF545017.1 | EF545044.1 | -- | -- | EF545095.1 | -- | -- | -- | -- | -- | -- | -- |
| *Morelia spilota* | EF545015.1 | EF545042.1 | AY988035.1 | -- | U69851.1 | -- | -- | AY988052.1 | -- | AY988069.1 | -- | -- |
| *Morelia viridis* | EF545022.1 | EF545048.1 | -- | -- | EF545098.1 | -- | -- | -- | EU366442.1 | -- | -- | -- |
| *Mussurana bicolor* | GQ457787.1 | GQ457729.1 | -- | GQ457849.1 | -- | -- | -- | -- | -- | -- | -- | -- |
| *Myriopholis adleri* | GQ469246.1 | GQ469246.1 | GQ469172.1 | GQ469058.1 | -- | -- | -- | GQ469013.1 | -- | -- | GQ469035.1 | -- |
| *Myriopholis algeriensis* | GQ469243.1 | GQ469243.1 | GQ469173.1 | -- | -- | -- | -- | GQ469014.1 | -- | -- | GQ469036.1 | -- |
| *Myriopholis blanfordi* | GQ469241.1 | GQ469241.1 | GQ469176.1 | GQ469061.1 | -- | -- | -- | -- | -- | -- | GQ469039.1 | -- |
| *Myriopholis boueti* | GQ469248.1 | GQ469248.1 | -- | GQ469062.1 | -- | -- | -- | GQ469017.1 | -- | -- | GQ469040.1 | -- |
| *Myriopholis longicauda* | GQ469244.1 | GQ469244.1 | GQ469186.1 | GQ469071.1 | -- | -- | -- | GQ469025.1 | -- | -- | GQ469048.1 | -- |
| *Myriopholis macrorhyncha* | GQ469245.1 | GQ469245.1 | GQ469187.1 | GQ469072.1 | -- | -- | -- | GQ469026.1 | -- | -- | GQ469049.1 | -- |
| *Myriopholis rouxestevae* | GQ469247.1 | GQ469247.1 | GQ469190.1 | GQ469075.1 | -- | -- | -- | GQ469029.1 | -- | -- | GQ469052.1 | -- |
| *Myron richardsonii* | EF395893.1 | EF395869.1 | -- | EF395941.1 | EF395918.1 | -- | -- | -- | -- | -- | -- | -- |
| *Myrrophis chinensis* | EF395878.1 | EF395854.1 | -- | EF395928.1 | EF395903.1 | -- | GU997183.1 | -- | -- | -- | -- | -- |
| *Naja anchietae* | -- | -- | -- | -- | GQ387114.1 | -- | GQ387087.1 | -- | -- | -- | -- | -- |
| *Naja annulata* | -- | AY188049.1 | -- | AY058925.1 | -- | AY058998.1 | AY058970.1 | -- | -- | -- | -- | -- |
| *Naja annulifera* | GQ359667.1 | GQ359753.1 | -- | -- | GQ359504.1 | -- | GQ387090.1 | -- | -- | -- | -- | -- |
| *Naja arabica* | GQ359663.1 | GQ359749.1 | -- | -- | GQ359500.1 | -- | GQ387074.1 | -- | -- | -- | -- | -- |
| *Naja ashei* | GQ359656.1 | GQ359742.1 | -- | -- | GQ359493.1 | -- | GQ359575.1 | -- | -- | -- | -- | -- |
| *Naja atra* | EU913475.1 | EU913475 | -- | -- | EU913475 | DQ302759.1 | EU913475 | -- | -- | HQ902537.1 | HQ902537.1 | -- |
| *Naja haje* | GQ359664.1 | GQ359747.1 | -- | -- | GQ359501.1 | -- | GQ359580.1 | -- | -- | -- | -- | -- |
| *Naja kaouthia* | JN687924.1 | GQ359757.1 | EU402654.1 | AY058938.1 | FR693728.1 | AY059008.1 | EU624209.1 | EU390930.1 | JF412633.1 | EU402857.1 | -- | -- |
| *Naja katiensis* | GQ359657.1 | GQ359743.1 | -- | -- | GQ359494.1 | -- | DQ897707.1 | -- | -- | -- | -- | -- |
| *Naja melanoleuca* | U96801.1 | JF357949.1 | -- | -- | FR693726.1 | -- | DQ897689.1 | -- | JF357955.1 | -- | -- | -- |
| *Naja mossambica* | GQ359658.1 | GQ359744.1 | -- | -- | GQ359495.1 | -- | DQ897727.1 | -- | -- | -- | -- | -- |
| *Naja multifasciata* | -- | -- | -- | AY058941.1 | -- | AY058996.1 | AY058985.1 | -- | -- | -- | -- | -- |
| *Naja naja* | DQ343648.1 | DQ343648 | -- | EU366445.1 | DQ343648 | DQ343648.1 | DQ343648 | -- | EU366432.1 | -- | -- | -- |
| *Naja nigricincta* | -- | -- | -- | -- | DQ897752.1 | -- | DQ897709.1 | -- | -- | -- | -- | -- |
| *Naja nigricollis* | EU624237.1 | GQ359754.1 | -- | -- | GQ359505.1 | -- | DQ897697.1 | -- | -- | -- | -- | -- |
| *Naja nivea* | EU624238.1 | GQ359755.1 | -- | AY058939.1 | FR693729.1 | AY059009.1 | AY058983.1 | -- | -- | -- | -- | -- |
| *Naja nubiae* | GQ359660.1 | GQ359746.1 | -- | -- | GQ359497.1 | -- | GQ359579.1 | -- | -- | -- | -- | -- |
| *Naja pallida* | GQ359659.1 | GQ359745.1 | -- | -- | FR693723.1 | -- | GQ359578.1 | -- | -- | -- | -- | -- |
| *Naja senegalensis* | -- | -- | -- | -- | GQ387109.1 | -- | GQ387081.1 | -- | -- | -- | -- | -- |
| *Naja siamensis* | JN687926.1 | JN687927.1 | -- | -- | AF155214.1 | -- | -- | -- | -- | -- | -- | -- |
| *Naja sputatrix* | -- | -- | -- | -- | DQ897734.1 | -- | DQ897691.1 | -- | -- | -- | -- | -- |
| *Naja sumatrana* | JN687928.1 | JN687929.1 | -- | -- | -- | -- | -- | -- | -- | -- | -- | -- |
| *Namibiana occidentalis* | GQ469251.1 | GQ469251.1 | GQ469189.1 | GQ469074.1 | -- | -- | -- | GQ469028.1 | -- | -- | GQ469051.1 | -- |
| *Natriciteres olivacea* | AF544772.1 | AF544801.1 | -- | AF471146.1 | AF471058.1 | -- | -- | -- | -- | -- | -- | -- |
| *Natrix maura* | AF402623.1 | -- | -- | -- | AY487690.1 | AY870614.1 | AY487785.1 | -- | -- | -- | -- | -- |
| *Natrix natrix* | AY122682.1 | -- | EU402655.1 | AF544697.1 | AY487749.1 | AY870630.1 | AY487794.1 | KF234022.1 | -- | EU402858.1 | -- | -- |
| *Natrix tessellata* | -- | -- | -- | -- | AY866531.1 | AY870641.1 | AY873734.1 | -- | -- | -- | -- | -- |
| *Nerodia clarkii* | -- | -- | -- | -- | KP765665.1 | -- | -- | -- | -- | -- | -- | -- |
| *Nerodia cyclopion* | AF402626.1 | -- | KF258598.1 | -- | AF402909.1 | AF384828.1 | KF258632.1 | KF234028.1 | -- | -- | -- | -- |
| *Nerodia erythrogaster* | AF402629.1 | -- | -- | JN090137.1 | AF402912.1 | AF420083.1 | AF420084.1 | -- | -- | -- | -- | -- |
| *Nerodia fasciata* | AF402627.1 | -- | KF258597.1 | -- | AF402910.1 | AY870612.1 | AY873705.1 | KF234027.1 | -- | -- | -- | -- |
| *Nerodia floridana* | AF402628.1 | -- | -- | -- | AF402911.1 | AF384830.1 | -- | -- | -- | -- | -- | -- |
| *Nerodia harteri* | AF402652.1 | -- | -- | -- | JQ743510.1 | AF384854.1 | -- | -- | -- | -- | -- | -- |
| *Nerodia paucimaculata* | -- | -- | -- | -- | JQ743512.1 | -- | -- | -- | -- | -- | -- | -- |
| *Nerodia rhombifer* | Z46452.1 | Z46481.1 | -- | -- | AF402915.1 | AF384834.1 | -- | -- | -- | -- | -- | -- |
| *Nerodia sipedon* | AF402630.1 | JF964960.1 | -- | -- | AF402913.1 | DQ915154.1 | JF964960 | -- | -- | -- | -- | -- |
| *Nerodia taxispilota* | AF402631.1 | -- | -- | -- | AF402914.1 | AF384833.1 | U49322.1 | -- | -- | -- | -- | -- |
| *Ninia atrata* | GQ457814.1 | JQ598882.1 | JQ599037.1 | GQ457874.1 | JQ598937.1 | -- | GQ334659.1 | GQ334683.1 | -- | -- | -- | -- |
| *Ninia sebae* | -- | -- | -- | GQ895821.1 | GQ895879.1 | -- | -- | -- | -- | -- | -- | -- |
| *Notechis scutatus* | U96802.1 | EU547180.1 | EU402656.1 | EU546944.1 | EU547082.1 | AY058994.1 | EU547034.1 | EU390932.1 | EU402859.1 | EU402859.1 | -- | -- |
| *Nothopsis rugosus* | GU018159.1 | GU018177.1 | -- | -- | -- | -- | -- | -- | -- | -- | -- | -- |
| *Oligodon arnensis* | KC347327.1 | KC347365.1 | -- | KC347404.1 | KC347464.1 | -- | KC347504.1 | -- | -- | KC347442.1 | -- | -- |
| *Oligodon barroni* | HM591523.1 | HM591523.1 | -- | -- | -- | -- | -- | -- | -- | -- | -- | -- |
| *Oligodon calamarius* | KC347328.1 | KC347366.1 | -- | KC347405.1 | KC347478.1 | -- | KC347511.1 | -- | -- | KC347443.1 | -- | -- |
| *Oligodon chinensis* | HM591525.1 | HM591525.1 | -- | KF732937.1 | KF732930.1 | -- | KF732923.1 | -- | -- | -- | -- | -- |
| *Oligodon cinereus* | HM591507.1 | HM591507.1 | -- | AF471101.1 | AF471033.1 | -- | -- | -- | -- | -- | -- | -- |
| *Oligodon cruentatus* | HM591517.1 | HM591517.1 | -- | -- | -- | -- | -- | -- | -- | -- | -- | -- |
| *Oligodon cyclurus* | HM591535.1 | HM591535.1 | -- | -- | -- | -- | -- | -- | -- | -- | -- | -- |
| *Oligodon formosanus* | HM591533.1 | HM591533.1 | -- | KF732936.1 | KF732929.1 | -- | KF732922.1 | -- | -- | -- | -- | -- |
| *Oligodon maculatus* | HM591511.1 | HM591511.1 | -- | KC010337.1 | KC010387.1 | -- | -- | -- | -- | -- | -- | -- |
| *Oligodon modestus* | HM591498.1 | HM591498.1 | -- | -- | -- | -- | -- | -- | -- | -- | -- | -- |
| *Oligodon ocellatus* | HM591534.1 | HM591534.1 | -- | -- | -- | -- | -- | -- | -- | -- | -- | -- |
| *Oligodon octolineatus* | HM591519.1 | HM591519.1 | -- | -- | -- | -- | U49316.1 | -- | -- | -- | -- | -- |
| *Oligodon planiceps* | HM591514.1 | HM591514.1 | -- | -- | -- | -- | -- | -- | -- | -- | -- | -- |
| *Oligodon* sp. | HM591528.1 | HM591528.1 | -- | -- | -- | -- | -- | -- | -- | -- | -- | -- |
| *Oligodon splendidus* | HM591509.1 | HM591509.1 | -- | -- | -- | -- | -- | -- | -- | -- | -- | -- |
| *Oligodon sublineatus* | KC347329.1 | KC347367.1 | -- | KC347406.1 | KC347465.1 | -- | KC347521.1 | -- | -- | KC347444.1 | -- | -- |
| *Oligodon taeniatus* | HM591522.1 | HM591522.1 | -- | -- | -- | -- | -- | -- | -- | -- | -- | -- |
| *Oligodon taeniolatus* | KC347330.1 | KC347368.1 | -- | KC347407.1 | KC347483.1 | -- | KC347505.1 | -- | -- | KC347445.1 | -- | -- |
| *Oligodon theobaldi* | HM591515.1 | HM591515.1 | -- | -- | -- | -- | -- | -- | -- | -- | -- | -- |
| *Oligodon torquatus* | HM591513.1 | HM591513.1 | -- | -- | -- | -- | -- | -- | -- | -- | -- | -- |
| *Oligodon venustus* | HM591500.1 | HM591500.1 | -- | -- | -- | -- | -- | -- | -- | -- | -- | -- |
| *Oocatochus rufodorsatus* | KC990020.1 | KC990020 | -- | DQ902081.1 | KC990020 | DQ902232.1 | DQ902301.1 | -- | -- | -- | -- | -- |
| *Opheodrys aestivus* | -- | -- | -- | AF471147.1 | AF471057.1 | -- | -- | -- | -- | -- | -- | -- |
| *Opheodrys vernalis* | -- | -- | -- | GQ927317.1 | GQ927322.1 | -- | -- | -- | -- | -- | -- | -- |
| *Ophiophagus hannah* | EU921899.1 | JN687931.1 | -- | AY058940.1 | EU921899 | AY059002.1 | EU921899 | AZIM01004301.1 | -- | -- | -- | -- |
| *Ophryacus undulatus* | AF057209.1 | AF057256.1 | -- | -- | AY223586.1 | -- | AY223633.1 | -- | -- | -- | -- | -- |
| *Opisthotropis cheni* | -- | -- | -- | JQ687441.1 | GQ281779.1 | JQ687457.1 | JQ687416.1 | KJ685757.1 | KJ685595.1 | -- | -- | -- |
| *Opisthotropis guangxiensis* | -- | -- | -- | JQ687447.1 | GQ281776.1 | JQ687462.1 | JQ687422.1 | -- | -- | -- | -- | -- |
| *Opisthotropis lateralis* | -- | -- | -- | JQ687445.1 | GQ281782.1 | JQ687461.1 | JQ687420.1 | -- | -- | -- | -- | -- |
| *Opisthotropis latouchii* | -- | -- | -- | JQ687446.1 | GQ281783.1 | -- | JQ687421.1 | -- | -- | -- | -- | -- |
| *Oreocalamus hanitschi* | -- | -- | -- | -- | -- | -- | U49306.1 | -- | -- | -- | -- | -- |
| *Oreocryptophis porphyraceus* | GQ181130.1 | GQ181130 | -- | DQ902076.1 | KF669255.1 | DQ902226.1 | GQ181130 | -- | -- | -- | -- | -- |
| *Orientocoluber spinalis* | AY541508.1 | AY376773.1 | -- | AY486948.1 | AY486924.1 | AY487017.1 | AY487056.1 | -- | -- | -- | -- | -- |
| *Orthriophis cantoris* | AY122769.1 | -- | -- | DQ902095.1 | DQ902135.1 | DQ902246.1 | DQ902315.1 | -- | -- | -- | -- | -- |
| *Orthriophis hodgsoni* | AY122778.1 | -- | -- | DQ902096.1 | DQ902136.1 | DQ902247.1 | DQ902318.1 | -- | -- | -- | -- | -- |
| *Orthriophis moellendorffi* | AY122787.1 | -- | -- | DQ902074.1 | DQ902116.1 | DQ902223.1 | DQ902295.1 | -- | -- | -- | -- | -- |
| *Orthriophis taeniurus* | AF233940.2 | HM439981.1 | -- | DQ902085.1 | EF076709.1 | -- | DQ902305.1 | -- | -- | -- | -- | -- |
| *Ovophis monticola* | HQ325249.1 | HQ325065.1 | -- | -- | HQ325126.1 | -- | HQ325187.1 | -- | -- | -- | -- | -- |
| *Ovophis okinavensis* | DQ305418.1 | AB175670 | -- | -- | AB175670 | AB175670.1 | AB175670 | -- | -- | -- | -- | -- |
| *Ovophis tonkinensis* | HQ325252.1 | HQ325070.1 | -- | -- | HQ325130.1 | -- | HQ325191.1 | -- | -- | -- | -- | -- |
| *Ovophis zayuensis* | HQ325304.1 | HQ325089.1 | -- | -- | HQ325150.1 | -- | HQ325208.1 | -- | -- | -- | -- | -- |
| *Oxybelis aeneus* | HM565765.1 | HM582225.1 | -- | AF471148.1 | AF471056.1 | -- | -- | -- | -- | -- | -- | -- |
| *Oxybelis fulgidus* | AF158432.1 | HM582226.1 | -- | HQ157830.1 | -- | -- | -- | -- | -- | -- | -- | -- |
| *Oxyrhabdium leporinum* | -- | -- | -- | DQ112081.1 | AF471029.1 | -- | -- | -- | -- | -- | -- | FJ404466.1 |
| *Oxyrhopus clathratus* | GQ457815.1 | GQ457754.1 | -- | GQ457875.1 | -- | -- | -- | -- | -- | -- | -- | -- |
| *Oxyrhopus formosus* | AF158411.1 | AF158482.1 | -- | -- | -- | -- | -- | -- | -- | -- | -- | -- |
| *Oxyrhopus guibei* | -- | JQ627289.1 | JQ599038.1 | JQ598989.1 | JQ598938.1 | -- | -- | -- | -- | -- | -- | -- |
| *Oxyrhopus melanogenys* | AF158422.1 | AF158489.1 | -- | JQ598990.1 | -- | -- | -- | -- | -- | -- | -- | -- |
| *Oxyrhopus petolarius* | GU018150.1 | GU018170.1 | -- | -- | GQ334554.1 | -- | -- | GQ334684.1 | -- | -- | -- | -- |
| *Oxyrhopus rhombifer* | GQ457816.1 | GQ457755.1 | -- | GQ457876.1 | -- | -- | -- | -- | -- | -- | -- | -- |
| *Oxyrhopus trigeminus* | JQ598824.1 | JQ598884.1 | -- | -- | JQ598939.1 | -- | -- | -- | -- | -- | -- | -- |
| *Oxyuranus microlepidotus* | EU547099.1 | -- | -- | EU366450.1 | EU547050.1 | -- | EF210823.1 | -- | EU366439.1 | -- | -- | -- |
| *Oxyuranus scutellatus* | EU547100.1 | EU547149.1 | -- | EU546916.1 | EU547051.1 | -- | EF210827.1 | -- | EU546877.1 | -- | -- | -- |
| *Oxyuranus temporalis* | -- | -- | -- | -- | -- | -- | JN834014.1 | -- | -- | -- | -- | -- |
| *Pantherophis alleghaniensis* | -- | -- | -- | FJ627793.1 | AF283644.1 | -- | -- | -- | -- | -- | -- | -- |
| *Pantherophis bairdi* | AY122812.1 | -- | -- | DQ902061.1 | GU073395.1 | DQ902209.1 | AF138755.1 | -- | -- | -- | -- | -- |
| *Pantherophis emoryi* | -- | -- | -- | FJ627791.1 | AF337173.1 | FJ627839.1 | -- | -- | -- | -- | -- | -- |
| *Pantherophis guttatus* | AM236349.1 | AM236349.1 | -- | DQ902070.1 | AM236349.1 | DQ902218.1 | AM236349.1 | -- | -- | -- | -- | -- |
| *Pantherophis obsoletus* | AY122843.1 | Z46493.1 | -- | AF471140.1 | DQ538339.1 | DQ902224.1 | DQ902296.1 | -- | -- | -- | -- | -- |
| *Pantherophis ramspotti* | -- | -- | -- | -- | FJ267684.1 | -- | -- | -- | -- | -- | -- | -- |
| *Pantherophis slowinskii* | DQ523162.1 | DQ523162 | -- | FJ627792.1 | DQ523162 | DQ523162.1 | DQ523162 | -- | -- | -- | -- | -- |
| *Pantherophis spiloides* | -- | -- | -- | -- | AF283643.1 | FJ627835.1 | FJ627849.1 | -- | -- | -- | -- | -- |
| *Pantherophis vulpinus* | AY122817.1 | -- | -- | DQ902089.1 | FJ267660.1 | DQ902238.1 | DQ902306.1 | -- | -- | -- | -- | -- |
| *Parahydrophis mertoni* | -- | DQ234048.1 | -- | FJ587177.1 | KC014451.1 | -- | FJ593201.1 | -- | FJ587099.1 | -- | -- | -- |
| *Paraphimophis rusticus* | JQ598802.1 | JQ598864.1 | -- | JQ598974.1 | JQ598923.1 | -- | -- | -- | -- | -- | -- | -- |
| *Parastenophis betsileanus* | FJ404190.1 | GU994852.1 | -- | GU994827.1 | GU994802.1 | -- | FJ404387.1 | -- | -- | -- | -- | FJ404461.1 |
| *Parasuta monachus* | EU547116.1 | EU547165.1 | -- | EU546929.1 | EU547067.1 | -- | EU547019.1 | -- | EU546890.1 | -- | -- | -- |
| *Parasuta spectabilis* | EU547114.1 | EU547163.1 | -- | EU546928.1 | EU547065.1 | -- | EU547017.1 | -- | EU546889.1 | -- | -- | -- |
| *Pareas atayal* | -- | -- | -- | KJ642198.1 | KJ642114.1 | -- | -- | -- | -- | -- | -- | -- |
| *Pareas boulengeri* | -- | -- | -- | JF827707.1 | JF827681.1 | -- | JF827658.1 | -- | -- | -- | -- | -- |
| *Pareas carinatus* | AF544773.1 | AF544802.1 | FJ433985.1 | JF827702.1 | JF827677.1 | -- | JF827653.1 | FJ434086.1 | -- | -- | -- | EF144096.1 |
| *Pareas formosensis* | -- | -- | -- | JF827718.1 | JF827692.1 | -- | JF827669.1 | -- | -- | -- | -- | -- |
| *Pareas hamptoni* | -- | -- | EU402657.1 | JF827703.1 | AY425809.1 | -- | JF827654.1 | EU390933.1 | -- | EU402860.1 | -- | -- |
| *Pareas iwasakii* | -- | -- | -- | KJ642207.1 | KJ642156.1 | -- | -- | -- | -- | -- | -- | -- |
| *Pareas komaii* | -- | -- | -- | KJ642210.1 | KJ642161.1 | -- | -- | -- | -- | -- | -- | -- |
| *Pareas margaritophorus* | -- | -- | -- | JF827698.1 | JF827675.1 | -- | -- | -- | -- | -- | -- | -- |
| *Pareas monticola* | -- | -- | -- | JF827715.1 | JF827689.1 | -- | JF827666.1 | -- | -- | -- | -- | -- |
| *Pareas nuchalis* | -- | -- | -- | -- | -- | -- | U49311.1 | -- | -- | -- | -- | -- |
| *Pareas stanleyi* | -- | -- | -- | JN230703.1 | JN230704.1 | -- | JN230705.1 | -- | -- | -- | -- | -- |
| *Paroplocephalus atriceps* | EU547129.1 | EU547178.1 | -- | EU546942.1 | EU547080.1 | -- | EU547032.1 | -- | EU546903.1 | -- | -- | -- |
| *Phalotris bilineatus* | JQ598827.1 | JQ598887.1 | -- | -- | JQ598943.1 | -- | -- | -- | -- | -- | -- | -- |
| *Phalotris lativittatus* | JQ598825.1 | JQ598885.1 | -- | JQ598991.1 | -- | -- | -- | -- | -- | -- | -- | -- |
| *Phalotris lemniscatus* | GQ457817.1 | GQ457756.1 | JQ599039.1 | GQ457877.1 | JQ598941.1 | -- | -- | -- | -- | -- | -- | -- |
| *Phalotris mertensi* | JQ598826.1 | JQ598886.1 |  |  |  |  |  |  |  |  | -- |  |
| *Phalotris nasutus* | GQ457818.1 | GQ457757.1 | -- | GQ895822.1 | GQ895880.1 | -- | -- | -- | -- | -- | -- | -- |
| *Philodryas aestiva* | GQ457819.1 | GQ457758.1 | -- | GQ457879.1 | -- | -- | -- | -- | -- | -- | -- | -- |
| *Philodryas agassizii* | GQ457823.1 | GQ457762.1 | -- | GQ457883.1 | GQ895883.1 | -- | -- | -- | -- | -- | -- | -- |
| *Philodryas argentea* | AF158413.1 | GQ457780.1 | JQ599040.1 | GQ457899.1 | JQ598944.1 | -- | -- | -- | -- | -- | -- | -- |
| *Philodryas baroni* | AF158469.1 | AF158534.1 | -- | -- | AF236812.1 | -- | -- | -- | -- | -- | -- | -- |
| *Philodryas chamissonis* | -- | -- | -- | -- | -- | -- | HM639951.1 | -- | -- | -- | -- | -- |
| *Philodryas georgeboulengeri* | -- | -- | -- | GQ895838.1 | AF471053.1 | -- | -- | -- | -- | -- | -- | -- |
| *Philodryas mattogrossensis* | GQ457820.1 | GQ457759.1 | -- | GQ457880.1 | -- | -- | -- | -- | -- | -- | -- | -- |
| *Philodryas nattereri* | JQ598829.1 | JQ598889.1 | -- | JQ598992.1 | AF236806.1 | -- | -- | -- | -- | -- | -- | -- |
| *Philodryas olfersii* | AF158417.1 | AF158484.1 | JQ599041.1 | JQ598993.1 | JQ598945.1 | -- | -- | -- | -- | -- | -- | -- |
| *Philodryas patagoniensis* | GQ457821.1 | JQ627296.1 | -- | GQ457881.1 | -- | -- | -- | -- | -- | -- | -- | -- |
| *Philodryas psammophidea* | GU018149.1 | GU018168.1 | -- | -- | -- | -- | -- | -- | -- | -- | -- | -- |
| *Philodryas trilineata* | -- | -- | -- | -- | -- | -- | HM639963.1 | -- | -- | -- | -- | -- |
| *Philodryas viridissima* | AF158419.1 | AF158474.1 | -- | -- | AF236807.1 | -- | -- | -- | -- | -- | -- | -- |
| *Philothamnus angolensis* | -- | AY611886.1 | -- | -- | AY612068.1 | -- | -- | -- | -- | -- | -- | -- |
| *Philothamnus carinatus* | -- | AY611870.1 | -- | -- | AY612052.1 | -- | -- | -- | -- | -- | -- | -- |
| *Philothamnus girardi* | -- | FJ913475.1 | -- | -- | FJ913495.1 | -- | -- | -- | -- | -- | -- | -- |
| *Philothamnus heterodermus* | -- | AY611856.1 | -- | AF471149.1 | AY612038.1 | -- | -- | -- | -- | -- | -- | -- |
| *Philothamnus hoplogaster* | -- | FJ913484.1 | -- | -- | FJ913496.1 | -- | -- | -- | -- | -- | -- | -- |
| *Philothamnus natalensis* | -- | AY611887.1 | -- | -- | AY612069.1 | -- | -- | -- | -- | -- | -- | -- |
| *Philothamnus nitidus* | -- | AY611871.1 | -- | -- | AY612053.1 | -- | -- | -- | -- | -- | -- | -- |
| *Philothamnus semivariegatus* | -- | AY611889.1 | -- | -- | AY612071.1 | -- | -- | -- | -- | -- | -- | -- |
| *Philothamnus thomensis* | -- | FJ913477.1 | -- | -- | FJ913490.1 | -- | -- | -- | -- | -- | -- | -- |
| *Phimophis guerini* | GQ457822.1 | GQ457761.1 | -- | GQ457882.1 | -- | -- | -- | -- | -- | -- | -- | -- |
| *Phisalixella tulearensis* | -- | GU994837.1 | -- | GU994812.1 | GU994787.1 | -- | -- | -- | -- | -- | -- | -- |
| *Phisalixella variabilis* | -- | GU994839.1 | -- | GU994814.1 | GU994788.1 | -- | -- | -- | -- | -- | -- | -- |
| *Phrynonax poecilonotus* | -- | -- | -- | KF669705.1 | KF669671.1 | -- | KF669687.1 | -- | -- | -- | -- | -- |
| *Phrynonax polylepis* | -- | -- | -- | KF669710.1 | KF669676.1 | -- | KF669693.1 | -- | -- | -- | -- | -- |
| *Phyllorhynchus browni* | -- | -- | -- | KP765644.1 | KP765666.1 | -- | -- | -- | -- | -- | -- | -- |
| *Phyllorhynchus decurtatus* | HM591497.1 | HM591497.1 | FJ434004.1 | AF544728.1 | AF471083.1 | -- | -- | FJ434105.1 | -- | -- | AY487385.1 | EF144115.1 |
| *Phytolopsis punctata* | EF395887.1 | EF395863.1 | -- | EF395935.1 | EF395912.1 | -- | -- | -- | -- | -- | -- | -- |
| *Pituophis catenifer* | -- | -- | -- | FJ627790.1 | AF337112.1 | FJ627842.1 | AF141106.1 | -- | -- | -- | -- | -- |
| *Pituophis deppei* | AY122827.1 | -- | -- | FJ627801.1 | FJ627818.1 | FJ627848.1 | JF308315.1 | -- | -- | -- | -- | -- |
| *Pituophis lineaticollis* | AF512746.1 | AF512746.1 | -- | FJ627804.1 | -- | FJ627841.1 | JF308340.1 | -- | -- | -- | -- | -- |
| *Pituophis melanoleucus* | AY122824.1 | -- | -- | FJ627797.1 | AF337100.1 | DQ902244.1 | EU272828.1 | -- | -- | -- | -- | -- |
| *Pituophis ruthveni* | FJ623966.1 | -- | -- | DQ902092.1 | AF337111.1 | -- | AF141121.1 | -- | -- | -- | -- | -- |
| *Pituophis vertebralis* | FJ623965.1 | -- | -- | FJ627789.1 | FJ627819.1 | -- | AF141126.2 | -- | -- | -- | -- | -- |
| *Plagiopholis styani* | -- | -- | -- | EU496916.1 | EU496918.1 | -- | -- | -- | -- | -- | -- | -- |
| *Platyceps collaris* | AY039157.1 |  | -- | AY486946.1 | AY486922.1 | AY487014.1 | AY487053.1 | -- | -- | -- | -- | -- |
| *Platyceps elegantissimus* | AY039147.1 | -- | -- | -- | -- | -- | -- | -- | -- | -- | -- | -- |
| *Platyceps florulentus* | AY039130.1 | -- | -- | AY486939.1 | AY486915.1 | AY487004.1 | AY487043.1 | -- | -- | -- | -- | -- |
| *Platyceps karelini* | AY647232.1 | AY188082.1 | -- | AY486942.1 | AY486918.1 | AY487008.1 | AY487052.1 | -- | -- | -- | -- | -- |
| *Platyceps najadum* | AY039141.1 | -- | -- | AY486936.1 | AY486912.1 | AY487009.1 | AY487038.1 | -- | -- | -- | -- | -- |
| *Platyceps rhodorachis* | AY039154.1 | -- | -- | AY486945.1 | AY486921.1 | AY487012.1 | AY487051.1 | -- | -- | -- | -- | -- |
| *Platyceps variabilis* | AY647234.1 | -- | -- | -- | -- | -- | -- | -- | -- | -- | -- | -- |
| *Platyceps ventromaculatus* | AY039134.1 | -- | -- | -- | -- | -- | -- | -- | -- | -- | -- | -- |
| *Pliocercus elapoides* | -- | -- | -- | GQ895824.1 | GQ895882.1 | -- | -- | -- | -- | -- | -- | -- |
| *Polemon acanthias* | FJ404138.1 | AY611848.1 | -- | AY611940.1 | AY612031.1 | -- | FJ404341.1 | -- | -- | -- | -- | FJ404413.1 |
| *Polemon collaris* | FJ404139.1 | AY611846.1 | -- | AY611938.1 | AY612029.1 | -- | FJ404342.1 | -- | -- | -- | -- | FJ404414.1 |
| *Polemon notatus* | FJ404140.1 | AY611847.1 | -- | AY611939.1 | AY612030.1 | -- | FJ404343.1 | -- | -- | -- | -- | FJ404415.1 |
| *Porthidium arcosae* | EU624241.1 | GQ372871.1 | -- | -- | -- | -- | AF292613.1 | -- | -- | -- | -- | -- |
| *Porthidium dunni* | AY223654.1 | AY223667.1 | -- | -- | AY223581.1 | -- | AY223630.1 | -- | -- | -- | -- | -- |
| *Porthidium hespere* | -- | -- | -- | -- | EU017534.1 | -- | EU016099.1 | -- | -- | -- | -- | -- |
| *Porthidium lansbergii* | AY223655.1 | AY223668.1 | -- | -- | AY223582.1 | -- | AY223631.1 | -- | -- | -- | -- | -- |
| *Porthidium nasutum* | AF057204.1 | AF057251.1 | -- | -- | DQ061210.1 | -- | DQ061235.1 | -- | -- | -- | -- | -- |
| *Porthidium ophryomegas* | AF057205.1 | AF057252.1 | -- | -- | DQ061216.1 | -- | DQ061241.1 | -- | -- | -- | -- | -- |
| *Porthidium porrasi* | DQ305421.1 | DQ305444.1 | -- | -- | DQ061214.1 | -- | DQ061239.1 | -- | -- | -- | -- | -- |
| *Porthidium yucatanicum* | JN870189.1 | JN870198.1 | -- | -- | DQ061215.1 | -- | DQ061244.1 | -- | -- | -- | -- | -- |
| *Proatheris superciliaris* | EU624263.1 | EU624296.1 | -- | -- | AJ275685.1 | -- | EU624230.1 | -- | -- | -- | -- | -- |
| *Prosymna greigerti* | -- | JF340124.1 | -- | -- | -- | -- | -- | -- | -- | -- | -- | -- |
| *Prosymna janii* | FJ404193.1 | FJ404222.1 | -- | FJ404293.1 | FJ404319.1 | -- | FJ404389.1 | -- | -- | -- | -- | FJ404464.1 |
| *Prosymna meleagris* | -- | JF340123.1 | -- | -- | -- | -- | -- | -- | -- | -- | -- | -- |
| *Prosymna ruspolii* | -- | -- | -- | DQ486171.1 | DQ486347.1 | -- | DQ486323.1 | -- | -- | -- | -- | -- |
| *Prosymna visseri* | FJ404192.1 | AY188072.1 | -- | AY187994.1 | AY188033.1 | -- | -- | -- | -- | -- | -- | FJ404463.1 |
| *Protobothrops cornutus* | AY294276.1 | FR695502.1 | -- | -- | FR695490.1 | KF110978.1 | AY294262.1 | -- | -- | -- | -- | -- |
| *Protobothrops dabieshanensis* | KF003004.1 | KF003004.1 | -- | -- | KF003004.1 | KF003004.1 | KF003004.1 | -- | -- | -- | -- | -- |
| *Protobothrops elegans* | AF057201.1 | AF057248.1 | -- | -- | AY223575.1 | -- | U41893.1 | -- | -- | -- | -- | -- |
| *Protobothrops flavoviridis* | AY352792.1 | AF057247.1 | -- | -- | AY223574.1 | -- | AY352826.1 | -- | -- | -- | -- | -- |
| *Protobothrops jerdonii* | AY763178.1 | KF170931.1 | -- | -- | HM567524.1 | NC_021402.1 | HM567456.1 | -- | -- | -- | -- | -- |
| *Protobothrops kaulbacki* | DQ666056.1 | DQ666055.1 | -- | -- | -- | -- | DQ666057.1 | -- | -- | -- | -- | -- |
| *Protobothrops mangshanensis* | AY352787.1 | AY352726.1 | -- | JQ687524.1 | HM567537.1 | NC_026052.1 | HM567469.1 | -- | -- | -- | -- | -- |
| *Protobothrops maolanensis* | JN799405.1 | JN799400.1 | -- | -- | JN799401.1 | KF039900.1 | JN799409.1 | -- | -- | -- | -- | -- |
| *Protobothrops mucrosquamatus* | KC438281.1 | KC438281 | -- | -- | KC438281 | KC438281.1 | KC438281 | -- | -- | -- | -- | -- |
| *Protobothrops sieversorum* | AY352782.1 | AY352721.1 | -- | -- | AY352753.1 | -- | AY352816.1 | -- | -- | -- | -- | -- |
| *Protobothrops tokarensis* | Z46446.1 | AF057249.1 | -- | -- | AY223576.1 | -- | AY223628.1 | -- | -- | -- | -- | -- |
| *Protobothrops xiangchengensis* | KF460436.1 | KF460436 | -- | -- | KF460436 | KF460436.1 | HM567470.1 | -- | -- | -- | -- | -- |
| *Psammodynastes pictus* | FJ404186.1 | FJ404221.1 | -- | FJ387218.1 | FJ404318.1 | -- | FJ404383.1 | -- | -- | -- | -- | FJ404457.1 |
| *Psammodynastes pulverulentus* | AF544784.1 | AF544813.1 | -- | AF471157.1 | AF471031.1 | -- | -- | -- | -- | -- | -- | -- |
| *Psammophis aegyptius* | -- | -- | -- | -- | -- | -- | EF128025.1 | -- | -- | -- | -- | -- |
| *Psammophis angolensis* | -- | -- | -- | DQ486189.1 | DQ486410.1 | -- | DQ486248.1 | -- | -- | -- | -- | -- |
| *Psammophis biseriatus* | -- | -- | -- | -- | DQ486448.1 | -- | DQ486284.1 | -- | -- | -- | -- | -- |
| *Psammophis brevirostris* | -- | -- | -- | -- | DQ486395.1 | -- | DQ486234.1 | -- | -- | -- | -- | -- |
| *Psammophis condanarus* | Z46450.1 | Z46479.1 | -- | AF471104.1 | AF471075.1 | AY058991.1 | AY058987.1 | -- | -- | -- | -- | -- |
| *Psammophis crucifer* | -- | -- | -- | DQ486188.1 | DQ486397.1 | -- | DQ486236.1 | -- | -- | -- | -- | -- |
| *Psammophis elegans* | -- | -- | -- | -- | -- | -- | EU526862.1 | -- | -- | -- | -- | -- |
| *Psammophis jallae* | -- | -- | -- | -- | DQ486409.1 | -- | DQ486247.1 | -- | -- | -- | -- | -- |
| *Psammophis leightoni* | -- | -- | -- | DQ486197.1 | DQ486467.1 | -- | DQ486303.1 | -- | -- | -- | -- | -- |
| *Psammophis leopardinus* | -- | -- | -- | -- | DQ486456.1 | -- | DQ486292.1 | -- | -- | -- | -- | -- |
| *Psammophis lineatus* | FJ404152.1 | FJ404216.1 | -- | FJ404256.1 | FJ404313.1 | -- | FJ404354.1 | -- | -- | -- | -- | FJ404426.1 |
| *Psammophis lineolatus* | FJ404117.1 | AY188073.1 | -- | DQ486195.1 | DQ486450.1 | -- | DQ486286.1 | -- | -- | -- | -- | FJ404392.1 |
| *Psammophis mossambicus* | FJ404118.1 | FJ404217.1 | -- | DQ486185.1 | DQ486383.1 | -- | DQ486222.1 | -- | -- | -- | -- | FJ404393.1 |
| *Psammophis namibensis* | -- | -- | -- | -- | DQ486455.1 | -- | DQ486291.1 | -- | -- | -- | -- | -- |
| *Psammophis notostictus* | -- | -- | -- | DQ486182.1 | DQ486366.1 | -- | DQ486205.1 | -- | -- | -- | -- | -- |
| *Psammophis orientalis* | FJ404119.1 | FJ404218.1 | -- | FJ404225.1 | DQ486390.1 | -- | DQ486229.1 | -- | -- | -- | -- | FJ404394.1 |
| *Psammophis phillipsii* | FJ404120.1 | AY611879.1 | -- | AY611970.1 | AY612061.1 | -- | DQ486290.1 | -- | -- | -- | -- | FJ404395.1 |
| *Psammophis praeornatus* | FJ404153.1 | -- | -- | FJ387216.1 | -- | -- | FJ404355.1 | -- | -- | -- | -- | FJ404427.1 |
| *Psammophis punctulatus* | -- | -- | -- | DQ486186.1 | DQ486387.1 | -- | DQ486226.1 | -- | -- | -- | -- | -- |
| *Psammophis rukwae* | -- | -- | -- | -- | DQ486452.1 | -- | DQ486279.1 | -- | -- | -- | -- | -- |
| *Psammophis schokari* | DQ451926.1 | AY611852.1 | -- | AY611943.1 | AY612034.1 | -- | EF128005.1 | -- | -- | -- | -- | FJ404396.1 |
| *Psammophis sibilans* | FJ404122.1 | FJ404219.1 | -- | FJ404228.1 | FJ404316.1 | -- | EF128028.1 | -- | -- | -- | -- | FJ404397.1 |
| *Psammophis* sp. 1 | -- | -- | -- | -- | DQ486419.1 | -- | DQ486256.1 | -- | -- | -- | -- | -- |
| *Psammophis* sp. 2 | -- | -- | JQ73082.1 | -- | -- | -- | -- | -- | -- | JQ073203.1 | JQ073203.1 | -- |
| *Psammophis* sp. 3 | FJ404123.1 | FJ404220.1 | -- | FJ387217.1 | FJ404317.1 | -- | FJ404326.1 | -- | -- | -- | -- | -- |
| *Psammophis subtaeniatus* | -- | -- | -- | -- | DQ486408.1 | -- | DQ486253.1 | -- | -- | -- | -- | -- |
| *Psammophis sudanensis* | -- | -- | -- | DQ486184.1 | DQ486444.1 | -- | DQ486280.1 | -- | -- | -- | -- | -- |
| *Psammophis tanganicus* | -- | -- | -- | DQ486183.1 | DQ486378.1 | -- | DQ486217.1 | -- | -- | -- | -- | -- |
| *Psammophis trigrammus* | -- | -- | -- | DQ486196.1 | DQ486458.1 | -- | DQ486294.1 | -- | -- | -- | -- | -- |
| *Psammophylax acutus* | -- | -- | -- | DQ486192.1 | DQ486425.1 | -- | DQ486262.1 | -- | -- | -- | -- | -- |
| *Psammophylax rhombeatus* | FJ404124.1 | FJ404215.1 | -- | DQ486166.1 | DQ486342.1 | -- | DQ486318.1 | -- | -- | -- | -- | FJ404399.1 |
| *Psammophylax tritaeniatus* | -- | -- | -- | DQ486190.1 | DQ486414.1 | -- | DQ486252.1 | -- | -- | -- | -- | -- |
| *Psammophylax variabilis* | AF544774.1 | AY611864.1 | FJ433996.1 | DQ486193.1 | AY612046.1 | -- | DQ486274.1 | FJ434097.1 | -- | -- | AY487380.1 | EF144107.1 |
| *Pseudalsophis dorsalis* | JQ598832.1 | JQ598892.1 | JQ599042.1 | JQ598994.1 | JQ598946.1 | -- | -- | -- | -- | -- | -- | -- |
| *Pseudalsophis elegans* | AF158401.1 | AF158470.1 | -- | -- | -- | -- | -- | -- | -- | -- | -- | -- |
| *Pseudaspis cana* | FJ404187.1 | AY611898.1 | -- | DQ486167.1 | AY612080.1 | AY058992.1 | DQ486319.1 | -- | -- | -- | -- | FJ404458.1 |
| *Pseudechis australis* | AJ749361.1 | EU547144.1 | -- | EU546912.1 | EU547046.1 | -- | AY340174.1 | -- | EU546873.1 | -- | -- | -- |
| *Pseudechis butleri* | AJ749360.1 | AJ749372.1 | -- | -- | AY340151.1 | -- | AY340180.1 | -- | -- | -- | -- | -- |
| *Pseudechis colletti* | AJ749353.1 | AJ749378.1 | -- | -- | AY340142.1 | -- | AY340171.1 | -- | -- | -- | -- | -- |
| *Pseudechis guttatus* | AJ749352.1 | AJ749371.1 | -- | -- | AY340143.1 | -- | AY340172.1 | -- | -- | -- | -- | -- |
| *Pseudechis papuanus* | AJ749354.1 | AJ749373.1 | -- | -- | AY340144.1 | -- | AY340173.1 | -- | -- | -- | -- | -- |
| *Pseudechis porphyriacus* | EU547096.1 | EU547145.1 | -- | EU546913.1 | EU547047.1 | -- | AY340170.1 | -- | EU546874.1 | -- | -- | -- |
| *Pseudelaphe flavirufa* | AY122840.1 | -- | -- | DQ902068.1 | DQ902109.1 | DQ902216.1 | DQ902289.1 | -- | -- | -- | -- | -- |
| *Pseudoboa coronata* | AF158412.1 | GQ457763.1 | -- | GQ457884.1 | -- | -- | -- | -- | -- | -- | -- | -- |
| *Pseudoboa neuwiedii* | AF158423.1 | AF158490.1 | -- | GQ895825.1 | GQ895884.1 | -- | -- | -- | -- | -- | -- | -- |
| *Pseudoboa nigra* | GQ457825.1 | GQ457764.1 | JQ599043.1 | AF544729.1 | JQ598948.1 | -- | -- | -- | -- | -- | -- | -- |
| *Pseudoboodon lemniscatus* | -- | -- | -- | DQ486174.1 | DQ486350.1 | -- | DQ486325.1 | -- | -- | -- | -- | -- |
| *Pseudocerastes fieldi* | EU624264.1 | -- | -- | -- | AJ275716.1 | -- | -- | -- | -- | -- | -- | -- |
| *Pseudocerastes persicus* | -- | AJ275769.1 | -- | -- | AJ275716.1 | -- | -- | -- | -- | -- | -- | -- |
| *Pseudocerastes urarachnoides* | -- | -- | -- | -- | KF314715.1 | -- | -- | -- | -- | -- | -- | -- |
| *Pseudoeryx plicatilis* | AF158418.1 | GQ457765.1 | -- | GQ895826.1 | GQ895885.1 | -- | -- | -- | -- | -- | -- | -- |
| *Pseudoferania polylepis* | EF395886.1 | EF395862.1 | -- | EF473655.1 | EF395911.1 | -- | -- | -- | -- | -- | -- | -- |
| *Pseudoficimia frontalis* | -- | -- | -- | GQ895827.1 | GQ895886.1 | -- | -- | -- | -- | -- | -- | -- |
| *Pseudoleptodeira latifasciata* | EU728579.1 | EU728579.1 | -- | -- | EU728579.1 | EU728579.1 | GQ334661.1 | FJ455190.1 |  |  |  |  |
| *Pseudonaja affinis* | -- | -- | -- | -- | -- | -- | DQ098441.1 | -- | -- | -- | -- | -- |
| *Pseudonaja guttata* | -- | -- | -- | -- | -- | -- | DQ098469.1 | -- | -- | -- | -- | -- |
| *Pseudonaja inframacula* | -- | -- | -- | -- | -- | -- | DQ098474.1 | -- | -- | -- | -- | -- |
| *Pseudonaja ingrami* | -- | -- | -- | -- | -- | -- | DQ098488.1 | -- | -- | -- | -- | -- |
| *Pseudonaja modesta* | EU547098.1 | EU547147.1 | -- | EU546915.1 | EU547049.1 | -- | DQ098492.1 | -- | EU546876.1 | -- | -- | -- |
| *Pseudonaja nuchalis* | -- | -- | -- | -- | -- | -- | EF210839.1 | -- | -- | -- | -- | -- |
| *Pseudonaja textilis* | EU547097.1 | EU547146.1 | -- | EU546914.1 | EU547048.1 | -- | EU258951.1 | -- | EU546875.1 | -- | -- | -- |
| *Pseudorabdion oxycephalum* | -- | -- | -- | DQ112083.1 | AF471073.1 | -- | -- | -- | -- | -- | -- | -- |
| *Pseudotomodon trigonatus* | GQ457827.1 | GQ457766.1 | -- | GQ457887.1 | -- | -- | -- | -- | -- | -- | -- | -- |
| *Pseudotyphlops philippinus* | KC347331.1 | KC347369.1 | -- | KC347408.1 | -- | -- | KC347492.1 | -- | -- | KC347446.1 | -- | -- |
| *Pseudoxenodon bambusicola* | JQ598833.1 | JQ598893.1 | JQ599044.1 | JQ598996.1 | -- | -- | -- | FJ434101.1 | -- | -- | EF144090.1 | EF144111.1 |
| *Pseudoxenodon karlschmidti* | JF697319.1 | JF697330.1 | JQ599045.1 | AF471102.1 | AF471080.1 | -- | -- | -- | -- | -- | -- | -- |
| *Pseudoxenodon macrops* | JF697322.1 | JF697333.1 | -- | JF697344.1 | JQ598949.1 | -- | -- | -- | -- | -- | -- | -- |
| *Pseudoxenodon* sp. | JF697318.1 | JF697329.1 | -- | JF697341.1 | -- | -- | -- | -- | -- | -- | -- | -- |
| *Pseudoxyrhopus ambreensis* | FJ404188.1 | AY188074.1 | -- | AY187996.1 | -- | -- | FJ404385.1 | -- | -- | -- | -- | FJ404459.1 |
| *Psomophis genimaculatus* | GQ457828.1 | GQ457767.1 | -- | GQ457888.1 | -- | -- | -- | -- | -- | -- | -- | -- |
| *Psomophis joberti* | GQ457829.1 | GQ457768.1 | JQ599046.1 | GQ895828.1 | GQ895887.1 | -- | -- | -- | -- | -- | -- | -- |
| *Psomophis obtusus* | JQ598836.1 | JQ598896.1 | -- | -- | -- | -- | -- | -- | -- | -- | -- | -- |
| *Ptyas dhumnades* | AF236676.2 | HM439989.1 | -- | -- | DQ272474.1 | -- | -- | -- | -- | -- | -- | -- |
| *Ptyas korros* | AY122670.1 | -- | -- | AY486953.1 | AY486929.1 | AY487023.1 | AY487062.1 | -- | -- | -- | -- | -- |
| *Ptyas luzonensis* | -- | -- | -- | -- | KC010342.1 | -- | -- | -- | -- | -- | -- | -- |
| *Ptyas mucosa* | AY122828.1 | KC589121.1 | -- | GQ225670.1 | AF471054.1 | AY487024.1 | AY487063.1 | -- | -- | -- | -- | -- |
| *Ptychophis flavovirgatus* | GQ457830.1 | GQ457769.1 | -- | GQ457890.1 | -- | -- | -- | -- | -- | -- | -- | -- |
| *Python anchietae* | -- | -- | KF811067.1 | KF811103.1 | KF811118.1 | -- | -- | -- | -- | KF811166.1 | -- | -- |
| *Python bivittatus* | KF010492.1 | KF010492.1 | XM_007433022.1 | AF435016.1 | JX401131.1 | -- | -- | XM_007435795.1 | -- | -- | -- | -- |
| *Python brongersmai* | EF545039.1 | EF545066.1 | -- | -- | EF545107.1 | -- | -- | -- | -- | -- | -- | -- |
| *Python curtus* | AF368060.1 | AF215277.1 | KF811068.1 | KF811104.1 | KF811119.1 | -- | -- | -- | -- | -- | -- | -- |
| *Python molurus* | EF545038.1 | HM581978 | EU402658.1 | GQ225667.1 | U69853.1 | NC_015812.1 | HM581978 | -- | -- | -- | -- | -- |
| *Python regius* | AB177878.1 | AB177878 | -- | -- | AF337116.1 | AB177878.1 | AB177878 | -- | -- | -- | -- | -- |
| *Python sebae* | EF545037.1 | EF545064.1 | KF811069.1 | KF811105.1 | U69863.1 | -- | -- | -- | -- | KF811167.1 | -- | -- |
| *Pythonodipsas carinata* | FJ404189.1 | AY188075.1 | -- | AY187997.1 | -- | -- | FJ404386.1 | -- | -- | -- | -- | FJ404460.1 |
| *Ramphotyphlops acuticaudus* | -- | -- | GU902381.1 | -- | JQ910543.1 | -- | -- | GU902554.1 | -- | -- | GU902631.1 | -- |
| *Ramphotyphlops lineatus* | -- | -- | GU902384.1 | -- | -- | -- | -- | GU902557.1 | -- | -- | GU902634.1 | -- |
| *Ramphotyphlops* sp. | -- | -- | GU902420.1 | -- | -- | -- | -- | AY988060.1 | -- | AY988077.1 | GU902670.1 | -- |
| *Regina grahamii* | AF402635.1 | -- | KF258599.1 | -- | AF402918.1 | AF384837.1 | KF258633.1 | KF234029.1 | -- | -- | -- | -- |
| *Regina septemvittata* | AF402634.1 | -- | KF258595.1 | -- | AF402917.1 | AF384836.1 | KF258629.1 | KF234025.1 | -- | -- | -- | -- |
| *Rena dissecta* | GQ469230.1 | GQ469230.1 | -- | -- | -- | -- | -- | -- | -- | -- | -- | -- |
| *Rena dulcis* | AF512726.1 | GQ469229.1 | GQ469182.1 | GQ469067.1 | -- | -- | -- | GQ469022.1 | -- | -- | GQ469045.1 | -- |
| *Rena humilis* | GQ469228.1 | AB079597 | EU402648.1 | AY099979.1 | AY099991.1 | AB079597.1 | AB079597 | EU390928.1 | -- | EU402851.1 | -- | -- |
| *Rena* sp. | GQ469231.1 | GQ469231.1 | -- | -- | -- | -- | -- | -- | -- | -- | -- | -- |
| *Rhabdophis adleri* | -- | -- | -- | KF800921.1 | KF800931.1 | -- | -- | -- | -- | -- | -- | -- |
| *Rhabdophis chrysargos* | -- | -- | -- | -- | HG763866.1 | -- | -- | -- | -- | -- | -- | -- |
| *Rhabdophis guangdongensis* | -- | -- | -- | KF800920.1 | KF800930.1 | -- | -- | -- | -- | -- | -- | -- |
| *Rhabdophis himalayanus* | -- | -- | -- | KF800919.1 | KF800929.1 | -- | -- | -- | -- | -- | -- | -- |
| *Rhabdophis leonardi* | -- | -- | -- | KF800922.1 | KF800933.1 | -- | -- | -- | -- | -- | -- | -- |
| *Rhabdophis nigrocinctus* | -- | -- | -- | KF800926.1 | KF800936.1 | -- | -- | -- | -- | -- | -- | -- |
| *Rhabdophis nuchalis* | AF236678.2 | -- | -- | KF800925.1 | GQ281786.1 | JQ687454.1 | JQ687413.1 | KJ685753.1 | KJ685591.1 | -- | -- | -- |
| *Rhabdophis* sp. | -- | -- | -- | -- | HG763864.1 | -- | -- | -- | -- | -- | -- | -- |
| *Rhabdophis subminiatus* | AF544776.1 | AF544805.1 | JQ599047.1 | AF544713.1 | GQ281777.1 | -- | JQ687411.1 | KJ685760.1 | KJ685598.1 | -- | -- | -- |
| *Rhabdophis swinhonis* | -- | -- | -- | AB861888.1 | AB842176.1 | -- | -- | -- | -- | -- | -- | -- |
| *Rhabdophis tigrinus* | AF236679.2 | -- | -- | AF471119.1 | AF471051.1 | JQ687460.1 | JQ687419.1 | KJ685762.1 | KJ685600.1 | -- | -- | -- |
| *Rhachidelus brazili* | JQ598837.1 | JQ598897.1 | JQ599048.1 | -- | JQ598952.1 | -- | -- | -- | -- | -- | -- | -- |
| *Rhadinaea flavilata* | -- | -- | -- | AF471152.1 | AF471078.1 | -- | -- | -- | -- | -- | -- | -- |
| *Rhadinaea fulvivittis* | -- | -- | -- | -- | EF078539.1 | -- | EF078587.1 | GU353275.1 | -- | -- | -- | -- |
| *Rhagerhis moilensis* | AY643313.1 | HQ267802.1 | -- | DQ486157.1 | DQ486333.1 | -- | DQ486309.1 | -- | -- | -- | -- | -- |
| *Rhamphiophis oxyrhynchus* | Z46443.1 | FJ404213.1 | JQ599049.1 | AF544710.1 | JQ598953.1 | -- | -- | -- | -- | -- | -- | FJ404400.1 |
| *Rhamphiophis rostratus* | FJ404126.1 | AY611897.1 | -- | DQ486187.1 | DQ486394.1 | -- | DQ486233.1 | -- | -- | -- | -- | FJ404401.1 |
| *Rhamphiophis rubropunctatus* | FJ404127.1 | -- | -- | FJ404232.1 | FJ404310.1 | -- | FJ404330.1 | -- | -- | -- | -- | FJ404402.1 |
| *Rhinechis scalaris* | AY122802.1 | -- | -- | AY486956.1 | AY486932.1 | AY487029.1 | AY487068.1 | -- | -- | -- | -- | -- |
| *Rhinobothryum lentiginosum* | HM565767.1 | HM582227.1 | -- | AF544693.1 | -- | -- | -- | -- | -- | -- | -- | -- |
| *Rhinocheilus lecontei* | AY122850.1 | -- | -- | FJ627788.1 | AF337109.1 | FJ627838.1 | AF138773.1 | -- | -- | -- | -- | -- |
| *Rhinoleptus koniagui* | GQ469242.1 | GQ469242.1 | GQ469193.1 | GQ469078.1 | -- | -- | -- | GQ469032.1 | -- | -- | GQ469055.1 | -- |
| *Rhinophis blythii* | KC347332.1 | AY701049.1 | -- | KC347409.1 | -- | -- | KC347517.1 | -- | -- | KC347447.1 | -- | -- |
| *Rhinophis dorsimaculatus* | AY701009.1 | AY701040.1 | -- | -- | -- | -- | -- | -- | -- | -- | -- | -- |
| *Rhinophis drummondhayi* | AY700997.1 | AY701028.1 | FJ433966.1 | AF544719.1 | AF544673.1 | -- | -- | FJ434071.1 | -- | -- | AY487386.1 | FJ433896.1 |
| *Rhinophis erangaviraji* | KC347333.1 | KC347371.1 | -- | KC347410.1 | KC347490.1 | -- | KC347503.1 | -- | -- | KC347448.1 | -- | -- |
| *Rhinophis homolepis* | AY701015.1 | AY701046.1 | -- | -- | KC347489.1 | -- | KC347522.1 | -- | -- | -- | -- | -- |
| *Rhinophis oxyrhynchus* | AY701013.1 | AY701044.1 | -- | -- | -- | -- | -- | -- | -- | -- | -- | -- |
| *Rhinophis philippinus* | AY701006.1 | AY701037.1 | -- | -- | -- | GQ200594.1 | -- | -- | -- | -- | -- | -- |
| *Rhinophis travancoricus* | AY701010.1 | AY701041.1 | -- | -- | -- | -- | -- | -- | -- | -- | -- | -- |
| *Rhinoplocephalus bicolor* | EU547117.1 | EU547166.1 | -- | EU546930.1 | EU547068.1 | -- | EU547020.1 | -- | EU546891.1 | -- | -- | -- |
| *Rhinotyphlops lalandei* | -- | -- | GU902386.1 | -- | -- | -- | -- | GU902559.1 | -- | -- | GU902636.1 | -- |
| *Rhinotyphlops unitaeniatus* | -- | -- | GU902452.1 | -- | -- | -- | -- | -- | -- | -- | -- | -- |
| *Rodriguesophis iglesiasi* | JQ598831.1 | JQ598891.1 | -- | GQ895823.1 | GQ895881.1 | -- | -- | -- | -- | -- | -- | -- |
| *Salvadora grahamiae* | AY122680.1 | -- | -- | KP765649.1 | KP765667.1 | -- | -- | -- | -- | -- | -- | -- |
| *Salvadora hexalepis* | -- | -- | -- | -- | -- | -- | AF138748.1 | -- | -- | -- | -- | -- |
| *Salvadora mexicana* | -- | -- | -- | AY486958.1 | AY486934.1 | AY487036.1 | AY487075.1 | -- | -- | -- | -- | -- |
| *Sanzinia madagascariensis* | EU403568.1 | AY336066.1 | AY988033.1 | EU403580.1 | U69866.1 | -- | -- | AY988050.1 | -- | AY988067.1 | -- | -- |
| *Scaphiodontophis annulatus* | -- | -- | -- | GQ927318.1 | GQ927323.1 | -- | -- | -- | GQ927325.1 | GQ927325.1 | -- | -- |
| *Scaphiophis albopunctatus* | -- | -- | -- | DQ486169.1 | DQ486345.1 | -- | DQ486321.1 | -- | -- | -- | -- | -- |
| *Senticolis triaspis* | AY122816.1 | -- | -- | DQ902086.1 | DQ902127.1 | DQ902237.1 | AF138775.1 | -- | -- | -- | -- | -- |
| *Siagonodon septemstriatus* | GQ469232.1 | GQ469232.1 | GQ469191.1 | GQ469076.1 | -- | -- | -- | GQ469030.1 | -- | -- | GQ469053.1 | -- |
| *Sibon nebulatus* | AF544777.1 | AF544806.1 | -- | AF544736.1 | EU728583 | NC_013985.1 | GQ334662.1 | GQ334685.1 | -- | -- | -- | -- |
| *Sibon noalamina* | -- | KP209376.1 | -- | -- | -- | -- | -- | -- | -- | -- | -- | -- |
| *Sibynomorphus mikanii* | GQ457831.1 | JQ627298.1 | JQ599050.1 | GQ457891.1 | JQ598954.1 | -- | -- | -- | -- | -- | -- | -- |
| *Sibynomorphus neuwiedi* | JQ598838.1 | JQ598898.1 | -- | -- | -- | -- | -- | -- | -- | -- | -- | -- |
| *Sibynomorphus turgidus* | JQ598839.1 | JQ598899.1 | -- | -- | -- | -- | -- | -- | -- | -- | -- | -- |
| *Sibynomorphus ventrimaculatus* | JQ598840.1 | JQ598900.1 | -- | JQ598997.1 | -- | -- | -- | -- | -- | -- | -- | -- |
| *Sibynophis bistrigatus* | -- | -- | -- | KC000112.1 | KC000127.1 | KC000130.1 | -- | -- | -- | KC000105.1 | -- | -- |
| *Sibynophis chinensis* | KF360246.1 | KF360246 | -- | KC000113.1 | KF360246 | KC000131.1 | KF360246 | -- | -- | KC000103.1 | -- | -- |
| *Sibynophis collaris* | -- | -- | -- | KC000120.1 | KC000122.1 | KC000133.1 | JN211315 | -- | -- | KC000109.1 | -- | -- |
| *Sibynophis subpunctatus* | KC347335.1 | KC347373.1 | -- | KC347411.1 | KC347471.1 | -- | KC347516.1 | -- | -- | KC347449.1 | -- | -- |
| *Sibynophis triangularis* | -- | -- | -- | KC000116.1 | KC000123.1 | KC000132.1 | -- | -- | -- | KC000106.1 | -- | -- |
| *Simalia amethistina* | EF545018.1 | EF545045.1 | -- | -- | KJ666587.1 | -- | -- | -- | -- | -- | -- | -- |
| *Simalia boeleni* | EF545020.1 | EF545047.1 | KF811070.1 | KF811106.1 | KJ666597.1 | -- | -- | -- | -- | KF811168.1 | -- | -- |
| *Simalia kinghorni* | -- | -- | -- | -- | KJ666591.1 | -- | -- | -- | -- | -- | -- | -- |
| *Simalia nauta* | -- | -- | KF811071.1 | KF811107.1 | KJ666615.1 | -- | -- | -- | -- | KF811169.1 | -- | -- |
| *Simalia oenpelliensis* | EF545019.1 | EF545046.1 | -- | -- | KJ666593.1 | -- | -- | -- | -- | -- | -- | -- |
| *Simalia tracyae* | -- | -- | KF811072.1 | KF811108.1 | KJ666588.1 | -- | -- | -- | -- | KF811170.1 | -- | -- |
| *Simoselaps anomalus* | EU547110.1 | EU547159.1 | -- | EU546924.1 | EU547061.1 | -- | EU547014.1 | -- | EU546885.1 | -- | -- | -- |
| *Simoselaps bertholdi* | EU547111.1 | EU547160.1 | -- | EU546925.1 | EU547062.1 | -- | EU547015.1 | -- | EU546886.1 | -- | -- | -- |
| *Simoselaps bimaculatus* | KF709681.1 | EU547157.1 | -- | EU546920.1 | EU547059.1 | -- | EU547013.1 | -- | -- | -- | -- | -- |
| *Simoselaps littoralis* | KF709682.1 | KF736329.1 | -- | -- | -- | -- | -- | -- | -- | -- | -- | -- |
| *Simoselaps minimus* | KF709688.1 | KF736358.1 | -- | -- | -- | -- | -- | -- | -- | -- | -- | -- |
| *Sinomicrurus japonicus* | D31615.1 | -- | -- | AY058926.1 | AF217831.1 | AY059005.1 | AY058971.1 | -- | -- | -- | -- | -- |
| *Sinomicrurus kelloggi* | -- | -- | -- | EF137424.1 | EF137417.1 | -- | EF137409.1 | -- | -- | -- | -- | -- |
| *Sinomicrurus macclellandi* | D31616.1 | -- | -- | EF137425.1 | EF137418.1 | -- | EF137410.1 | -- | -- | -- | -- | -- |
| *Sinonatrix aequifasciata* | -- | -- | -- | JQ687440.1 | JQ687430.1 | JQ687456.1 | JQ687415.1 | -- | -- | -- | -- | -- |
| *Sinonatrix annularis* | AF236677.2 | HM439988.1 | -- | AF544712.1 | JQ687431.1 | -- | JQ687424.1 | -- | KJ685604.1 | -- | -- | -- |
| *Sinonatrix percarinata* | -- | -- | -- | JQ687439.1 | GQ281784.1 | JQ687455.1 | JQ687414.1 | -- | KJ685607.1 | -- | -- | -- |
| *Siphlophis cervinus* | AF158466.1 | AF158536.1 | -- | GQ895829.1 | GQ895888.1 | -- | -- | -- | -- | -- | -- | -- |
| *Siphlophis compressus* | AF158467.1 | GQ457772.1 | -- | GQ457893.1 | GQ895894.1 | -- | -- | -- | -- | -- | -- | -- |
| *Siphlophis longicaudatus* | JQ598842.1 | JQ598902.1 | -- | JQ598999.1 | -- | -- | -- | -- | -- | -- | -- | -- |
| *Siphlophis pulcher* | GQ457834.1 | GQ457773.1 | JQ599051.1 | GQ457894.1 | JQ598955.1 | -- | -- | -- | -- | -- | -- | -- |
| *Sistrurus catenatus* | DQ464268.1 | AF259119.1 | -- | KF410311.1 | AY223610.1 | JQ609661.1 | HQ257759.1 | KF410327.1 | -- | -- | -- | -- |
| *Sistrurus miliarius* | AF057228.1 | AF259120.1 | -- | -- | EU483385.1 | GQ359815.1 | U41889.1 | -- | -- | -- | -- | -- |
| *Sonora aemula* | -- | -- | -- | JQ265952.1 | JQ265959.1 | -- | JQ265979.1 | -- | -- | JQ265970.1 | -- | -- |
| *Sonora michoacanensis* | -- | -- | -- | JQ265951.1 | JQ265958.1 | -- | JQ265980.1 | -- | -- | JQ265969.1 | -- | -- |
| *Sonora mutabilis* | -- | -- | -- | JQ265950.1 | JQ265956.1 | -- | JQ265975.1 | -- | -- | JQ265960.1 | -- | -- |
| *Sonora semiannulata* | -- | -- | EU402659.1 | AF471164.1 | AF471048.1 | -- | JQ265981.1 | EU390934.1 | -- | JQ265971.1 | -- | -- |
| *Sordellina punctata* | JQ598843.1 | JQ598903.1 | JQ599052.1 | JQ599000.1 | JQ598956.1 | -- | -- | -- | -- | -- | -- | -- |
| *Spalerosophis diadema* | AY039148.1 | HQ267787.1 | -- | AY486950.1 | AY486926.1 | -- | AY487059.1 | -- | -- | -- | -- | -- |
| *Spalerosophis microlepis* | AY647230.1 | -- | -- | -- | -- | -- | -- | -- | -- | -- | -- | -- |
| *Spilotes pullatus* | HM565768.1 | HM582228.1 | -- | AF471110.1 | AF471041.1 | -- | KF669677.1 | -- | -- | -- | -- | -- |
| *Spilotes sulphureus* | HM565766.1 | -- | -- | HQ157831.1 | KF669664.1 | -- | KF669683.1 | -- | -- | -- | -- | -- |
| *Stenorrhina freminvillei* | HM565769.1 | -- | -- | GQ895830.1 | GQ895889.1 | -- | -- | -- | -- | -- | -- | -- |
| *Stichophanes ningshaanensis* | NC_026083.1 | NC_026083.1 | -- | KJ638717.1 | KJ638715.1 | KJ719252.1 | KJ719252.1 | -- | -- | -- | -- | -- |
| *Stoliczkia borneensis* | AF544779.1 | AF544808.1 | FJ433982.1 | AF544721.1 | -- | -- | -- | FJ434083.1 | -- | -- | AY487398.1 | EF144094.1 |
| *Storeria dekayi* | AF402639.1 | JQ598904.1 | KF258593.1 | AF471154.1 | AF471050.1 | EF417460.1 | KF258627.1 | KF234023.1 | -- | -- | -- | -- |
| *Storeria occipitomaculata* | AF402638.1 | -- | -- | -- | AF402921.1 | AF384840.1 | U49323.1 | -- | -- | -- | -- | -- |
| *Storeria storerioides* | -- | -- | KF258600.1 | -- | KF258651.1 | -- | KF258634.1 | KF234030.1 | -- | -- | -- | -- |
| *Subsessor bocourti* | EF395877.1 | EF395853.1 | -- | EF395927.1 | EF395902.1 | -- | -- | -- | -- | -- | -- | -- |
| *Suta fasciata* | EU547113.1 | EU547162.1 | -- | EU546927.1 | EU547064.1 | -- | EU547016.1 | -- | EU546888.1 | -- | -- | -- |
| *Suta suta* | EU547115.1 | EU547164.1 | -- | EU366452.1 | EU547066.1 | -- | EU547018.1 | -- | EU366436.1 | -- | -- | -- |
| *Sympholis lippiens* | -- | -- | -- | GQ895831.1 | GQ895890.1 | -- | -- | -- | -- | -- | -- | -- |
| *Tachymenis chilensis* | -- | -- | -- | -- | -- | -- | HM639913.1 | -- | -- | -- | -- | -- |
| *Tachymenis peruviana* | GU018147.1 | GQ457774.1 | JQ599054.1 | GQ457895.1 | -- | -- | -- | -- | -- | -- | -- | -- |
| *Taeniophallus affinis* | GQ457792.1 | GQ457733.1 | JQ599055.1 | GQ457853.1 | JQ598957.1 | -- | -- | -- | -- | -- | -- | -- |
| *Taeniophallus brevirostris* | GQ457793.1 | GQ457734.1 | JQ599056.1 | GQ457854.1 | JQ598958.1 | -- | -- | -- | -- | -- | -- | -- |
| *Taeniophallus nicagus* | JQ598845.1 | JQ598906.1 | -- | JQ599001.1 | -- | -- | -- | -- | -- | -- | -- | -- |
| *Tantalophis discolor* | -- | -- | -- | -- | EF078541.1 | -- | EF078589.1 | FJ810240.1 | -- | -- | -- | -- |
| *Tantilla coronata* | -- | -- | -- | KP765653.1 | KP765669.1 | -- | -- | -- | -- | -- | -- | -- |
| *Tantilla gracilis* | -- | -- | -- | KP765654.1 | KP765670.1 | -- | -- | -- | -- | -- | -- | -- |
| *Tantilla hobartsmithi* | -- | -- | -- | KP765650.1 | KP765671.1 | -- | -- | -- | -- | -- | -- | -- |
| *Tantilla melanocephala* | AF158424.1 | AF158491.1 | -- | -- | -- | -- | -- | -- | -- | -- | -- | -- |
| *Tantilla nigriceps* | -- | -- | -- | KP765655.1 | KP765672.1 | -- | -- | -- | -- | -- | -- | -- |
| *Tantilla planiceps* | -- | -- | -- | KP765651.1 | KP765673.1 | -- | -- | -- | -- | -- | -- | -- |
| *Tantilla relicta* | -- | -- | -- | AF471107.1 | AF471045.1 | -- | -- | -- | -- | -- | -- | -- |
| *Telescopus dhara* | HQ658443.1 | HQ267786.1 | -- | -- | -- | -- | -- | -- | -- | -- | -- | -- |
| *Telescopus fallax* | -- | AY188078.1 | -- | AF471108.1 | AF471043.1 | -- | -- | -- | -- | -- | -- | -- |
| *Tetracheilostoma breuili* | GQ469205.1 | GQ469205.1 | -- | -- | -- | -- | -- | -- | -- | -- | -- | -- |
| *Tetracheilostoma carlae* | GQ469202.1 | GQ469202.1 | -- | -- | -- | -- | -- | -- | -- | -- | -- | -- |
| *Thamnodynastes hypoconia* | JQ598846.1 | -- | -- | -- | -- | -- | -- | -- | -- | -- | -- | -- |
| *Thamnodynastes lanei* | GQ457836.1 | GQ457775.1 | -- | -- | -- | -- | -- | -- | -- | -- | -- | -- |
| *Thamnodynastes pallidus* | GU018155.1 | GU018166.1 | -- | GQ895832.1 | GQ895891.1 | -- | -- | -- | -- | -- | -- | -- |
| *Thamnodynastes rutilus* | GQ457837.1 | GQ457776.1 | -- | GQ457896.1 | -- | -- | -- | -- | -- | -- | -- | -- |
| *Thamnodynastes strigatus* | JQ598847.1 | JQ598907.1 | JQ599057.1 | -- | JQ598959.1 | -- | -- | -- | -- | -- | -- | -- |
| *Thamnophis atratus* | -- | -- | -- | -- | AF420085.1 | AF420087.1 | AF420088.1 | -- | -- | -- | -- | -- |
| *Thamnophis brachystoma* | -- | -- | -- | -- | AF420089.1 | AF420091.1 | AF420092.1 | -- | -- | -- | -- | -- |
| *Thamnophis butleri* | AF402640.1 | -- | KF258594.1 | -- | AF402923.1 | AF420094.1 | KF258628.1 | KF234024.1 | -- | -- | -- | -- |
| *Thamnophis chrysocephalus* | -- | -- | -- | -- | -- | AF420097.1 | AF420098.1 | -- | -- | -- | -- | -- |
| *Thamnophis couchii* | AF402653.1 | -- | -- | -- | AF402936.1 | AF420105.1 | AF420106.1 | -- | -- | -- | -- | -- |
| *Thamnophis cyrtopsis* | AF402641.1 | -- | -- | -- | EF417412.1 | AF420101.1 | EF417364.1 | -- | -- | -- | -- | -- |
| *Thamnophis elegans* | AF402642.1 | -- | -- | -- | EF417411.1 | AY136238.1 | EF417363.1 | -- | -- | -- | -- | -- |
| *Thamnophis eques* | -- | -- | -- | -- | AF420117.1 | AF420119.1 | AF420120.1 | -- | -- | -- | -- | -- |
| *Thamnophis exsul* | -- | -- | -- | -- | AF420125.1 | AF420127.1 | AF420128.1 | -- | -- | -- | -- | -- |
| *Thamnophis fulvus* | -- | -- | -- | -- | AF420129.1 | AF420131.1 | AF420132.1 | -- | -- | -- | -- | -- |
| *Thamnophis gigas* | -- | -- | -- | -- | AF420133.1 | AF420209.1 | AF414094.1 | -- | -- | -- | -- | -- |
| *Thamnophis godmani* | -- | -- | -- | AF471165.1 | -- | AF420137.1 | AF420138.1 | -- | -- | -- | -- | -- |
| *Thamnophis hammondii* | -- | -- | -- | -- | AF420139.1 | AF420141.1 | AF420142.1 | -- | -- | -- | -- | -- |
| *Thamnophis marcianus* | AF402643.1 | -- | EU402660.1 | -- | AF402926.1 | AF420145.1 | AF420146.1 | EU390935.1 | -- | EU402862.1 |  | -- |
| *Thamnophis melanogaster* | -- | -- | -- | -- | EF417410.1 | AF420149.1 | EF417362.1 | -- | -- | -- | -- | -- |
| *Thamnophis nigronuchalis* | -- | -- | -- | -- | AF420153.1 | AF420155.1 | AF420156.1 | -- | -- | -- | -- | -- |
| *Thamnophis ordinoides* | AF402644.1 | -- | -- | -- | AF402927.1 | AF420159.1 | AF420160.1 | -- | -- | -- | -- | -- |
| *Thamnophis proximus* | AF402645.1 | -- | KF258607.1 | -- | AF402928.1 | AF420163.1 | KF258641.1 | KF234037.1 | -- | -- | -- | -- |
| *Thamnophis pulchrilatus* | -- | -- | -- | -- | AF420165.1 | AF420167.1 | AF420168.1 | -- | -- | -- | -- | -- |
| *Thamnophis radix* | AF402651.1 | -- | -- | -- | AF402934.1 | AF420171.1 | AF420172.1 | -- | -- | -- | -- | -- |
| *Thamnophis rufipunctatus* | -- | -- | -- | -- | AF420173.1 | AF420175.1 | AF420176.1 | -- | -- | -- | -- | -- |
| *Thamnophis sauritus* | -- | -- | -- | -- | AF420177.1 | AF420179.1 | AF420180.1 | -- | -- | -- | -- | -- |
| *Thamnophis scalaris* | -- | -- | -- | -- | AF420181.1 | AF420183.1 | AF420184.1 | -- | -- | -- | -- | -- |
| *Thamnophis scaliger* | -- | -- | -- | -- | AF420189.1 | AF420187.1 | AF420188.1 | -- | -- | -- | -- | -- |
| *Thamnophis sirtalis* | AF402646.1 | -- | JQ599058.1 | DQ902094.1 | AF402930.1 | DQ995395.1 | AY136272.1 | -- | -- | -- | -- | -- |
| *Thamnophis sumichrasti* | -- | -- | -- | -- | AF420197.1 | AF420199.1 | AF420200.1 | -- | -- | -- | -- | -- |
| *Thamnophis valida* | -- | -- | -- | -- | EF417399.1 | EF417447.1 | EF417351.1 | -- | -- | -- | -- | -- |
| *Thamnosophis epistibes* | -- | DQ979965.1 | -- | DQ979972.1 | DQ979979.1 | -- | -- | -- | -- | -- | -- | -- |
| *Thamnosophis infrasignatus* | -- | -- | -- | -- | FJ403475.1 | -- | -- | -- | -- | -- | -- | -- |
| *Thamnosophis lateralis* | -- | DQ979963.1 | JQ073078.1 | -- | DQ979977.1 | -- | -- | -- | -- | JQ073199.1 | JQ073199.1 | -- |
| *Thamnosophis martae* | -- | DQ979967.1 | -- | DQ979974.1 | DQ979981.1 | -- | -- | -- | -- | -- | -- | -- |
| *Thamnosophis mavotenda* | -- | -- | -- | -- | FJ403478.1 | -- | -- | -- | -- | -- | -- | -- |
| *Thamnosophis stumpffi* | -- | DQ979969.1 | -- | DQ979976.1 | DQ979983.1 | -- | -- | -- | -- | -- | -- | -- |
| *Thelotornis capensis* | -- | -- | -- | AF471109.1 | AF471042.1 | -- | -- | -- | -- | -- | -- | -- |
| *Thermophis baileyi* | -- | -- | -- | EU496917.1 | EU864147.1 | JF411072.1 | EU864149.1 | -- | -- | -- | -- | -- |
| *Thermophis shangrila* | -- | -- | -- | KF514883.1 | KF038430.1 | -- | KF038435.1 | -- | -- | -- | -- | -- |
| *Thermophis zhaoermii* | GQ166168.1 | GQ166168 | -- | KF514882.1 | GQ166168 | JF411071.1 | EU864150.1 | -- | -- | -- | -- | -- |
| *Thrasops jacksonii* | -- | -- | -- | DQ112084.1 | AF471044.1 | -- | -- | -- | -- | -- | -- | -- |
| *Tomodon dorsatum* | GQ457838.1 | GQ457777.1 | JQ599059.1 | GQ895833.1 | GQ895892.1 | -- | -- | -- | -- | -- | -- | -- |
| *Toxicocalamus loriae* | GQ397244.1 | GQ397235.1 | -- | GQ397225.1 | GQ397170.1 | -- | GQ397211.1 | -- | GQ397197.1 | -- | -- | -- |
| *Toxicocalamus preussi* | EU547092.1 | EU547141.1 | -- | EU546909.1 | EU547043.1 | -- | EU547001.1 | -- | EU546870.1 | -- | -- | -- |
| *Toxicodryas pulverulenta* | -- | -- | -- | AF471118.2 | AF471047.1 | -- | -- | -- | -- | -- | -- | -- |
| *Trachischium monticola* | -- | -- | -- | JQ687453.1 | JQ687435.1 | -- | JQ687428.1 | -- | KJ685570.1 | -- | -- | -- |
| *Trachyboa boulengeri* | AF512730.1 | AF512730.1 | EU402661.1 | -- | -- | -- | U49295.1 | EU390936.1 | -- | EU402863.1 | -- | -- |
| *Trachyboa gularis* | AF544756.1 | AF544829.1 | FJ433963.1 | AY491999.1 | -- | -- | -- | FJ434068.1 | -- | -- | AY487409.1 | FJ433893.1 |
| *Tretanorhinus nigroluteus* | -- | -- | -- | GQ895834.1 | GQ895893.1 | -- | -- | -- | -- | -- | -- | -- |
| *Tretanorhinus variabilis* | AF158460.1 | AF158529.1 | -- | -- | -- | -- | -- | -- | -- | -- | -- | -- |
| *Trilepida macrolepis* | GQ469226.1 | GQ469226.1 | -- | -- | -- | -- | -- | -- | -- | -- | -- | -- |
| *Trimeresurus albolabris* | AY352803.1 | KF311102 | -- | -- | KF311102 | KF311102.1 | AY352837.1 | -- | -- | -- | -- | -- |
| *Trimeresurus andersonii* | AY352801.1 | AY352740.1 | -- | -- | -- | -- | AY352835.1 | -- | -- | -- | -- | -- |
| *Trimeresurus borneensis* | AY352783.1 | AY352722.1 | -- | -- | AY352754.1 | -- | AY352817.1 | -- | -- | -- | -- | -- |
| *Trimeresurus cantori* | AY352802.1 | AF057243.1 | -- | -- | AY223568.1 | -- | AY352836.1 | -- | -- | -- | -- | -- |
| *Trimeresurus cardamomensis* | KR021096.1 | KR021137.1 | -- | -- | -- | -- | KR021070.1 | -- | -- | -- | -- | -- |
| *Trimeresurus erythrurus* | AF517161.1 | AF517174.1 | -- | -- | AY352768.1 | -- | AY352834.1 | -- | -- | -- | -- | -- |
| *Trimeresurus fasciatus* | GQ428492.1 | GQ428466.1 | -- | -- | GQ428475.1 | -- | GQ428482.1 | -- | -- | -- | -- | -- |
| *Trimeresurus flavomaculatus* | AY059535.1 | AY059551.1 | -- | -- | AY352764.1 | -- | AY059584.1 | -- | -- | -- | -- | -- |
| *Trimeresurus gracilis* | AY352789.1 | AY352728.1 | -- | -- | DQ305461.1 | -- | AY352823.1 | -- | -- | -- | -- | -- |
| *Trimeresurus gramineus* | AY352793.1 | AY352731.1 | -- | -- | AY352762.1 | -- | AY352827.1 | -- | -- | -- | -- | -- |
| *Trimeresurus gumprechti* | AY352798.1 | AY352736.1 | -- | -- | AY352766.1 | -- | AY352832.1 | -- | -- | -- | -- | -- |
| *Trimeresurus hageni* | AY059536.1 | AY059552.1 | -- | -- | AY059567.1 | -- | AY059585.1 | -- | -- | -- | -- | -- |
| *Trimeresurus insularis* | AY059534.1 | AF517172.1 | -- | -- | AY059568.1 | -- | AY059586.1 | -- | -- | -- | -- | -- |
| *Trimeresurus kanburiensis* | AY289219.1 | AY352737.1 | -- | -- | -- | -- | -- | -- | -- | -- | -- | -- |
| *Trimeresurus macrops* | GQ428493.1 | AF517176.1 | -- | -- | -- | -- | AF517219.1 | -- | -- | -- | -- | -- |
| *Trimeresurus malabaricus* | AY059548.1 | AY059564.1 | -- | -- | AY352763.1 | -- | AY059587.1 | -- | -- | -- | -- | -- |
| *Trimeresurus malcolmi* | AY371758.1 | AY371793.1 | -- | -- | -- | -- | AY371861.1 | -- | -- | -- | -- | -- |
| *Trimeresurus mcgregori* | AY371756.1 | AY371795.1 | -- | -- | -- | -- | -- | -- | -- | -- | -- | -- |
| *Trimeresurus medoensis* | AY352797.1 | AY352735.1 | -- | -- | AY352765.1 | -- | AY352831.1 | -- | -- | -- | -- | -- |
| *Trimeresurus popeiorum* | AY059538.1 | AY059554.1 | -- | -- | AY059571.1 | -- | AY059590.1 | -- | -- | -- | -- | -- |
| *Trimeresurus puniceus* | AF517164.1 | AF517177.1 | -- | -- | AY352757.1 | -- | AF517220.1 | -- | -- | -- | -- | -- |
| *Trimeresurus purpureomaculatus* | AY352806.1 | AY352745.1 | -- | -- | AY352772.1 | -- | AY352840.1 | -- | -- | -- | -- | -- |
| *Trimeresurus rubeus* | KR021105.1 | KR021141.1 | -- | -- | -- | -- | KR021075.1 | -- | -- | -- | -- | -- |
| *Trimeresurus schultzei* | AY352785.1 | AY352725.1 | -- | -- | AY352756.1 | -- | AY352819.1 | -- | -- | -- | -- | -- |
| *Trimeresurus septentrionalis* | AY352784.1 | AY352724.1 | -- | -- | AY352755.1 | -- | AY352818.1 | -- | -- | -- | -- | -- |
| *Trimeresurus sichuanensis* | HQ850445.1 | HQ850446.1 | -- | -- | HQ850448.1 | -- | HQ850450.1 | -- | -- | -- | -- | -- |
| *Trimeresurus stejnegeri* | AY059547.1 | FJ752492 | -- | -- | FJ752492 | FJ752492.1 | AY059595.1 | -- | -- | -- | -- | -- |
| *Trimeresurus sumatranus* | AY371760.1 | AY371788.1 | -- | -- | -- | -- | AY371866.1 | -- | -- | -- | -- | -- |
| *Trimeresurus tibetanus* | AY352776.1 | AY352715.1 | -- | -- | AY352749.1 | -- | AY352810.1 | -- | -- | -- | -- | -- |
| *Trimeresurus trigonocephalus* | AY059549.1 | KC347374.1 | -- | KC347412.1 | KC347479.1 | -- | AY059597.1 | -- | -- | -- | -- | -- |
| *Trimeresurus truongsonensis* | EU443817.1 | EU443818.1 | -- | -- | EU443815.1 | -- | EU443816.1 | -- | -- | -- | -- | -- |
| *Trimeresurus venustus* | AY289216.1 | AY352723.1 | -- | -- | AF171914.1 | -- | AY289228.1 | -- | -- | -- | -- | -- |
| *Trimeresurus vogeli* | AF517170.1 | AF517183.1 | -- | -- | AY059574.1 | -- | AF517225.1 | -- | -- | -- | -- | -- |
| *Trimeresurus wiroti* | -- | -- | -- | -- | DQ646788.1 | -- | -- | -- | -- | -- | -- | -- |
| *Trimeresurus yunnanensis* | EU443811.1 | EU443812.1 | -- | -- | EF597522.1 | -- | EF597527.1 | -- | -- | -- | -- | -- |
| *Trimetopon gracile* | GU018160.1 | GU018178.1 | -- | -- | -- | -- | -- | -- | -- | -- | -- | -- |
| *Trimorphodon biscutatus* | -- | -- | EU402662.1 | GQ927319.1 | GQ927324.1 | -- | DQ497506.1 | EU390937.1 | -- | EU402864.1 | -- | -- |
| *Tropidechis carinatus* | EU547130.1 | EU547179.1 | -- | EU546943.1 | EU547081.1 | -- | EU547033.1 | -- | EU546904.1 | -- | -- | -- |
| *Tropidoclonion lineatum* | AF402648.1 | -- | KF258604.1 | -- | AF402931.1 | AF420207.1 | KF258638.1 | KF234034.1 | -- | -- | -- | -- |
| *Tropidodipsas sartorii* | -- | -- | -- | -- | EF078540.1 | -- | EF078588.1 | -- | -- | -- | -- | -- |
| *Tropidodryas serra* | JQ598848.1 | JQ598908.1 | -- | -- | JQ598961.1 | -- | -- | -- | -- | -- | -- | -- |
| *Tropidodryas striaticeps* | GQ457839.1 | GQ457778.1 | JQ599060.1 | -- | AF236811.1 | -- | -- | -- | -- | -- | -- | -- |
| *Tropidolaemus wagleri* | AY352788.1 | AY352727.1 | -- | -- | GQ428472.1 | -- | AY352822.1 | -- | -- | -- | -- | -- |
| *Tropidophis canus* | -- | -- | KF811073.1 | KF811109.1 | KF811123.1 | -- | -- | KF811142.1 | -- | KF811171.1 | -- | -- |
| *Tropidophis feicki* | AF512733.1 | AF512733.1 | KF811074.1 | KF811110.1 | KF811124.1 | -- | -- | KF811143.1 | -- | -- | -- | -- |
| *Tropidophis greenwayi* | AF512731.1 | AF512731.1 | KF811075.1 | KF811111.1 | KF811125.1 | -- | -- | -- | -- | -- | -- | -- |
| *Tropidophis haetianus* | FJ755181.1 | FJ755181 | EU402663.1 | AY099962.1 | FJ755181 | FJ755181.1 | FJ755181 | EU390938.1 | -- | AY988073.1 | -- | -- |
| *Tropidophis melanurus* | AF512734.1 | AF512734.1 | FJ433962.1 | AF544725.1 | -- | -- | -- | FJ434067.1 | -- | -- | AY487384.1 | FJ433892.1 |
| *Tropidophis pardalis* | AF512732.1 | AF512732.1 | -- | -- | -- | -- | -- | -- | -- | -- | -- | -- |
| *Tropidophis taczanowskyi* | -- | -- | KF811076.1 | KF811112.1 | KF811126.1 | -- | -- | KF811144.1 | -- | -- | -- | -- |
| *Tropidophis wrighti* | Z46445.1 | Z46476.1 | -- | -- | -- | -- | -- | -- | -- | -- | -- | -- |
| *Typhlophis squamosus* | -- | -- | GU902398.1 | -- | -- | -- | -- | -- | -- | -- | GU902648.1 | -- |
| *Typhlops agoralionis* | KF993133.1 | KF993182.1 | GU902422.1 | -- | KF993234.1 | -- | -- | GU902592.1 | -- | -- | GU902672.1 | -- |
| *Typhlops anchaurus* |  | KF993183.1 | KF993231.1 | -- | KF993235.1 | -- | -- | GU902593.1 | -- | -- | GU902673.1 | -- |
| *Typhlops anousius* | KF993135.1 | KF993184.1 | GU902445.1 | -- | KF993236.1 | -- | -- | GU902615.1 | -- | -- | -- | -- |
| *Typhlops arator* | KF993136.1 | KF993185.1 | GU902424.1 | -- | KF993237.1 | -- | -- | GU902594.1 | -- | -- | GU902674.1 | -- |
| *Typhlops biminiensis* | AF366748.1 | AF366817.1 | -- | -- | -- | -- | -- | -- | -- | -- | -- | -- |
| *Typhlops capitulatus* | AF366701.1 | AF366770.1 | GU902425.1 | -- | KF993242.1 | -- | -- | GU902595.1 | -- | -- | GU902675.1 | -- |
| *Typhlops catapontus* | KF993142.1 | KF993190.1 | GU902426.1 | -- | KF993243.1 | -- | -- | GU902596.1 | -- | -- | GU902676.1 | -- |
| *Typhlops caymanensis* | KF993143.1 | KF993191.1 | GU902427.1 | -- | KF993244.1 | -- | -- | GU902597.1 | -- | -- | GU902677.1 | -- |
| *Typhlops contorhinus* | KF993145.1 | KF993193.1 | GU902446.1 | -- | KF993246.1 | -- | -- | GU902616.1 | -- | -- | GU902692.1 | -- |
| *Typhlops dominicanus* | AF366724.1 | AF366793.1 | GU902428.1 | -- | KF993249.1 | -- | -- | GU902598.1 | -- | -- | -- | -- |
| *Typhlops eperopeus* | KF993150.1 | KF993198.1 | GU902444.1 | -- | KF993251.1 | KF993292.1 | -- | GU902614.1 | -- | -- | GU902690.1 | -- |
| *Typhlops geotomus* | KF993151.1 | KF993199.1 | -- | -- | KF993252.1 | -- | -- | -- | -- | -- | -- | -- |
| *Typhlops granti* | AF366713.1 | AF366782.1 | GU902430.1 | -- | KF993253.1 | -- | -- | GU902600.1 | -- | -- | GU902678.1 | -- |
| *Typhlops guadeloupensis* | KF993153.1 | KF993201.1 | -- | -- | KF993254.1 | -- | -- | -- | -- | -- | -- | -- |
| *Typhlops hectus* | AF366731.1 | AF366800.1 | KF993232.1 | -- | KF993257.1 | -- | -- | -- | -- | -- | -- | -- |
| *Typhlops hypomethes* | KF993157.1 | AF366786.1 | GU902431.1 | -- | KF993258.1 | -- | -- | GU902601.1 | -- | -- | GU902679.1 | -- |
| *Typhlops jamaicensis* | AF366695.1 | AF366764.1 | EU402664.1 | AF544733.1 | KF993259.1 | -- | -- | GU902602.1 | -- | EU402866.1 | AY487387.1 | FJ433888.1 |
| *Typhlops lumbricalis* | AF366700.1 | AF366769.1 | FJ433958.1 | -- | -- | -- | -- | GU902603.1 | -- | -- | GU902680.1 | -- |
| *Typhlops monastus* | AF366719.1 | AF366788.1 | GU902434.1 | -- | KF993262.1 | -- | -- | GU902599.1 | -- | -- | -- | -- |
| *Typhlops naugus* | KF993162.1 | KF993210.1 | -- | -- | KF993263.1 | -- | -- | -- | -- | -- | -- | -- |
| *Typhlops notorachius* | KF993163.1 | KF993211.1 | GU902436.1 | -- | KF993264.1 | KF993296.1 | -- | GU902606.1 | -- | -- | GU902682.1 | -- |
| *Typhlops perimychus* | KF993167.1 | KF993215.1 | KF993233.1 | -- | KF993268.1 | -- | -- | -- | -- | -- | -- | -- |
| *Typhlops platycephalus* | AF366714.1 | AF366783.1 | GU902437.1 | AY099981.1 | AY099992.1 | -- | -- | GU902607.1 | -- | -- | GU902683.1 | -- |
| *Typhlops proancylops* | KF993169.1 | KF993218.1 | -- | -- | -- | -- | -- | -- | -- | -- | -- | -- |
| *Typhlops pusillus* | AF366754.1 | AF366825.1 | -- | -- | KF993273.1 | -- | -- | -- | -- | -- | -- | -- |
| *Typhlops richardi* | AF366715.1 | AF366784.1 | GU902438.1 | -- | KF993277.1 | -- | -- | GU902608.1 | -- | -- | GU902684.1 | -- |
| *Typhlops rostellatus* | KF993177.1 | AF366777.1 | GU902439.1 | -- | KF993278.1 | -- | -- | GU902609.1 | -- | -- | GU902685.1 | -- |
| *Typhlops schwartzi* | KF993178.1 | AF366779.1 | GU902440.1 | -- | KF993279.1 | KF993302.1 | -- | GU902610.1 | -- | -- | GU902686.1 | -- |
| *Typhlops* sp. 1 | KF993168.1 | KF993217.1 | -- | -- | KF993270.1 | KF993300.1 | -- | -- | -- | -- | -- | -- |
| *Typhlops* sp. 2 | KF993164.1 | KF993212.1 | -- | -- | KF993265.1 | KF993297.1 | -- | -- | -- | -- | -- | -- |
| *Typhlops* sp. 3 | KF993147.1 | KF993195.1 | -- | -- | KF993248.1 | KF993290.1 | -- | -- | -- | -- | -- | -- |
| *Typhlops* sp. 4 | KF993165.1 | KF993213.1 | -- | -- | KF993266.1 | KF993298.1 | -- | -- | -- | -- | -- | -- |
| *Typhlops* sp. 5 | KF993146.1 | KF993194.1 | -- | -- | KF993247.1 | KF993289.1 | -- | -- | -- | -- | -- | -- |
| *Typhlops* sp. 6 | KF993139.1 | KF993187.1 | -- | -- | KF993240.1 | KF993286.1 | -- | -- | -- | -- | -- | -- |
| *Typhlops* sp. 7 | KF993159.1 | KF993207.1 | -- | -- | KF993260.1 | KF993294.1 | -- | -- | -- | -- | -- | -- |
| *Typhlops* sp. 8 | KF993155.1 | KF993203.1 | -- | -- | KF993256.1 | -- | -- | -- | -- | -- | -- | -- |
| *Typhlops* sp. 9 | KF993160.1 | KF993208.1 | -- | -- | KF993261.1 | KF993295.1 | -- | -- | -- | -- | -- | -- |
| *Typhlops* sp. 11 | KF993149.1 | KF993197.1 | -- | -- | KF993250.1 | KF993291.1 | -- | -- | -- | -- | -- | - |
| *Typhlops* sp. 12 | KF993137.1 | KF993186.1 | -- | -- | KF993238.1 | KF993285.1 | -- | -- | -- | -- | -- | -- |
| *Typhlops* sp. 13 | KF993140.1 | KF993188.1 | -- | -- | KF993241.1 | KF993287.1 | -- | -- | -- | -- | -- | -- |
| *Typhlops* sp. 14 | KF993144.1 | KF993192.1 | -- | -- | KF993245.1 | KF993288.1 | -- | -- | -- | -- | -- | -- |
| *Typhlops* sp. 15 | KF993154.1 | KF993202.1 | -- | -- | KF993255.1 | KF993293.1 | -- | -- | -- | -- | -- | -- |
| *Typhlops* sp. 16 | KF993175.1 | KF993224.1 | -- | -- | KF993276.1 | -- | -- | -- | -- | -- | -- | -- |
| *Typhlops* sp. 17 | KF993173.1 | KF993222.1 | -- | -- | KF993274.1 | -- | -- | -- | -- | -- | -- | -- |
| *Typhlops* sp. 18 | KF993174.1 | KF993223.1 | -- | -- | KF993275.1 | -- | -- | -- | -- | -- | -- | -- |
| *Typhlops* sp. 19 | KF993170.1 | KF993219.1 | -- | -- | KF993271.1 | KF993301.1 | -- | --- | -- | -- | -- | -- |
| *Typhlops* sp. 20 | KF993171.1 | KF993220.1 | -- | -- | KF993272.1 | KF993173.1 | -- | -- | -- | -- | -- | -- |
| *Typhlops sulcatus* | KF993179.1 | AF366771.1 | GU902441.1 | -- | KF993280.1 | -- | -- | GU902611.1 | -- | -- | GU902687.1 | -- |
| *Typhlops sylleptor* | KF993180.1 | KF993228.1 | GU902442.1 | -- | KF993281.1 | -- | -- | GU902612.1 | -- | -- | GU902688.1 | -- |
| *Typhlops syntherus* | AF366703.1 | AF366772.1 | GU902443.1 | -- | KF993282.1 | -- | -- | GU902613.1 | -- | -- | GU902689.1 | -- |
| *Typhlops titanops* | KF993181.1 | KF993230.1 | -- | -- | KF993284.1 | -- | -- | -- | -- | -- | -- | -- |
| *Ungaliophis continentalis* | AF512741.1 | AF544833.1 | EU402665.1 | AF544724.1 | U69870.1 | -- | -- | FJ434081.1 | -- | EU402867.1 | EU402867.1 | FJ433910.1 |
| *Uromacer catesbyi* | AF158454.1 | AF158523.1 | -- | -- | FJ416714.1 | FJ416752.1 | FJ416788.1 | -- | -- | -- | -- | FJ416825.1 |
| *Uromacer frenatus* | AF158444.1 | AF158513.1 | -- | -- | FJ416715.1 | FJ416753.1 | FJ416789.1 | -- | -- | -- | -- | FJ416826.1 |
| *Uromacer oxyrhynchus* | FJ416701.1 | FJ416712.1 | -- | -- | FJ416716.1 | FJ416754.1 | FJ416790.1 | -- | -- | -- | -- | FJ416827.1 |
| *Uropeltis ceylanicus* | -- | -- | -- | -- | DQ887839.1 | -- | -- | -- | -- | -- | -- | -- |
| *Uropeltis liura* | AY701003.1 | AY701034.1 | -- | -- | -- | -- | -- | -- | -- | -- | -- | -- |
| *Uropeltis melanogaster* | AF512739.1 | AF512739.1 | FJ433965.1 | -- | -- | -- | -- | FJ434070.1 | -- | -- | AY487399.1 | FJ433895.1 |
| *Uropeltis phillipsi* | AY701011.1 | AY701042.1 | -- | AF471100.1 | AF471034.1 | -- | -- | -- | -- | -- | -- | -- |
| *Uropeltis* sp. | AY701001.1 | AY701032.1 | -- | -- | -- | -- | -- | -- | -- | -- | -- | -- |
| *Vermicella annulata* | KF709676.1 | KF736401.1 | -- | -- | -- | -- | -- | -- | -- | -- | -- | -- |
| *Vermicella calonotus* | KF709674.1 | EU547158.1 | -- | EU546923.1 | EU547060.1 | -- | EF210841.1 | -- | EU546884.1 | -- | -- | -- |
| *Vermicella intermedia* | EU547104.1 | EU547153.1 | -- | EU546919.1 | EU547055.1 | -- | EF210842.1 | -- | EU546880.1 | -- | -- | -- |
| *Vermicella multifasciata* | KF709678.1 | KF736403.1 | -- | -- | -- | -- | -- | -- | -- | -- | -- | -- |
| *Vermicella snelli* | KF709675.1 | KF736400.1 | -- | -- | -- | -- | -- | -- | -- | -- | -- | -- |
| *Vipera ammodytes* | EU624266.1 | EU624297.1 | -- | -- | DQ186504.1 | AY321077.1 | EU624232.1 | -- | -- | -- | -- | -- |
| *Vipera aspis* | JN870190.1 | -- | -- | -- | JX649673.1 | AY321085.1 | -- | -- | -- | -- | -- | -- |
| *Vipera berus* | EU543221.1 | DQ186081.1 | -- | -- | KC176730.1 | AY321075.1 | EU624233.1 | -- | -- | -- | -- | -- |
| *Vipera dinniki* | -- | AJ275773.1 | -- | -- | KC176731.1 | -- | -- | -- | -- | -- | -- | -- |
| *Vipera eriwanensis* | -- | -- | -- | -- | FR727090.1 | -- | FR727021.1 | -- | -- | -- | -- | -- |
| *Vipera kaznakovi* | -- | -- | -- | -- | KC176736.1 | -- | FR727034.1 | -- | -- | -- | -- | -- |
| *Vipera latastei* | -- | -- | -- | -- | JX649607.1 | AY321074.1 | JX649540.1 | -- | -- | -- | -- | -- |
| *Vipera lotievi* | -- | -- | -- | -- | KC176737.1 | -- | FR727030.1 | -- | -- | -- | -- | -- |
| *Vipera orlovi* | -- | -- | -- | -- | KC176746.1 | -- | -- | -- | -- | -- | -- | -- |
| *Vipera renardi* | -- | -- | -- | -- | KC176729.1 | -- | FR727033.1 | -- | -- | -- | -- | -- |
| *Vipera seoanei* | -- | AJ275782.1 | -- | -- | KC176748.1 | AY321071.1 | FR727035.1 | -- | -- | -- | -- | -- |
| *Vipera ursinii* | AF236687.2 | -- | -- | AF433658.1 | FR727045.1 | AY321069.1 | FR726957.1 | -- | -- | -- | -- | -- |
| *Virginia valeriae* | -- | -- | KF258605.1 | KP765645.1 | KF258656.1 | -- | KF258639.1 | KF234035.1 | -- | -- | -- | -- |
| *Walterinnesia aegyptia* | U96807.1 | HQ267785.1 | -- | AY058943.1 | -- | AY059001.1 | AY058988.1 | -- | -- | -- | -- | -- |
| *Xenocalamus transvaalensis* | FJ404141.1 | AY611842.1 | -- | AY611934.1 | AY612025.1 | -- | FJ404344.1 | -- | -- | -- | -- | FJ404416.1 |
| *Xenochrophis asperrimus* | KC347338.1 | KC347376.1 | -- | KC347413.1 | KC347480.1 | -- | Pyron RS-J | -- | -- | KC347451.1 | -- | -- |
| *Xenochrophis flavipunctatus* | AF544780.1 | AF544809.1 | FJ434001.1 | AF544714.1 | -- | FJ416748.1 | -- | FJ434102.1 | -- | -- | -- | EF144112.1 |
| *Xenochrophis piscator* | GQ225679.1 | -- | EU402666.1 | GQ225669.1 | GQ281778.1 | -- | -- | EU390941.1 | -- | EU402868.1 | -- | -- |
| *Xenochrophis punctulatus* | -- | -- | -- | AF471106.1 | AF471079.1 | AY487035.1 | AY487074.1 | -- | -- | -- | -- | -- |
| *Xenochrophis schnurrenbergeri* | GQ225678.1 | -- | -- | GQ225668.1 | GQ225660.1 | -- | -- | -- | -- | -- | -- | -- |
| *Xenochrophis trianguligerus* | -- | -- | -- | -- | -- | -- | U49321.1 | -- | -- | -- | -- | -- |
| *Xenochrophis vittatus* | EF395871.1 | EF395846.1 | -- | EF395920.1 | EF395895.1 | -- | -- | -- | -- | -- | -- | -- |
| *Xenodermus javanicus* | AF544781.1 | AF544810.1 | EU402667.1 | AF544711.1 | -- | -- | U49320.1 | -- | -- | EU402869.1 | -- | -- |
| *Xenodon dorbignyi* | GQ457812.1 | GQ457752.1 | -- | GQ457872.1 | -- | -- | -- | -- | -- | -- | -- | -- |
| *Xenodon guentheri* | JQ598849.1 | JQ598909.1 | -- | -- | -- | -- | -- | -- | -- | -- | -- | -- |
| *Xenodon histricus* | GQ457813.1 | GQ457753.1 | JQ599061.1 | GQ457873.1 | JQ598962.1 | -- | -- | -- | -- | -- | -- | -- |
| *Xenodon matogrossensis* | JQ598850.1 | JQ598910.1 | -- | -- | -- | -- | -- | -- | -- | -- | -- | -- |
| *Xenodon merremi* | GQ457840.1 | JQ598911.1 | JQ599062.1 | GQ895836.1 | GQ895896.1 | -- | -- | -- | -- | -- | -- | -- |
| *Xenodon nattereri* | JQ598851.1 | JQ598912.1 | -- | -- | -- | -- | -- | -- | -- | -- | -- | -- |
| *Xenodon neuwiedii* | GQ457841.1 | GQ457779.1 | -- | -- | AF236814.1 | -- | -- | -- | -- | -- | -- | -- |
| *Xenodon pulcher* | JQ598852.1 | JQ598913.1 | -- | -- | -- | -- | -- | -- | -- | -- | -- | -- |
| *Xenodon semicinctus* | GU018156.1 | GU018173.1 | -- | -- | GQ895877.1 | -- | -- | -- | -- | -- | -- | -- |
| *Xenodon severus* | Z46449.1 | Z46474.1 | JQ599063.1 | -- | JQ598964.1 | -- | -- | -- | -- | -- | -- | -- |
| *Xenodon werneri* | AF158468.1 | AF158538.1 | -- | -- | -- | -- | -- | -- | -- | -- | -- | -- |
| *Xenopeltis unicolor* | AB179620.1 | AB179620 | EU402668.1 | AF544689.1 | AB179620 | AB179620.1 | AB179620 | DQ465562.1 | -- | DQ465564.1 | -- | FJ433898.1 |
| *Xenophidion schaeferi* | -- | -- | -- | -- | AY574279.1 | -- | -- | -- | -- | -- | -- | -- |
| *Xenopholis scalaris* | GU018145.1 | GU018164.1 | -- | GQ895837.1 | GQ895897.1 | -- | -- | -- | -- | -- | -- | -- |
| *Xenopholis undulatus* | JQ598855.1 | JQ598916.1 | -- | JQ599003.1 | -- | -- | -- | -- | -- | -- | -- | -- |
| *Xenotyphlops grandidieri* | -- | -- | GU902457.1 | -- | KF770844.1 | -- | -- | GU902627.2 | -- | JQ073250.1 | GU902702.1 | -- |
| *Xenotyphlops* sp. | -- | -- | GU902456.1 | -- | -- | -- | -- | GU902626.2 | -- | -- | GU902701.1 | -- |
| *Xerotyphlops socotranus* | -- | -- | KC848452.1 | -- | -- | -- | -- | KC848455.1 | -- | -- | -- | -- |
| *Xerotyphlops vermicularis* | HQ113879.1 | -- | GU902397.1 | -- | JQ910544.1 | JQ045201.1 | -- | KF992927.1 | -- | -- | KF992948.1 | -- |
| *Zamenis hohenackeri* | AY122849.1 | -- | -- | DQ902098.1 | DQ902137.1 | DQ902250.1 | DQ902320.1 | -- | -- | -- | -- | -- |
| *Zamenis lineatus* | AY122782.1 | -- | -- | DQ902099.1 | HQ392567.1 | DQ902251.1 | DQ902319.1 | -- | -- | -- | -- | -- |
| *Zamenis longissimus* | AY122780.1 | -- | -- | DQ902072.1 | DQ902138.1 | DQ902221.1 | DQ902293.1 | -- | -- | -- | -- | -- |
| *Zamenis persicus* | AY122789.1 | -- | -- | DQ902075.1 | DQ902117.1 | DQ902225.1 | DQ902297.1 | -- | -- | -- | -- | -- |
| *Zamenis situla* | AY122805.1 | -- | -- | DQ902083.1 | KF639748.1 | DQ902234.1 | -- | -- | -- | -- | -- | -- |
