# Supplementary material for: A Species-Level Phylogeny of Extant Snakes with Description of a New Colubrid Subfamily and Genus
Source: PLoS One. 2016 Sep 7;11(9):e0161070. doi: 10.1371/journal.pone.0161070 (PMC5014348; doi:10.1371/journal.pone.0161070)
Supplement: S2 Table — Tissue samples for Boiga siamensis FMNH267726, Chrysopelea ornata LSUHC7158, and Psammodynastes pictus FMNH267940 were represented by clear chromatograms, but placed poorly in preliminary phylogenetic trees, so they were not included in the final data matrix. Tropidolaemus subannulatus KU327425 was identified as a rogue taxon by RogueNaRok and was pruned from the dataset and thus, is not represented in the phylogeny. (DOCX) [file pone.0161070.s005.docx]

**S2 Table. List of taxa, institutional voucher numbers, and GenBank accession numbers for tissue samples extracted and sequenced in this study.** Tissue samples for *Boiga siamensis* FMNH267726, *Chrysopelea ornata* LSUHC7158, and *Psammodynastes pictus* FMNH267940 were represented by clear chromatograms, but placed poorly in preliminary phylogenetic trees, so they were not included in the final data matrix. *Tropidolaemus subannulatus* KU327425 was identified as a rogue taxon by RogueNaRok and was pruned from the dataset and thus, is not represented in the phylogeny.

| **Taxon** | **Voucher #** | **16S** | **CMOS** | **CYTB** | **ND4** | **NT3** | **RAG-1** |
| --- | --- | --- | --- | --- | --- | --- | --- |
| *Ahaetulla fasciolata* | LSUHC9831 | KX660203 | KX660343 | KX660477 | KX660602 | KX652020 | KX660103 |
| *Ahaetulla fasciolata* | LSUHC8837 | KX660204 | KX660344 | KX660478 | KX660603 | KX652021 | KX660104 |
| *Ahaetulla fronticincta* | CAS222635 | KX660173 | KX660312 | KX660447 | KX660576 |  |  |
| *Ahaetulla fronticincta* | CAS245687 | KX660174 | KX660313 | KX660448 |  | KX652000 |  |
| *Ahaetulla mycterizans* | ADM001 | KX660161 | KX660300 | KX660437 | KX660564 | KX651988 | KX660076 |
| *Ahaetulla mycterizans* | LSUHC8913 | KX660205 | KX660345 | KX660479 | KX660604 | KX652022 | KX660105 |
| *Ahaetulla nasuta* | FMNH255023 | KX660194 | KX660333 | KX660467 | KX660594 | KX652016 | KX660096 |
| *Ahaetulla prasina* | FMNH269042 | KX660195 | KX660334 |  | KX660595 | KX652017 | KX660097 |
| *Ahaetulla prasina* | LSUHC8586 | KX660206 | KX660346 | KX660480 | KX660605 | KX652023 | KX660106 |
| *Asthenodipsas laevis* | FMNH241296 | KX660196 | KX660335 | KX660468 | KX660596 |  |  |
| *Asthenodipsas malaccanus* | FMNH273617 | KX660197 | KX660336 | KX660469 | KX660597 |  |  |
| *Boiga cyanea* | LSUHC7879 | KX660207 | KX660347 |  | KX660606 | KX652024 | KX660107 |
| *Boiga cyanea* | LSUHC6820 | KX660208 | KX660348 |  | KX660607 | KX652025 | KX660108 |
| *Boiga drapiezii* | LSUHC8157 | KX660209 | KX660349 | KX660481 |  | KX652026 | KX660109 |
| *Boiga drapiezii* | LSUHC7295 | KX660210 | KX660350 | KX660482 | KX660608 |  | KX660110 |
| *Boiga jaspidea* | LSUHC7679 | KX660211 | KX660351 | KX660483 |  | KX652027 |  |
| *Boiga jaspidea* | LSUHC7656 | KX660212 | KX660352 | KX660484 | KX660609 | KX652028 | KX660111 |
| *Boiga nigriceps* | LSUHC7020 | KX660213 | KX660353 | KX660485 | KX660610 | KX652029 | KX660112 |
| *Boiga nigriceps* | LSUHC4494 | KX660214 | KX660354 | KX660486 | KX660611 | KX652030 | KX660113 |
| *Boiga quincunciata* | CAS235862 | KX660176 | KX660315 | KX660450 | KX660578 | KX652002 | KX660087 |
| *Boiga quincunciata* | CAS221434 | KX660177 | KX660316 | KX660451 | KX660579 | KX652003 | KX660088 |
| *Boiga siamensis* | CAS215627 | KX660175 | KX660314 | KX660449 | KX660577 | KX652001 | KX660086 |
| *Boiga siamensis* | FMNH267726 |  |  | KX660470 |  |  |  |
| *Boiga siamensis* | LSUHC8502 | KX660215 | KX660355 | KX660487 | KX660612 | KX652031 | KX660114 |
| *Boiga siamensis* | LSUHC8527 | KX660216 | KX660356 | KX660488 | KX660613 | KX652032 | KX660115 |
| *Boiga siamensis* | CAS210147 | KX660257 | KX660398 | KX660527 | KX660645 | KX652068 | KX660141 |
| *Bothrops oligolepis* | LSUMNS9374 | KX660260 |  |  | KX660646 | KX652071 |  |
| *Chironius exoletus* | AMNH152259 | KX660154 | KX660293 | KX660432 | KX660558 | KX651981 | KX660069 |
| *Chironius exoletus* | LSUMNS16518 | KX660261 | KX660401 | KX660530 | KX660647 | KX652072 |  |
| *Chironius fuscus* | KU214847 | KX660240 | KX660381 | KX660510 | KX660637 | KX652054 |  |
| *Chironius fuscus* | LSUMNS13875 | KX660262 | KX660402 | KX660531 | KX660648 | KX652073 | KX660142 |
| *Chironius monticola* | AMNH150692 | KX660155 | KX660294 | KX660433 | KX660559 | KX651982 | KX660070 |
| *Chironius multiventris* | YPM16079 | KX660188 | KX660326 | KX660461 | KX660587 | KX652011 | KX660093 |
| *Chironius multiventris* | YPM16080 | KX660189 | KX660327 | KX660462 | KX660588 | KX652012 | KX660094 |
| *Chironius multiventris* | LSUMNS12556 | KX660263 | KX660403 | KX660532 | KX660649 | KX652074 | KX660143 |
| *Chironius multiventris* | LSUMNS17665 | KX660264 | KX660404 | KX660533 | KX660650 | KX652075 | KX660144 |
| *Chironius scurrulus* | AMNH152263 | KX660156 | KX660295 | KX660434 | KX660560 | KX651983 | KX660071 |
| *Chironius scurrulus* | LSUMNS8940 | KX660265 | KX660405 | KX660534 | KX660651 | KX652076 | KX660145 |
| *Chironius scurrulus* | LSUMNS9368 | KX660266 |  | KX660535 | KX660652 | KX652077 | KX660146 |
| *Chrysopelea ornata* | LSUHC7158 |  | KX660357 | KX660489 | KX660614 |  | KX660116 |
| *Chrysopelea ornata* | LSUHC8851 | KX660217 | KX660358 |  | KX660615 | KX652033 | KX660117 |
| *Chrysopelea pelias* | LSUHC3900 | KX660218 | KX660359 | KX660490 | KX660616 | KX652034 | KX660118 |
| *Chrysopelea pelias* | LSUHC7296 | KX660219 | KX660360 | KX660491 | KX660617 | KX652035 | KX660119 |
| *Cyclophiops multicinctus* | AMNH153707 | KX660157 | KX660296 | KX660435 |  | KX651984 | KX660072 |
| *Cyclophiops multicinctus* | FMNH255569 | KX660198 | KX660337 | KX660471 |  |  | KX660098 |
| *Cyclophiops multicinctus* | KU291939 | KX660241 | KX660382 | KX660511 |  | KX652055 | KX660136 |
| *Dasypeltis atra* | CAS201642 | KX660178 | KX660317 | KX660452 |  | KX652004 |  |
| *Dasypeltis atra* | CAS201640 | KX660179 | KX660318 |  |  | KX652005 |  |
| *Dasypeltis fasciata* | YPM13202 | KX660190 | KX660328 | KX660463 | KX660589 | KX652013 |  |
| *Dendrelaphis cyanochloris* | CAS221428 | KX660181 | KX660320 | KX660454 | KX660580 | KX652007 | KX660089 |
| *Dendrelaphis cyanochloris* | LSUHC6768 | KX660223 | KX660364 | KX660492 | KX660621 | KX652039 | KX660122 |
| *Dendrelaphis formosus* | ADM0002 | KX660162 | KX660301 | KX660438 | KX660565 | KX651989 | KX660077 |
| *Dendrelaphis formosus* | FMNH267935 | KX660199 | KX660338 | KX660472 | KX660598 |  | KX660099 |
| *Dendrelaphis fulginosus* | KU302993 | KX660242 | KX660383 | KX660512 | KX660638 | KX652056 | KX660137 |
| *Dendrelaphis fulginosus* | KU304098 | KX660243 | KX660384 | KX660513 | KX660639 |  |  |
| *Dendrelaphis haasi* | LSUHC10042 | KX660224 | KX660365 | KX660493 | KX660622 | KX652040 | KX660123 |
| *Dendrelaphis marenae* | KU324549 | KX660244 | KX660385 | KX660514 | KX660640 | KX652057 |  |
| *Dendrelaphis marenae* | KU324552 | KX660245 | KX660386 | KX660515 |  | KX652058 | KX660138 |
| *Dendrelaphis ngansonensis* | AMNH148550 | KX660158 | KX660297 |  | KX660561 | KX651985 | KX660073 |
| *Dendrelaphis ngansonensis* | AMNH147134 | KX660159 | KX660298 |  | KX660562 | KX651986 | KX660074 |
| *Dendrelaphis pictus* | CAS222690 | KX660182 | KX660321 | KX660455 | KX660581 | KX652008 | KX660090 |
| *Dendrelaphis pictus* | CAS210338 | KX660183 | KX660322 | KX660456 | KX660582 | KX652009 | KX660091 |
| *Dendrelaphis striatus* | LSUHC10012 | KX660226 | KX660367 | KX660495 | KX660624 | KX652042 | KX660124 |
| *Dendrelaphis striatus* | LSUHC4792 | KX660227 | KX660368 | KX660496 | KX660625 | KX652043 | KX660125 |
| *Dendrelaphis subocularis* | LSUHC7429 | KX660225 | KX660366 | KX660494 | KX660623 | KX652041 |  |
| *Dendroaspis jamesoni* | YPM12647 | KX660191 | KX660329 | KX660464 | KX660590 |  |  |
| *Dendroaspis jamesoni* | YPM13380 | KX660192 | KX660330 |  | KX660591 |  |  |
| *Dendrophidion percarinatum* | MVZ204099 |  | KX660429 | KX660554 | KX660665 | KX652101 |  |
| *Dendrophidion percarinatum* | MVZ204098 | KX660291 | KX660431 | KX660556 | KX660667 | KX652103 | KX660153 |
| *Dipsadoboa brevirostris* | MVZ245373 | KX660285 | KX660424 |  | KX660661 |  | KX660149 |
| *Dipsadoboa duchesnii* | CAS197901 | KX660180 | KX660319 | KX660453 |  | KX652006 |  |
| *Dipsadoboa weileri* | MVZ253208 | KX660288 | KX660427 | KX660552 |  |  |  |
| *Dipsas pavonina* | LSUMNS13989 | KX660267 |  | KX660536 |  | KX652078 | KX660147 |
| *Dipsas pavonina* | LSUMNS14372 | KX660268 |  | KX660537 |  | KX652079 |  |
| *Dipsas peruana* | LSUMNS1532 | KX660269 | KX660406 | KX660538 |  | KX652080 |  |
| *Dryocalamus davisonii* | FMNH255034 | KX660200 | KX660339 | KX660473 |  |  | KX660100 |
| *Dryocalamus davisonii* | LSUHC8479 | KX660228 | KX660369 | KX660497 | KX660626 | KX652044 | KX660126 |
| *Dryocalamus davisonii* | KU328512 | KX660246 | KX660387 | KX660516 |  | KX652059 |  |
| *Dryocalamus subannulatus* | LSUHC5051 | KX660229 | KX660370 | KX660498 | KX660627 | KX652045 | KX660127 |
| *Dryocalamus subannulatus* | LSUHC5576 | KX660230 | KX660371 | KX660499 | KX660628 | KX652046 | KX660128 |
| *Dryocalamus tristrigatus* | FMNH269033 | KX660201 | KX660340 | KX660474 | KX660599 | KX652018 | KX660101 |
| *Dryophiops philippina* | KU321727 | KX660247 | KX660388 | KX660517 | KX660641 | KX652060 | KX660139 |
| *Dryophiops philippina* | KU328968 | KX660248 | KX660389 | KX660518 | KX660642 | KX652061 |  |
| *Dryophiops rubescens* | LSUHC6264 | KX660256 | KX660397 | KX660526 |  | KX652067 |  |
| *Leptophis depressirostris* | YPM16868 |  | KX660331 | KX660465 | KX660592 | KX652014 |  |
| *Leptophis depressirostris* | LSUMNS146385 | KX660270 | KX660407 | KX660539 |  | KX652081 |  |
| *Leptophis diplotropis* | LSUMNS6328 | KX660271 | KX660408 | KX660540 |  | KX652082 | KX660148 |
| *Lepturophis albofuscus* | LSUHC3867 | KX660231 | KX660372 | KX660500 | KX660629 | KX652047 | KX660129 |
| *Lepturophis albofuscus* | LSUHC4588 | KX660232 | KX660373 | KX660501 | KX660630 | KX652048 | KX660130 |
| *Liopeltis frenatus* | CAS225548 | KX660184 | KX660323 | KX660457 | KX660583 |  | KX660092 |
| *Macropisthodon rhodolemas* | ADM0003 | KX660258 | KX660399 | KX660528 |  | KX652069 |  |
| *Micrurus alleni* | ADM258 | KX660163 | KX660302 |  | KX660566 | KX651990 |  |
| *Micrurus alleni* | ADM272 | KX660164 | KX660303 | KX660439 | KX660567 | KX651991 |  |
| *Oxyrhopus fitzingeri* | LSUMNS6586 | KX660272 | KX660409 | KX660541 |  | KX652083 |  |
| *Oxyrhopus formosus* | KU214892 | KX660249 | KX660390 | KX660519 |  | KX652062 |  |
| *Oxyrhopus formosus* | LSUMNS17730 | KX660273 | KX660410 | KX660542 |  | KX652084 |  |
| *Oxyrhopus formosus* | LSUMNS17734 |  | KX660411 | KX660543 |  | KX652085 |  |
| *Oxyrhopus trigeminus* | LSUMNS14365 | KX660274 |  |  | KX660653 | KX652086 |  |
| *Oxyrhopus trigeminus* | LSUMNS14425 |  |  | KX660544 |  | KX652087 |  |
| *Philothamnus irregularis* | KU290488 | KX660250 | KX660391 | KX660520 |  | KX652063 |  |
| *Philothamnus natalensis* | LSUMNS20681 |  | KX660412 |  |  | KX652088 |  |
| *Philothamnus natalensis* | MVZ233358 | KX660290 | KX660430 | KX660555 | KX660666 | KX652102 |  |
| *Phrynonax poecilonotus* | AMNH152285 | KX660160 | KX660299 | KX660436 | KX660563 | KX651987 | KX660075 |
| *Phrynonax poecilonotus* | ADM0005 | KX660167 | KX660306 | KX660441 | KX660570 | KX651994 | KX660080 |
| *Phrynonax shropshirei* | LSUMNS7085 | KX660275 | KX660413 |  | KX660654 | KX652089 |  |
| *Phrynonax shropshirei* | LSUMNS7806 |  | KX660414 |  | KX660655 | KX652090 |  |
| *Pliocercus euryzona* | ADM240 | KX660165 | KX660304 | KX660440 | KX660568 | KX651992 | KX660078 |
| *Pliocercus euryzona* | ADM241 | KX660166 | KX660305 |  | KX660569 | KX651993 | KX660079 |
| *Psammodynastes pictus* | FMNH267940 |  | KX660341 | KX660475 | KX660600 |  |  |
| *Psammodynastes pictus* | LSUHC4093 | KX660237 | KX660378 | KX660507 |  |  |  |
| *Pseudorabdion longiceps* | ADM0004 | KX660259 | KX660400 | KX660529 |  | KX652070 |  |
| *Ptyas carinata* | LSUHC10004 | KX660238 | KX660379 | KX660508 | KX660635 |  |  |
| *Ptyas carinata* | LSUHC7600 | KX660239 | KX660380 | KX660509 | KX660636 | KX652053 | KX660135 |
| *Ptyas fusca* | ADM0006 | KX660168 | KX660307 | KX660442 | KX660571 | KX651995 | KX660081 |
| *Ptyas fusca* | FMNH269019 | KX660202 | KX660342 | KX660476 | KX660601 | KX652019 | KX660102 |
| *Ptyas luzonensis* | KU329295 |  |  | KX660521 |  |  | KX660140 |
| *Ptyas luzonensis* | KU330134 | KX660251 | KX660392 | KX660522 |  | KX652064 |  |
| *Ptyas nigromarginata* | CAS236202 | KX660185 | KX660324 | KX660458 | KX660584 |  |  |
| *Ptyas nigromarginata* | CAS241947 | KX660186 | KX660325 | KX660459 | KX660585 | KX652010 |  |
| *Sibon annulatus* | ADM242 | KX660169 | KX660308 | KX660443 | KX660572 | KX651996 | KX660082 |
| *Sibon annulatus* | ADM0007 | KX660170 | KX660309 | KX660444 | KX660573 | KX651997 | KX660083 |
| *Sibon dimidiatus* | LSUMNS6689 | KX660278 | KX660417 |  | KX660658 | KX652093 |  |
| *Sibynomorphus turgidus* | LSUMNS6459 | KX660279 | KX660418 | KX660547 | KX660659 | KX652094 |  |
| *Sibynomorphus vagus* | KU219121 | KX660252 | KX660393 |  |  |  |  |
| *Spilotes pullatus* | ADM259 | KX660284 | KX660423 | KX660550 |  |  |  |
| *Spilotes sulphureus* | LSUMNS8937 | KX660276 | KX660415 | KX660545 | KX660656 | KX652091 |  |
| *Spilotes sulphureus* | LSUMNS14023 | KX660277 | KX660416 | KX660546 | KX660657 | KX652092 |  |
| *Telescopus beetzi* | MVZ226830 | KX660287 | KX660426 | KX660551 | KX660663 | KX652099 | KX660151 |
| *Telescopus semiannulatus* | LSUMNS6065 | KX660280 | KX660419 | KX660548 |  | KX652095 |  |
| *Thamnodynastes hypoconia* | KU290727 | KX660253 | KX660394 | KX660523 | KX660643 |  |  |
| *Thamnodynastes strigatus* | LSUMNS6563 | KX660281 | KX660420 |  |  | KX652096 |  |
| *Thelotornis capensis* | LSUMNS4846 | KX660282 | KX660421 |  | KX660660 | KX652097 |  |
| *Thelotornis kirtlandii* | YPM12649 | KX660193 | KX660332 | KX660466 | KX660593 | KX652015 | KX660095 |
| *Thelotornis kirtlandii* | MVZ252602 | KX660292 |  | KX660557 |  | KX652104 |  |
| *Thrasops flavigularis* | MVZ253440 | KX660286 | KX660425 |  | KX660662 |  | KX660150 |
| *Toxicodryas blandingii* | LSUMNS20224 | KX660283 | KX660422 | KX660549 |  | KX652098 |  |
| *Toxicodryas pulverulenta* | CAS220642 | KX660187 |  | KX660460 | KX660586 |  |  |
| *Trimeresurus buniana* | LSUHC6118 | KX660233 | KX660374 | KX660502 | KX660631 | KX652049 | KX660131 |
| *Trimeresurus buniana* | LSUHC5608 | KX660234 | KX660375 | KX660503 | KX660632 | KX652050 |  |
| *Trimeresurus cardamonensis* | LSUHC10089 | KX660220 | KX660361 |  | KX660618 | KX652036 | KX660120 |
| *Trimeresurus fucatus* | LSUHC6688 |  |  | KX660504 |  |  | KX660132 |
| *Trimeresurus fucatus* | LSUHC7566 | KX660235 | KX660376 | KX660505 | KX660633 | KX652051 | KX660133 |
| *Trimeresurus honsonensis* | LSUHC8655 | KX660221 | KX660362 |  | KX660619 | KX652037 |  |
| *Trimeresurus honsonensis* | LSUHC8602 | KX660222 | KX660363 |  | KX660620 | KX652038 | KX660121 |
| *Trimeresurus nebularis* | LSUHC10268 | KX660236 | KX660377 | KX660506 | KX660634 | KX652052 | KX660134 |
| *Tropidodipsas fischeri* | MVZ143527 | KX660289 | KX660428 | KX660553 | KX660664 | KX652100 | KX660152 |
| *Tropidolaemus subannulatus* | KU327425 | KX660254 | KX660395 | KX660524 |  | KX652065 |  |
| *Tropidolaemus subannulatus* | KU307696 | KX660255 | KX660396 | KX660525 | KX660644 | KX652066 |  |
| *Ungaliophis panamensis* | ADM239 | KX660171 | KX660310 | KX660445 | KX660574 | KX651998 | KX660084 |
| *Ungaliophis panamensis* | ADM270 | KX660172 | KX660311 | KX660446 | KX660575 | KX651999 | KX660085 |

Voucher Abbreviations: AMNH, American Museum of Natural History; ADM, Alexander D. McKelvy Field Series; CAS, California Academy of Sciences; FMNH, Field Museum of Natural History; KU, University of Kansas Museum of Natural History; LSUHC, La Sierra University Herpetological Collection; LSUMNS, Louisiana State University Museum of Natural Science; MVZ, Museum of Vertebrate Zoology at the University of California-Berkeley; YPM, Yale Peabody Museum of Natural History.
