# Supplementary material for: A Species-Level Phylogeny of Extant Snakes with Description of a New Colubrid Subfamily and Genus
Source: PLoS One. 2016 Sep 7;11(9):e0161070. doi: 10.1371/journal.pone.0161070 (PMC5014348; doi:10.1371/journal.pone.0161070)
Supplement: S3 Table — (DOCX) [file pone.0161070.s006.docx]

**S3 Table. Six loci, gene type, gene length, primer name, PCR annealing temperature and primer source.**

| **Locus** | **Gene Type** | **Length** | **Primer Name** | **Annealing Temperature °C** | **Primer Source** |
| --- | --- | --- | --- | --- | --- |
| 16S | Mitochondrial | 400bp | Forward – L2510 | 50° | Palumbi et al., 1991 |
|  |  |  | Reverse – H3056 | 50° | Hedges, 1994 |
| Cytochrome b | Mitochondrial | 1117bp | Forward – H14910 | 50° | Burbrink et al., 2000 |
|  |  |  | Reverse – THRSN2 | 50° | Burbrink et al., 2000 |
| ND4 | Mitochondrial | 820bp | Forward – ND4 | 50° | Forstner et al., 1995 |
|  |  |  | Reverse - LEU | 50° | Forstner et al., 1995 |
| NT3 | Mitochondrial | 481bp | Forward – NT3-F3 | 50° | Noonan and Chippindale, 2006 |
|  |  |  | Reverse – NT3-R4 | 50° | Noonan and Chippindale, 2006 |
| c-mos | Nuclear | 570bp | Forward – S77 | 50° | Lawson et al., 2005 |
|  |  |  | Reverse – S78 | 50° | Lawson et al., 2005 |
| RAG-1 | Nuclear | 1000bp | Forward – tc0225F | 50° | Castoe and Parkinson, unpubl. |
|  |  |  | Reverse – tc2000R | 50° | Castoe and Parkinson, unpubl. |
