# Supplementary material for: A Species-Level Phylogeny of Extant Snakes with Description of a New Colubrid Subfamily and Genus
Source: PLoS One. 2016 Sep 7;11(9):e0161070. doi: 10.1371/journal.pone.0161070 (PMC5014348; doi:10.1371/journal.pone.0161070)
Supplement: S4 Table — Each taxon is associated with a raw improvement score (R.I.S.), which represents the fraction of improvement in bootstrap support values throughout the tree when the selected taxon is pruned and all rogue taxa above it are also pruned. We performed one run and chose to sacrifice relatively lower node support values to maximize the number of taxa represented in the phylogeny. Thus we elected to only prune taxa with R.I.S. greater than 0.8, resulting in a total of 22 pruned taxa (highlighted in bold). (DOCX) [file pone.0161070.s007.docx]

**S4 Table. Rogue taxa as identified by RogueNaRok Web-Server (**[**http://rnr.h-its.org/submit**](http://rnr.h-its.org/submit)**)**. Each taxon is associated with a raw improvement score (R.I.S.), which represents the fraction of improvement in bootstrap support values throughout the tree when the selected taxon is pruned and all rogue taxa above it are also pruned. We performed one run and chose to sacrifice relatively lower node support values to maximize the number of taxa represented in the phylogeny. Thus we elected to only prune taxa with R.I.S. greater than 0.8, resulting in a total of 22 pruned taxa (highlighted in bold).

| **Taxa** | **R.I.S.** |
| --- | --- |
| ***Tachymenis chilensis*** | **3.407738** |
| ***Xenochrophis trianguligerus*** | **2.955357** |
| ***Dendrelaphis calligaster*** | **2.522817** |
| ***Nothopsis rugosus*** | **1.938492** |
| ***Crisantophis nevermanni*** | **1.965278** |
| ***Oreocalamus hanitschi*** | **1.844246** |
| ***Typhlops lumbricalis*** | **1.753968** |
| ***Trimetopon gracile*** | **1.654762** |
| ***Uropeltis ceylanicus*** | **1.600198** |
| ***Tropidolaemus subannulatus* KU327425** | **1.570437** |
| ***Lycognathophis seychellensis*** | **1.347222** |
| ***Tretanorhinus variabilis*** | **1.272817** |
| ***Erythrolamprus reginae*** | **1.16369** |
| ***Calliophis bivirgata*** | **1.121032** |
| ***Typhlops proancylops*** | **1.103175** |
| ***Corallus caninus*** | **1.051587** |
| ***Psammophis* sp. 2** | **0.992063** |
| ***Cacophis squamulosus*** | **0.969246** |
| ***Mussurana bicolor*** | **0.958333** |
| ***Ficimia streckeri*** | **0.874008** |
| ***Gyalopion canum*** | **1.080357** |
| ***Lycodon stormi*** | **0.809524** |
| *Chironius bicarinatus* | 0.71131 |
| *Crotalus enyo* | 0.685516 |
| *Oxyrhopus trigeminus* LSUMNS14425 | 0.685516 |
| *Atractus badius* | 0.684524 |
| *Tantilla melanocephala* | 0.667659 |
| *Pseustes shropshirei* LSUMNS7085 | 0.661706 |
| *Micrurus decoratus* | 0.637897 |
| *Platyceps variabilis* | 0.593254 |
| *Python anchietae* | 0.546627 |
| *Toxicodryas pulverulenta* | 0.545635 |
| *Macropisthodon rhodolemas* ADM0003 | 0.545635 |
| *Lycodon futsingensis* | 0.53373 |
| *Scaphiophis albopunctatus* | 0.439484 |
| *Alluaudina bellyi* | 0.397817 |
| *Hydrophis stokesii* | 0.394841 |
| *Rhinotyphlops unitaeniatus* | 0.391865 |
| *Xenochrophis flavipunctatus* | 0.390873 |
| *Anilios guentheri* | 0.390873 |
| *Suta suta* | 0.375 |
| *Thamnophis godmani* | 0.375 |
| *Atractaspis irregularis* | 0.365079 |
| *Dipsas albifrons* | 0.350198 |
| *Erpeton tentaculatum* | 0.318452 |
| *Tropidodipsas sartorii* | 0.294643 |
| *Ditypophis sp* | 0.292659 |
| *Antaresia perthensis* | 0.290675 |
| *Hydrophis macdowelli* | 0.289683 |
| *Philodryas viridissima* | 0.271825 |
| *Clelia clelia* | 0.250992 |
| *Hierophis andreanus* | 0.25 |
| *Rhinobothryum lentiginosum* | 0.231151 |
| *Lampropeltis elapsoides* | 0.225198 |
| *Xenophidion schaeferi* | 0.223214 |
| *Rhinophis travancoricus* | 0.22123 |
| *Erythrolamprus typhlus* | 0.21627 |
| *Anilios affinis* | 0.21131 |
| *Boiga kraepelini* | 0.209325 |
| *Atractus reticulatus* | 0.203373 |
| *Sibon noalamina* | 0.203373 |
| *Pantherophis alleghaniensis* | 0.199405 |
| *Ramphotyphlops sp* | 0.198413 |
| *Tropidophis canus* | 0.196429 |
| *Gomesophis brasiliensis* | 0.190476 |
| *Micrelaps bicoloratus* | 0.188492 |
| *Atheris desaixi* | 0.180556 |
| *Diadophis punctatus* | 0.178571 |
| *Crotalus cerastes* | 0.174603 |
| *Telescopus dhara* | 0.173611 |
| *Oligodon modestus* | 0.172619 |
| *Oligodon venustus* | 0.678571 |
| *Typhlops sulcatus* | 0.117063 |
| *Typhlops jamaicensis* | 0.117063 |
| *Psammophis angolensis* | 0.115079 |
| *Crotalus willardi* | 0.113095 |
| *Geophis occabus* | 0.10119 |
| *Anilios polygrammicus* | 0.100198 |
| *Oxyrhopus petolarius* | 0.100198 |
| *Trimeresurus sichuanensis* | 0.100198 |
| *Oxyrhabdium leporinum* | 0.098214 |
| *Rhinoleptus koniagui* | 0.09623 |
| *Dipsas pratti* | 0.095238 |
| *Erythrolamprus breviceps* | 0.089286 |
| *Hydrophis platurus* | 0.089286 |
| *Anilios nigrescens* | 0.078373 |
| *Dieurostus dussumieri* | 0.069444 |
| *Atractus zidoki* | 0.069444 |
| *Platyceps florulentus* | 0.061508 |
| *Hemibungarus calligaster* | 0.060516 |
| *Hydrophis spiralis* | 0.059524 |
| *Trimeresurus popeiorum* | 0.046627 |
| *Lygophis lineatus* | 0.042659 |
| *Hemachatus haemachatus* | 0.039683 |
| *Dasypeltis atra* CAS201640 | 0.034722 |
| *Dipsadoboa shrevei* | 0.018849 |
| *Rhabdophis subminiatus* | 0.013889 |
| *Trimeresurus tibetanus* | 0.013889 |
| *Elaphe climacophora* | 0.013889 |
| *Micrurus hemprichii* | 0.005952 |
| *Morelia carinata* | 0.002976 |
